# Stereoselective Synthesis and Catalytical Application of Perillaldehyde-Based 3-Amino-1,2-diol Regioisomers

**DOI:** 10.3390/ijms25084325

**Published:** 2024-04-13

**Authors:** Márton Benedek Háznagy, Antal Csámpai, Imre Ugrai, Barnabás Molnár, Matti Haukka, Zsolt Szakonyi

**Affiliations:** 1 Institute of Pharmacognosy, University of Szeged, H-6720 Szeged, Hungary; haznagy.marton.benedek@szte.hu; 2 Institute of Pharmaceutical Chemistry, Interdisciplinary Excellence Center, University of Szeged, Eötvös utca 6, H-6720 Szeged, Hungary; ugrai.imre@gmail.com; 3 Institute of Chemistry, Eötvös Loránd University, P.O. Box 32, H-1518 Budapest, Hungary; antal.csampai@ttk.elte.hu; 4 Department of Molecular and Analytical Chemistry, Interdisciplinary Excellence Centre, University of Szeged, Dóm Tér 7-8, H-6720 Szeged, Hungary; barnabas.molnar@chem.u-szeged.hu; 5 Department of Chemistry, University of Jyväskylä, P.O. Box 35, 40351 Jyväskylä, Finland; matti.o.haukka@jyu.fi

**Keywords:** monoterpene, chiral, aminodiol, enantioselective, regioselective, catalyst, diethylzinc

## Abstract

A library of regioisomeric monoterpene-based aminodiols was synthesised and applied as chiral catalysts in the addition of diethylzinc to benzaldehyde. The synthesis of the first type of aminodiols was achieved starting from (−)-8,9-dihydroperillaldehyde via reductive amination, followed by Boc protection and dihydroxylation with the OsO_4_/NMO system. Separation of formed stereoisomers resulted in a library of aminodiol diastereoisomers. The library of regioisomeric analogues was obtained starting from (−)-8,9-dihydroperillic alcohol, which was transformed into a mixture of allylic trichloroacetamides via Overman rearrangement. Changing the protecting group to a Boc function, the protected enamines were subjected to dihydroxylation with the OsO_4_/NMO system, leading to a 71:16:13 mixture of diastereoisomers, which were separated, affording the three isomers in isolated form. The obtained primary aminodiols were transformed into secondary derivatives. The regioselectivity of the ring closure of the *N*-benzyl-substituted aminodiols with formaldehyde was also investigated, resulting in 1,3-oxazines in an exclusive manner. To explain the stability difference between diastereoisomeric 1,3-oxazines, a series of comparative theoretical modelling studies was carried out. The obtained potential catalysts were applied in the reaction of aromatic aldehydes and diethylzinc with moderate to good enantioselectivities (up to 94% *ee*), whereas the opposite chiral selectivity was observed between secondary aminodiols and their ring-closed 1,3-oxazine analogues.

## 1. Introduction

Chiral catalysis, that is, the development and application of new chiral catalysts, is an evergreen topic in the field of organic, applied, and pharmaceutical chemistry. In recent years, aminodiols and their *N*-heterocyclic analogues have proved to be important building blocks of new chiral catalysts or even complex bioactive molecules with significant biological activities [1,2,3,4]. Several aminodiol-based nucleoside analogues prepared recently have been shown to possess cardiovascular, cytostatic, and antiviral effects [5,6,7,8,9,10]. The Abbott aminodiol, a useful building block for the synthesis of the potent renin inhibitors Zankiren^®^ and Enalkiren^®^, was introduced into the therapy of hypertension [11,12]. Aminodiols can also exert antidepressive activity. For example, (*S*,*S*)-reboxetine, a selective norepinephrine reuptake inhibitor, was approved in many countries for the treatment of unipolar depression [13]. Other aminodiols may serve as starting materials for the synthesis of biologically active natural compounds. For example, cytoxazone, a microbial metabolite isolated from Streptomyces species, is a selective modulator of the secretion of TH2 cytokine [14,15].

Besides their biological interest, aminodiols have also been applied as starting materials in asymmetric syntheses or as chiral auxiliaries and ligands in enantioselective transformations [1,16,17,18]. To develop new, efficient, and commercially available chiral catalysts, chiral natural products including (+)- and (−)-α-pinene [19,20], (−)-nopinone [21], (+)-carene [22,23], (+)-sabinol [24], (−)-pulegone [25], or camphore and fenchon [26] can serve as important starting materials for the synthesis of aminodiols. Monoterpene-based aminodiols have been demonstrated to be excellent chiral auxiliaries in a wide range of stereoselective transformations, including Grignard addition [27,28] and intramolecular radical cyclisation [29].

In the present study, our aim was to prepare a library of diastereoisomeric and regioisomeric analogues of 3-amino-1,2-diols derived from commercially available (−)-perillaldehyde as the chiral source. The study of the steric effect of both the aminodiol functionality and nitrogen substituents was also planned, applying the resulting trifunctional potential catalysts in the enantioselective addition of diethylzinc on benzaldehyde as a model reaction.

## 2. Results

### 2.1. Synthesis of Aminodiols via Reductive Amination of Perillaldehyde

**Scheme 1:**
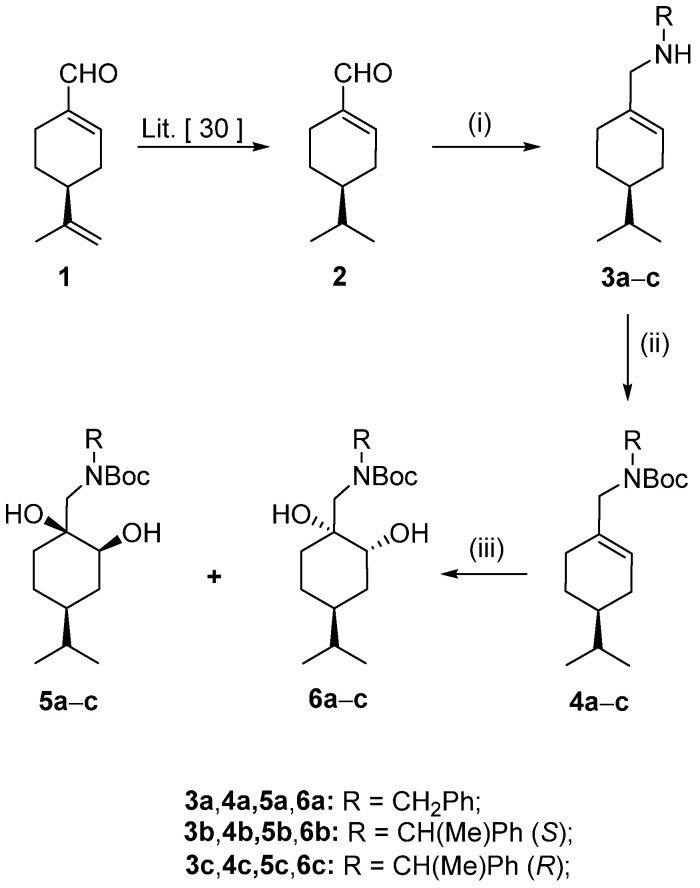
Synthesis of BOC-protected aminodiols. (i) (1) 1.05 eq. R-NH_2_ (R = a: CH_2_Ph, b: CH(Me)Ph (*S*), c: CH(Me)(*R*)), dry EtOH, rt, 2 h; (2) 3 eq. NaBH_4_, dry EtOH, rt, 2 h, 66–86%; (ii) Boc_2_O, TEA, DMAP, THF; rt, 90–95%; (iii) 50% NMO/H_2_O, cat. 2% OsO_4_ in *tert*-BuOH, acetone/H_2_O, rt, 2 h, 25–48%, **5**:**6** = 1:1.

In our first approach, chiral aminodiols were synthesised from readily available (−)-(*S*)-peryllaldehyde **1**, starting with a reduction according to a literature method using Pt/C catalyst in an *n*-hexane/EtOAc solvent system to obtain **2** [30]. ^1^H-NMR monitoring was applied during the reaction to set up the appropriate conditions to afford the desired product. The resulting aldehyde was transformed into allylamines in two steps by reductive amination with (*S*)- and (*R*)-1-phenylethylamine **3a**–**c** in good yield. The amine moieties were protected by the BOC (*tert*-butyloxycarbonyl) group, and **4a**–**c** were hydroxylated in a stereoselective manner with the OsO_4_/NMO catalytic system to produce *cis*-vicinal aminodiols **5a**–**c** (1*S*,2*S*) and **6a**–**c** (1*R*,2*R*), whereas the obtained diastereomers in 1:1 mixture were successfully separated by column chromatography (Scheme 1).

**Scheme 2:**
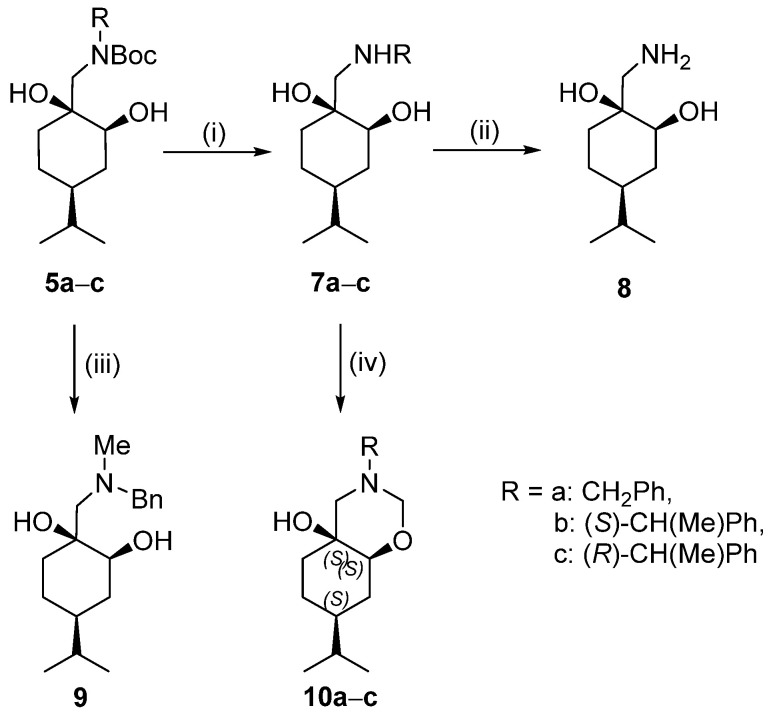
Preparation of aminodiol derivatives starting from **5a**–**c**: (i) Et_2_O, 18% HCl, overnight,, 82–88%; (ii) 10% Pd/C, *n*-hexane/EtOAc 1:2 mixture, 1 atm, H_2_, rt, 5 h, 35%; (iii) R = Bn, 3 eq. LiAlH_4_, THF, reflux, 6 h, 60%; (iv) 35% aq. CH_2_O, Et_2_O, rt, 3 h 70–96%.

The protecting groups of **5a**–**c** were removed by TFA treatment, providing **7a**–**c**. Debenzylation of **7a** was then carried out to reach primary aminodiol **8**. LiAlH_4_ reduction of **5a** resulted in *N*-methyl-*N*-benzyl derivative **9**. Furthermore, the cyclisation reaction of **7a**–**c** was examined using formaldehyde as the aldehyde source. It is worth noting that in all cases, the six-membered 1,3-oxazine derivatives were reached, while the formation of a spirooxazolidine ring could not be determined in the crude product (Scheme 2).

**Scheme 3:**
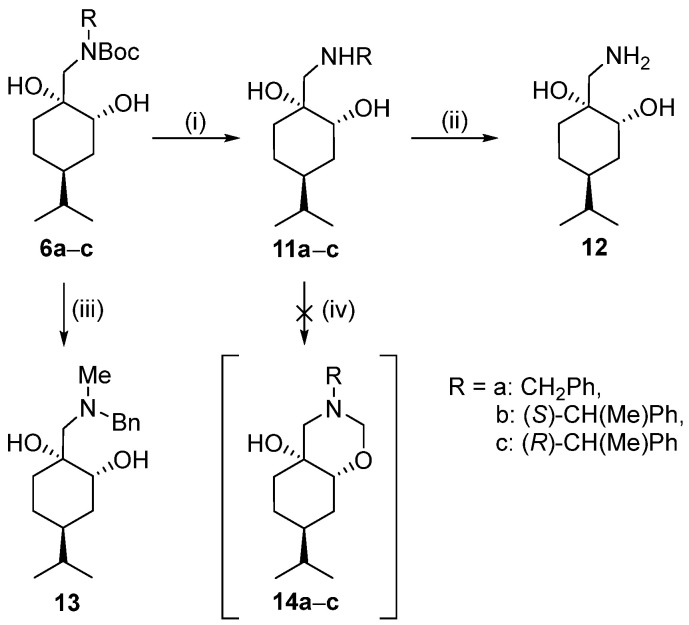
Preparation of aminodiol derivatives starting from **6a**–**c**: (i) TFA, DCM, rt, 2 h, 78–97%; (ii) 10 % Pd/C, *n*-hexane/EtOAc 1:1 mixture, 1 atm, H_2_, rt, 5 h, 45%; (iii) R = Bn, 3 eq. LiAlH_4_, THF, reflux, 6 h, 58%; (iv) 35% aq. CH_2_O, Et_2_O, rt, 3 h—product could not be isolated.

**Figure 1:**
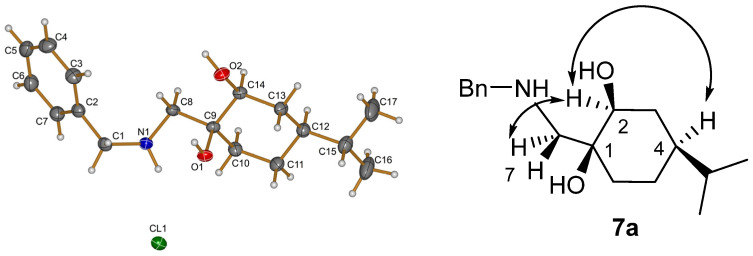
Structural determination of aminodiol **7a** by X-ray crystallography and NOESY experiments.

The relative configuration of diastereoisomer **7a** was determined by means of NOESY experiments: clear NOE signals were observed between the H-2 and H-4, H-2 and H-7, as well as the OH-1 and OH-2 protons (Figure 1). Debenzylation via hydrogenolysis of compounds **7a**–**7c** over Pd/C in MeOH resulted in primary aminodiol **8** yielding the same compound with a moderate yield (Scheme 3). Besides NOESY experiments, the structure was also elucidated by X-ray crystallography (Figure 1).

To compare the reactivity and ring closing ability of **7a**–**c** with their diastereoisomers, the above-mentioned reactions were accomplished with the (1*R*,2*R*) **6a**–**c** compounds as well. The deprotected **11a**–**c**, the *N*-methyl-*N*-benzyl **13**, and the primary aminodiol **12** products were obtained similarly to the diastereoisomeric analogues, while in the case of ring closure with formaldehyde, no ring-closed product could be isolated (Scheme 3).

**Figure 2:**
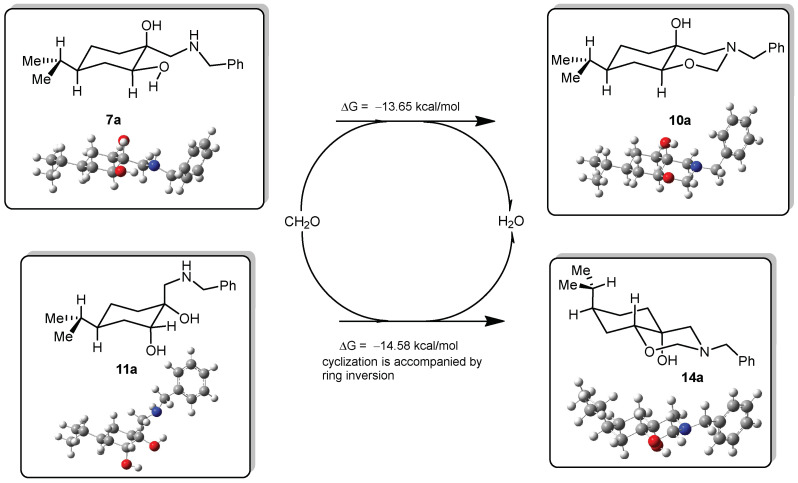
Calculated thermodynamic data of formaldehyde-mediated cyclisation reactions of diastereomeric aminodiols **7a** and **14a**.

To rationalise the spectacular difference observed in the tendency of **7a** and **11a** to undergo formaldehyde-mediated annulation to form 1,3-oxazine, we carried out a series of comparative theoretical modelling studies (details can be found in Section 4). Although fused 1,3-oxazine **14a** could not be isolated, presumably due to its decomposition during chromatographic purification on silica, the calculated changes in Gibbs free energy unambiguously indicate that both cyclisations (**7a** to **10a** and **11a** to **14a**) are thermodynamically feasible processes (Figure 2). It must be noted here that cyclisation of **11a** to **14a** is accompanied by the inversion of the fused cyclohexane ring, forcing the bulky isopropyl group into an axial position of the resulting *trans*-fused rigid bicyclic framework.

**Figure 3:**
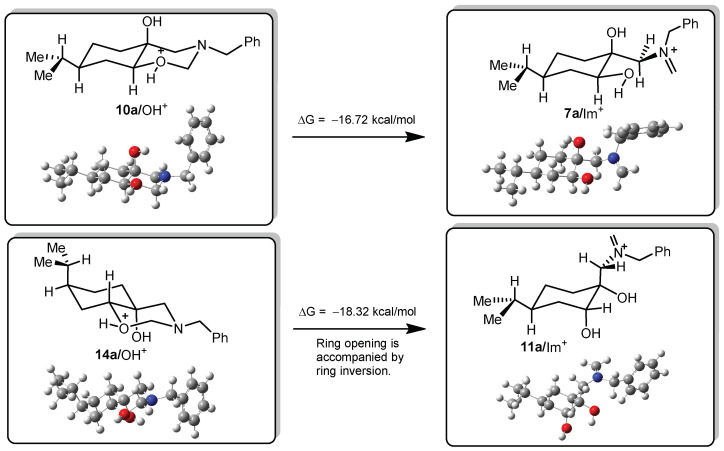
Calculated thermodynamics of acid-promoted iminium-generating ring opening of *O*-protonated diastereomeric cyclohexane-fused 1,3-oxazines **10a**/OH^+^ and **14a**/OH^+^, as assumed to be effected during chromatographic workup on silica. The highly exothermic energetics disclosed for both ring-opening reactions analysed here clearly indicate that formaldehyde-mediated annulations cannot proceed via iminium intermediates.

Since TLC monitoring indicated the transitional formation of **14a**, we assume that its isolation was prevented by a hydrolytic decomposition taking place on the acidic silica surface during the chromatographic purification of the crude product. This view gains support from the structural characteristics and energetic data of further comparative DFT modelling studies on the iminium-generating ring opening of the diastereomeric *O*-protonated oxazines **10a**/OH^+^ → **10a**/Im^+^ and **14a**/OH^+^ → **14a**/Im^+^ (Figure 3). The energetic results unambiguously show that both ring fissions are thermodynamically feasible. However, the second process is accompanied by the inversion of the cyclohexane ring with the reorientation of the isopropyl group into equatorial position and a significant spatial separation of the iminium moiety and the axially oriented hydroxyl group in **11a**/Im^+^. The latter is expected to undergo precursor-regenerating hydrolysis (**11a**/Im^+^ + H_2_O → **11a** + CH_2_O) rather than ring inversion-enabled recyclisation to **14a**/OH^+^. On the other hand, upon neutralisation of the medium, sequential recyclisation and *O*-deprotonation of **7a**/Im^+^ can be considered as a smooth chemical route proceeding via the interaction of proximal nucleophilic and electrophilic functional groups on the highly rigid cyclohexane ring to reconstruct the isolable fused oxazine **10a**.

**Figure 4:**
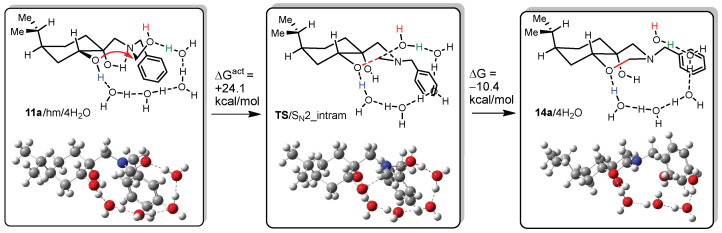
Calculated kinetics and thermodynamics of the cyclisation of the ring-inverted *N*-hydroxymethyl derivative **11a** taking place with the S_N_2 reaction promoted by a cascade of proton shifts along the H-bond-connected cluster chain of four water molecules.

The aforementioned results, including the highly exothermic energetics disclosed for both ring-opening reactions analysed above, clearly indicate that formaldehyde-mediated annulations cannot proceed via iminium-type intermediates. Therefore, we propose here an alternative pathway for the formation of fused 1,3-oxazines as exemplified by theoretically modelling the one-step ring closure of the ring-inverted *N*-hydroxymethylated intermediate coupled to a four-membered water chain cluster (**11a**/hm/4H_2_O → **14a**/4H_2_O, Figure 4). Accordingly, we assume that a H-bonded optimally arranged linear cluster of four water molecules strongly facilitates the kinetically and thermodynamically feasible one-step intramolecular S_N_2 reaction. It simultaneously activates the nucleophilic skeletal hydroxyl group and the leaving hydroxyl group in the pending hemiaminal residue by a concerted domino proton-migration along the H-bond-coupled chain of water molecules. As for the size of the linear H-bond transferring cluster, we found that it is the involvement of the four water molecules that allows the construction of such an ideal molecular architecture, which is suitable for undergoing ring closure via a concerted mechanism. It is also reasonable to rationalise the formation of **10a** along this pathway. The validity of this and closely related reaction pathways is further supported by other well-documented examples [31,32].

### 2.2. Synthesis of Aminodiols via Overman Rearrangement of Perillyl Alcohol

(−)-(*S*)-Dihydroperyllaldehyde **2** proved to be an excellent starting material not only for the preparation of aminodiols **5**–**13** but also for the synthesis of regioisomeric 3-amino-1,2-diols, where the amino function is connected directly to the cyclohexane ring. Reduction of **2** served 8,9-dihydroperillic alcohol **15**. The Overmann rearrangement of **15** led to the diastereoisomeric mixture of *N*-trichloroacetyl-protected allyl amines in a ratio of 1*R*:1*S* = 85:15 based on data determined by GC (Chirasil-DEX CB column) analysis of the crude product. The relative configuration of major product **18a**, based on the NOESY spectra, was found to be similar to the literature example [20,33,34].

**Scheme 4:**
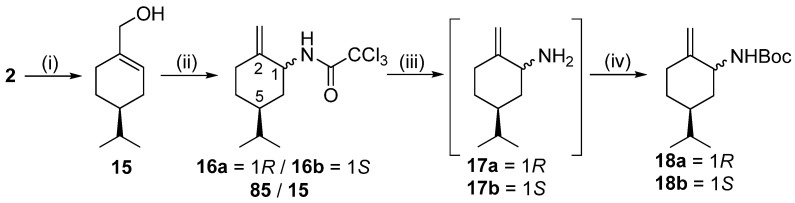
Synthesis of the regioisomer allylamines via Overman rearrangement: (i) NaBH_4_, MeOH, 0 °C to rt, 1 h, 95%; (ii) (1) CCl_3_CN, dry DCM, DBU, Ar atm, 0 °C to rt, 2 h; (2) toluene, Ar atm, 130 °C, 24 h, 72%, *de* = 70% for **16a**; (iii) (1) 5M NaOH/H_2_O, EtOH/DCM 2/1, 50 °C, 15 h; (iv) Boc_2_O, cat. DMAP, THF, TEA, rt, 12 h, 59%.

Since separation of the diastereomers was not feasible, the mixture of **16a** and **16b** was reacted in the following steps. The trichloroacetyl protecting group was exchanged for easily handlable Boc by alkaline hydrolysis followed by BOC_2_O treatment, affording **18a** and **18b**. Despite chromatographic efforts, the resulting diastereoisomers could not be separated. Therefore, the mixture of diastereoisomers was used in the next step (Scheme 4).

**Figure 5:**
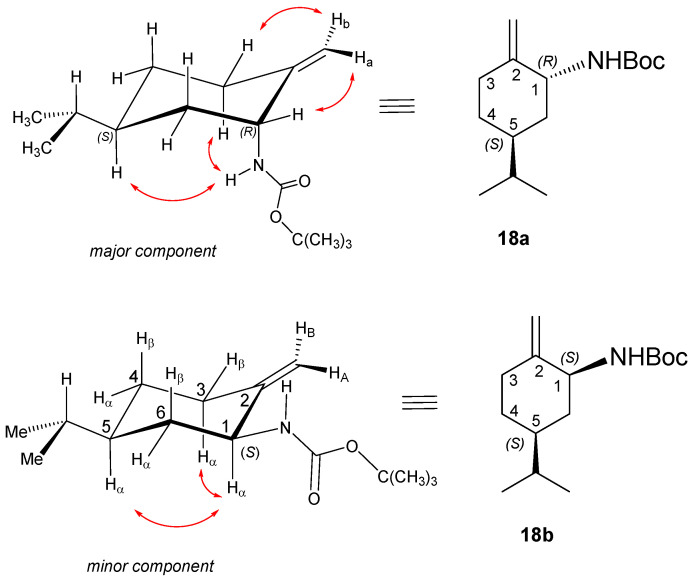
Structural determination of the diastereoisomers **18a** and **18b** by NOESY experiments.

The relative configuration of major diastereoisomer **18a** was determined by means of NOESY experiments. Clear NOE signals were observed between the H-2 and the methylene H_a_, between NH and H-3_ax_ and H-5, as well as between the H-3_eq_ and the methylene H_b_ protons (Figure 5).

**Scheme 5:**
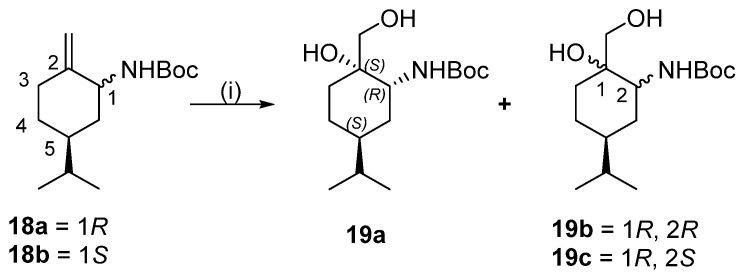
Dihydroxylation of the protected allylamine mixture (i) 50% NMO/H_2_O, cat. 2% OsO_4_ in *tert*-BuOH, rt, 72 h, **19a**: 48%, **19b** and **19c**: 33%.

The mixture of allyl amines was dihydroxylated with the OsO_4_/NMO oxidiser system, resulting in a mixture of diasteroisomers (**19a**–**c**) in a ratio **19a**:**19b**:**19c** = 71:16:13. At this stage, isomer **19a** could be isolated by column chromatography, while isomers **19b** and **19c** were obtained as an inseparable mixture (Scheme 5).

**Scheme 6:**
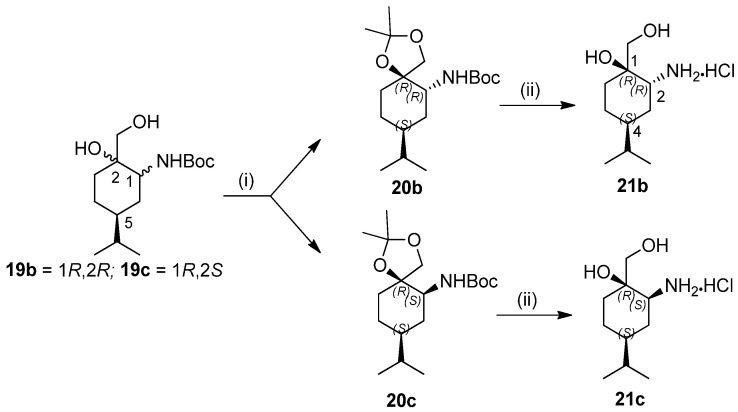
Separation of **19b** and **19c** via formation of acetonides. Reactions and conditions: (i) (a) dry acetone, cat. PTSA, Ar atm, rt; (b) flash chromatography, **20b**: 56%, **20c**: 18%; (ii) 10% HCl, Et_2_O, rt, 24 h, **21b**: 86%, **21c**: 80%.

**Figure 6:**
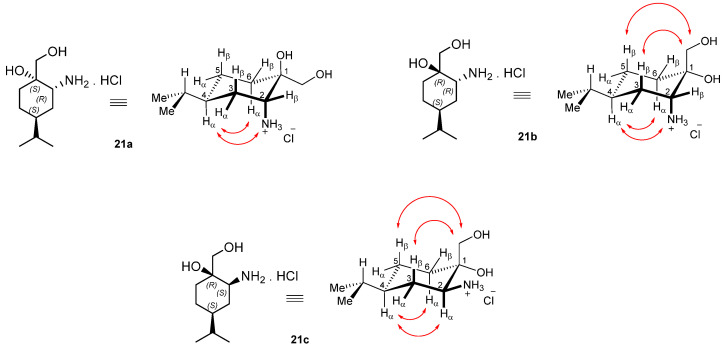
Structural determination of diastereoisomers **21a**–**c** by NOESY experiments.

To isolate the other isomers too, acetal derivatives **20b** and **20c** were prepared with acetone from the two-component mixture, which allowed successful separation of **20b** and **20c**. BOC and acetal protective groups were removed in a single step under acidic conditions. The obtained relative configurations of epimers were determined via 2D NMR techniques (COSY, HMBC, HSQC, and NOESY) (Scheme 6 and Figure 6).

The stereostructures of **21a**, **21b**, and **21c** and the two diastereomeric components **18a** (major isomer) and **18b** (minor isomer) were determined by ^1^H- and ^13^C-NMR spectroscopy. The assignment of the ^1^H- and ^13^C-NMR signals was supported by 2D-COSY, 2D-HSQC, 2D-HMBC, and 2D-NOESY experiments. The stereostructure of **18b**/minor was identified on the basis of its separated diagnostic signals and cross peaks discernible in the 1D and 2D spectra registered on the isolated ca. 85/15 mixture (^1^H-NMR) of **18a**/major and **18b**/*minor* (see later). In accordance with the general expectations, the isopropyl group was in the equatorial position in all of these cyclohexane derivatives, as evidenced by the NOE interactions detected between the axially positioned proton pair 4H_α_/6H_α_. A further interaction involving 4H_α_ and 2H_α_ was in accordance with the equatorial position of the NH_3_^+^ group on the rigid, six-membered skeleton in **21c**. Providing additional evidence for their relative configuration, characteristic NOE interactions in **21a** and **21b** were disclosed between 4H_α_ and the NH_3_^+^ group. In **18a**/*major*, the axial position of the Boc-protected amino group was confirmed by the NOE interaction between the NH- and 4H_α_ protons. Providing further evidence for its stereostructure with the axially positioned Boc-protected amino group, further interactions were also detected in the NOESY spectrum of **18a**/*major* between proton pairs H_A_/1H_β_ and H_B_/3H_β_ (Figure 5). The axial position of the hydroxymethyl group in **21b** and **21c** was confirmed by the NOE interaction of its protons with 5H_β_ and 3H_β_. In **18b**/*minor,* the equatorial position of the Boc-protected amino group was reflected in the NOE interaction detected between the axially positioned proton pair 1H_α_/3H_α_ producing signals of well-resolved coupling patterns featuring significant splits due to characteristic 1,2-diaxial coupling interactions. Further supporting the relative configuration of the two diastereomers, the marked upfield shift of the ^13^C signal of terminal methylene (=CH_2_) in **18b**/*minor* relative to that measured for **18a**/*major* (104.9 ppm and 149.0 ppm, respectively) referred to a local steric congestion associated with the proximity of the bulky Boc-protected amino group and the exocyclic olefin residue on rigid ring system of **18b**/*minor* (Figure 5).

**Scheme 7:**
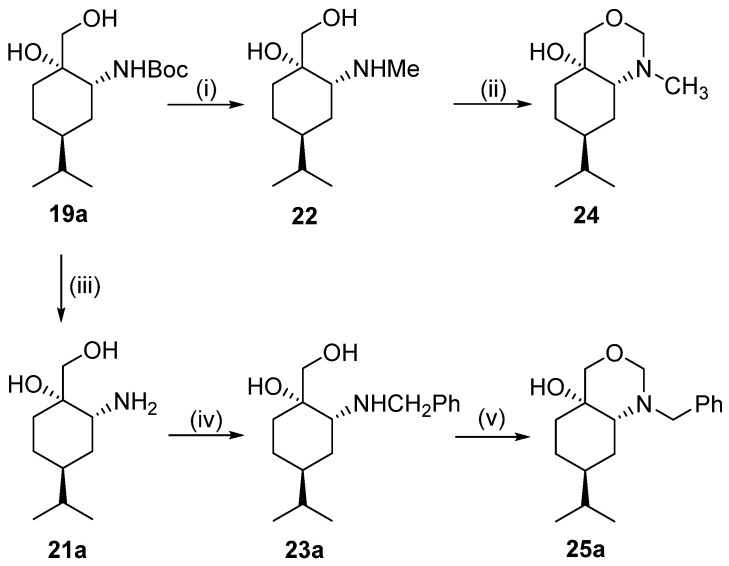
Preparation of aminodiol library. Reactions and conditions: (i) 3 eq. LiAlH_4_, THF, rt, 2 h, 65%; (ii) 40% CH_2_O, Et_2_O, rt, 1 h, 66%; (iii) 10% HCl, Et_2_O, rt, 24 h, 86%; (iv) (1) benzaldehyde, dry EtOH, rt, 2 h; (2) NaBH_4_, EtOH, rt, 6 h, 54%; (v) 40% CH_2_O sol., Et_2_O, rt, 1 h, 89%.

Reduction of **19a**, performed with LiAlH_4_, served the *N*-methylaminodiol **22**, while deprotection of **19a** and reductive alkylation of the formed **21a** with benzaldehyde in the presence of NaBH_4_ provided *N*-benzyl derivative **23a**. Ring closure of aminodiols with a secondary amino function was carried out with formaldehyde. Interestingly, only the six-membered 1,3-oxazoline ring systems **24** and **25a** were formed in both cases (Scheme 7).

**Scheme 8:**
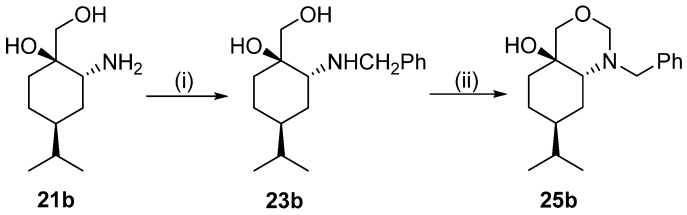
Synthesis of aminodiol library. Reaction conditions: (i) (1) PhCHO, dry EtOH, rt, 2 h; (2) NaBH_4_, dry EtOH, rt, 6 h, 89% overall; (ii) 40% CH_2_O, Et_2_O, rt, 1 h, 80%.

Similar to the above process, starting from the liberated base **21b**, *N*-benzyl aminodiol **23b** and its ring-closed 1,3-oxazoline ring system derivative **25b** were produced (Scheme 8).

### 2.3. Application of Aminodiol Libraries as Catalysts in the Addition Reaction of Diethyzinc to Benzaldehyde

**Scheme 9:**
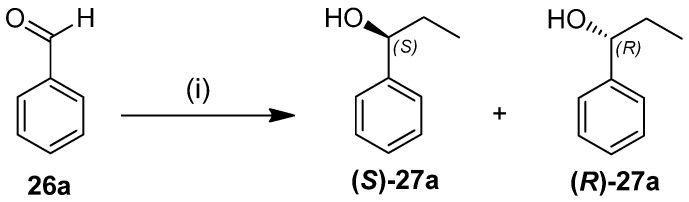
Model reaction of diethylzinc with benzaldehyde **26a**. Reactions and conditions: (i) Et_2_Zn, 10 mol% catalyst, *n*-hexane, Ar atm., rt, 20 h, 51–92%.

The prepared aminodiol derivatives were applied as chiral catalysts in the enantioselective addition of diethylzinc to benzaldehyde (**26**) as a model reaction, where (*S*)- and (*R*)-1-phenyl-1-propanol (**(*S*)-27** and **(*R*)-27**) were formed as products (Scheme 9).

Our results are presented in Table 1. The enantiomeric ratio of the *S* and *R* isomers was determined by a chiral GC CHIRASIL-DEX CB column according to literature methods [35,36,37].

In these addition reactions, low to excellent catalytic activities were observed. Interestingly, aminodiol diastereoisomers provided opposite chiral induction, and **7a**–**c** isomers proved better catalysts with *S* selectivity than **11a**–**c** (best *ee* = 68% for **7a**). The best catalytic activity was observed in the case of **10a**, one of the 1,3-oxazoline *N*-benzyl derivatives, which afforded the best *ee* value (94% *ee*) with *R* selectivity (Table 1, entry 6, *ee* = 94%). Interestingly, oxazine **25a** (*ee* = 62%, entry 21) promotes the *R* absolute configuration, whereas the diasteroisomer primer aminodiol **21b** (*ee* = 60%, entry 15) provides the *S* configuration. Furthermore, all tested primer aminodiols (**8**, **12**, **21a**, **21b** and **21c**) promote the *S* configuration.

With the best catalyst for (*S*)-selectivity (**7a**) and for (*R*)-selectivity (**10a**), the diethylzinc addition reaction was extended to further aromatic aldehydes (Scheme 10). Our results are presented in Table 2. The enantiomeric purities of the 1-aryl-1-propanols obtained were determined by chiral HPLC analysis on a Chiralcel OD-H column (see Supporting Information Figures S147–S162), according to literature methods [23].

## 3. Discussion

Starting from natural (−)-(*S*)-peryllaldehyde (**1**), a monoterpene-based aminodiol library with five sub-libraries containing diastereo- and regioisomeric aminodiols was created, and the reaction of these aminodiols with formaldehyde resulted in bicyclic monoterpene-fused condensed 1,3-oxazines through stereochemistry-dependent ring closure. Molecular modelling was applied to explain this phenomenon. The aminodiols and their ring-closed derivatives were used as chiral catalysts in the enantioselective addition of diethylzinc to benzaldehyde from moderate to excellent activities. Comparing the regioisomers and diastereoisomers, aminodiols bearing vicinal hydroxyl groups on the cyclohexane skeleton with the (1*S*,2*S*,4*S*) configuration proved to be more efficient catalysts mainly with *S* selectivity. The ring closure of these aminodiols also showed the best but opposite (*R*) catalytic activity, when the obtained 1,3-oxazines **10a**–**c** were applied in the addition of diethylzinc to benzaldehyde. The catalytic activity study was extended by applying the best catalyst for *meta* and *para* substituted aromatic aldehydes and, in the case of p-tolylaldehy aminodiol, **7a** gave (*R*) selectivity. It is interesting to note that in the same enantiomeric library, we could find catalysts for both (*S*) and (*R*) selectivities. The 1,3-oxazines obtained proved to be excellent catalysts in the additions of diethylzinc to aromatic aldehydes, probably as a consequence of their conformationally constrained structures. Regioisomeric aminodiols and 1,3-oxazines proved less effective catalysts in the applied model reaction with similar selectivities.

## 4. Materials and Methods

^1^H NMR and ^13^C NMR spectra were recorded with a Bruker Avance DRX 400 [400 and 100 MHz, respectively, *δ* = 0 ppm (tetramethylsilane)] and Bruker Avance DRX 500 [500 and 125 MHZ, respectively, *δ* = 0 ppm (tetramethylsilane)] (both Bruker Biospin, Karlsruhe, Baden Württemberg, Germany). Chemical shifts (*δ*) were expressed in ppm relative to tetramethylsilane as an internal reference. *J* values were given in Hz.. GC measurements were made with a PerkinElmer Autiosystem KL GC consisting of a flame ionisation detector and a Turbochrom Workstation data system (PerkinElmer Corporation, Norwalk, CT, USA). Separation of the enantiomers of the *O*-acetyl derivatives of 1-phenyl-1-propanol was performed on a CHIRASIL-DEX CB column (2500 × 0.265 mm inner diameter, Agilent Technologies, Inc., Santa Clara, CA, USA; see Supporting Information, Figures S147–S156). Chiral HPLC analysis was performed on a Chiralcel OD-H column (250 × 4.6 mm, see Supporting Information, Figures S157–S162). UV detection was monitored at 210 nm or at 254 nm.

Optical rotations were performed with a PerkinElmer 341 polarimeter (PerkinElmer Inc., Shelton, CT, USA). Melting points were determined with a Kofler apparatus (Nagema, Dresden, Germany). Chromatographic separations were performed with Merck Kieselgel 60 (230–400 mesh ASTM, Merck Co., Darmstadt, Germany). Reactions were monitored with Merck Kieselgel 60 F254-precoated TLC plates (0.25 mm thickness, Merck Co., Darmstadt, Germany). All ^1^H-, ^13^C- NMR, HMQC, HMBC, and NOESY spectra are found in the Supporting Information (see Figures S1–S144).

X-Ray structural determination of compound **7a** was determined by a Rigaku Oxford Diffraction Supernova diffractometer ((Rigaku Oxford Diffraction, Yarnton, UK) using Cu Kα radiation. The *CrysAlisPro* software package (version: 1.171.37.35) was used for cell refinement and data reduction (see Supporting Information part, Table S1).

**Starting materials:** (−)-(*S*)-peryllaldehyde (**1**) was sourced commercially from Merck Co (Merck Co., Darmstadt, Germany). All chemicals and solvents were used as supplied. THF and toluene were dried over Na wire. (*S*)-4-Isopropylcyclohex-1-ene carbaldehyde (**2**) and (*S*)-4-isopropylcyclohex-1-ene-1-ylmethanol (**15**) were prepared according to literature procedures, and all spectroscopic data were similar to those reported therein [30].

**General method for the reductive amination of 2 with amines**

The appropriate benzylamine (1.05 equiv, 27.6 mmol) was added to a stirred solution of compound **2** (4.00 g, 26.28 mmol) in dry EtOH (200 mL). The mixture was stirred at room temperature for 1 h. Afterwards, the solvent was evaporated, and the residue was redissolved in dry EtOH (200 mL) and then stirred for an additional hour. NaBH_4_ (2.98 g, 78.84 mmol) was added in small portions to the reaction mixture, which was stirred at room temperature (for compound **3a**) or under reflux (for compounds **3b** and **3c**) for 2 h. Next, the solvent was removed under vacuum, and the crude product was dissolved in ice-cold H_2_O (70 mL) and extracted with dichloromethane (DCM) (3 × 100 mL). The combined organic phase was dried (Na_2_SO_4_), filtered, and evaporated. The crude product was purified via column chromatography on silica gel by applying toluene/EtOH 9:1, and then hydrochloride salts of the compounds were formed to yield **3a**–**c**.

(*S*)-*N*-Benzyl-1-(4-Isopropylcyclohex-1-en-1-yl)methanamine hydrochloride **3a**

Prepared with benzylamine according to the general method. Yield: 6.32 g (86%); white crystals; m.p.: 178–185 °C; [α${]}_{20}^{D}$ = −92 (c 0.25, MeOH). ^1^H NMR (DMSO-*d*_6_, 400.1 MHz): δ = 0.86 (3H, d, *J* = 6.8 Hz), 0.87 (3H, d, *J* = 6.8 Hz), 1.10–1.27 (2H, m), 1.41–1.51 (1H, m), 1.67–1.79 (2H, m), 1.99–2.17 (3H, m), 3.39 (2H, br s), 4.04 (2H, br s), 5.80–5.85 (1H, m), 7.38–7.45 (3H, m), 7.55–7.61 (2H, m), 9.50 (2H, br s); ^13^C NMR (100.6 MHz, DMSO-*d*_6_): δ = 20.3, 20.6, 26.2, 28.0, 29.2, 31.2, 32.4, 39.8, 50.1, 52.3, 129.4, 129.7, 129.9, 130.4, 131.1, 132.8.; HR-MS (ESI): *m*/*z* calcd for C_17_H_26_N [M + H]^+^: 244.20598; found: 244.20532.

(*S*)-*N*-(((*S*)-4-Isopropylcyclohex-1-en-1-yl)methyl)-1-phenylethanamine hydrochloride **3b**

Prepared with (*S*)-1-phenylethylamine according to the general method. Yield: 5.64 g (73%), white crystals, m.p.: 208–212 °C; [α${]}_{20}^{D}$ = −43 (c 0.25, MeOH); ^1^H NMR (DMSO-*d*_6_, 400.1 MHz): δ = 0.85 (3H, d, *J* = 6.8 Hz), 0.86 (3H, d, *J* = 6.8 Hz), 1.08–1.24 (2H, m), 1.41–1.50 (1H, m), 1.56 (3H, d, *J* = 6.6 Hz), 1.64–1.77 (2H, m), 1.88–2.14 (3H, m), 3.08 (1H, d, *J* = 13.3 Hz), 3.24 (1H, d, *J* = 13.4 Hz), 4.13–4.26 (1H, m), 5.65–5.73 (1H, m), 7.34–7.46 (3H, m), 7.50–7.61 (2H, m), 9.24 (2H, br s); ^13^C NMR (100.6 MHz, DMSO-*d*_6_): δ = 20.3, 20.5, 20.6, 26.3, 28.0, 29.1, 32.4, 39.8, 51.3, 57.7, 128.6, 129.0, 129.4, 129.7, 138.4; HR-MS (ESI): *m*/*z* calcd for C_18_H_28_N [M + H]^+^: 258.22163; found: 258.22098.

(*R*)-*N*-(((*S*)-4-Isopropylcyclohex-1-en-1-yl)methyl)-1-phenylethanamine hydrochloride **3c**

Prepared with (*R*)-1-phenylethylamine according to the general method. Yield: 5.10 g (66%), white crystals, m.p.: 204–206 °C; [α${]}_{20}^{D}$ = −76 (c 0.25 MeOH); ^1^H NMR (DMSO-*d*_6_, 400.1 MHz): δ = 0.86 (3H, d, *J* = 6.7 Hz), 0.87 (3H, d, *J* = 6.7 Hz), 1.02–1.14 (1H, m), 1.16–1.26 (1H, m), 1.40–1.51 (1H, m), 1.61 (3H, d, *J* = 6.8 Hz), 1.63–1.75 (2H, m), 1.97–2.07 (3H, m), 3.11 (1H, d, *J* = 13.5 Hz), 3.27 (1H, d, *J* = 13.5 Hz), 4.26 (1H, dd, *J* = 6.8, 13.8 Hz), 5.66–5.73 (1H, m), 7.35–7.47 (3H, m), 7.56–7.65 (2H, m), 9.30 (1H, br s), 9.73 (1H, br s); ^13^C NMR (100.6 MHz, DMSO-*d*_6_): δ = 20.3, 20.6, 26.2, 28.0, 29.2, 31.2, 32.4, 39.8, 50.1, 52.3, 129.4, 129.7, 129.9, 130.4, 131.1, 132.8; HR-MS (ESI): *m*/*z* calcd for C_18_H_28_N [M + H]^+^: 258.22163; found: 258.22099.

**General procedure for *tert*-butyloxycarbonyl (BOC) protection of compounds 3a–c**

To a stirred solution of the liberated bases of allylamines **3a**–**c** (12 mmol) in dry THF (30 mL), di-*tert*-butyl dicarbonate (2.88 g, 13.2 mmol for **4a**; 5.76 g, 26.4 mmol for **4b**, and 3.56 g, 16.32 mmol for **4c**), TEA (3.64 g 36 mmol), and a catalytic amount of DMAP (0.15 g, 1.2 mmol) were added. The mixture was stirred overnight at rt. After completion of the reaction, indicated by means of TLC, the solvent was evaporated. The crude product was purified by column chromatography on silica gel by using *n*-hexane/EtOAc 9:1 for **4a**, *n*-hexane/EtOAc 19:1 for **4b**, or *n*-hexane/Et_2_O 19:1 for **4c**.

(*S*)-*tert*-Butyl benzyl((4-isopropylcyclohex-1-en-1-yl)methyl)carbamate **4a**

Prepared from **3a** according to the general method. Yield: product: 3.92 g (95%) (mixture of two rotamers in CDCl_3_); colourless oil, [α${]}_{20}^{D}$ = −55 (c 0.25, MeOH). ^1^H NMR (CDCl_3_, 400.1 MHz): δ = 0.88 (3H, d, *J* = 6.8 Hz), 0.89 (3H, d, *J* = 6.8 Hz), 1.08–1.31 (2H, m), 1.40–1.51 (1H, m, overlapped with s), 1.46 (9H, s), 1.68–1.79 (2H, m), 1.82–2.08 (3H, m), 3.57–3.85 (2H, m), 4.26–4.46 (2H, m), 5.41–5.49 (1H, m), 7.16–7.34 (5H, m); ^13^C NMR (100.6 MHz, CDCl_3_): δ = 19.8, 19.9, 20.1, 24.1, 26.2, 26.9, 28.6, 28.9, 29.5, 31.3, 32.3, 33.0, 40.2, 44.3, 48.6, 49.1, 51.6, 51.9, 79.7, 124.3, 126.7, 127.1, 127.6, 128.2, 128.5, 133.7, 138.7, 156.1; HR-MS (ESI): *m*/*z* calcd for C_18_H_26_NO_2_ [M − CH(CH_3_)_3_ + H + H]^+^: 288.19581; found: 288.19506.

*tert*-Butyl (((*S*)-4-isopropylcyclohex-1-en-1-yl)methyl)((*S*)-1-phenylethyl)carbamate **(4b)**

Prepared from **3b** according to the general method. Yield: 3.99 g (93%), colourless oil, [α${]}_{20}^{D}$ = −120 (c 0.25, MeOH) ^1^H NMR (DMSO-*d*_6_, 400.1 MHz): δ = 0.83 (3H, d, *J* = 6.1 Hz), 0.84 (3H, d, *J* = 6.1 Hz), 0.93–0.95 (1H, m), 1.09–1.19 (1H, m), 1.33 (9H, s), 1.35–1.53 (2H, m), 1.46 (3H, d, *J* = 7.0 Hz), 1.56–1.70 (2H, m), 1.74–1.96 (3H, m), 3.60 (2H, br s), 5.35 (1H, s), 7.19–7.36 (5H, m); ^13^C NMR (100.6 MHz, DMSO-*d*_6_): δ = 17.9, 20.0, 20.2, 26.0, 26.8, 28.5, 28.6, 32.1, 32.8, 40.1, 44.0, 53.4, 79.0, 127.2, 127.3, 128.5, 135.2, 142.2, 155.4. HR-MS (ESI): *m*/*z* calcd for C_19_H_28_NO_2_ [M − CH(CH_3_)_3_ + H + H]^+^: 302.21146; found: 302.21089.

*tert*-Butyl (((*S*)-4-isopropylcyclohex-1-en-1-yl)methyl)((*R*)-1-phenylethyl)carbamate **4c**

Prepared from **3c** according to the general method. Yield: 3.86 g (90%) colourless oil, [α${]}_{20}^{D}$ = +4 (c 0.25, MeOH) ^1^H NMR (DMSO-*d*_6_, 400.1 MHz): δ = 0.83 (3H, d, *J* = 6.1 Hz), 0.84 (3H, d, *J* = 6.1 Hz), 0.93–0.95 (1H, m), 1.09–1.19 (1H, m), 1.33 (9H, s), 1.35–1.53 (2H, m), 1.46 (3H, d, *J* = 7.0 Hz), 1.56–1.70 (2H, m), 1.74–1.96 (3H, m), 3.60 (2H, br s), 5.35 (1H, s), 7.19–7.36 (5H, m); ^13^C NMR (100.6 MHz, DMSO-*d*_6_): δ = 17.9, 20.0, 20.2, 26.0, 26.8, 28.5, 28.6, 32.1, 32.8, 40.1, 44.0, 53.4, 79.0, 127.2, 127.3, 128.5, 135.2, 142.2, 155.4. HR-MS (ESI): *m*/*z* calcd for C_19_H_28_NO_2_ [M -CH(CH_3_)_3_ + H + H]^+^: 302.21146; found: 302.21089.

**General method for dihydroxylation of 4a–c**

To a solution of **4a**–**c** (10 mmol) in acetone (50 mL), an aqueous solution of 4-methylmorpholine-4-oxide (NMO) (8.5 mL, 50% aqueous sol.) and a solution of OsO_4_ in *tert*-BuOH (4.5 mL, 2% *tert*-BuOH solution) were added in one portion. The mixture was stirred at room temperature overnight, and then quenched by the addition of a saturated aqueous solution of Na_2_SO_3_ (80 mL) and extracted with EtOAc (3 × 60 mL). The combined organic phase was dried, filtered, and evaporated. The products were purified by means of column chromatography on silica gel in *n*-hexane/EtOAc 4:1 mixture.

*tert*-Butyl benzyl(((1*S*,2*S*,4*S*)-1,2-dihydroxy-4-isopropylcyclohexyl)methyl)carbamate **5a**

Prepared from **4a** according to the general method. Yield: 0.95 g (25%) light yellow oil [α${]}_{20}^{D}$ = −15 (c 0.25, MeOH) ^1^H NMR (DMSO-*d*_6_, 400.1 MHz): δ = 0.85 (3H, d, *J* = 6.0 Hz), 0.86 (3H, d, *J* = 6.0 Hz), 1.09–1.20 (1H, m), 1.34–1.71 (6H, m), 1.44 (9H, s), 1.80–1.87 (1H, m), 3.31 (3H, dd, overlapped with br s, *J* = 15.5, 35.6 Hz), 3.59–3.65 (1H, m), 4.33 (1H, br s), 4.50 (2H, br s), 7.15–7.37 (5H, m); ^13^C NMR (100.6 MHz, DMSO-*d*_6_): δ = 20.4, 20.5, 25.2, 28.4, 29.8, 30.1, 30.4, 32.2, 37.5, 53.4, 53. 6, 70.0, 74.8, 81.5, 127.2, 127.5, 128.8, 138.0, 154.6. HR-MS (ESI): *m*/*z* calcd for C_22_H_36_NO_4_ [M + H]^+^: 378.26389; found: 378.26325, calcd for C_22_H_35_NO_4_Na [M + Na]^+^: 400.24583; found: 400.24510.

*tert*-Butyl benzyl(((1*R*,2*R*,4*S*)-1,2-dihydroxy-4-isopropylcyclohexyl)methyl)carbamate **6a**

Prepared from **4a** according to the general method. Yield: 1.72 g (46%) white crystals, m.p. 103–106 °C; [α${]}_{20}^{D}$ = −36 (c 0.25, MeOH); ^1^H NMR (CDCl_3_, 400.1 MHz): δ = 0.88 (6H, d, *J* = 6.8 Hz), 1.06–1.16 (1H, m), 1.24–1.55 (5H, m), 1.41 (9H, s), 1.70–1.79 (2H, m), 2.95 (1H, br s), 2.99 (1H, d, *J* = 15.1 Hz), 3.42 (1H, dt, *J* = 5.0, 11.5 Hz), 3.54 (1H, d, *J* = 14.7 Hz), 4.28 (2H, d, overlapped with br s, *J* = 15.1 Hz), 4.65 (1H, d, *J* = 15.1 Hz), 7.12–7.36 (5H, m); ^13^C NMR (100.6 MHz, CDCl_3_): δ = 20.2, 20.3, 24.3, 28.7, 32.6, 32.9, 34.2, 42.9, 53.8, 55.5, 71.8, 74.2, 81.6, 127.4, 127.7, 129.0, 138.3, 158.5. HR-MS (ESI): *m*/*z* calcd for C_22_H_36_NO_4_ [M + H]^+^: 378.26389; found: 378.26332, calcd for C_22_H_35_NO_4_Na [M + Na]^+^: 400.24583; found: 400.24518.

*tert*-Butyl (((1*S*,2*S*,4*S*)-1,2-dihydroxy-4-isopropylcyclohexyl)methyl)((*S*)-1-phenylethyl)carbamate **5b**

Prepared from **4b** according to the general method. Yield: 1.68 g (43%), oil, [α${]}_{20}^{D}$ = +2 (c 0.26, MeOH); ^1^H NMR (CDCl_3_, 400.1 MHz): δ = 0.88 (3H, d, *J* = 6.8 Hz), 0.87 (3H, d, *J* = 6.2 Hz), 0.91–1.01 (1H, m), 1.23–1.46 (5H, m), 1.32 (9H, s), 1.68 (3H, d, *J* = 7.1 Hz), 1.69–1.77 (2H, m), 3.19 (1H, d, *J* = 14.8 Hz), 3.38 (1H, dd, *J* = 4.6, 11.3), 3.57 (1H, d, *J* = 14.8 Hz), 4.73 (1H, dd, *J* = 6.9, 14.1 Hz), 7.19–7.35 (5H, m); ^13^C NMR (100.6 MHz, CDCl_3_): δ = 19.6, 20.2, 20.3, 24.4, 28.6, 32.9, 33.0, 34.2, 42.9, 57.7, 58.9, 72.4, 73.5, 81.7, 126.5, 127.4, 128.7, 142.7, 158.8. HR-MS (ESI): *m*/*z* calcd for C_23_H_38_NO_4_ [M + H]^+^: 392.27954; found: 392.27903, calcd for C_23_H_37_NO_4_Na [M + Na]^+^: 414.26148; found: 414.26077.

*tert*-Butyl (((1*R*,2*R*,4*S*)-1,2-dihydroxy-4-isopropylcyclohexyl)methyl)((*S*)-1-phenylethyl)carbamate **6b**

Prepared from **4b** according to the general method. Yield: 1.58 g (38%), oil, [α${]}_{20}^{D}$ = −5 (c 0.26, MeOH); ^1^H NMR (CDCl_3_, 400.1 MHz): δ = 0.79 (3H, d, *J* = 6.2 Hz), 1.02–1.14 (1H, m), 1.24–1.53 (5H, m), 1.30 (9H, s), 1.48–1.60 (2H, m), 1.65 (3H, d, *J* = 7.0 Hz), 1.74–1.82 (1H, m), 3.29 (1H, d, *J* = 14.8 Hz), 3.44 (1H, d, *J* = 14.8), 3.57–3.62 (1H, m), 4.81 (1H, dd, *J* = 6.9, 14.1 Hz), 7.21–7.36 (5H, m); ^13^C NMR (100.6 MHz, CDCl_3_): δ = 18.5, 20.8, 21.0, 25.0, 28.6, 30.1, 30.5, 32.6, 38.1, 54.9, 59.1, 70.8, 74.1, 81.8, 127.0, 127.5, 128.7, 142.4, 159.0. HR-MS (ESI): *m*/*z* calcd for C_23_H_38_NO_4_ [M + H]^+^: 392.27954; found: 392.27901, calcd for C_23_H_37_NO_4_Na [M + Na]^+^: 414.26148; found: 414.26077.

*tert*-Butyl (((1*S*,2*S*,4*S*)-1,2-dihydroxy-4-isopropylcyclohexyl)methyl)((*R*)-1-phenylethyl)carbamate (**5c**)

Prepared from **4c** according to the general method. Yield: 1.92 g (48%) oil, [α${]}_{20}^{D}$ = −18 (c 0.255, MeOH), ^1^H NMR (400.1 MHz, CDCl_3_): δ = 0.92 (6H, d, *J* = 6.8 Hz), 1.06–1.17 (1H, m), 1.34 (9H, s), 1.32–1.56 (7H, m), 1.72 (3H, d, *J* = 7.0 Hz), 1.73–1.81 (2H, m), 3.22 (1H, d, *J* = 14.6 Hz), 3.37–3.49 (2H, m), 3.60 (3H, d, *J* = 14.6 Hz), 4.77 (1H, q, *J* = 7.0, 13.8 Hz), 7.22–7.39 (5H, m). ^13^C NMR (100.6 MHz, CDCl_3_): δ = 19.8, 20.3, 20.5, 24.5, 28.8, 33.0, 33.1, 43.3, 43.1, 57.9, 59.1, 72.6, 73.7, 81.9, 126.7, 127.5, 128.9, 142.8, 158.9; HR-MS (ESI): *m*/*z* calcd for C_23_H_38_NO_4_ [M + H]^+^: 392.27954; found: 392.27901, calcd for C_23_H_37_NO_4_Na [M + Na]^+^: 414.26148; found: 414.26072.

*tert*-Butyl (((1*R*,2*R*,4*S*)-1,2-dihydroxy-4-isopropylcyclohexyl)methyl)((*R*)-1-phenylethyl)carbamate **6c**

Prepared from **4c** according to the general method. Yield: 1.88 g (48%), oil, [α${]}_{20}^{D}$ = −15 (c 0.275, MeOH), ^1^H NMR (DMSO-*d*_6_, 400.1 MHz): mixture of two rotamers, δ = 0.88 (3H, d, *J* = 6.8 Hz), 0.87 (3H, d, *J* = 6.2 Hz), 0.91–1.01 (1H, m), 1.23–1.46 (5H, m), 1.32 (9H, s), 1.68 (3H, d, *J* = 7.1 Hz), 1.69–1.77 (2H, m), 3.19 (1H, d, *J* = 14.8 Hz), 3.38 (1H, dd, *J* = 4.6, 11.3), 3.57 (1H, d, *J* = 14.8 Hz), 4.73 (1H, dd, *J* = 6.9, 14.1 Hz), 7.19–7.35 (5H, m); ^13^C NMR (100.6 MHz, DMSO-*d*_6_): δ = 20.2, 20,3, 20.7, 20.8, 24.0, 25.3, 28.3, 30.7, 32.5, 33.2, 33.7, 42.6, 54.6, 69.7, 72.6, 74.4, 79.0, 79.2, 126.4, 126.7, 128.2, 128.3, 144.3, 156.1. HR-MS (ESI): *m*/*z* calcd for C_23_H_38_NO_4_ [M + H]^+^: 392.27954; found: 392.27882, calcd for C_23_H_37_NO_4_Na [M + Na]^+^: 414.26148; found: 414.26057.

**General procedure for the Boc deprotection of 5a–c and 6a–c**

To a stirred solution of compounds **5a**–**c** or **6a**–**c** (3 mmol) in Et_2_O (25 mL), an 18% aqueous solution of HCl (70 mL) was added and the mixture was stirred overnight. After the reaction was complete (indicated by TLC), the solvent was removed by vacuum evaporation. The resulting hydrochloride salt was filtered off and washed with Et_2_O.

(1*S*,2*S*,4*S*)-1-((Benzylamino)methyl)-4-isopropylcyclohexane-1,2-diol hydrochloride **7a**

Prepared from **5a** according to the general method. Yield: 0.83 g (88%); white crystals, m.p. 215–218 °C; [α${]}_{20}^{D}$ = −5 (c 0.225, MeOH). ^1^H NMR (DMSO-*d*_6_, 400.1 MHz): δ = 0.84 (6H, d, *J* = 6.7 Hz, CHMe_2_), 1.01–1.12 (1H, m, H4_β_), 1.15–1.46 (5H, m, CH_2_(5), CHMe_2_, H4_α_, H6_β_), 1.48–1.56 (1H, m, 6H_α_), 1.67–1.78 (1H, m, 3H_α_), 2.73–2.83 (1H, m, CH_2_NH), 3.01–3.11 (1H, m, CH_2_NH), 3.37 (1H, dd, *J* = 4.4, 11.3 Hz, CHOH), 4.10–4.24 (2H, m, CH_2_Ph), 7.37–7.48 (3H, m, 3 × CH_Ar_), 7.55–7.66 (2H, m, 2 × CH_Ar_), 8.77 (1H, br s, NH_2_^+^), 9.15 (1H, br s, NH_2_^+^); ^13^C NMR (100.6 MHz, DMSO-*d*_6_): δ = 20.6 and 20.7 (CHMe_2_), 23.9 (C5), 32.8 (CHMe_2_), 33.9 (C3), 34.0 (C6), 42.5 (C4), 51.8 (CH_2_NH), 55.6 (CH_2_Ph), 71.1 (C1), 73.8 (C2), 129.5 (2 × CH_Ar_), 129.8 (2 × CH_Ar_), 131.1, 132.7 (C_qAr_). HR-MS (ESI): *m*/*z* calcd for C_17_H_28_NO_2_ [M + H]^+^: 278.21146; found: 278.21069.

(1*S*,2*S*,4*S*)-4-Isopropyl-1-((((*S*)-1-phenylethyl)amino)methyl)cyclohexane-1,2-diol hydrochloride **7b**

Prepared from **5b** according to the general method. Yield: 0.81 g (82%); white crystals, m.p. 205–207 °C; [α${]}_{20}^{D}$ = +12 (c 0.255, MeOH). ^1^H NMR (DMSO-*d*_6_, 400.1 MHz): δ = 0.82 (6H, d, *J* = 6.7 Hz), 0.97–1.54 (7H, m), 1.64 (3H, d, *J* = 6.6 Hz), 1.77 (1H, d, *J* = 12.3 Hz), 2.43–2.51 (1H, m), 2.99–3.09 (1H, m), 3.32 (1H, d, *J* = 9.4 Hz), 3.37 (1H, br s,), 4.33–4.44 (1H, m), 4.67 (1H, s), 5.11 (1H, br s), 7.36–7.47 (3H, m), 7.60–7.67 (2H, m), 9.03 (1H, br s), 9.12 (1H, br s); ^13^C NMR (100.6 MHz, DMSO-*d*_6_): δ = 20.4, 20.5, 20.6, 23.8, 32.8, 33.8, 33.9, 42.5, 54.8, 59.4, 71.3, 73.3, 128.8, 129.6, 129.7, 138.1. HR-MS (ESI): *m*/*z* calcd for C_18_H_30_NO_2_ [M + H]^+^: 292.22711; found: 292.22641.

(1*S*,2*S*,4*S*)-4-Isopropyl-1-((((*R*)-1-phenylethyl)amino)methyl)cyclohexane-1,2-diol **7c**

Prepared from **5c** according to the general method. Yield: 0.73 g (84%), viscous oil; [α${]}_{20}^{D}$ = −18 (c 0.26, MeOH). ^1^H NMR (DMSO-*d*_6_, 400.1 MHz): *δ* = 0.82 (6H, d, *J* = 6.7 Hz), 0.93–1.03 (1H, m), 1.30–1.54 (1H, m), 1.11 (1H, dt, *J* = 4.4, 12.9 Hz), 1.16–1.48 (7H, m), 1.26 (3H, d, *J* = 6.8 Hz), 2.29 (1H, d, *J* = 12.2 Hz), 2.48 (1H, d, *J* = 12.2 Hz), 3.30 (1H, dd, *J* = 4.3, 11.6 Hz), 3.67 (1H, q, *J* = 6.5, 12.9 Hz), 7.18–7.35 (5H, m); ^13^C NMR (100.6 MHz, DMSO-*d*_6_): δ = 20.2, 20.3, 23.9, 24.8, 32.5, 33.4, 34.3, 42.1, 57.4, 58.5, 71.4, 74.6, 126.9, 127.2, 128.8, 146.0. HR-MS (ESI): *m*/*z* calcd for C_18_H_30_NO_2_ [M + H]^+^: 292.22711; found: 292.22652.

(1*R*,2*R*,4*S*)-1-((Benzylamino)methyl)-4-isopropylcyclohexane-1,2-diol hydrochloride **11a**

Prepared from **6a** according to the general method. Yield: 0.91 g (97%); white crystals, m.p. 172–174 °C; [α${]}_{20}^{D}$ = −13 (c 0.265, MeOH). ^1^H NMR (DMSO-*d*_6_, 400.1 MHz): δ = 0.78 (3H, d, *J* = 9.4 Hz, CHMe_2_), 0.80 (3H, d, *J* = 9.4 Hz, CHMe_2_), 0.96–1.08 (1H, m, H5_α_), 1.18–1.28 (1H, m, H6_α_), 1.30–1.54 (4H m, H5_β_, CHMe_2_, CH_2_(3)), 1,56–1.67 (2H, m, H4, H6_β_), 2.85 (2H, br s, CH_2_NH), 3.53 (1H, d, *J* = 3.3 Hz, H2), 4.15 (2H, s, CH_2_Ph), 4.80 (1H, br s, NH_2_^+^), 4.89 (1H, s, NH_2_^+^), 7.37–7.46 (3H, m, 3 × CH_Ar_), 7.55–7.64 (2H, m, 2 × CH_Ar_); ^13^C NMR (100.6 MHz, DMSO-*d*_6_): δ = 21.1 (CHMe_2_), 24.9 (C5), 30.2 (CHMe_2_), 30.6 (C3), 33.3 (C6), 37.8 (C4), 51.5 (CH_2_NH), 52.3 (CH_2_Ph), 70.0 (C2), 71.6 (C1), 129.5 (2 × CH_Ar_), 129.8 (CH_Ar_), 131.2 (2 × CH_Ar_), 132.5 (C_qAr_). HR-MS (ESI): *m*/*z* calcd for C_17_H_28_NO_2_ [M + H]^+^: 278.21146; found: 278.21068.

(1*R*,2*R*,4*S*)-4-Isopropyl-1-((((*S*)-1-phenylethyl)amino)methyl)cyclohexane-1,2-diol hydrochloride **11b**

Prepared from **6b** according to the general method. Yield: 0.78 g (79%), white crystals. m.p. 172–174 °C, [α${]}_{20}^{D}$ = −15 (c 0.26, MeOH) ^1^H NMR (DMSO-*d*_6_, 400.1 MHz): δ = 0.83 (3H, d, *J* = 6.6 Hz), 1.00–1.53 (7H, m), 1.63 (3H, d, *J* = 6.8 Hz), 1.66–1.76 (1H, m), 2.72–2.89 (1H, m), 3.28–3.38 (1H, m), 4.30–4.43 (1H, m), 4.61 (1H, s), 5.09 (1H, br s), 7.35–7.48 (3H, m), 7.55–7.65 (2H, m), 8.69 (1H, br s), 9.27 (1H, br s); ^13^C NMR (100.6 MHz, DMSO-*d*_6_): 20.2, 20.5, 20.7, 32.8, 33.8, 42.5, 54.7, 59.2, 71.1, 73.7, 128.8, 129.7, 129.8, 138.1. HR-MS (ESI): *m*/*z* calcd for C_18_H_30_NO_2_ [M + H]^+^: 292.22711; found: 292.22634.

(1*R*,2*R*,4*S*)-4-Isopropyl-1-((((*R*)-1-phenylethyl)amino)methyl)cyclohexane-1,2-diol hydrochloride **11c**

Prepared from **6c** according to the general method**.** Yield: 0.77 g (78%); white crystals m.p. 198–200 °C; [α${]}_{20}^{D}$ = +6 (c 0.26, MeOH). ^1^H NMR (DMSO-*d*_6_, 400.1 MHz): δ = 0.76 (3H, d, *J* = 8.3 Hz), 0.78 (3H, d, *J* = 8.3 Hz), 0.98–1.14 (1H, m), 1.26–1.63 (6H, m), 1.65 (3H, d, *J* = 6.9 Hz), 2.51–2.60 (1H, m), 2.84–2.95 (1H, m), 3.37 (1H, br s), 3.45 (1H, br s), 4.30–4.41 (1H, m), 4.78 (1H, br s), 4.93 (1H, s), 7.35–7.47 (3H, m), 7.57–7.65 (2H, m), 8.67 (1H, br s), 9.35 (1H, br s); ^13^C NMR (100.6 MHz, DMSO-*d*_6_): δ = 20.2, 21.1, 24.9, 30.2, 30.6, 33.2, 37.7, 51.8, 59.0, 69.7, 71.6, 128.9, 129.7, 129.8, 138.0. HR-MS (ESI): *m*/*z* calcd for C_18_H_30_NO_2_ [M + H]^+^: 292.22711; found: 292.22647.

**General procedure for debenzylation of 7a and 11a**

To a stirred suspension of palladium-on-carbon (10% Pd/C, 0.10 g) in a mixture of *n*-hexane/EtOAc (24 mL) (1:2 mixture for **7a**, 1:1 mixture for **11a**), **7a** or **11a** (1.8 mmol) was added and the reaction mixture was stirred under a H_2_ atmosphere at room temperature and normal pressure. After the reaction was completed (monitored by TLC), the mixture was filtered through a short Celite pad, the solvent was concentrated, and the hydrochloride salts of compounds were formed.

(1*S*,2*S*,4*S*)-1-Aminomethyl-4-isopropylcyclohexane-1,2-diol hydrochloride **8**

Prepared from **7a** according to the general method. Yield: 0.14 g (35%); white crystals m.p. 136–137 °C; [α${]}_{20}^{D}$ = −4 (c 0.265, MeOH). ^1^H NMR (DMSO-*d*_6_, 400.1 MHz): δ = 0.81 (6H, d, *J* = 6.8 Hz, CHMe_2_), 0.98–1.44 (6H, m, H3_α_, H(4), CH_2_(5), CHMe_2_, H6_β_), 1.46–1.54 (1H, m, H6_α_), 1.60–1.69 (1H, m, H3_α_), 2.57–2.69 (1H, m, CH_2_NH), 2.91–3.02 (1H, m, CH_2_NH), 3.28–3.38 (1H, m, overlapped with H_2_O, CHOH), 4.46 (1H, br s, CHOH), 4.93 (1H, br s, C_q_OH), 7.78 (3H, br s, NH_3_^+^); ^13^C NMR (100.6 MHz, DMSO-*d*_6_): δ = 20.6 and 20.7 (CHMe_2_), 24.0 (C3), 32.8 (CHMe_2_), 33.5 (C5), 34.0 (C6), 42.6 (C4), 47.9 (CH_2_NH), 70.9 (C1), 73.6 (C2). HR-MS (ESI): *m*/*z* calcd for C_10_H_22_NO_2_ [M + H]^+^: 188.16451; found: 188.16423.

(1*R*,2*R*,4*S*)-1-Aminomethyl-4-isopropylcyclohexane-1,2-diol hydrochloride **12**

Prepared from **11a** according to the general method. Yield: 0.18 g (45%); white crystals m.p. 176–178 °C; [α${]}_{20}^{D}$ = −14 (c 0.265, MeOH). ^1^H NMR (DMSO-*d*_6_, 400.1 MHz,): δ = 0.82 (3H, d, *J* = 8.4 Hz, CHMe_2_), 0.84 (3H, d, *J* = 8.4 Hz, CHMe_2_), 1.04–1.18 (1H, m, CHMe_2_), 1.27–1.69 (7H, m, CH(4), CH_2_(3), CH_2_(5), CH_2_(6)), 2.80 (2H, br s, (CH_2_NH)), 3.54 (1H, br d, *J* = 3.7 Hz, CHOH), 4.69 (1H, br s, CHOH), 4.72 (1H, br s, C_q_OH), 7.83 (3H, br s, NH_3_^+^); ^13^C NMR (100.6 MHz, DMSO-*d*_6_): δ = 21.19 and 21.20 (CHMe_2_), 25.1 (C5), 30.2 (C3), 30.5 (CHMe_2_), 33.5 (C6), 37.8 (C4), 45.1 (CH_2_NH), 69.8 (C2), 71.4 (C1). HR-MS (ESI): *m*/*z* calcd for C_10_H_22_NO_2_ [M + H]^+^: 188.16451; found: 188.16419.

**General procedure used to form the *N*-methyl derivatives of compounds 9 and 13**

A solution of appropriate compound (**5a** or **6a**) (1.00 g, 2.65 mmol) in dry THF (12 mL) was added to a stirred suspension of LiAlH_4_ (0.30 g, 7.95 mmol) in dry THF (15 mL) carefully at 0 °C. The mixture was stirred at reflux for 6 h. When the TLC indicated, a mixture of H_2_O (1 mL) and THF (16 mL) was added dropwise with cooling. The precipitated material was filtered off and washed with THF. The filtrate was dried (Na_2_SO_4_), filtered, and concentrated in a vacuum. The crude products were purified via column chromatography on silica gel by applying DCM/MeOH 19:1 (for compound **9**) *n*-hexane/EtOAc 2:1 (for compound **13**).

(1*S*,2*S*,4*S*)-1-((Benzyl(methyl)amino)methyl)-4-isopropylcyclohexane-1,2-diol (**9**)

Prepared from **5a** according to the general method. Yield: 0.46 g (60%), colourless oil, [α${]}_{20}^{D}$ = −44 (c 0.25, MeOH). ^1^H NMR (DMSO-*d*_6_, 400.1 MHz): δ = 0.84 (6H, d, *J* = 6.6 Hz), 0.77–0.87 (1H, m), 0.81–0.86 (1H, m), 0.98–1.09 (1H, m), 1.21–1.34 (5H, m), 1.37–1.57 (3H, m), 2.20 (3H, s), 2.41 (1H, d, *J* = 13.5 Hz), 2.53 (1H, d, *J* = 13.5 Hz), 3.39 (1H, dd, *J* = 4.4, 11.4 Hz), 3.50 (1H, d, *J* = 13.3 Hz), 3.67 (1H, d, *J* = 13.3 Hz), 3.72 (1H, br s), 5.06 (1H, br s), 7.21–7.26 (1H, m), 7.28–7.35 (4H, m); ^13^C NMR (100.6 MHz, DMSO-*d*_6_): δ = 20.2, 20.3, 23.9, 28.7, 32.5, 33.5, 34.2, 42.4, 44.6, 64.0, 66.3, 72.9, 73.5, 127.3, 128.6, 129.1, 139.9. HR-MS (ESI): *m*/*z* calcd for C_18_H_30_NO_2_ [M + H]^+^: 292.22711; found: 292.22635.

(1*R*,2*R*,4*S*)-1-((Benzyl(methyl)amino)methyl)-4-isopropylcyclohexane-1,2-diol **13**

Prepared from **6a** according to the general method. Yield: 0.45 g (58%), colourless oil, [α${]}_{20}^{D}$ = −8 (c 0.25, MeOH). ^1^H NMR (DMSO-*d*_6_, 400.1 MHz): δ = 0.73 (3H, d, *J* = 9.0 Hz), 0.74 (3H, d, *J* = 9.0 Hz), 0.77–0.87 (1H, m), 0.99–1.07 (1H, m), 1.25–1.44 (4H, m), 1.49–1.56 (1H, m), 2.25 (3H, s), 2.37 (2H, dd, *J* = 1.0, 14.1 Hz), 3.48 (1H, d, *J* = 12.8 Hz), 3.56 (1H, d, *J* = 12.8 Hz), 3.59–3.63 (1H, m), 3.79 (1H, br s), 4.39 (1H, br s), 7.19–7.25 (1H, m), 7.27–7.33 (4H, m); ^13^C NMR (100.6 MHz, DMSO-*d*_6_): δ = 20.4, 20.5, 25.5, 30.9, 31.8, 33.0, 36.7, 45.1, 61.9, 64.0, 69.6, 73.9, 127.4, 128.5, 140.1. HR-MS (ESI): *m*/*z* calcd for C_18_H_30_NO_2_ [M + H]^+^: 292.22711; found: 292.22635.

**General method for the preparation of 1,3 oxazines 10a–c**

To a stirred solution of aminodiol (**7a**–**c**) (0.15 g) in Et_2_O (6 mL), a 35% aqueous solution of formaldehyde (4.5 mL) was added. The reaction mixture was stirred at room temperature for 3 h, then extracted with a 10% aqueous solution of KOH (10 mL). The aqueous layer was extracted with Et_2_O (3 × 25 mL), and then the combined organic phase was washed with brine (3 × 25 mL). The organic layer was dried (NaSO_4_), filtered, and evaporated. The crude products were purified by column chromatography on silica gel by using an *n*-hexane/EtOAc 9:1 mixture for compound **10a** and a 4:1 mixture for compounds **10b** and **10c**.

(4a*S*,7*S*,8a*S*)-3-Benzyl-7-isopropyloctahydro-2*H*-benzo[*e*][1,3]oxazin-4a-ol **10a**

Prepared from **7a** according to the general method. Yield: 0.11 g (70%); white crystals; m.p. 155–157 °C; [α${]}_{20}^{D}$ = +38 (c 0.25, MeOH). ^1^H NMR (DMSO-*d*_6_, 400.1 MHz,): δ = 0.84 (3H, d, *J* = 6.8 Hz, CHMe_2_), 0.85 (3H, d, *J* = 6.8 Hz, CHMe_2_), 1.04–1.21 (2H, m, CH_2_(6)), 1.27–1.48 (6H, m, CH_2_(8), CHMe_2_, H7, CH_2_(5)), 2.18 (1H, d, *J* = 11.3 Hz, H4_α_), 2.63 (1H, d, *J* = 11.5 Hz, H4_β_), 3.14 (1H, dd, *J* = 4.8, 11.9 Hz, H8a), 3.58 (1H, d, *J* = 13.6 Hz, CH_2_Ph), 3.69 (1H, s, OH), 3.70 (1H, d, *J* = 13.8 Hz, CH_2_Ph), 3.80 (1H, d, *J* = 8.1 Hz, H2_α_), 4.36 (1H, dd, *J* = 1.2, 8.2 Hz, H2_β_), 7.20–7.27 (1H, m, CH_Ar_), 7.29–7.37 (4H, m, 4 × CH_Ar_); ^13^C NMR (100.6 MHz, DMSO-*d*_6_): δ = 20.5 and 20.7 (CHMe_2_), 24.1 (C6), 30.0 (C8), 33.0 (CHMe_2_), 33.5 (C5), 42.8 (C7), 57.3 (C4), 62.3 (CH_2_Ph), 67.2 (C4a), 82.1 (C8a), 85.5 (C2), 127.8 (CH_Ar_), 129.0 (2 × CH_Ar_), 129.5 (2 × CH_Ar_), 139.0 (C_qAr_). HR-MS (ESI): *m*/*z* calcd for C_18_H_28_NO_2_ [M + H]^+^: 290.21146 found: 290.21090.

(4a*S*,7*S*,8a*S*)-7-Isopropyl-3-((*S*)-1-phenylethyl)octahydro-2*H*-benzo[*e*][1,3]oxazin-4a-ol **10b**

Prepared from **7b** according to the general method. Yield: 0.15 g (96%), [α${]}_{20}^{D}$ = −19 (c 0.05, MeOH), ^1^H NMR (DMSO-*d*_6_, 400.1 MHz): δ = 0.83 (6H, dd, *J* = 1.2, 6.9 Hz), 1.00–1.18 (2H, m), 1.24 (1H, s), 1.27–1.35 (5H, m), 1.36–1.47 (3H, m), 2.00 (1H, d, *J* = 11.32 Hz), 2.68 (1H, dd, *J* = 1.6, 11.3 Hz), 3.04 (1H, dd, *J* = 4.7, 11.0 Hz), 3.57 (1H, d, *J* = 1.35 Hz), 3.70 (1H, d, *J* = 8.1 Hz), 3.81 (1H, q, *J* = 6.8, 13.5 Hz,), 7.19–7.26 (1H, m), 7.26–7.35 (4H, m); ^13^C NMR (100.6 MHz, DMSO-*d*_6_): δ = 20.4, 20.5, 20.7, 24.1, 30.0,32.9, 33.3, 42.8, 59.1, 60.0, 67.1, 82.1, 84.2, 86.4, 127.8, 128.2, 129.1, 143.8 HR-MS (ESI): *m*/*z* calcd for C_19_H_30_NO_2_ [M + H]^+^: 304.22711; found: 304.22631.

(4a*S*,7*S*,8a*S*)-7-Isopropyl-3-((*R*)-1-phenylethyl)octahydro-2*H*-benzo[*e*][1,3]oxazin-4a-ol **10c**

Prepared from **7c** according to the general method. Yield: 0.13 g (83%) [α${]}_{20}^{D}$ = −28 (c 0.07, MeOH) ^1^H NMR (CDCl_3_, 400.1 MHz): δ = 0.88 (6H, dd, *J* = 2.4, 6.8 Hz), 1.09–1.18 (2H, m), 1.26 (1H, s), 1.36 (3H, d, *J* = 6.9 Hz), 1.42–152 (4H, m), 1.57 (dt, *J* = 3.7, 11.9 Hz), 1.65 (dt, *J* = 3.3, 13.5 Hz), 2.07 (1H, d, *J* = 10.6 Hz), 2.86 (1H, dd, *J* = 2.2, 10.6 Hz), 3.08 (1H, dd, *J* = 4.4, 11.4 Hz), 3.54 (1H, dd, *J* = 6.8, 13.5 Hz), 3.66 (1H, d, *J* = 7.8 Hz), 4.45 (1H, dd, *J* = 2.1, 7.8 Hz), 7.22–7.26 (1H, m), 7.27–7.35 (4H, m); ^13^C NMR (100.6 MHz, CDCl_3_): δ = 19.1, 19.7, 20.0, 23.8, 29.5, 32.5, 32.7, 59.6, 59.8, 66.7, 82.0, 84.4, 127.2, 127.3, 128.4, 142.8. HR-MS (ESI): *m*/*z* calcd for C_19_H_30_NO_2_ [M + H]^+^: 304.22711; found: 304.22646.

(*S*)-(4)-Isopropylcyclohex-1-ene-1-ylmethanol **15**

To a solution of **2** (5.36 g, 35.2 mmol) in MeOH (110 mL), NaBH_4_ (3.99 g, 105.6 mmol) was added in small portions at 0 °C. The reaction mixture was stirred at room temperature for 1 h. When the reaction was completed (indicated by TLC), the solvent was evaporated. The residue was redissolved in water (100 mL) and extracted with DCM (3 × 100 mL). The combined organic phase was dried (Na_2_SO_4_), filtered, and concentrated to dryness. The crude product was used for the next step without further purification. Isolated product: 5.16 g (95%), light green transparent oil. All its spectroscopic data and physical properties agreed with those reported in the literature [30].

2,2,2-Trichloro-*N*-((1*R*,5*S*)-5-isopropyl-2-metilenecyclohexyl)acetamide (intermediate) **16a** and 2,2,2-trichloro-*N*-((1*S*,5*S*)-5-isopropyl-2-methylenecyclohexyl)acetamide **16b**

To a solution of **15** (10.48 g, 67.9 mmol) in dry DCM (320 mL), 1,8-diazabicyclo [5.4.0]undec-7-ene (12.41 g, 81.5 mmol, 12.2 mL) was added applying an Ar atmosphere. Trichloroacetonitrile (10.3 mL, 97.4 mmol) was added in small portions to the reaction mixture, and it was stirred for 2 h at room temperature. Upon completion of the reaction (indicated by TLC), the solvent was evaporated. The crude product was purified via chromatography on silica gel by using DCM. The top one-third of the column was dry Na_2_SO_4,_ and the bottom two-thirds of the column were silica gel. The fractions that contained the imidate intermediate were collected, and the solvent was evaporated. The slightly yellow oil product was dissolved in dry toluene (300 mL) and reacted in an autoclave at 130 °C in an Ar atmosphere for 24 h. When the reaction was completed, the mixture was extracted with a cold aqueous solution of HCl (5%, 3 × 100 mL), and the organic layer was dried, filtered, and evaporated. Based on ^1^H NMR measurements and GC determination (Chirasil-DEX CB column, 2 mL flow rate, 110 °C, Figure S145), the diastereomers formed a mixture with a ratio of 85:15. Although various column chromatography methods were carried out, the diastereomers were inseparable.

Yield: 14.52 g (72%) (two diastereomers); orange oil; [α${]}_{20}^{D}$ = −33 (c 0.505, MeOH); ^1^H NMR (CDCl_3_, 500.2 MHz): δ = 0.87 (3H, d, *J* = 1.6 Hz, minor), 0.88 (3H, d, *J* = 1.6 Hz, minor), 0.90 (3H, d, *J* = 6.7 Hz, major), 0.92 (3H, d, *J* = 6.8 Hz, major), 0.97–1.08 (1H, m), 1.23–1.41 (2H, m), 1.49–1.63 (4H, m), 1.73–1.86 (2H, m), 1.90–1.98 (1H, m), 2.05–2.21 (2H, m), 2.35 (1H, dt, *J* = 4.3; 14.4 Hz, major), 2.50 (1H, dt, *J* = 3.3; 13.8 Hz, minor), 4.34–4.43 (1H, m, minor), 4.49–4.57 (1H, m, major), 4.71 (1H, s, minor), 4.81 (1H, s, minor), 4.88 (1H, s, major), 4.97 (1H, s, major), 6.66 (1H, br s, minor), 6.79 (1H, br s, major); ^13^C NMR (125.8 MHz, CDCl_3_): δ = 19.7, 19.8, 19.9, 20.0, 28.8, 29.4, 30.0, 30.7, 31.0, 32.2, 34.9, 37.6, 38.8, 43.1, 52.8, 53.5, 71.7, 74.3, 104.9, 111.4, 145.1, 147.4, 160.7, 161.0; HR-MS (ESI): *m*/*z* calcd for C_12_H_19_Cl_3_NO [M + H]^+^: 298.05322; found: 298.05359.

*tert*-Butyl ((1*R*,5*S*)-5-isopropyl-2-methylenecyclohexyl)carbamate **18a** and *tert*-butyl ((1*S*,5*S*)-5-isopropyl-2-methylenecyclohexyl)carbamate **18b**

To a solution of **16a** and **16b** (11.11 g, 37.2 mmol) in EtOH/DCM 2:1 (66 mL), an aqueous solution of NaOH (5M solution, 525.6 mL) was added. The reaction was stirred at 50 °C for 15 h. As the TLC indicated, the reaction was cooled to room temperature, then extracted with DCM (3 × 150 mL). The combined organic phase was washed with brine (100 mL), dried, filtered, and evaporated. The residue was dissolved in dry THF (100 mL), and then triethylamine (11.29 g, 111.6 mmol), DMAP (0.45 g, 3.72 mmol), and di-*tert*-butyl dicarbonate (8.93 g, 40.9 mmol) were added to the reaction mixture. The mixture was stirred at room temperature for 12 h. After that, the solvent was evaporated, and the crude product was purified via column chromatography on silica gel by applying *n*-hexane/Et_2_O 9:1. The diastereomers were still inseparable in this step. The ratio of the formed diastereomers remained 85:15 based on the determination by GC (Chirasil-DEX CB column, 2 mL flow rate, 160 °C, Figure S146).

Yield (for the mixture of isomers): 5.56 g (59%); colourless viscous oil; [α${]}_{20}^{D}$ = −48 (c 0.250, MeOH); ^1^H NMR (DMSO-*d*_6_, 500.2 MHz) of **18a** (major): δ = 0.79 and 0.78 (6H, overlapping d’s, *J* = 6.7 Hz, CHMe_2_), 1.06 (1H, qa, *J* = 12.3 Hz, 4H_β_), 1.25 (1H, m, 6H_β_), 1.32 (9H, s, CMe_3_), 1.41 (1H, oct, *J* = 6.7 Hz, CHMe_2_), 1.48 (1H, m, 5H_α_), 1.56 (1H, m, 4Hα), 4.67 (1H, brs, H_A_); 1.61 (1H, br d, *J* = 12.2 Hz, 6H_α_), 2.13 (1H, td, *J* = 4.3, 13.9 Hz, 3H_β_), 2.22 (m, 1H, 3H_α_), 4.60 (1H, br s, H_B_); 4.03 (1H, br s, 1H_β_), 6.85 (1H, br s, NH). ^13^C-NMR (DMSO-*d*_6_, 125 MHz): δ = 20.3 and 20.4 (CHMe_2_), 28.7 (CMe_3_), 30.4 (4C), 30.5 (3C), 30.9 (CHMe_2_), 36.2 (6C), 37.7 (4C), 52.2 (1C), 78.0 (CMe_3_), 108.9 (=CH_2_), 149.0 (C2),155.3 (C=O).

**18b**/*minor*: ^1^H-NMR (DMSO-*d*_6_) separated diagnostic signals: δ = 0.94 (1H, qa, *J* = 10.6 Hz, 6H_β_), 1.92 (1H, ddd, *J* = 13.5 Hz, 3.9, 9.5 Hz, 3H_α_), 2.32 (dt, *J* = 13.5 Hz and 4.2 Hz, 1H, 3H_β_), 3.78 (1H, br t, *J* = 8.5 Hz, 1H_α_). ^13^C-NMR (DMSO-*d*_6_, 125 MHz) diagnostic signals separated in the 1D spectrum or identified on the basis of 2D-HSQC: δ = 34.3 (3C), 37.7 (6C, coalesced with 4C line of **18a**/*major*), 53.1 (1C), 104.9 (=CH_2_), 149.7 (C2), 155.5 (C=O). HR-MS (ESI): *m*/*z* calcd for C_11_H_20_NO_2_ [M − CH(CH_3_)_3_ + H + H]^+^: 198.14886; found: 198.14866.

**Dihydroxylation of compound mixture of 18a and 18b**

To a solution of **18a** and **18b** (5.40 g, 21.3 mmol) in acetone (100 mL), 4-methylmorpholine-4-oxide (22.5 mL, 96 mmol, 50% aq. solution) and OsO_4_ (1.5 mL, 2.0% *tert*-BuOH solution) were added. The reaction mixture was stirred for 72 h at room temperature, then was quenched with a saturated aqueous solution of Na_2_SO_3_ (20 mL), and extracted with ethyl acetate (3 × 100 mL). The combined organic phase was dried, filtered, and concentrated to dryness. Based on the ^1^H NMR spectra, **19a**, **19b**, and **19c** were formed in the ratio of 71:16:13. The crude product was purified by column chromatography on silica gel by using *n*-hexane/EtOAc 2:1). We found that **19a** was successfully isolated, while **19a** and **19b** remained as a mixture. Total yield: 4.96 g (81%).

*tert*-Butyl ((1*R*,2*S*,5*S*)-2-hydroxy-2-hydroxymethyl-5-isopropylcyclohexyl)-carbamate **19a**

Yield: 2.94 g (48%); white crystals; m.p.: 111–112 °C; [α${]}_{20}^{D}$ = −41 (c 0.275, MeOH); ^1^H NMR (CDCl_3_, 500.2 MHz): δ = 0.89 (3H, d, *J* = 6.7 Hz), 0.90 (3H, d, *J* = 6.7 Hz), 0.97–1.08 (1H, m), 1.19–1.28 (1H, m), 1.39–1.60 (5H, m), 1.46 (9H, s), 1.88 (1H, dt, *J* = 4.1; 13.2 Hz), 3.08 (1H, d, *J* = 12.8 Hz), 3.47 (1H, d, *J* = 12.1 Hz), 3.60–3.66 (1H, m), 4.82 (1H, d, *J* = 8.1 Hz); ^13^C NMR (125.8 MHz, CDCl_3_): δ = 19.6, 20.0, 23.2, 23.4, 28.3, 28.5, 29.7, 32.5, 38.5, 50.1, 67.2, 72.5, 80.7, 157.4; HR-MS (ESI): *m*/*z* calcd for C_17_H_26_N [M + H]^+^: 436.2410; found: 258.22099.

(1*R*,2*R*,5*S*)- and (1*S*,2*R*,5*S*)-*tert*-Butyl 2-hydroxy-2-hydroxymethyl-5-isopropylcyclohexyl)carbamate mixture **19a** and **19b**

Compounds **19b** and **19c** (2.02 g (33%); white crystals; were used in acetal preparation without further separation. HR-MS (ESI): *m*/*z* calcd for C_15_H_30_NO_4_ [M + H]^+^: 288.21693; found: 288.21649, calcd for C_15_H_29_NO_4_Na [M + Na]^+^: 310.19888; found: 310.19816.

**Acetal synthesis starting from 19b and 19c**

To a solution of Boc-protected aminodiol mixture **19a** and **19b** (1.20 g, 4.2 mmol) in dry acetone (100 mL), 4-methylbenzene-1-sulfonic acid (0.10 g 0.6 mmol) was added and stirred at room temperature. When the TLC indicated, the solvent was concentrated in a vacuum. The crude product was purified by column chromatography on silica gel, applying *n*-hexane/EtOAc 1:1 and then *n*-hexane/EtOAc 9:1 to yield compounds **20b** and **20c**.

*tert*-Butyl ((5*R*,6*R*,8*S*)-8-isopropyl-2,2-dimethyl-1,3-dioxaspiro [4,5]decane-6-yl)carbamate **20b**

Yield: 0.77 g (56%); white crystals; m.p.: 75–78 °C; [α${]}_{20}^{D}$ = −9 (c 0.490, MeOH); ^1^H NMR (CDCl_3_, 500.2 MHz): δ = 0.87 (3H, d, *J* = 6.8 Hz), 0.90 (3H, d, *J* = 6.8 Hz), 1.11–1.31 (2H, m), 1.18–1.29 (1H, m), 1.34–1.43 (1H, m), 1.39 (3H, s), 1.41 (3H, s), 1.46 (9H, s), 1.49–1.76 (5H, m, 1.83–1.93 (1H, m), 3.66–3.73 (1H, m), 3.73 (1H, d, *J* = 8.4 Hz), 3.93 (1H, d, *J* = 8.8 Hz), 4.72 (1H, br d, *J* = 5.8 Hz); ^13^C NMR (125.8 MHz, DMSO-*d*_6_): δ = 20.4, 20.6, 25.5, 27.0, 27.2, 28.4, 29.5, 32.1, 32.6, 38.0, 51.2 72.2, 82.0, 109.3, 155.9; HR-MS (ESI): *m*/*z* calcd for C_18_H_34_NO_4_ [M + H]^+^: 328.24823; found: 328.24784.

*tert*-Butyl ((5*R*,6*S*,8*S*)-8-isopropyl-2,2-dimethyl-1,3-dioxaspiro [4,5]decane-6-yl)carbamate **20c**

Yield: 0.24 g (18%); white crystals; m.p.: 167–168 °C; [α${]}_{20}^{D}$ = +11 (c 0.255, MeOH); ^1^H NMR (DMSO-*d*_6_, 500.2 MHz), δ = 0.79–0.94 (2H, m), 0.81 (3H, d, *J* = 5.8 Hz), 0.83 (3H, d, *J* = 5.8 Hz), 1.09–1.19 (1H, m), 1.23 (3H, s), 1.26 (3H, s), 1.32–1.41 (1H, m), 1.37 (9H, s), 1.46 (1H, dt, *J* = 2.5, 12.9 Hz), 1.57–1.66 (2H, m), 1.75–1.80 (1H, m), 3.43–3.51 (1H, m), 3.62 (1H, d, *J* = 9.0 Hz), 4.07 (1H, d, *J* = 9.0 Hz), 6.56 (1H, br d, *J* = 9.5 Hz); ^13^C NMR (125.8 MHz, DMSO-*d*_6_): δ = 20.2, 20.4, 26.7, 27.3, 27.7, 28.8, 32.2, 35.2, 37.8, 42.8, 54.2, 67.3, 77.7, 84.0, 108.3, 155.5; HR-MS (ESI): *m*/*z* calcd for C_18_H_34_NO_4_ [M + H]^+^: 328.24823; found: 328.24781.

**General procedure for the Boc and acetonide deprotection of compounds 19a, 20b, and 20c**

To a stirred solution of compounds **19a**, **20b**, and **20c** (0.70 mmol) in Et_2_O (6 mL), an aqueous solution of HCl (10%, 10 mL) was added. The reaction mixture was stirred vigorously for 24 h at room temperature. When the reaction was completed (indicated by TLC), it was extracted with Et_2_O (2 × 10 mL). The compounds were further purified as hydrochloride salts.

(1*S*,2*R*,4*S*)-2-Amino-1-hydroxymethyl-4-isopropylcyclohexanol hydrochloride **21a**

Prepared from **19a** according to the general method. Yield: 0.133 g (86%); white crystals, m.p.: 247–250 °C; [α${]}_{20}^{D}$ = −16 (c 0.700, MeOH); ^1^H-NMR (DMSO-*d*_6_): δ = 0.78 and 0.79 (6H, overlapping d’s, *J* = 6.7 Hz, CHMe_2_), 1.27–1.30 (4H, m, 3H_α_, 4H_β_ and 5H_α_ and 6H_β_), 1.32–1.36 (2H, m, CHMe_2_ and 4H_α_), 1.55–1.67 (2H, m, 3H_β_ and 6H_α_), 3.12 (m 1H, 1H_β_), 3.23 and 3.34 (2H, 2xd, *J* = 11.9 Hz, OCH_2_), 7.96 (3H, s, NH_3_^+^). The signals of the rapidly exchanging OH protons are merged in the broadened HDO signal of the solvent centred at c.a. 3.3 ppm. ^13^C-NMR (DMSO-*d*_6_): δ = 20.0 and 20.1 (CHMe_2_), 23.3 (C4), 28.5 (two coalesced lines (C3 and C6), 31.9 (CHMe_2_), 36.9 (C5), 52.0 (C1), 67.0 (OCH_2_), 70.5 (C2); HR-MS (ESI): *m*/*z* calcd for C_10_H_22_NO_2_ [M + H]^+^: 188.16451; found: 188.16451.

(1*R*,2*R*,4*S*)-2-Amino-1-hydroxymethyl-4-isopropylcyclohexanol hydrochloride **21b**

Prepared from **20b** according to the general method. Yield**:** 0.131 g (86%); white crystals m.p.: 142–143 °C; [α${]}_{20}^{D}$ = −7 (c 0.260, MeOH); ^1^H-NMR (DMSO-*d*_6_): δ = 0.78 and 0.80 (overlapping d’s, *J* = 6.7 Hz, 6H, CHMe_2_), 1.17 (m, H4_β_), 1.29 (m, 1H, 5H_α_), 1.38 (m, 1H, 3H_α_), 1.44 (oct, *J* = 6.7 Hz, 1H, CHMe_2_), 1.52–1.55 (m, 3H, 3H_β_, 4H_α_ and 6H_β_), 1.72 (br d, *J* = 11.3 Hz, 1H, 6H_α_), 3.19 (m 1H, 1H_β_), 3.29 and 3.36 (2 × d, *J* = 11.9 Hz, 2 × 1H, OCH_2_), 7.73 (s, 3H, NH_3_^+^). The signals of the rapidly exchanging OH protons are merged in the broadened HDO signal of the solvent centred at c.a. 3.3 ppm. ^13^C-NMR (DMSO-*d*_6_): δ = 20.78 and 20.80 (CHMe_2_), 24.0 (C4), 28.7 (C6), 28.8 (CHMe_2_), 29.4 (C3), 37.5 (C5), 50.1 (C1), 65.8 (OCH_2_), 71.0 (C2); HR-MS (ESI): *m*/*z* calcd for C_10_H_22_NO_2_ [M + H]^+^: 188.16451; found:188.16434.

(1*R*,2*S*,4*S*)-2-Amino-1-hydroxymethyl-4-isopropylcyclohexanol hydrochloride **21c**

Prepared from **20c** according to the general method. Yield**:** 0.124 g (80%); white crystals m.p.: 247–250 °C; [α${]}_{20}^{D}$ = +8 (c 0.320, MeOH); ^1^H-NMR (DMSO-*d*_6_): δ = 0.79 (6H, d, *J* = 6.7 Hz, CHMe_2_), 0.99 (1H, qad, *J* = 12.8 Hz and 3.6 Hz, H4_β_), 1.22 (1H, m, 5H_α_); 1.19 (1H, m, 3H_α_), 1.26 (1H, qa, *J* = 11.9 Hz; 6H_β_), 1.41 (1H, oct, *J* = 6.7 Hz, CHMe_2_), 1.46 (1H, br d, *J* = 13.0 Hz, 4H_α_), 1.79–1.83 (2H, m, 3H_β_ and 6H_α_), 2.93 (1H, m 1H_α_), 3.39 and 3.60 (2H, 2xd, *J* = 11.7 Hz, OCH_2_), 4.96 (1H, br s, CH_2_OH), 5.00 (1H, s, OH), 7.85 (3H, s, NH_3_^+^); ^13^C-NMR (DMSO-*d*_6_): δ = 20.6 and 20.8 (CHMe_2_), 26.0 (C4), 31.0 (C6), 32.1 (CHMe_2_), 35.3 (C3), 42.4 (C5), 58.6 (C1), 62.6 (OCH_2_), 71.8 (C2); HR-MS (ESI): *m*/*z* calcd for C_10_H_22_NO_2_ [M + H]^+^: 188.16451; found: 188.16430.

**General procedure for preparation of *N*-benzyl derivatives 23a and 23b**

To a solution of primer aminodiol base form **21a** or **21b** (0.19 g, 1.0 mmol) in dry EtOH (20 mL), 0.11 g (1.05 mmol) benzaldehyde was added, and the reaction mixture was stirred at room temperature. When the reaction was completed (monitored by means of TLC, 1 h), the solvent was concentrated and the residue was dissolved in dry EtOH (20 mL) and stirred for 1 h, and then NaBH_4_ (0.11 g, 3.0 mmol) was added in small portions. When the reaction was completed (indicated by TLC), the solvent was concentrated to dryness; then, H_2_O (15 mL) was poured into the residue and extracted with DCM (3 × 25 mL). After drying (Na_2_SO_4_), filtration, and solvent evaporation, the crude product was purified via column chromatography by applying a CHCl_3_/MeOH 9:1 mixture.

(1*S*,2*R*,4*S*)-2-Benzylamino-1-hydroxymethyl-4-isopropylcyclohexanol **23a**

Prepared from **21a** according to the general method. Yield: 0.150 g (54%); light yellow oil; [α${]}_{20}^{D}$ = −44 (c 0.405, MeOH); ^1^H NMR (DMSO-*d*_6_, 500.2 MHz): δ = 0.78 (3H, d, *J* = 6.8 Hz, CHMe_2_, 0.79 (3H, d, *J* = 6.8 Hz, CHMe_2_, 0.79–0.86 (1H, m, H5_α_), 1.02–1.13 (1H, m, CHMe_2_, 1.34–1.52 (5H, m H3_α_, H4, H5_β_, CH_2_(6)) 1.56–1.63 (1H, m H3_β_), 2.02 (1H, br s, OH), 2.60–2.66 (1H, m, H2), 3.30 (1H, d, *J* = 11.1 Hz, overlap with H_2_O sign, CH_2_OH), 3.39 (1H, d, *J* = 11.2 Hz, CH_2_OH, 3.60 (1H, d, *J* = 13.4 Hz, CH_2_Ph), 3.76 (1H, d, *J* = 13.2 Hz, CH_2_Ph), 4.15 (1H, s CH_2_OH, 4.58 (1H, br s, NH), 7.21–7.24 (1H, m, CH_Ar_), 7.29–7.34 (4H, m, 4 × CH_Ar_); ^13^C NMR (125.8 MHz, DMSO-*d*_6_): δ = 20.6 (CHMe_2_), 20.7 (CHMe_2_), 25.4 (C5), 29.0 (C3), 30.3 (CHMe_2_), 30.4 (C6), 37.0 (C4), 51.6 (CH_2_Ph), 56.6 (C2), 66.5 (CH_2_OH), 73.0 (C1), 127.0 (CH_Ar_), 128.4 (2 × CH_Ar_), 128.6 (2 × CH_Ar_), 141.9 (C_qAr_); HR-MS (ESI): *m*/*z* calcd for C_17_H_28_NO_2_ [M + H]^+^: 278.21146; found: 278.21096.

(1*R*,2*R*,4*S*)-2-Benzylamino-1-hydroxymethyl-4-isopropylcyclohexanol **23b**

Prepared from **21b** according to the general method. Yield: 0.246 g (89%); light yellow oil; [α${]}_{20}^{D}$ = −76 (c 0.245, MeOH); ^1^H NMR (DMSO-*d*_6_, 500.2 MHz): δ = 0.83 (6H, d, *J* = 5.6 Hz, 2 × CH_2_Me_2_) 1.22–1.42 (6H, m, H5_α_, CH_2_(3), H4, CH_2_(5)), 1.48–1.55 (1H, m, H5_β_), 1.59–1.67 (1H, m, H6_α_), 2.55–2.58 (1H, m, H6_β_), 3.29 (2H, dd, *J* = 10.8, 14.9 Hz CH_2_OH), 3.61 (1H, d, *J* = 13.8 Hz, CH_2_Ph), 3.77 (1H, d, *J* = 13.8 Hz, CH_2_Ph), 3.90 (1H, s, OH), 7.20–7.23 (1H, m, CH_Ar_), 729–7.33 (4H, m, 4 × CH_Ar_); ^13^C NMR (125.8 MHz, DMSO-*d*_6_): δ = 20.2 (CH_2_Me_2_), 20.3 (CH_2_Me_2_), 23.9 (C5), 27.4 (C3), 29.6 (CHMe_2_), 32.5, (C6), 36.4 (C4), 51.4 (CH_2_Ph), 59.2 (C2), 70.0 (CH_2_OH), 71.7 (C1), 127.0 (CH_Ar_), 128.5 (2 × CH_Ar_), 128.6 (2 × CH_Ar_), 141.6 (C_qAr_); HR-MS (ESI): *m*/*z* calcd for C_17_H_28_NO_2_ [M + H]^+^: 278.21146; found: 278.21073.

(1*S*,2*R*,4*S*)-1-Hydroxymethyl-4-isopropyl-2-(methylamino)cyclohexanol-hydrocloride **22**

To a suspension of LiAlH_4_ (0.18 g, 4.7 mmol) in dry THF (5 mL), a solution of compound **19a** (0.45 g, 1.57 mmol) in dry THF (5 mL) was added dropwise and stirred for 2 h, and then the excess of LiAlH_4_ was quenched with a mixture of H_2_O (0.36 mL) and THF (5 mL) at 0 °C. The suspension was stirred for 1 h at room temperature and then filtered. The inorganic residue was washed with THF (3 × 30 mL), and then the organic layer was dried, filtered, and concentrated to dryness. As its hydrochloride salt, the crude product was crystallised using a solution of HCl (10%, in EtOH/Et_2_O).

Yield: 0.243 g (65%); white crystals m.p.: 155–157 °C; [α${]}_{20}^{D}$ = −35 (c 0.265, MeOH); ^1^H NMR (DMSO-*d*_6_, 500.2 MHz): δ = 0.85 (3H, d, *J* = 7.1 Hz), 0.86 (3H, d, *J* = 7.1 Hz), 1.25–1.51 (5H, m), 1.63–1.77 (3H, m), 2.54 (3H, s), 3.07 (1H, br s), 3.38 (1H, d, *J* = 12.1 Hz), 3.49 (1H, d, *J* = 11.9 Hz), 4.89 (1H, s), 5.28 (1H, br s), 8.51 (2H, br s,); ^13^C NMR (125.8 MHz, DMSO-*d*_6_): δ = 20.1, 20.2, 23.3, 25.4, 29.3, 31.2, 32.6, 36.5, 60.8, 66.5, 70.8; HR-MS (ESI): *m*/*z* calcd for C_11_H_24_NO_2_ [M + H]^+^: 202.18016; found: 202.17983.

**General method for ring closure of compounds 22, 23a, and 23b with formaldehyde**

To a solution of aminodiol **22**, **23a**, and **23b** (0.58 mmol) in Et_2_O (10 mL), an aqueous solution of formaldehyde (40%, 5 mL) was added, and the mixture was stirred at room temperature for 1 h. An aqueous solution of NaOH (5%) was added to the reaction mixture to make it alkaline and extracted with Et_2_O (3 × 20 mL). The combined organic phase was dried (Na_2_SO_4_), filtered, and evaporated in vacuo. The crude products were purified by column chromatography (toluene/EtOH 4:1).

(4a*S*,7*S*,8a*R*)-7-Isopropyl-1-methyloctahyro-1*H*-benzo[d][1,3]oxazine-4a-ol **24**

Prepared from **22** according to the general method. Yield: 0.081 g (66%); brown oil; [α${]}_{20}^{D}$ = −38 (c 0.280, MeOH); ^1^H NMR (DMSO-d_6_, 500.2 MHz): δ = 0.83 (6H, d, *J* = 6.5 Hz), 1.21–1.27 (1H, m), 1.30–1.41 (4H, m), 1.44–1.52 (1H, m), 1.62–1.68 (1H, m), 1.87–1.91 (1H, m), 1.97 (3H, s), 3.10 (1H, d, *J* = 10.5 Hz,), 3.40 (1H, d, *J* = 11.3 Hz), 3.41 (1H, d, *J* = 7.3 Hz), 4.27 (1H, d, *J* = 7.4 Hz), 4.41 (1H, s); ^13^C NMR (125.8 MHz, DMSO-*d*_6_): δ = 20.0, 20.2, 24.9, 26.1, 31.6, 32.4, 35.8, 36.1, 65.6, 67.7, 77.3, 87.6; HR-MS (ESI): *m*/*z* calcd for C_12_H_24_NO_2_ [M + H]^+^: 214.18016; found: 214.17979.

(4a*S*,7*S*,8a*R*)-1-Benzyl-7-isopropyloctahydro-1*H*-benzo[d][1,3]oxazine-4a-ol **25a**

Prepared from **23a** according to the general method. Yield: 0.149 g (89%); yellowish-brown transparent oil; [α${]}_{20}^{D}$ = −45 (c 0.255, MeOH); ^1^H NMR (DMSO-*d*_6_, 500.2 MHz): δ = 0.77 (3H, d, *J* = 7.4 Hz, CHMe_2_), 0.79 (3H, d, *J* = 7.4 Hz, CHMe_2_), 1.22–1.50 (CH_2_(3, m, CH_2_(3), H7, CH_2_(5)), 1.52–1.60 (1H, m, H8_α_), 1.80–1.87 (1H, m H8_β_), 2.16–2.27 (2H, m H8a), 3.08 (1H, d, *J* = 14.3 Hz, H4_α_), 3.12 (1H, d, *J* = 10.6 Hz CH_2_Ph), 3.42 (1H, d, *J* = 10.5 Hz CH_2_Ph), 3.53 (1H, d, *J* = 7.8 Hz H2), 3.86 (1H, d, *J* = 14.3 Hz H4_β_), 4.20 (1H, d, *J* = 7.7 Hz H2), 4.47 (1H, s, OH), 7.21–7.35 (5H, m, 5 × CH_Ar_); ^13^C NMR (125.8 MHz, DMSO-*d*_6_): δ = 20.0 (CH_2_Me_2_), 20.2 (CH_2_Me_2_), 25.0 (C5), 26.1 (C6), 31.6 (CHMe_2_), 32.1 (C5), 36.4 (C7), 52.0 (CH_2_Ph), 65.4 (C8a), 66.0 (C4a), 77.3 (C4), 85.2 (C2), 127.3 (CH_Ar_), 128.7 (2 × CH_Ar_), 128.8 (2 × CH_Ar_), 139.2 (C_qAr_); HR-MS (ESI): *m*/*z* calcd for C_18_H_28_NO_2_ [M + H]^+^: 290.21146; found: 290.21082.

(4a*R*,7*S*,8a*R*)-1-Benzyl-7-isopropyloctahydro-1*H*-benzo[d][1,3]oxazine-4a-ol **25b**

Prepared from **23b** according to the general method. Yield: 0.134 g (80%); yellowish-brown transparent oil; [α${]}_{20}^{D}$ = −21 (c 0.250, MeOH); ^1^H NMR (DMSO-*d*_6_, 500.2 MHz): δ = 0.79 (3H, d, *J* = 7.8 Hz), 0.83 (3H, d, *J* = 7.8 Hz), 1.20–1.53 (5H, m), 1.56–1.66 (1H, m), 1.79–1.87 (1H, m), 2.16–2.29 (2H, m), 3.09 (1H, d, *J* = 14.3 Hz), 3.12 (1H, d, *J* = 10.6 Hz), 3.42 (1H, d, *J* = 10.5 Hz), 3.53 (1H, d, *J* = 7.8 Hz), 3.86 (1H, d, *J* = 14.3 Hz), 4.20 (1H, d, *J* = 7.7 Hz), 4.47 (1H, s), 7.21–7.35 (5H, m); ^13^C NMR (125.8 MHz, DMSO-*d*_6_): δ = 20.0, 20.2, 25.0, 26.1, 31.6, 32.1, 36.4, 52.0, 65.4, 66.0, 77.3, 85.2, 127.3, 128.7, 128.8, 139.2; HR-MS (ESI): *m*/*z* calcd for C_18_H_28_NO_2_ [M + H]^+^: 290.21146; found: 290.21123.

**General procedure for the reaction of benzaldehyde with diethylzinc in the presence of chiral catalysts.**

A solution of Et_2_Zn in *n*-hexane (1M, 4.5 mL) was added to the appropriate catalyst (10 mol%) under an Ar atmosphere at room temperature. The reaction mixture was stirred for 20 min at room temperature, and then benzaldehyde (0.156 g, 153 μL, 1.5 mmol) was added. The mixture was stirred for a further 20 h at room temperature, then quenched with a saturated solution of NH_4_Cl (50 mL) and extracted with EtOAc (2 × 30 mL). The combined organic layer was dried (Na_2_SO_4_), filtered, and evaporated. The *ee* values and absolute configurations of the obtained secondary alcohols were determined by chiral-phase GC by using a CHIRASIL-DEX CB column, at 90 °C, after *O*-acetylation in an AcO_2_/4-dimethylaminopyridine/pyridine system.

Identification of **27b**–**d** was achieved by chiral HPLC analysis on a Chiralcel OD-H column and the data are as follows: 1-(4-tolyl)-1-propanol **27b** *V*(*n*-hexane)/*V*(2-propanol) = 95:5, 0.5 mL/min, *t*_R1_ = 16.0 min for *R*-isomer, *t*_R2_ = 22.2 min for *S*-isomer. 1-(4-methoxyphenyl)-1-propanol **27c**; *V*(*n*-hexane)/*V*(2-propanol) = 95:5, 0.7 mL/min, 210 nm, *t*_R1_ = 15.9 min for *R*-isomer, *t*_R2_ = 18.0 min for *S*-isomer. 1-(3-Methoxyphenyl)-1-propanol **27d**; *V*(*n*-hexane)/*V*(2-propanol) = 98:2, 0.4 mL/min, 210 nm, *t*_R1_ = 74.9 min for *R*-isomer, *t*_R2_ = 77.8 min for *S*-isomer (Figures S157–S162).

**DFT calculations on compounds 10a and 14a**

All DFT calculations were carried out by Gaussian 09 Revision A.02 software (Gaussian Incorporation, Pittsburgh, PA, USA), package [Gaussian 09], using the M06-2X global hybrid DFT functional [38] and 6-31+G(d,p) basis set [39]. Structural optimisations and subsequent frequency calculations were supported by the IEFPCM solvent model [40] parameterised with the dielectric constant of water (ε = 78.4) to represent the approximate polarity of the experimental reaction conditions. The Gibbs free-energy values of optimised structures were obtained by correcting the computed total energy with zero-point vibrational energy (ZPE) and the calculated thermal corrections. The optimised structures are available from the authors.

## Figures and Tables

**Scheme 10:**
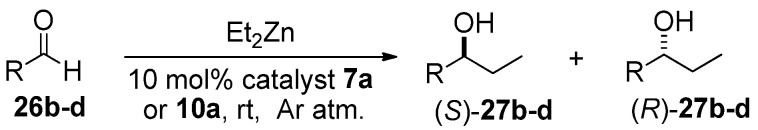
Model reaction of diethylzinc with aromatic aldehydes. Reactions and conditions: Et_2_Zn, 10 mol% catalyst, *n*-hexane, Ar atm., rt, 20 h, **b**: 4-MeC_6_H_4_, **c**: (4-MeO)C_6_H_4_, **d**: (3-MeO)C_6_H_4_, 80–90%.

**Table 1:** Addition of diethylzinc to benzaldehyde **26a**, catalysed by aminodiol derivatives.

Entry	Ligand ^a^	Yield ^b^ [%]	*ee* ^c^ [%]	Configuration of Major Isomer ^d^
1	**7a**	87	68	*S*
2	**7b**	80	60	*S*
3	**7c**	80	35	*S*
4	**8**	90	70	*S*
5	**9**	89	60	*R*
6	**10a**	85	94	*R*
7	**10b**	88	81	*R*
8	**10c**	84	86	*R*
9	**11a**	77	26	*R*
10	**11b**	85	68	*R*
11	**11c**	86	42	*S*
12	**12**	88	10	*S*
13	**13**	89	28	*S*
14	**21a**	92	4	*S*
15	**21b**	91	54	*S*
16	**21c**	67	12	*S*
17	**22**	75	20	*S*
18	**23a**	51	0	*-*
19	**23b**	71	18	*R*
20	**24**	82	20	*R*
21	**25a**	78	62	*R*
22	**25b**	89	8	*R*

^a^ 10 mol%. ^b^ Yields were measured after silica column chromatography. ^c^ Determined on the crude product by GC (Chirasil-DEX CB column). ^d^ Determined by comparing the R_t_ of the GC analysis and the optical rotations with literature data.

**Table 2:** Addition of diethylzinc to aromatic aldehydes **26b**–**d**, catalysed by aminodiol derivatives.

Entry	R	Ligand ^a^	Yield ^b^ [%]	*ee* ^c^ [%]	Configuration of Major Isomer ^d^
1	4-MeC_6_H_4_	**7a**	80	89	*R*
2	(4-MeO)C_6_H_4_	**7a**	85	52	*S*
3	(3-MeO)C_6_H_4_	**7a**	87	42	*S*
4	4-MeC_6_H_4_	**10a**	90	95.5	*R*
5	(4-MeO)C_6_H_4_	**10a**	89	95	*R*
6	(3-MeO)C_6_H_4_	**10a**	86	99	*R*

^a^ 10 mol%. ^b^ Yields were measured after silica column chromatography. ^c^ Determined on the crude product by chiral HPLC analysis on a Chiralcel OD-H column. ^d^ Determined by comparing the R_t_ of the HPLC analysis and the optical rotations with literature data [23].

## Data Availability

No new data were created or analyzed in this study. Data sharing is not applicable to this article.

## Supplementary Materials

The following supporting information can be downloaded at: https://www.mdpi.com/article/10.3390/ijms25084325/s1, references [41,42,43,44] are cited in Supplementary Materials.
