# Supplementary material for: Stereoselective Synthesis and Catalytical Application of Perillaldehyde-Based 3-Amino-1,2-diol Regioisomers
Source: Int J Mol Sci. 2024 Apr 13;25(8):4325. doi: 10.3390/ijms25084325 (PMC11050431; doi:10.3390/ijms25084325)
Supplement: Supplementary file 1 [file ijms-25-04325-s001.zip › ijms-2933297-supplementary.pdf]

Supporting information

for

Stereoselective synthesis and catalytical application of perillaldehyde-based 3-amino-1,2-diol regioisomers

Márton Háznagy [1,2], Antal Csámpai [3], Imre Ugrai [2], Barnabás Molnár [4], Matti Haukka [5] and Zsolt Szakonyi [2,*]

[1] Institute of Pharmacognosy, University of Szeged, H-6720 Szeged, Hungary; haznagy.marton.benedek@szte.hu
[2] Institute of Pharmaceutical Chemistry, University of Szeged, Interdisciplinary Excellence Center, H-6720 Szeged, Eötvös utca 6, Hungary; szakonyi.zsolt@szte.hu for Z.S.; ugrai.imre@gmail.com for I.U.
[3] Institute of Chemistry, Eötvös Loránd University, P.O. Box 32, H-1518 Budapest – 112, Hungary; antal.csampai@ttk.elte.hu
[4] Department of Molecular and Analytical Chemistry, Interdisciplinary Excellence Centre, University of Szeged, Dóm Tér 7-8, H-6720 Szeged, Hungary; barnabas.molnar@chem.u-szeged.hu
[5] Department of Chemistry, University of Jyväskylä, POB 35, 40351 Jyväskylä, Finland; matti.o.haukka@jyu.fi

* Correspondence: szakonyi.zsolt@szte.hu; Tel.: +36-62-546809

Contents

X-Ray structure determination of **7a**

^{1}H-, ^{13}C- NMR, and 2D NMR spectra of compound **3a**	Figure S1 – S3
^{1}H-, ^{13}C- NMR, and 2D NMR spectra of compound **3b**	Figure S4 – S8
^{1}H-, ^{13}C- NMR, and 2D NMR spectra of compound **3c**	Figure S9 – S13
^{1}H-, ^{13}C- NMR spectra of compound **4a**	Figure S14 – S15
^{1}H-, ^{13}C- NMR spectra of compound **4b**	Figure S16 – S20
^{1}H-, ^{13}C- NMR, and 2D NMR spectra of compound **4c**	Figure S21 – S25
^{1}H-, ^{13}C- NMR spectra of compound **5a**	Figure S26 – S27
^{1}H-, ^{13}C- NMR spectra of compound **6a**	Figure S28 – S29
^{1}H-, ^{13}C- NMR spectra of compound **5b**	Figure S30 – S31
^{1}H-, ^{13}C- NMR spectra of compound **6b**	Figure S32 – S33
^{1}H-, ^{13}C- NMR spectra of compound **5c**	Figure S34 – S35
^{1}H-, ^{13}C- NMR, and 2D NMR spectra of compound **6c**	Figure S36 – S39
^{1}H-, ^{13}C- NMR, and 2D NMR spectra of compound **7a**	Figure S40 – S44
^{1}H-, ^{13}C- NMR spectra of compound **7b**	Figure S45 – S46
^{1}H-, ^{13}C- NMR spectra of compound **7c**	Figure S47 – S48
^{1}H-, ^{13}C- NMR, and 2D NMR spectra of compound **11a**	Figure S49 – S54
^{1}H-, ^{13}C- NMR, and 2D NMR spectra of compound **11b**	Figure S55 – S59
^{1}H-, ^{13}C- NMR spectra of compound **11c**	Figure S60 – S61
^{1}H-, ^{13}C- NMR, and 2D NMR spectra of compound **8**	Figure S62 – S66
^{1}H-, ^{13}C- NMR, and 2D NMR spectra of compound **12**	Figure S67 – S71
^{1}H-, ^{13}C- NMR spectra of compound **9**	Figure S72 – S73
^{1}H-, ^{13}C- NMR spectra of compound **13**	Figure S74 – S75
^{1}H-, ^{13}C- NMR, and 2D NMR spectra of compound **10a**	Figure S76 – S81
^{1}H-, ^{13}C- NMR, and 2D NMR spectra of compound **10b**	Figure S82 – S86
^{1}H-, ^{13}C- NMR, and 2D NMR spectra of compound **10c**	Figure S87 – S91
^{1}H-, ^{13}C- NMR spectra of compound **16a-b**	Figure S92 – S93
^{1}H-, ^{13}C- NMR spectra of compound **18a-b**	Figure S94 – S95
^{1}H-, ^{13}C- NMR, and 2D NMR spectra of compound **19a**	Figure S96 – S99
^{1}H-, ^{13}C- NMR, and 2D NMR spectra of compound **19b-c**	Figure S100 – S102
^{1}H-, ^{13}C- NMR, and 2D NMR spectra of compound **20b**	Figure S103 – S106
^{1}H-, ^{13}C- NMR, and 2D NMR spectra of compound **20c**	Figure S107 – S110
^{1}H-, ^{13}C- NMR and NOESY spectra of compound **21a**	Figure S111 – S113
^{1}H-, ^{13}C- NMR, and NOESY spectra of compound **21b**	Figure S114 – S116
^{1}H-, ^{13}C- NMR, and NOESY spectra of compound **21c**	Figure S117 – S119
^{1}H-, ^{13}C- NMR, and 2D NMR spectra of compound **23a**	Figure S120 – S124

^{1}H-, ^{13}C- NMR, and 2D NMR spectra of compound **23b** Figure S125 – S130
^{1}H-, ^{13}C- NMR, and 2D NMR spectra of compound **22** Figure S131 – S134
^{1}H-, ^{13}C- NMR, and 2D NMR spectra of compound **25a** Figure S135 – S138
^{1}H-, ^{13}C- NMR spectra of compound **25b** Figure S139 – S140
^{1}H-, ^{13}C- NMR, and 2D NMR spectra of compound **24** Figure S141 – S144
Chiral GC determination of the ratio of **16a** and **16b** Figure S145
Chiral GC determination of the ratio of **18a** and **18b** Figure S146
Representative GC chromatograms of Et_2Zn/benzaldehyde model reactions Figure S147 – S156
HPLC chromatograms of the Et_2Zn/*p*-tolylaldehyde reactions Figure S157 – S158
HPLC chromatograms of the Et_2Zn/*p*-methoxybenzaldehyde reactions Figure S159 – S160
HPLC chromatograms of the Et_2Zn/*m*-methoxybenzaldehyde reactions Figure S161 – S162

X-Ray structure determination of compound 7a

The crystal of **7a** was immersed in cryo-oil, mounted in a loop, and measured at a temperature of 120 K. The X-ray diffraction data was collected on a Rigaku Oxford Diffraction Supernova diffractometer using Cu Kα radiation. The *CrysAlisPro*[1] software package was used for cell refinement and data reduction. An analytical absorption correction (*CrysAlisPro*[1]) was applied to the intensities before the structure solution. The structure was solved in the chiral space group $P2_1P2_12$ by direct methods (*SHELXS*[2]). Structural refinement was carried out using *SHELXL*[3] software with *SHELXLE*[4] graphical user interface. The NH_2 and OH hydrogen atoms were located from the difference Fourier map and refined isotropically. All other hydrogen atoms were positioned geometrically and constrained to ride on their parent atoms, with C-H = 0.95-1.00 Å and U_{iso} = 1.2-1.5·U_{eq}(parent atom). The crystallographic details are summarized in Table S1.

Table S1. Crystal Data.

	**7a**
CCDC	
empirical formula	$C_{17}H_{28}ClNO_2$
fw	313.85
temp (K)	120(2)
λ(Å)	1.54184
cryst syst	Orthorhombic
space group	$P2_12_12$
a (Å)	29.0568(6)
b (Å)	12.0858(2)
c (Å)	5.04192(7)
α(deg)	90
β (deg)	90
γ(deg)	90
V (Å^3)	1770.59(6)
Z	4
ρ_{calc} (Mg/m^3)	1.177
μ(Mo Kα) (mm^{-1})	1.936
No. reflns.	24785
Unique reflns.	3752
Completeness to θ=67.684°	99.9%
Absolute structure parameter	0.001(7)
GOOF (F^2)	1.031
R_{int}	0.0534
R1^a ($I \geq 2\sigma$)	0.0332
wR2^b ($I \geq 2\sigma$)	0.0776

^{a} $R1 = \Sigma||F_o| - |F_c||/\Sigma|F_o|$. $^b wR_2 = \{\Sigma[w(F_o^2 - F_c^2)^2]/\Sigma[w(F_o^2)^2]\}^{1/2}$

References

1. Rikagu Oxford Diffraction, *CrysAlisPro v. 1.171.37.35*, 2014, Rikagu Oxford Diffraction inc., Yarnton, Oxfordshire, England.
2. Sheldrick, G. M. *Acta Cryst.* **2015**, *A71*, 3-8.
3. Sheldrick, G. M. *Acta Cryst.* **2015**, *C71*, 3-8.
4. Hübschle, C. B.; Sheldrick, G. M.; Dittrich, B. *J. Appl. Cryst.* **2011**, *44*, 1281-1284.

Figure S1:

^{1}H-NMR of compound (*S*)-*N*-Benzyl-1-(4-Isopropylcyclohex-1-en-1-yl)methanamine hydrochloride **3a**

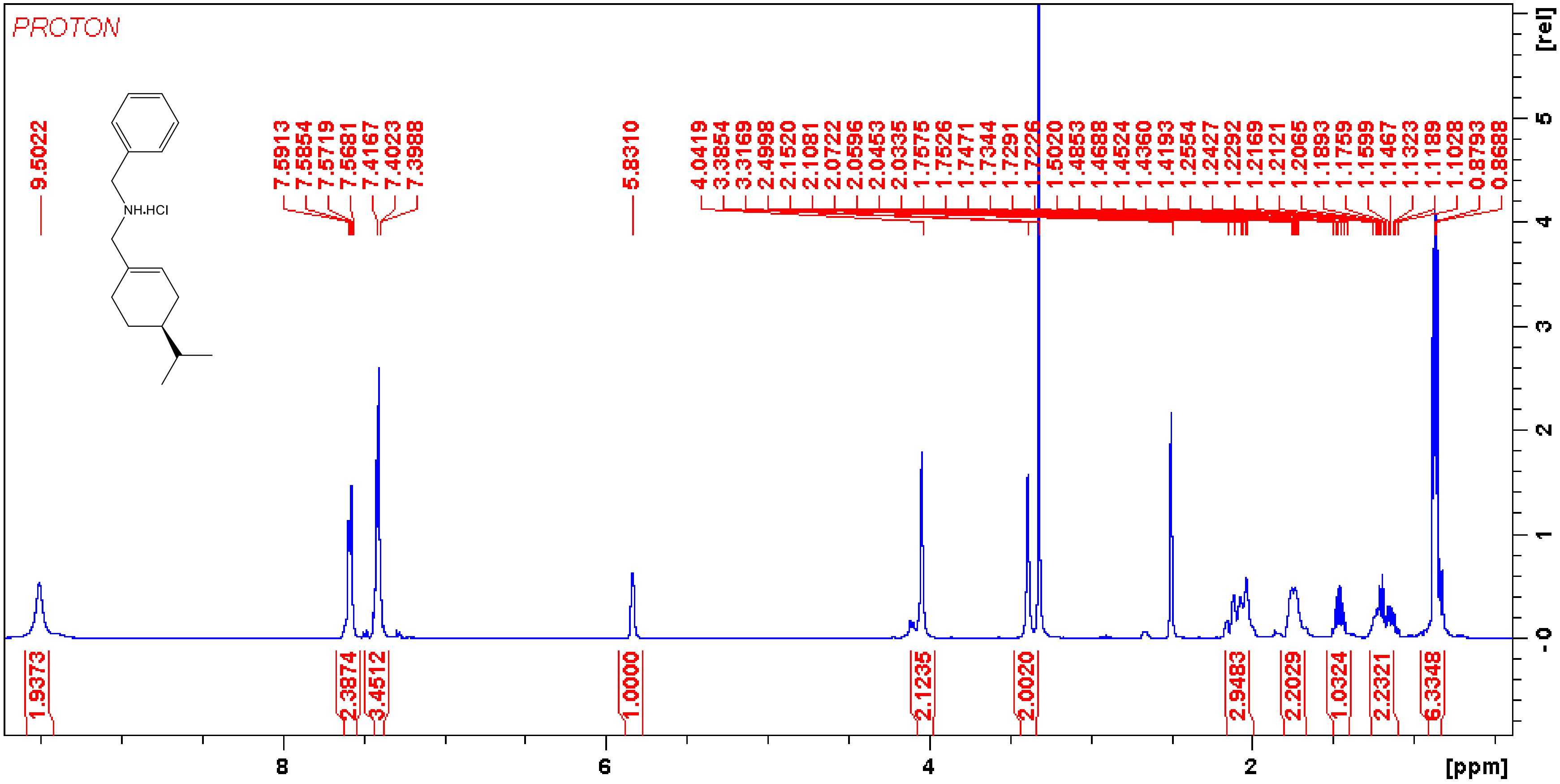

Figure S2:

^{13}C-NMR of compound (S)-N-Benzyl-1-(4-Isopropylcyclohex-1-en-1-yl)methanamine hydrochloride **3a**

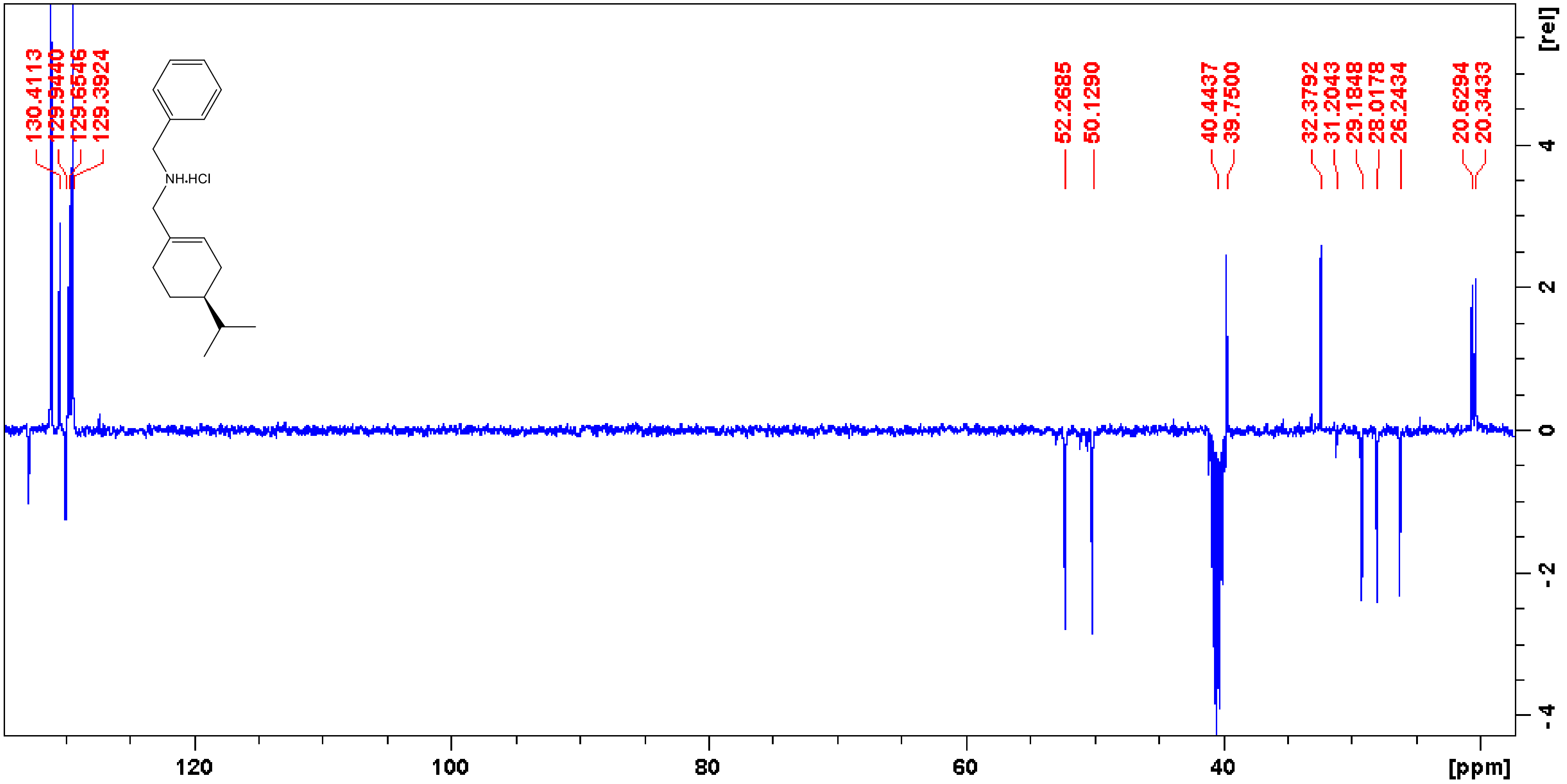

Figure S3:

COSY NMR of compound (*S*)-*N*-Benzyl-1-(4-Isopropylcyclohex-1-en-1-yl)methanamine hydrochloride **3a**

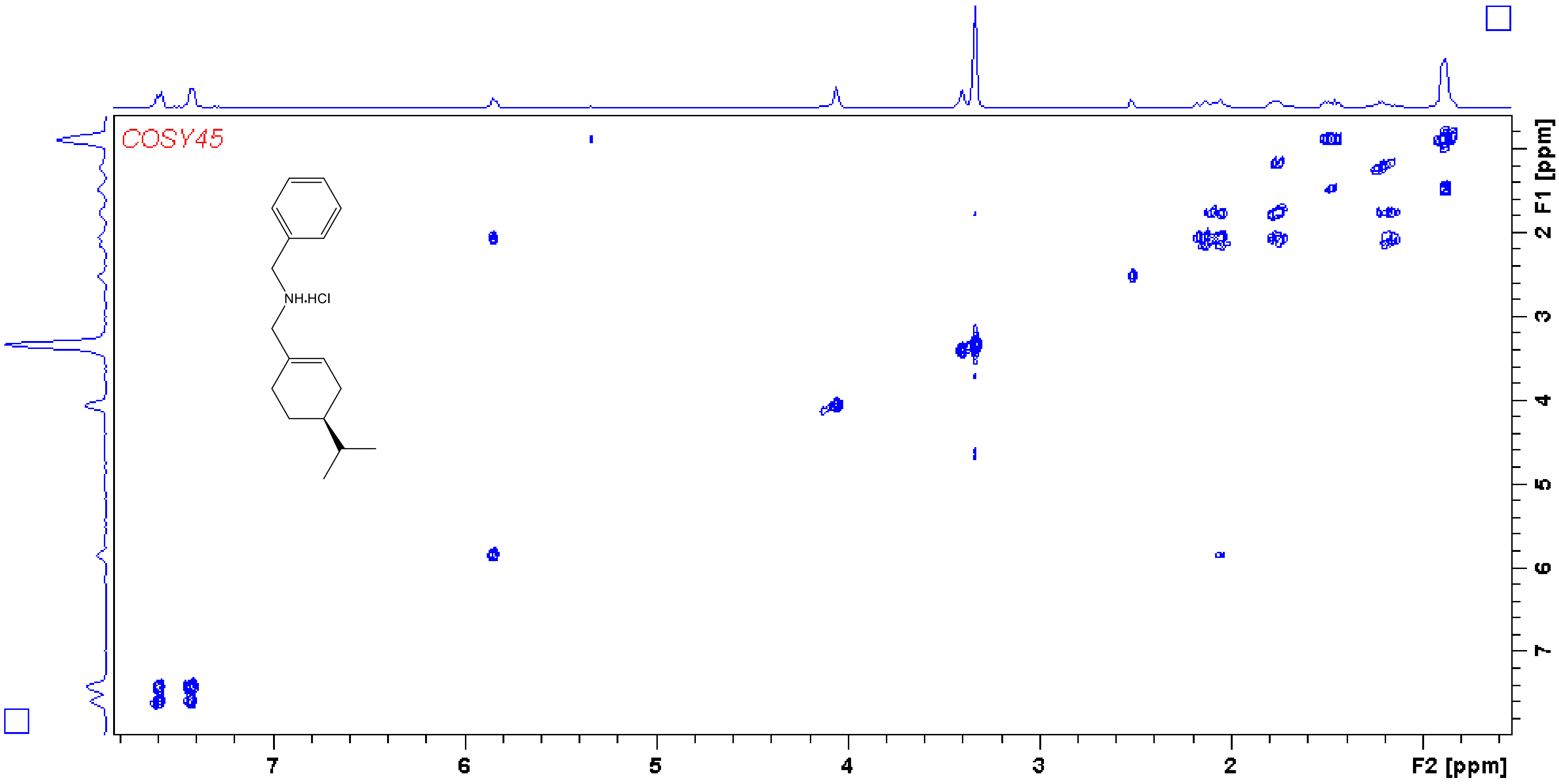

Figure S4:

^{1}H-NMR of compound (*R*)-*N*-(((*S*)-4-Isopropylcyclohex-1-en-1-yl)methyl)-1-phenylethanamine hydrochloride **3b**

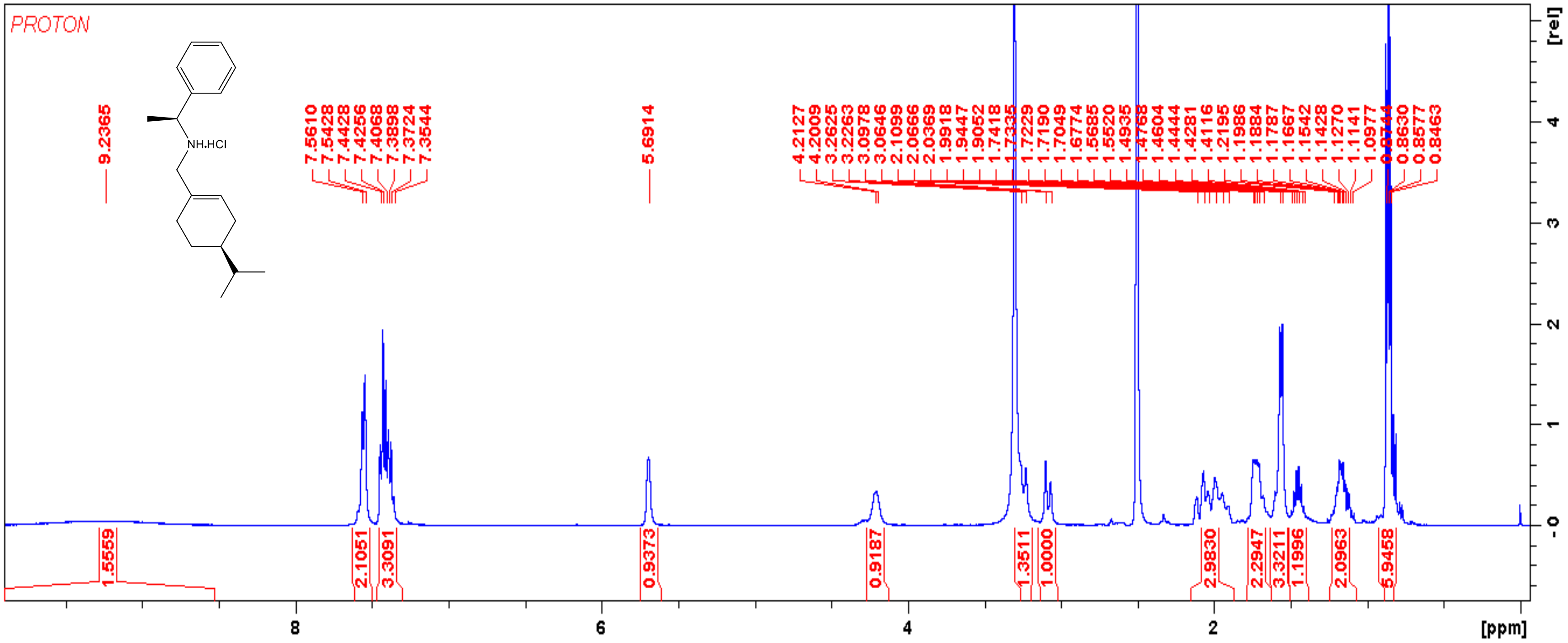

Figure S5:

^{13}C-NMR of compound (*R*)-*N*-(((*S*)-4-Isopropylcyclohex-1-en-1-yl)methyl)-1-phenylethanamine hydrochloride **3b**

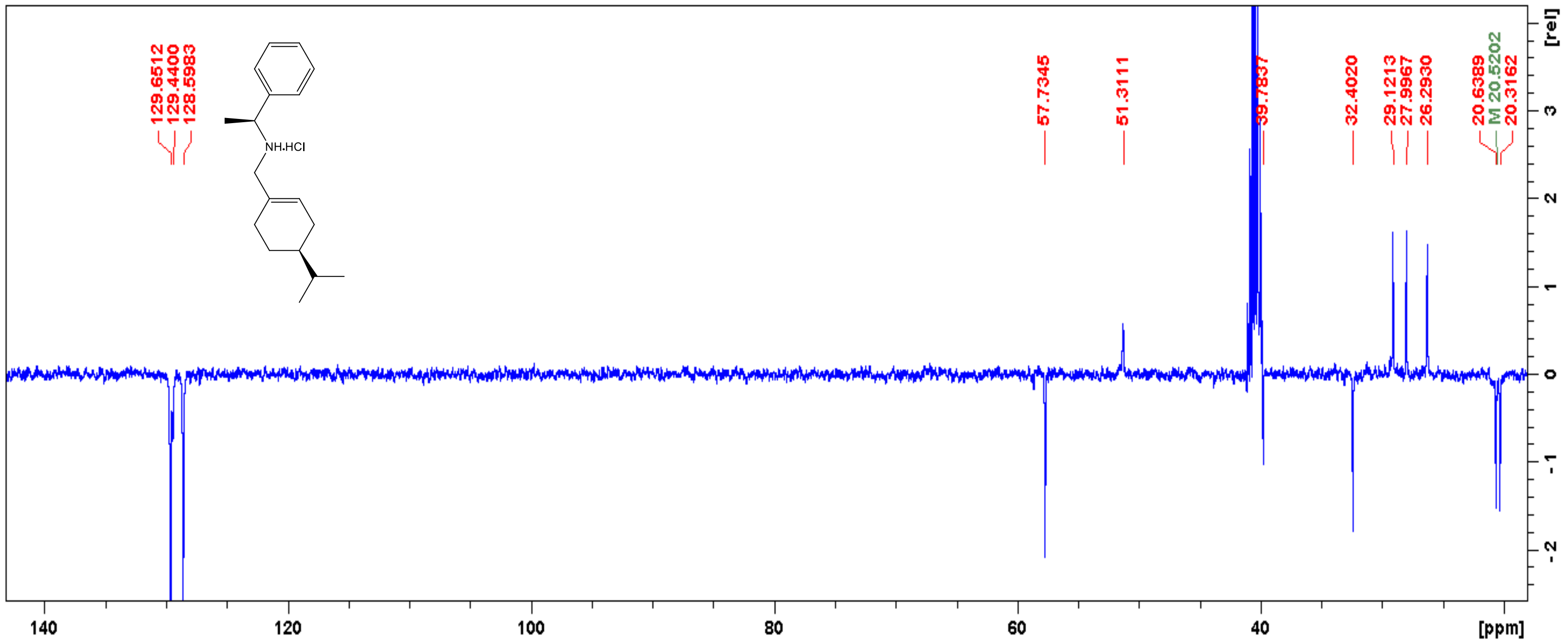

Figure S6:

COSY of compound (*R*)-*N*-(((*S*)-4-Isopropylcyclohex-1-en-1-yl)methyl)-1-phenylethanamine hydrochloride **3b**

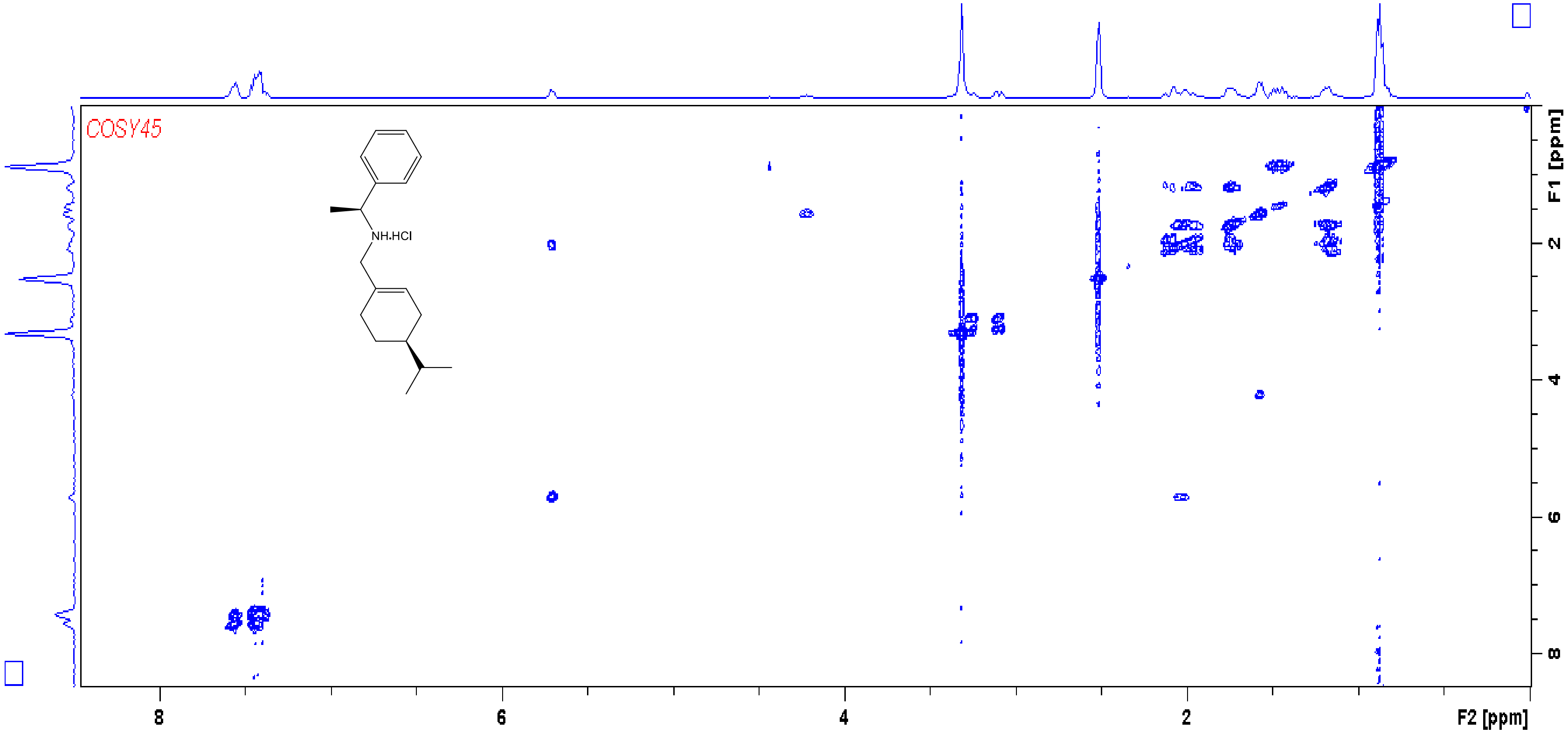

Figure S7:

HSQC NMR of compound (*R*)-*N*-(((*S*)-4-Isopropylcyclohex-1-en-1-yl)methyl)-1-phenylethanamine hydrochloride **3b**

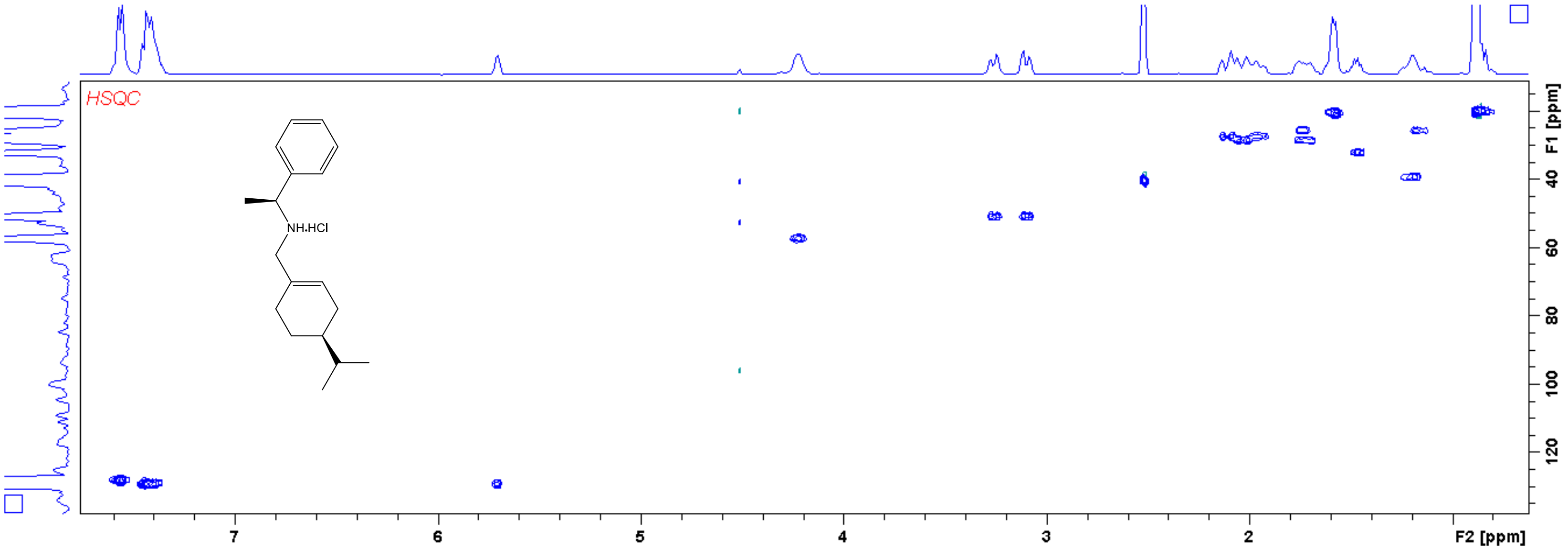

Figure S8:

HMBC NMR of compound (*R*)-*N*-(((*S*)-4-Isopropylcyclohex-1-en-1-yl)methyl)-1-phenylethanamine hydrochloride **3b**

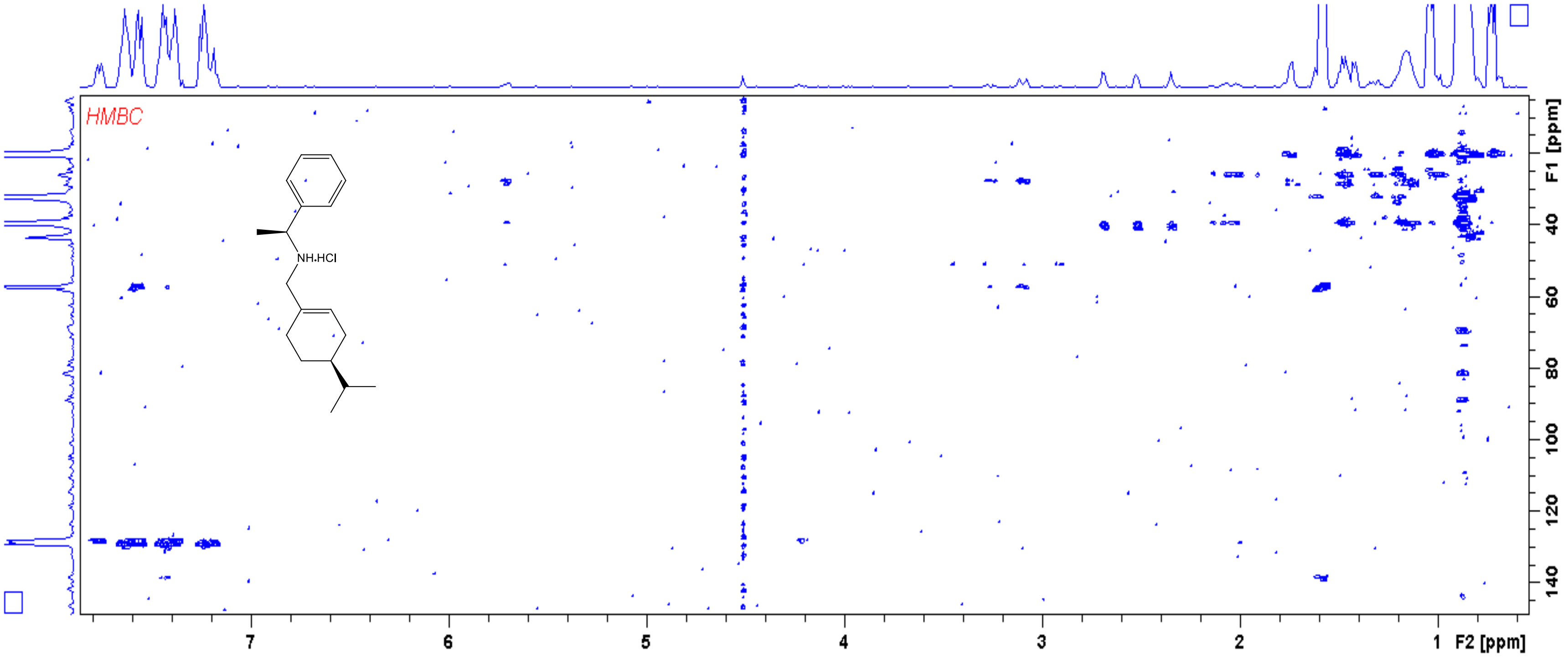

Figure S9:

^{1}H-NMR of compound (*S*)-*N*-(((*S*)-4-Isopropylcyclohex-1-en-1-yl)methyl)-1-phenylethanamine hydrochloride **3c**

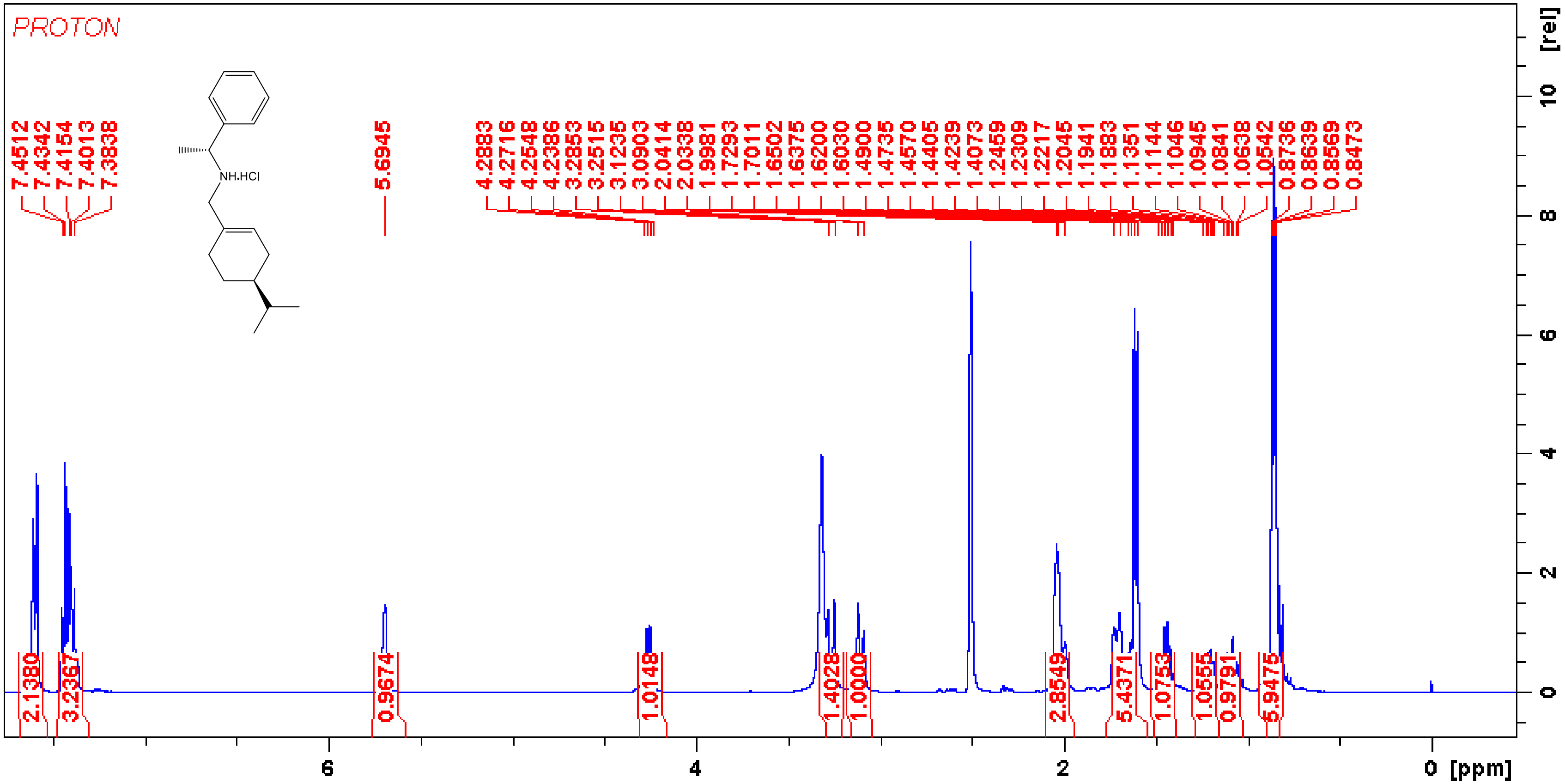

Figure S10:

^{13}C-NMR of compound (*S*)-*N*-(((*S*)-4-Isopropylcyclohex-1-en-1-yl)methyl)-1-phenylethanamine hydrochloride **3c**

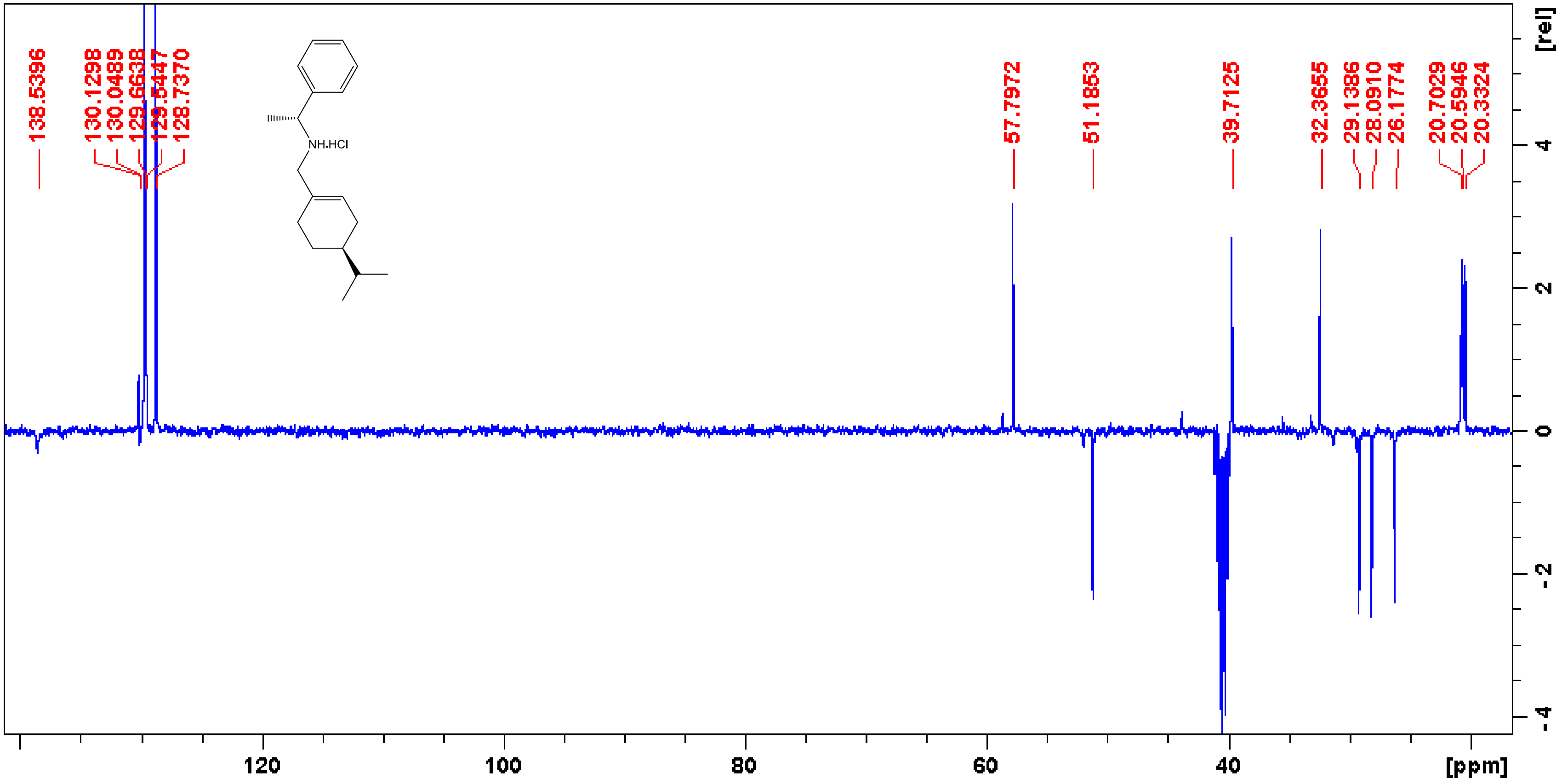

Figure S11:

COSY of compound (*S*)-*N*-(((*S*)-4-Isopropylcyclohex-1-en-1-yl)methyl)-1-phenylethanamine hydrochloride **3c**

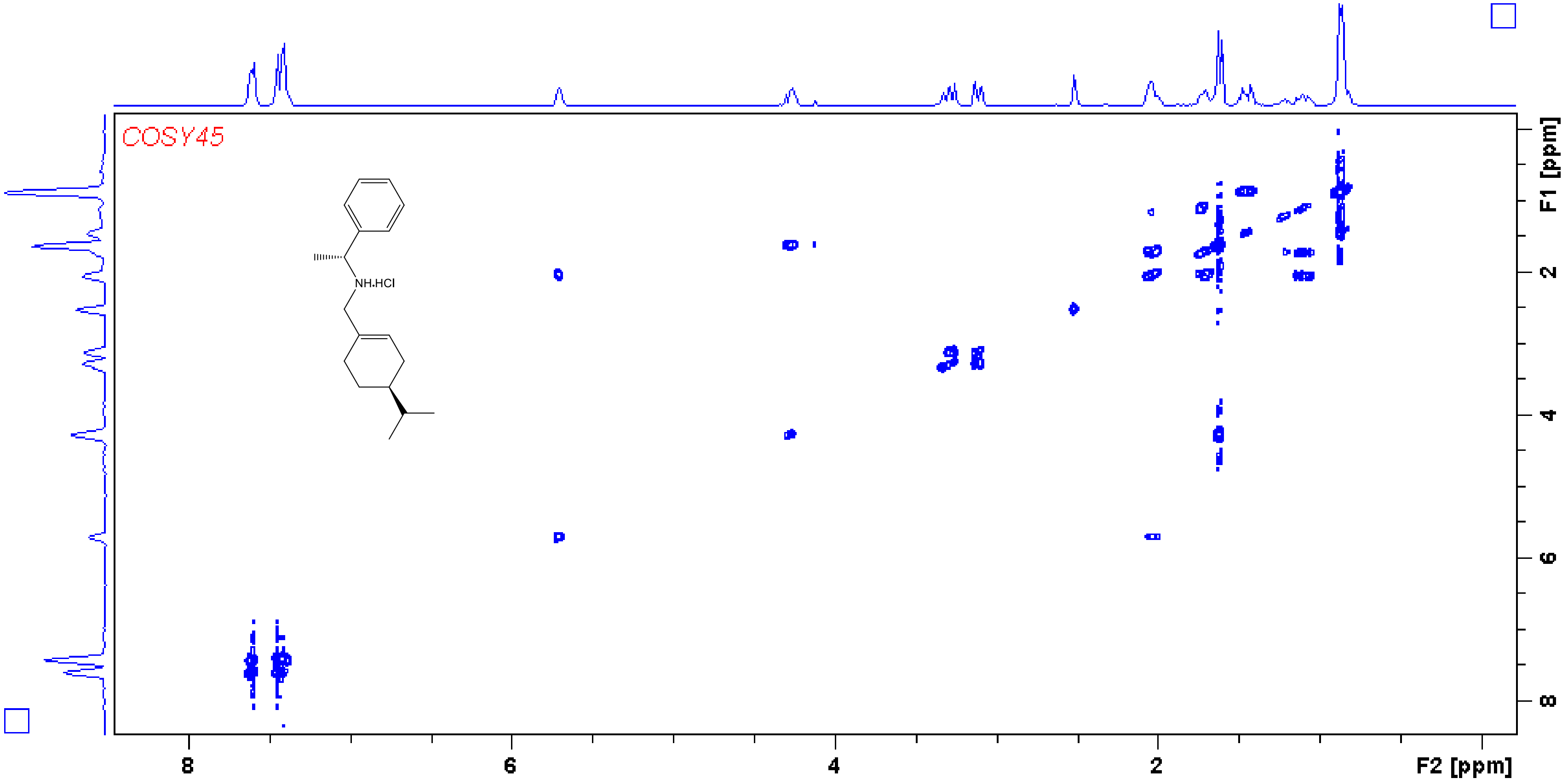

Figure S12:

HSQC of compound (*S*)-*N*-(((*S*)-4-Isopropylcyclohex-1-en-1-yl)methyl)-1-phenylethanamine hydrochloride **3c**

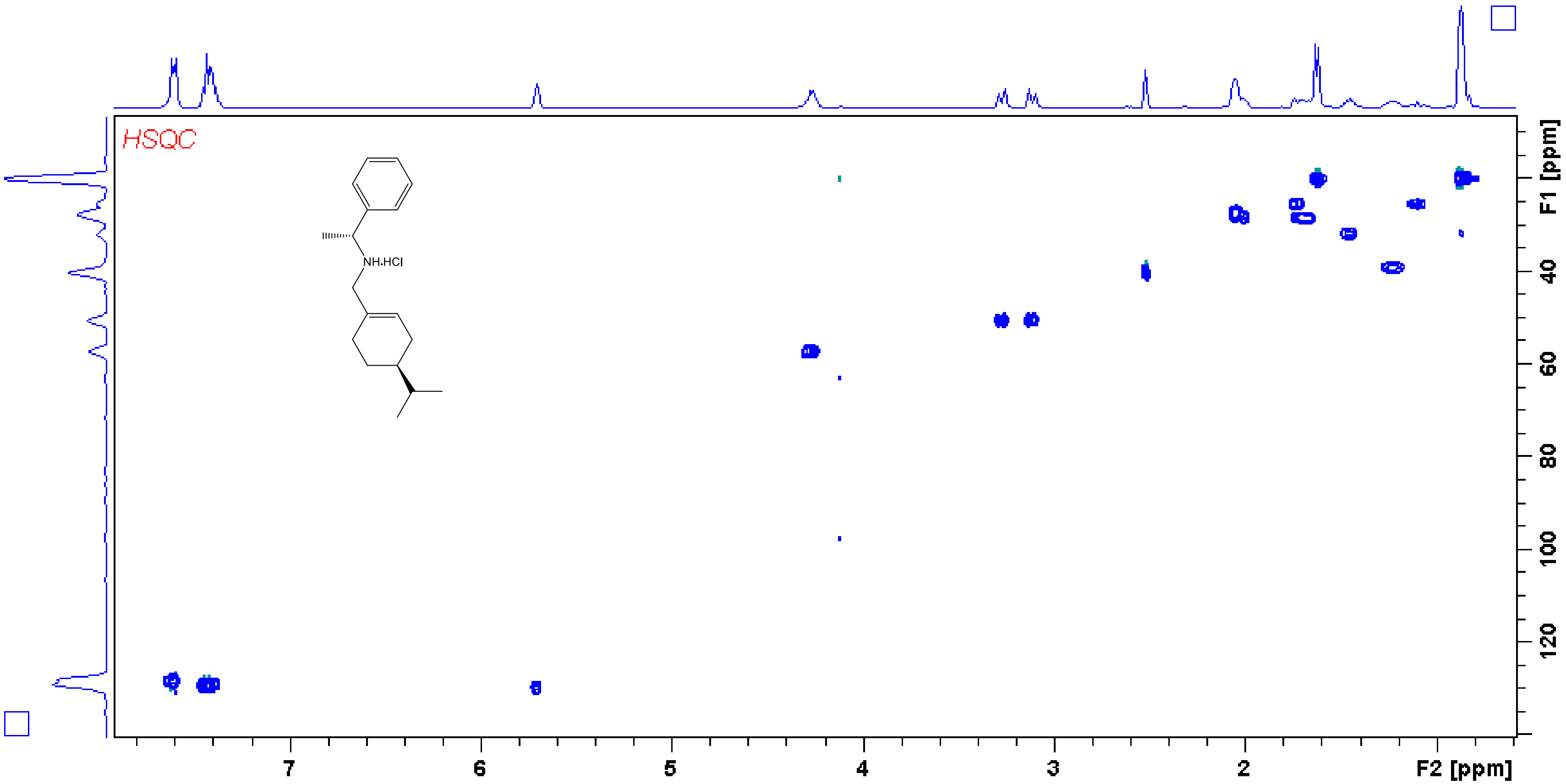

Figure S13:

HMBC of compound(*S*)-*N*-(((*S*)-4-Isopropylcyclohex-1-en-1-yl)methyl)-1-phenylethanamine hydrochloride **3c**

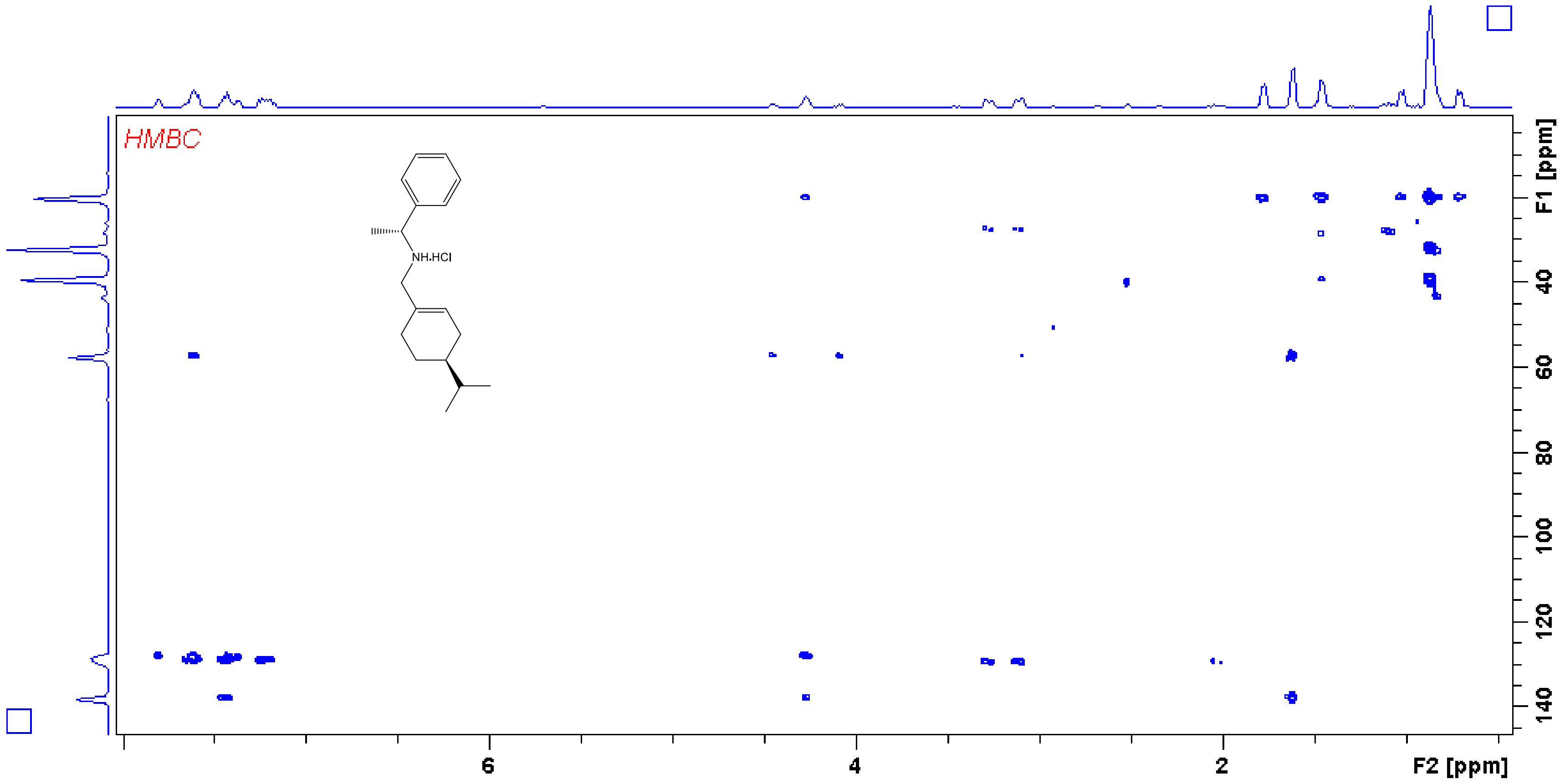

Figure S 14: ^{1}H-NMR of compound (*S*)-*tert*-butyl benzyl((4-isopropylcyclohex-1-en-1-yl)methyl)carbamate (**4a**

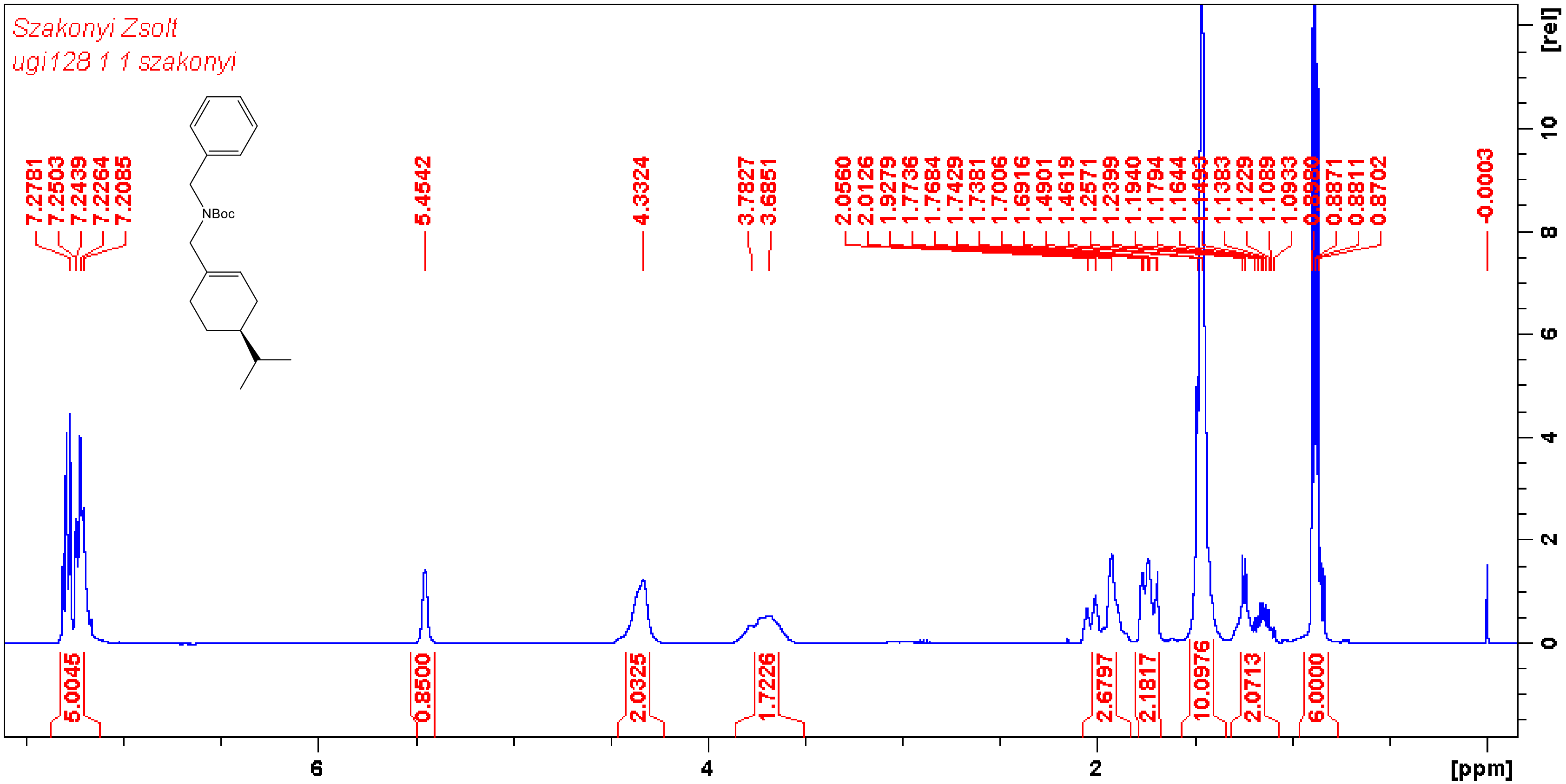

Figure S 15: ^{13}C-NMR of compound (*S*)-*tert*-butyl benzyl((4-isopropylcyclohex-1-en-1-yl)methyl)carbamate **4a**

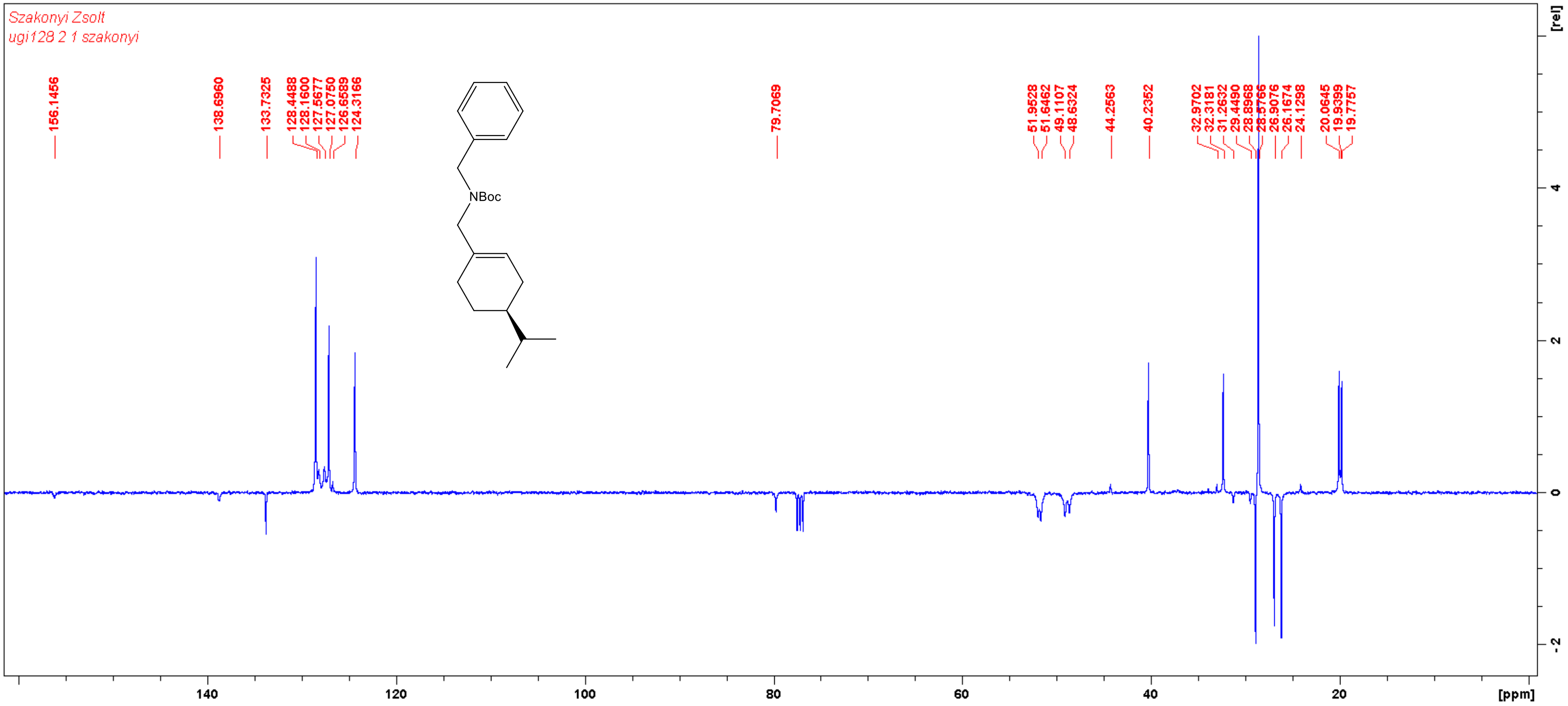

Figure S 16: ^{1}H-NMR of compound *tert*-butyl (((*S*)-4-isopropylcyclohex-1-en-1-yl)methyl)((*S*)-1-phenylethyl)carbamate **4b**

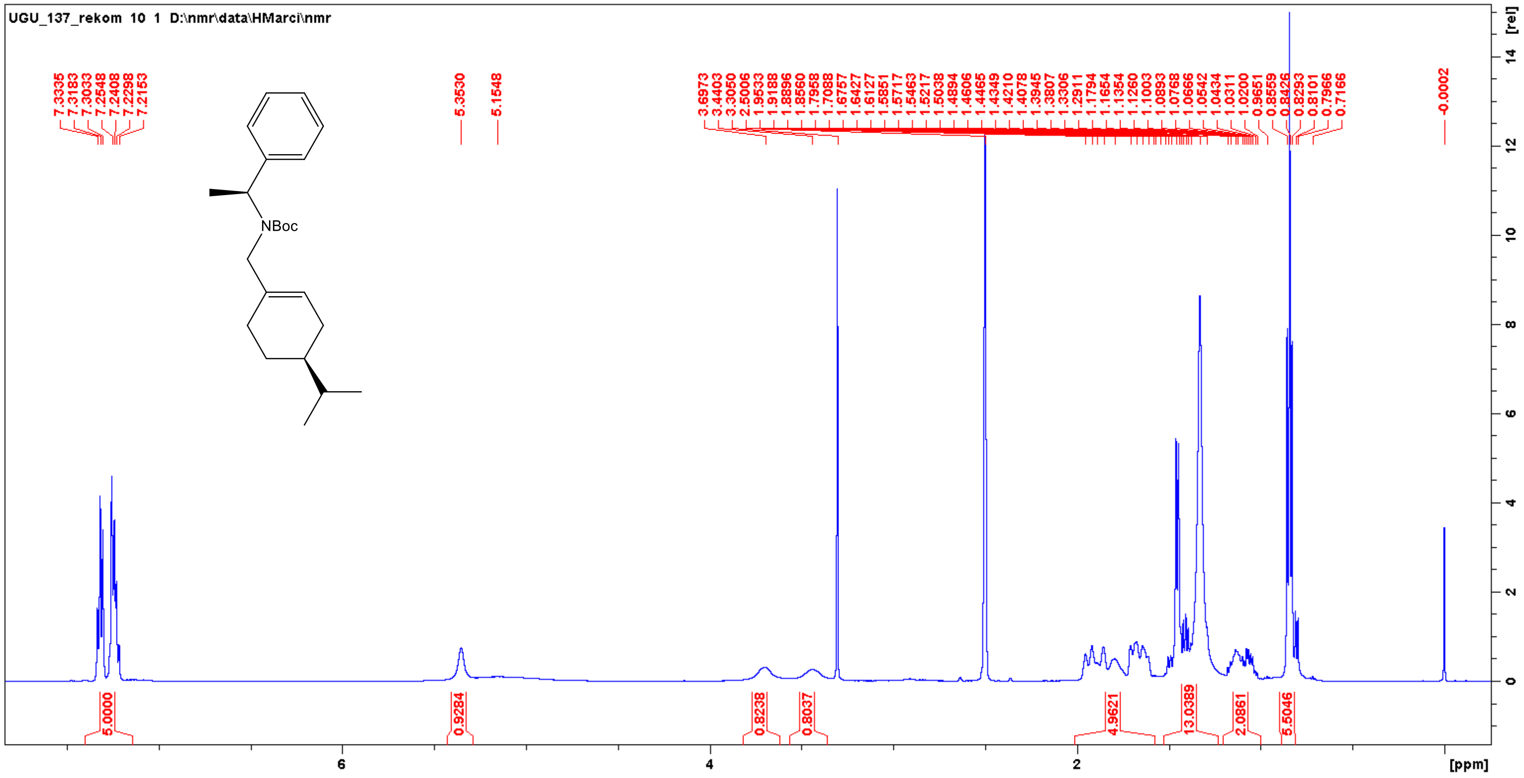

Figure S 17: ^{13}C-NMR of compound *tert*-butyl (((*S*)-4-isopropylcyclohex-1-en-1-yl)methyl)((*S*)-1-phenylethyl)carbamate **4b**

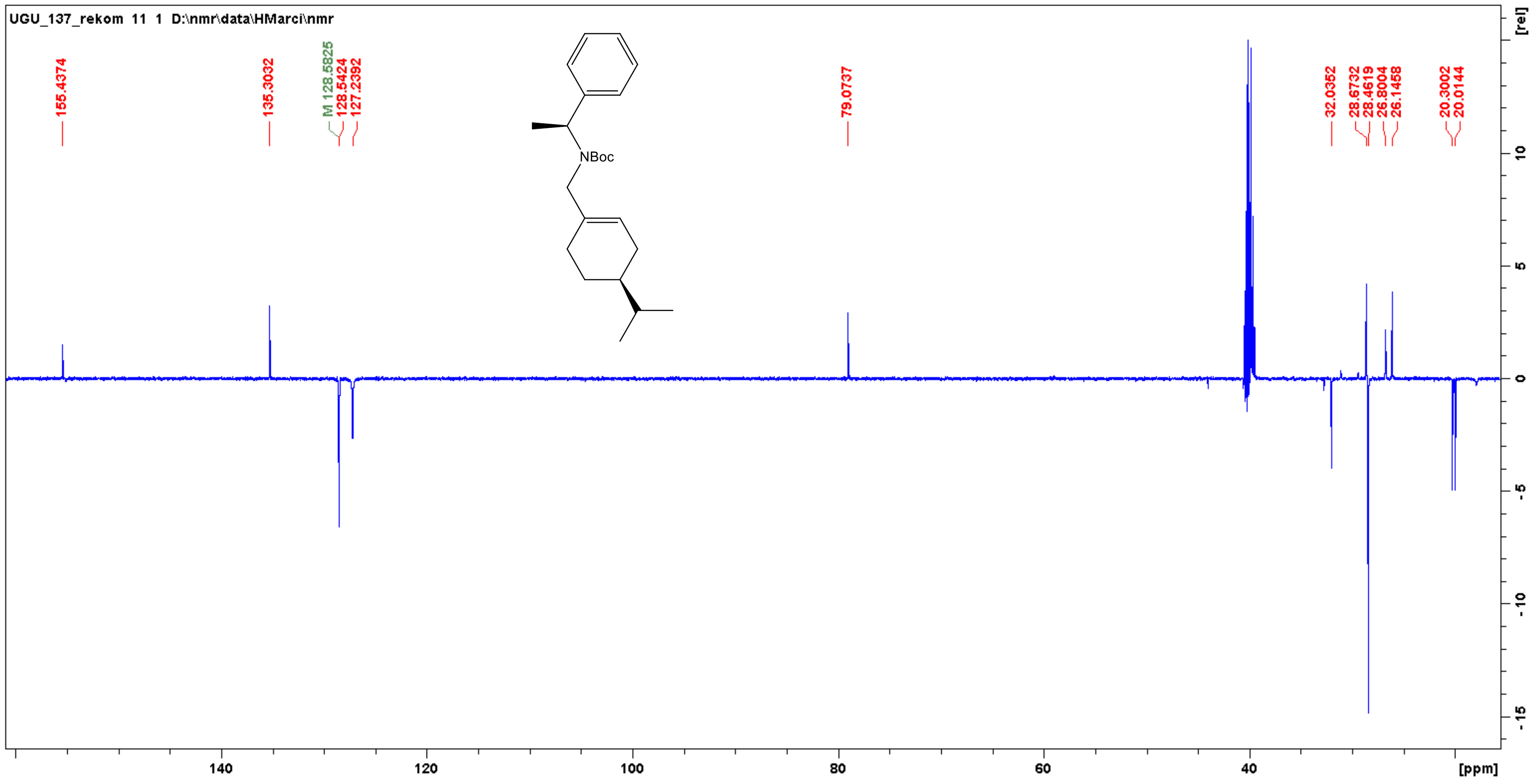

Figure S 18: COSY NMR of compound *tert*-butyl (((*S*)-4-isopropylcyclohex-1-en-1-yl)methyl)((*S*)-1-phenylethyl)carbamate **4b**

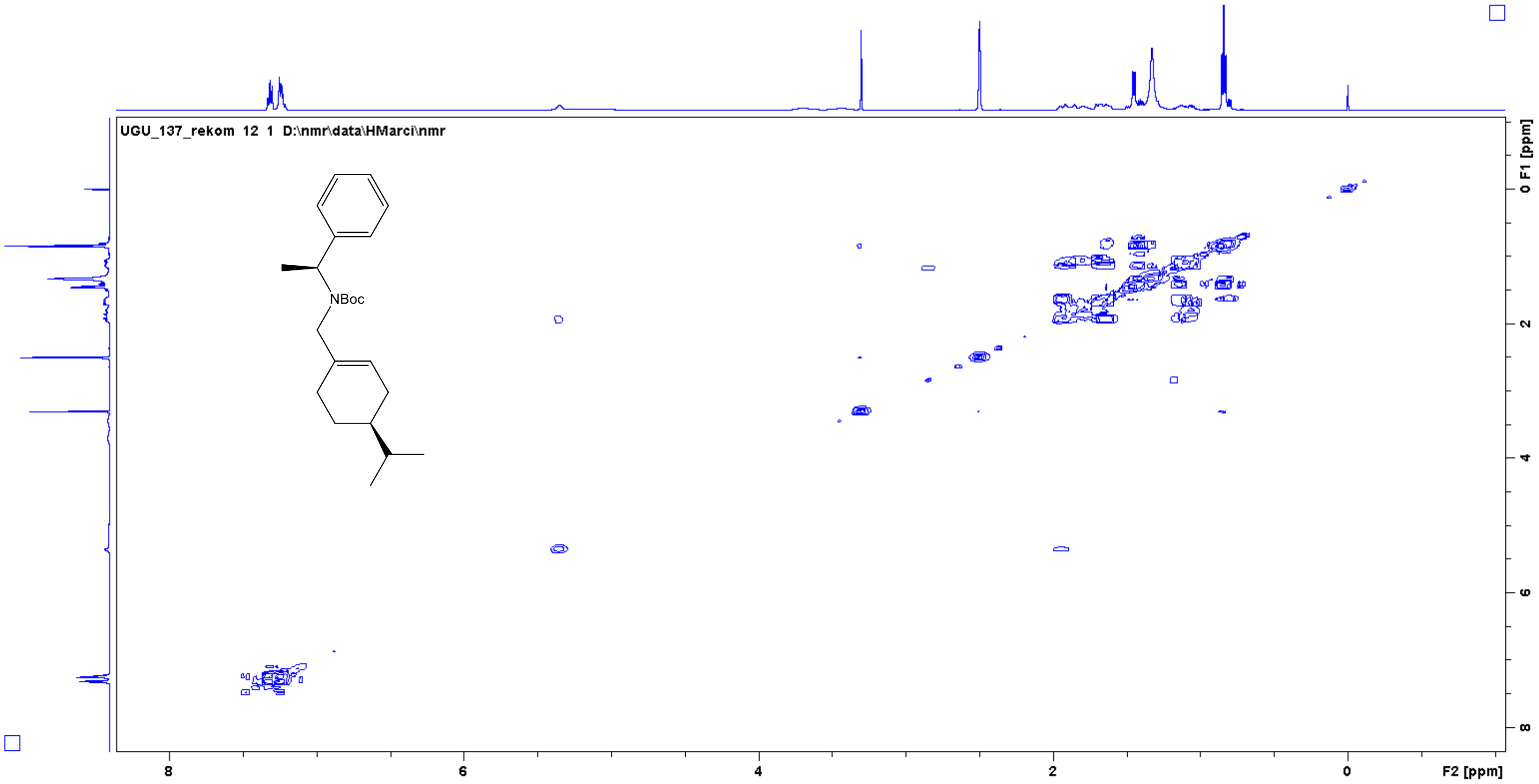

Figure S 19: HSQC NMR of compound *tert*-butyl (((*S*)-4-isopropylcyclohex-1-en-1-yl)methyl)((*S*)-1-phenylethyl)carbamate **4b**

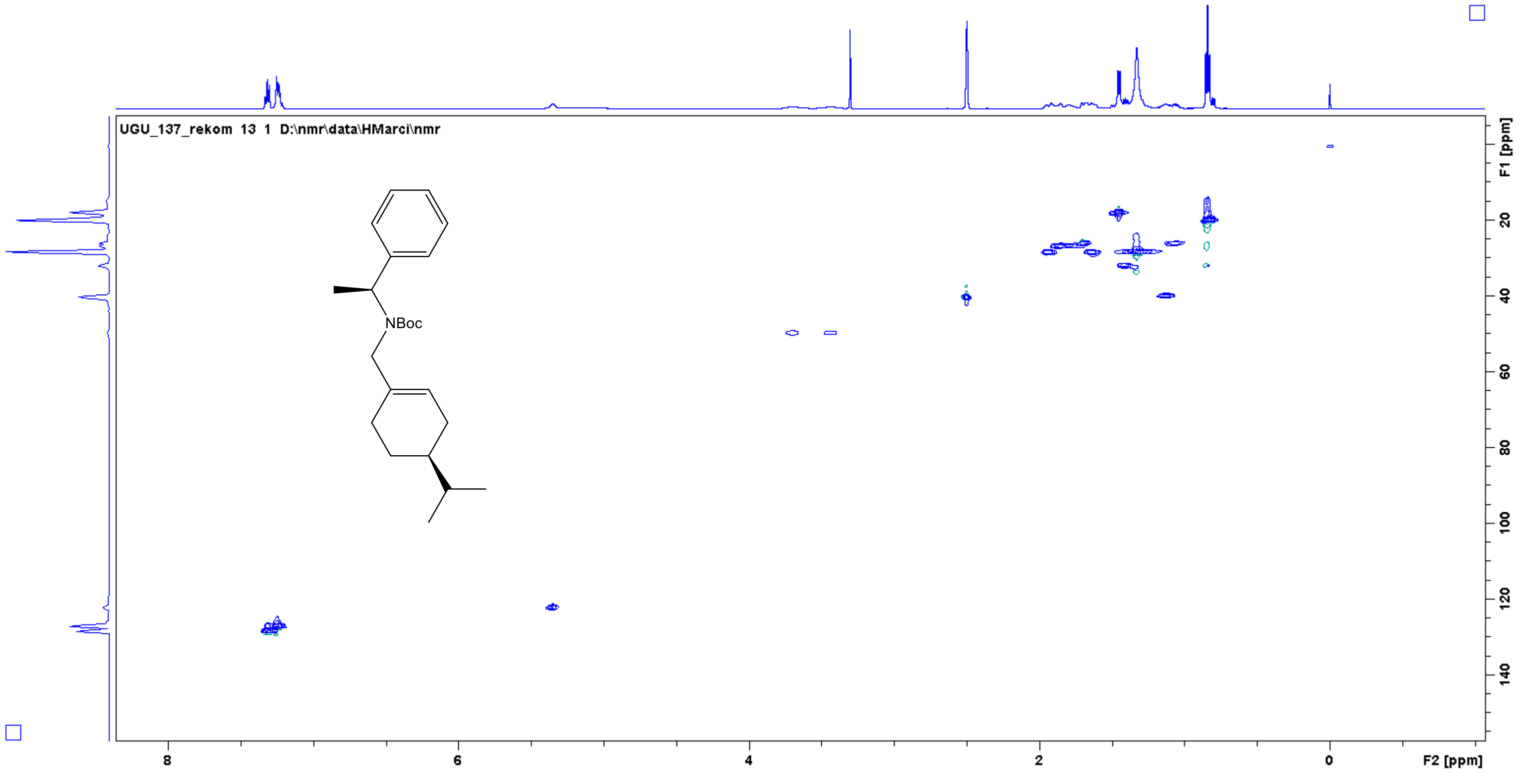

Figure S 20: HMBC NMR of compound *tert*-butyl (((*S*)-4-isopropylcyclohex-1-en-1-yl)methyl)((*S*)-1-phenylethyl)carbamate **4b**

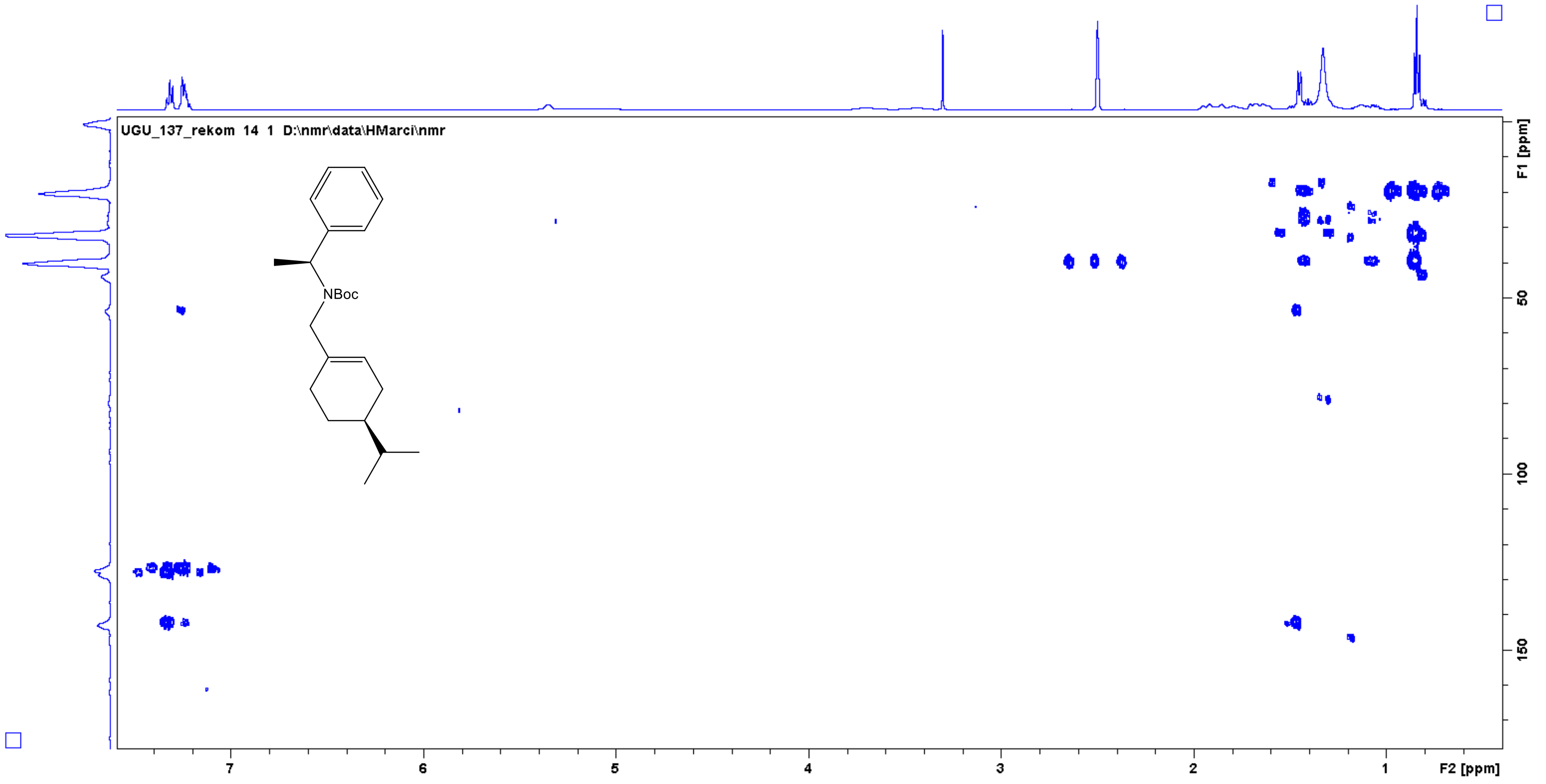

Figure S 21: ^{1}H-NMR of compound *tert*-butyl (((*S*)-4-isopropylcyclohex-1-en-1-yl)methyl)((*R*)-1-phenylethyl)carbamate **4c**

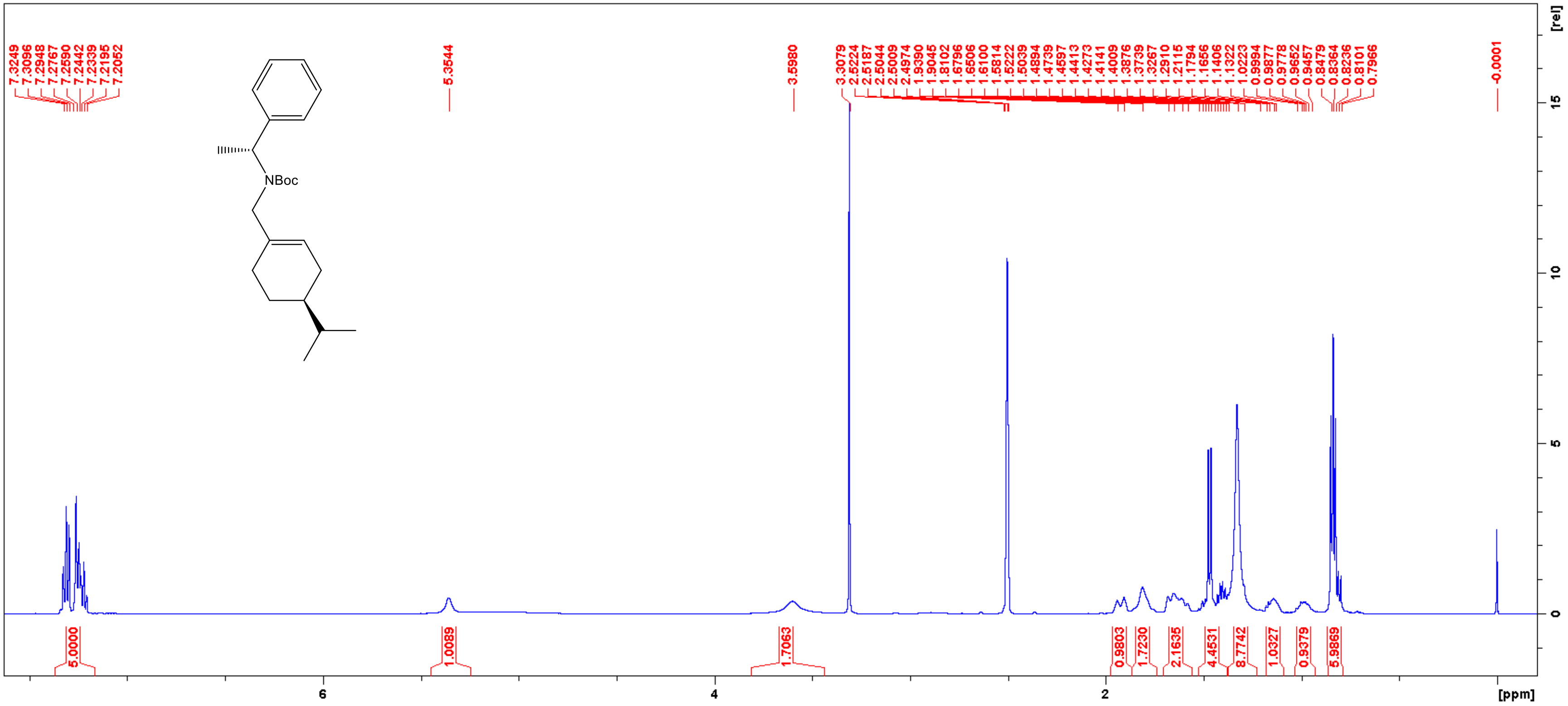

Figure S 22: ^{13}C-NMR of compound *tert*-butyl (((*S*)-4-isopropylcyclohex-1-en-1-yl)methyl)((*R*)-1-phenylethyl)carbamate **4c**

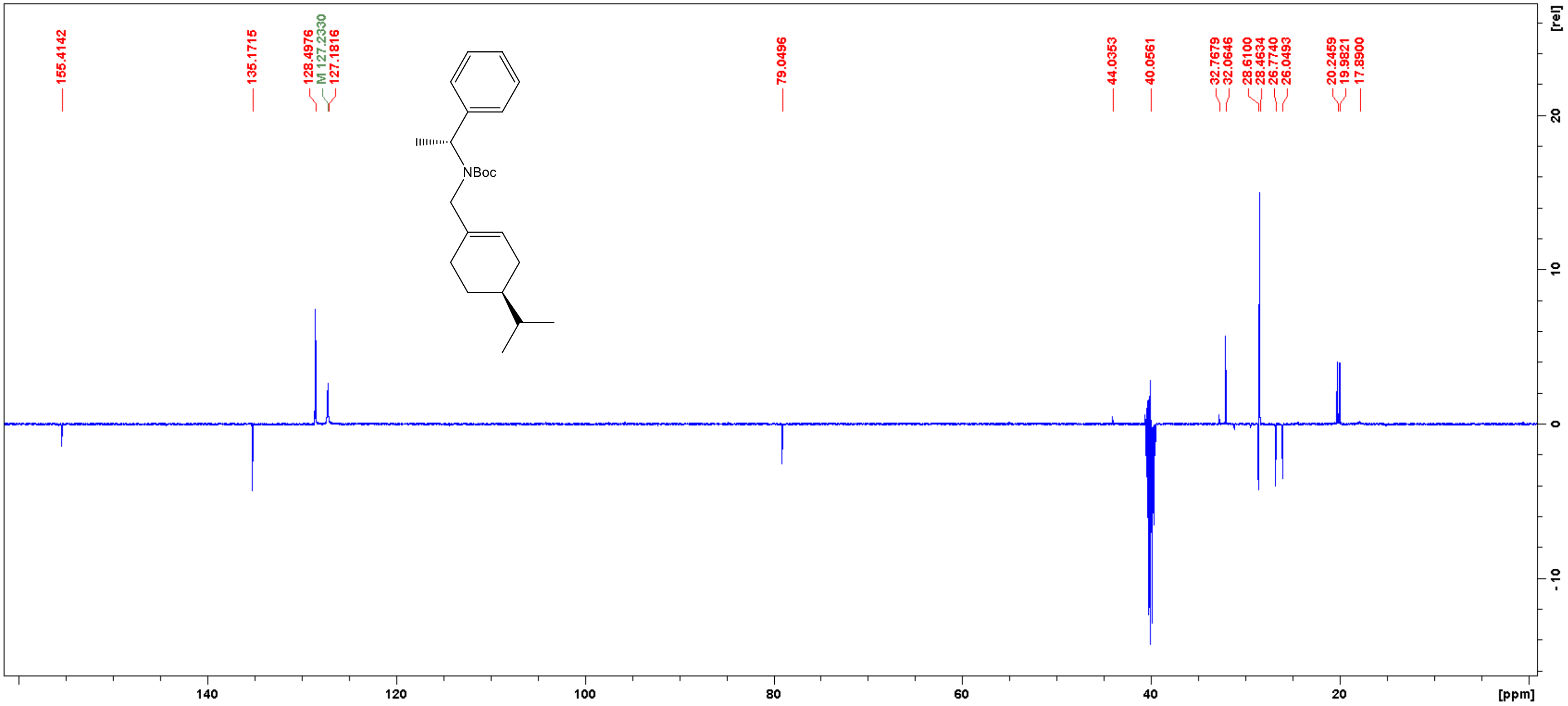

Figure S 23: COSY NMR of compound *tert*-butyl (((*S*)-4-isopropylcyclohex-1-en-1-yl)methyl)((*R*)-1-phenylethyl)carbamate **4c**

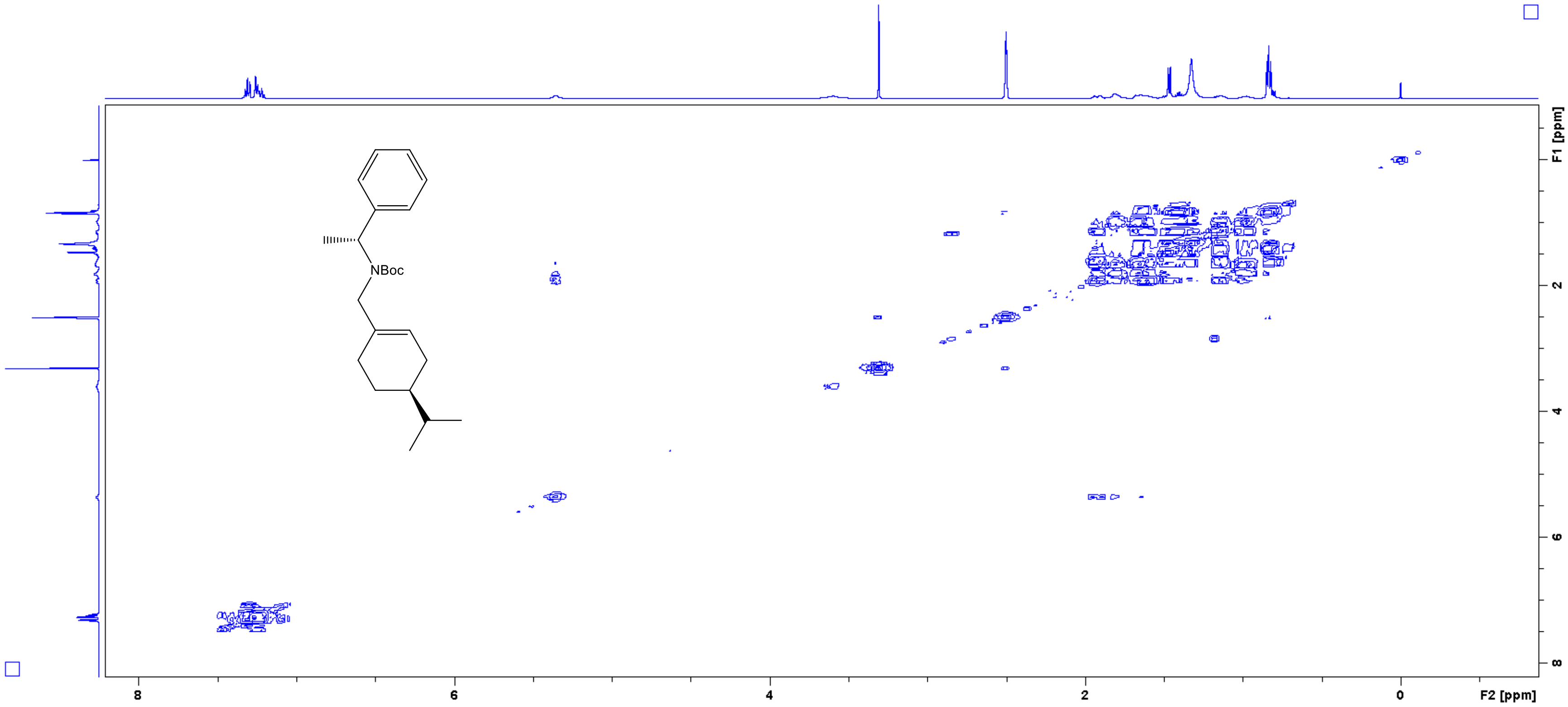

Figure S 24: HSQC NMR of compound *tert*-butyl (((*S*)-4-isopropylcyclohex-1-en-1-yl)methyl)((*R*)-1-phenylethyl)carbamate **4c**

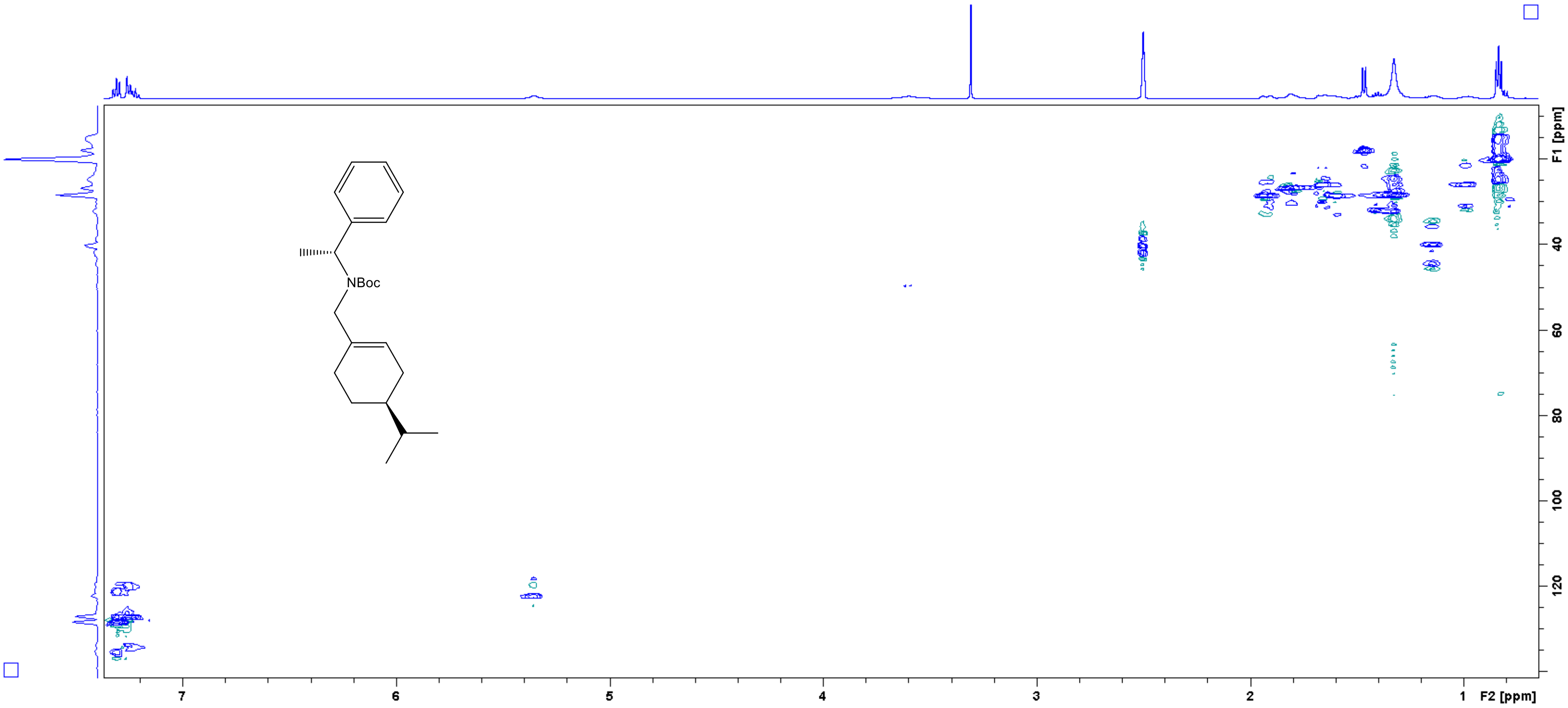

Figure S 25: HMBC NMR of compound *tert*-butyl (((*S*)-4-isopropylcyclohex-1-en-1-yl)methyl)((*R*)-1-phenylethyl)carbamate **4c**

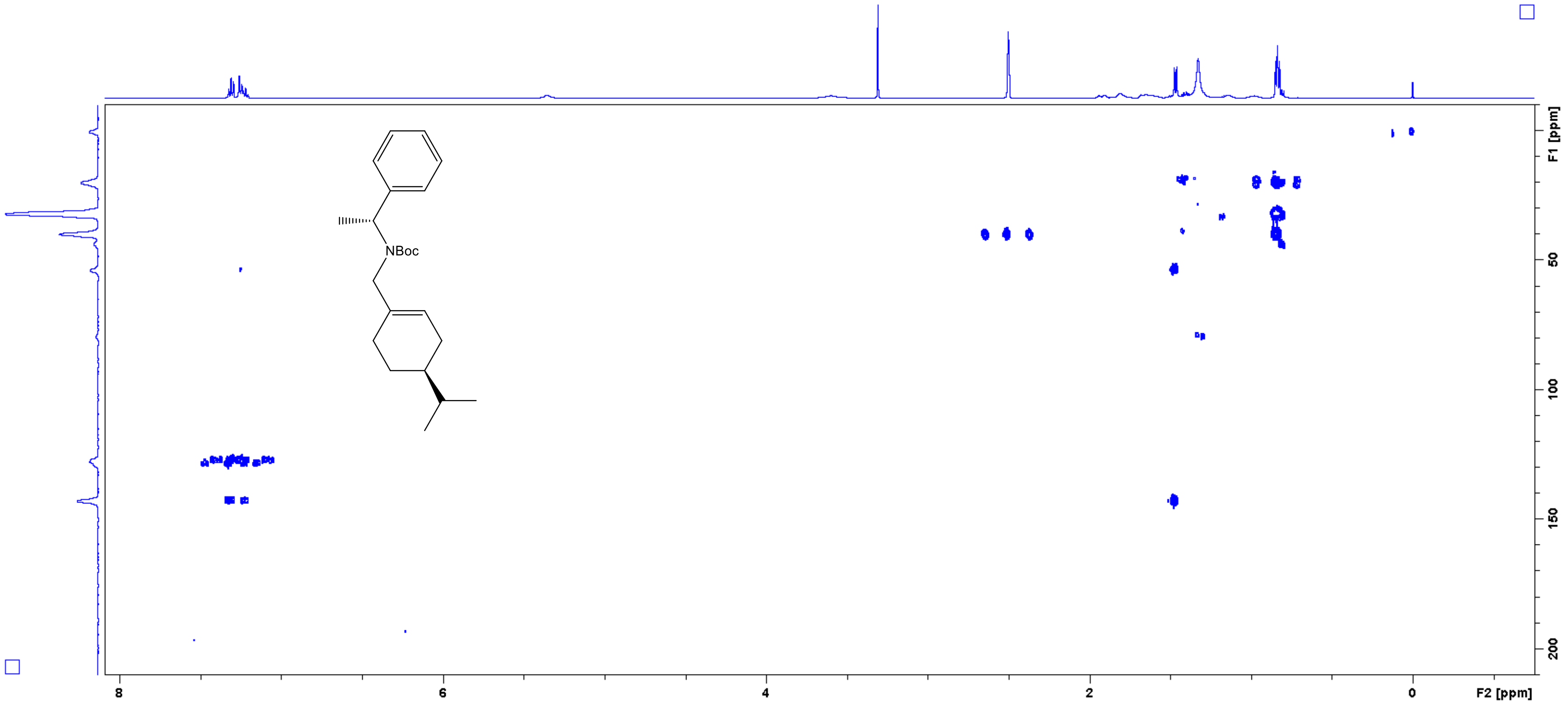

Figure S 26: ^{1}H-NMR of compound *tert*-butyl benzyl(((1*S*,2*S*,4*S*)-1,2-dihydroxy-4-isopropylcyclohexyl)methyl)carbamate **5a**

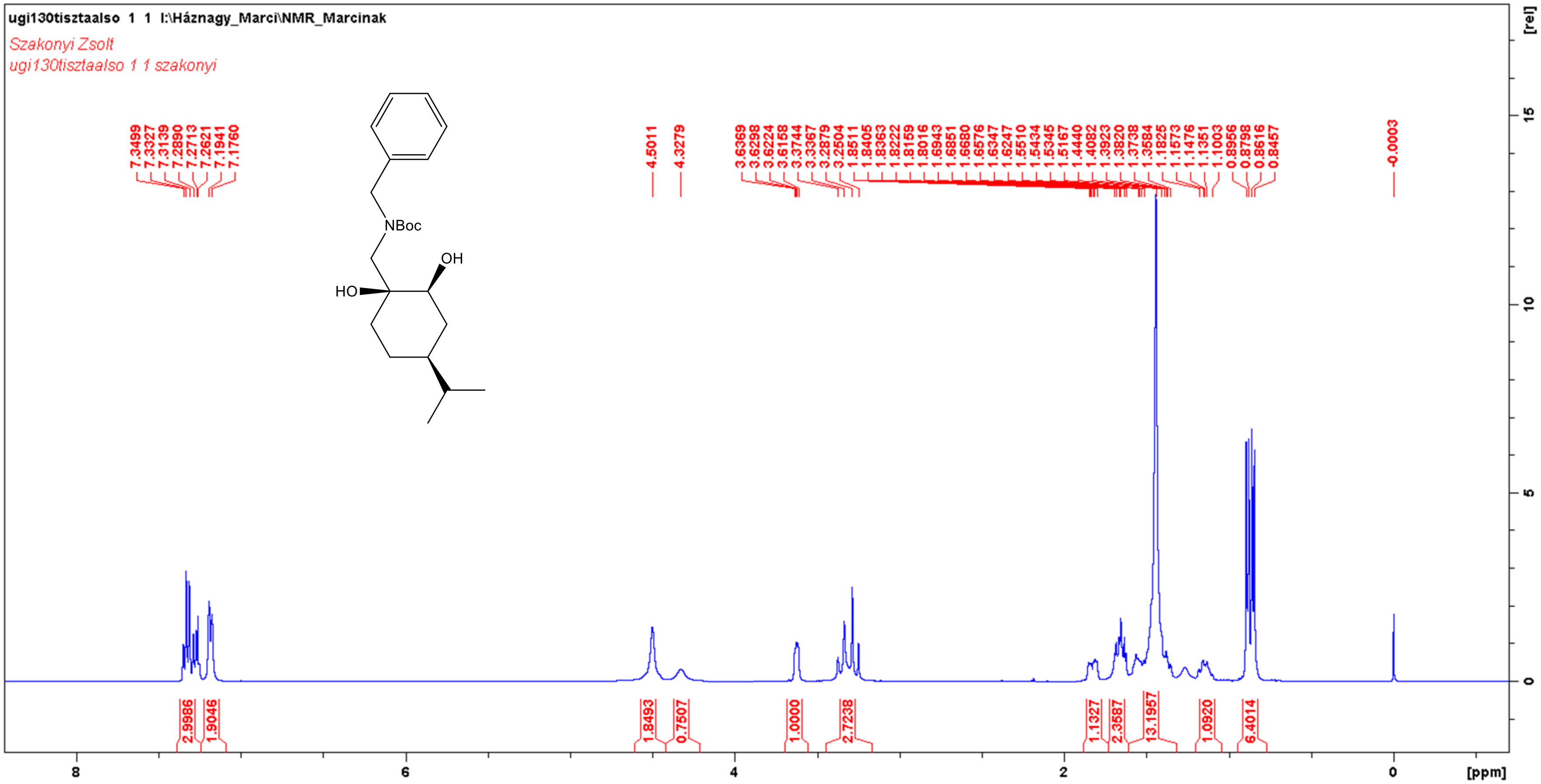

Figure S 27: ^{13}C-NMR of compound *tert*-butyl benzyl(((1*S*,2*S*,4*S*)-1,2-dihydroxy-4-isopropylcyclohexyl)methyl)carbamate **5a**

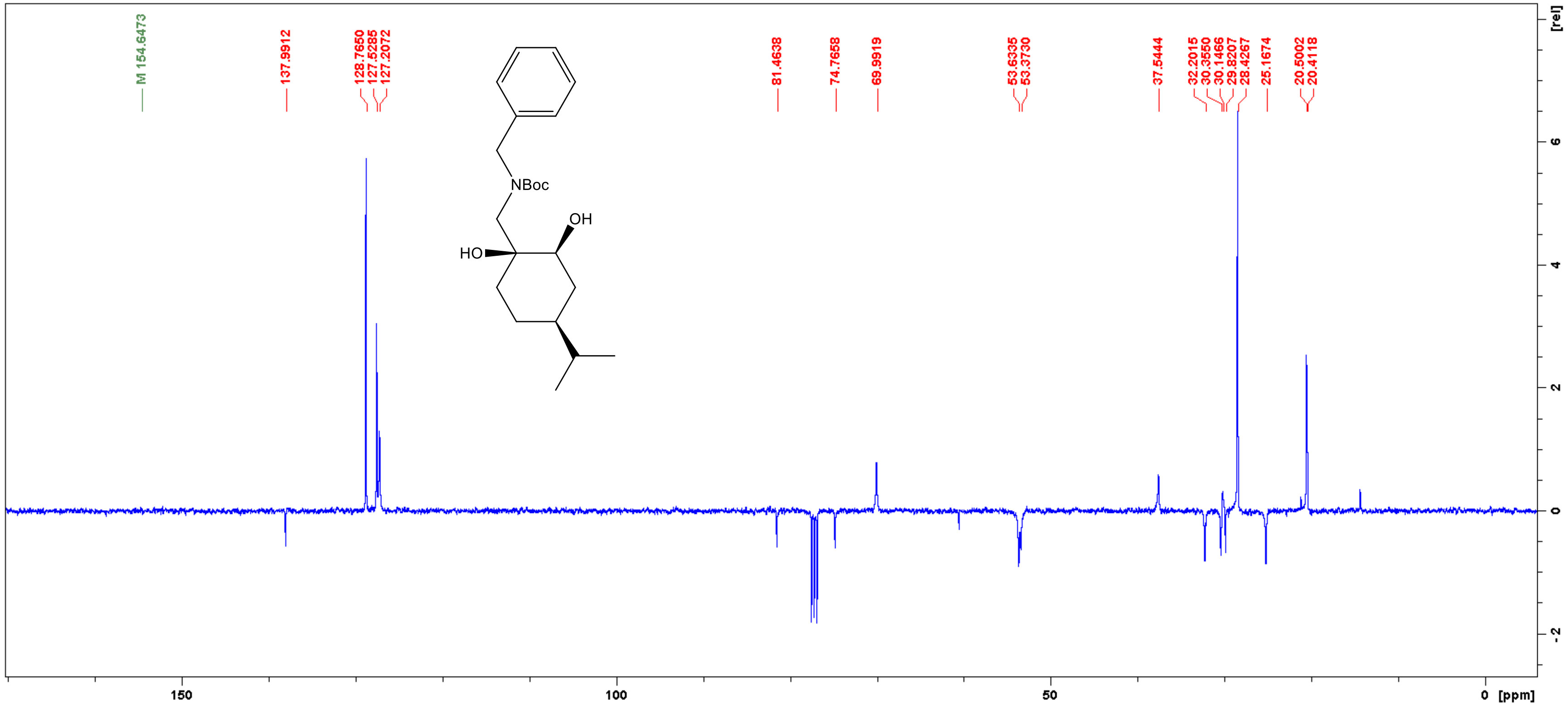

Figure S 28: ¹H-NMR of compound *tert*-butyl benzyl(((1*R*,2*R*,4*S*)-1,2-dihydroxy-4-isopropylcyclohexyl)methyl)carbamate **6a**

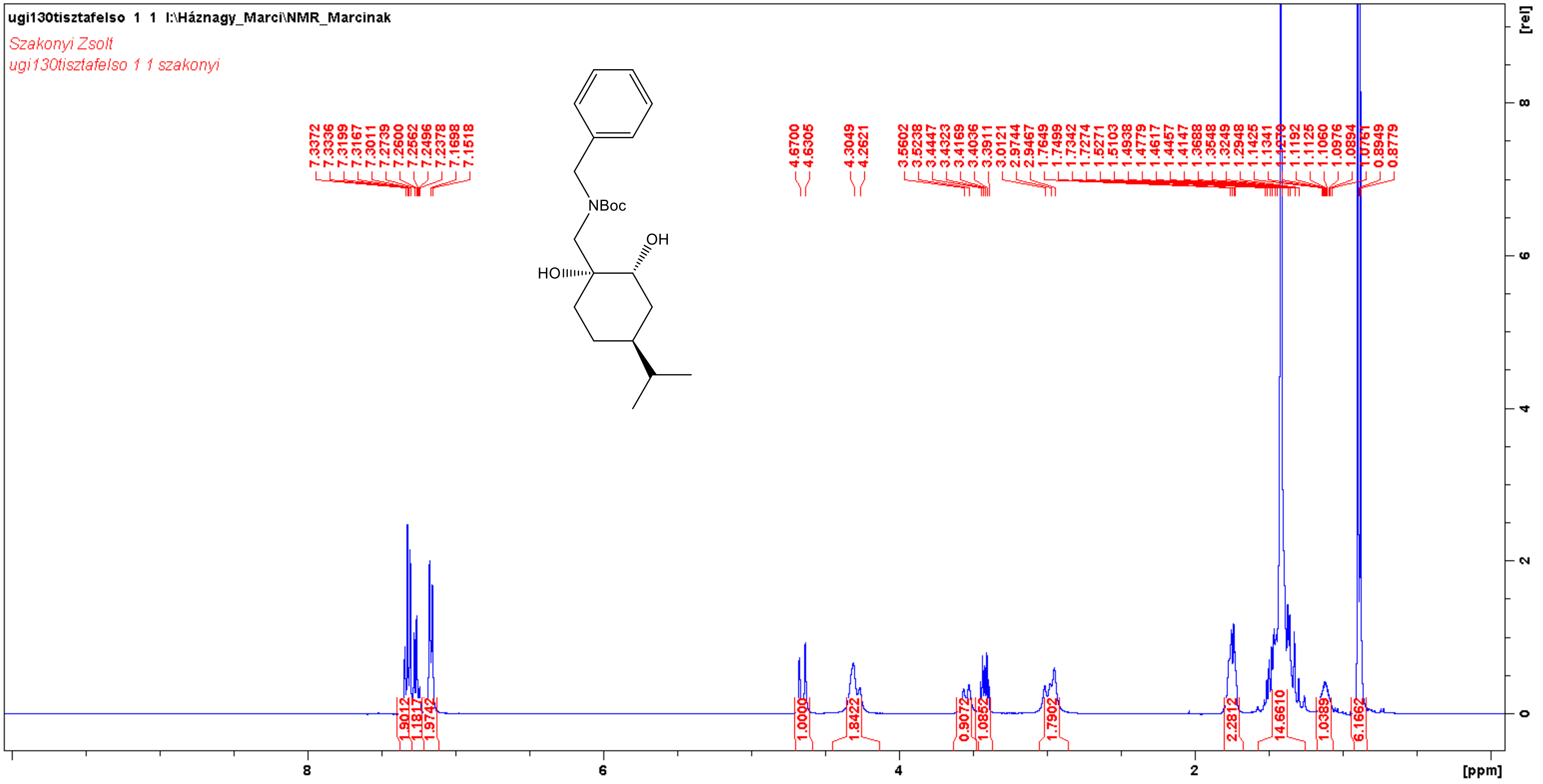

Figure S 29: ^{13}C-NMR of compound *tert*-butyl benzyl(((1*R*,2*R*,4*S*)-1,2-dihydroxy-4-isopropylcyclohexyl)methyl)carbamate **6a**

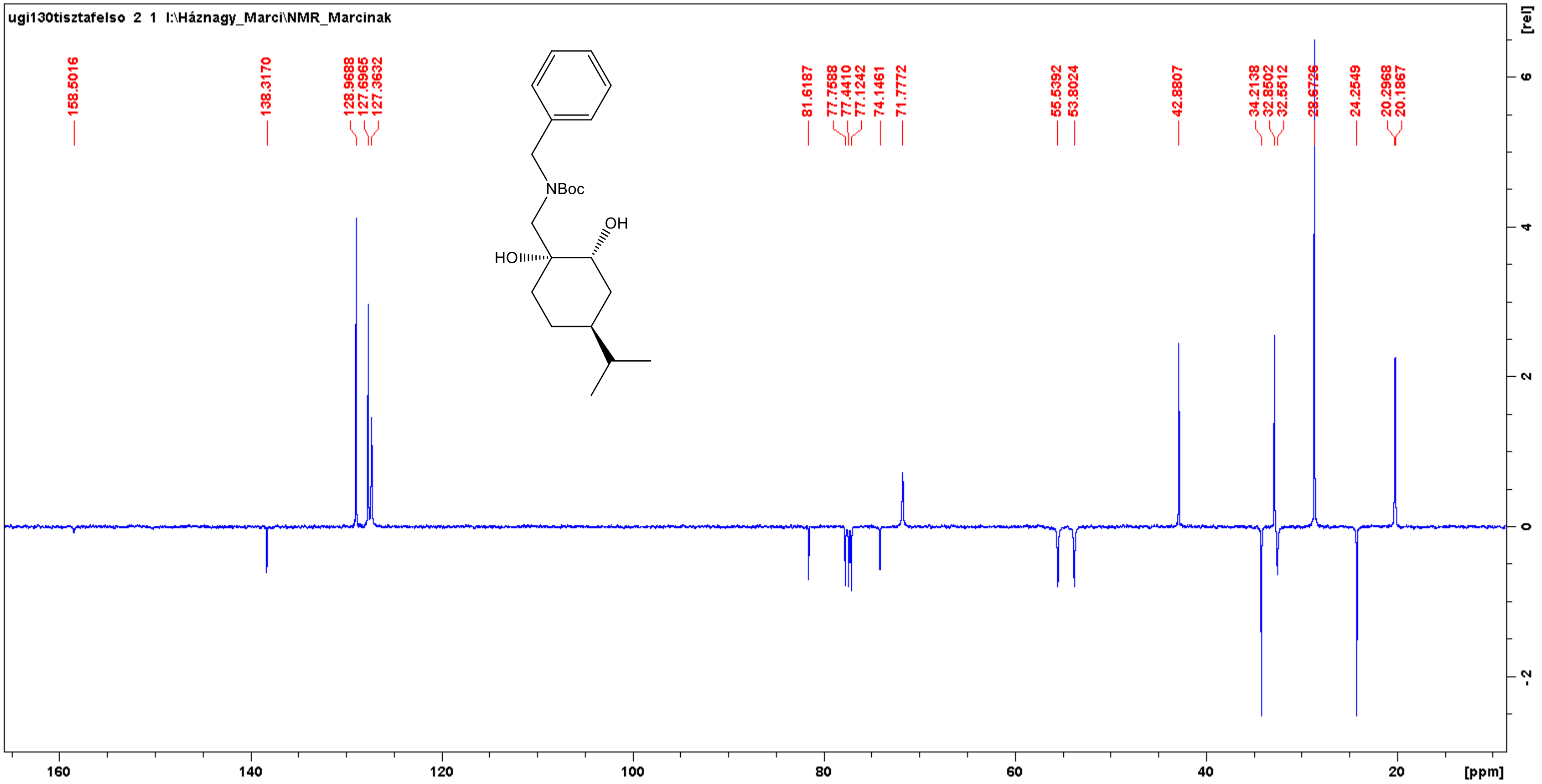

Figure S 30: ^{1}H-NMR of compound *tert*-butyl (((1*S*,2*S*,4*S*)-1,2-dihydroxy-4-isopropylcyclohexyl)methyl)((*S*)-1-phenylethyl)carbamate **5b**

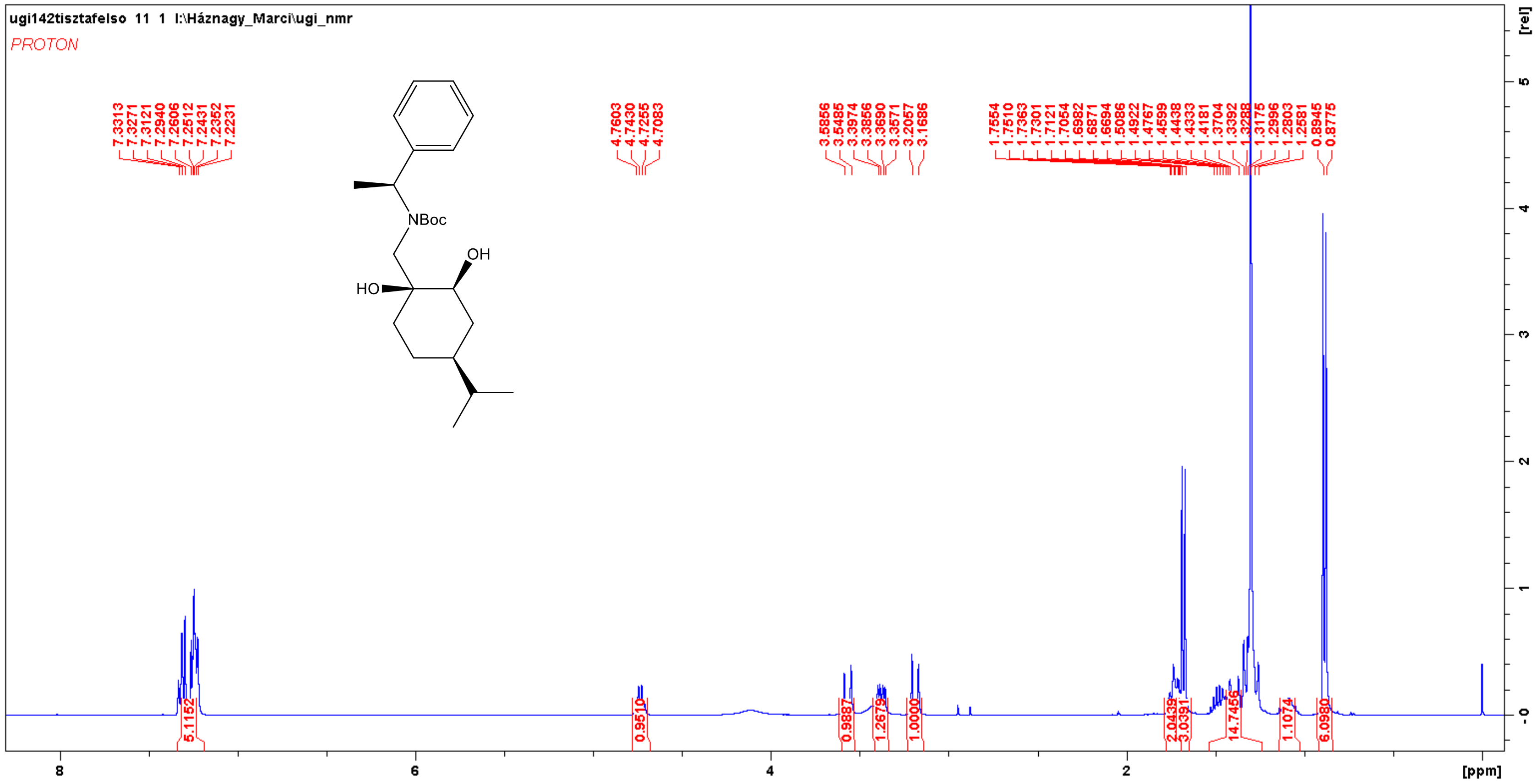

Figure S 31: ^{13}C-NMR of compound *tert*-butyl (((1*S*,2*S*,4*S*)-1,2-dihydroxy-4-isopropylcyclohexyl)methyl)((*S*)-1-phenylethyl)carbamate **5b**

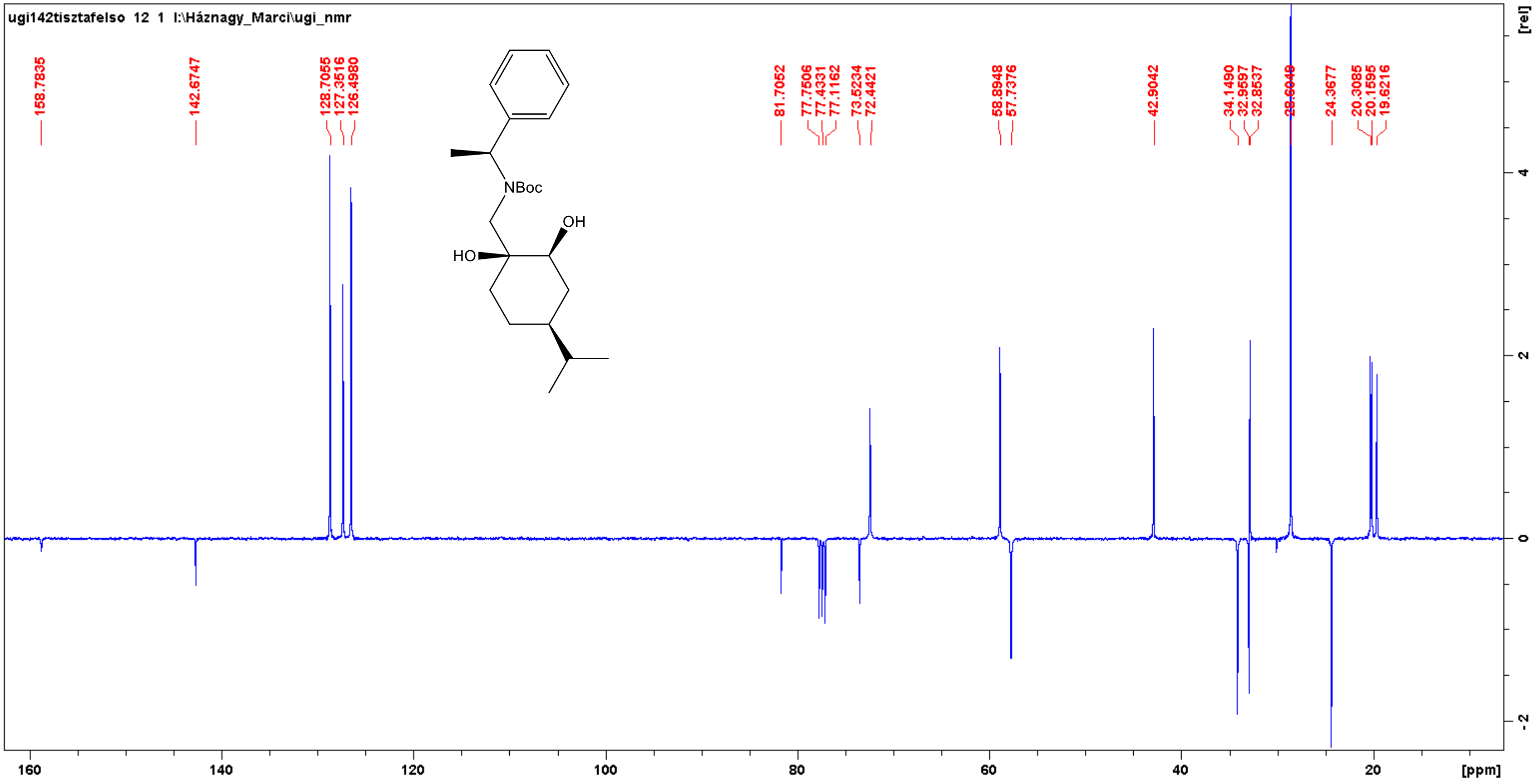

Figure S 32: ¹H-NMR of compound *tert*-butyl (((1*R*,2*R*,4*S*)-1,2-dihydroxy-4-isopropylcyclohexyl)methyl)((*S*)-1-phenylethyl)carbamate **6b**

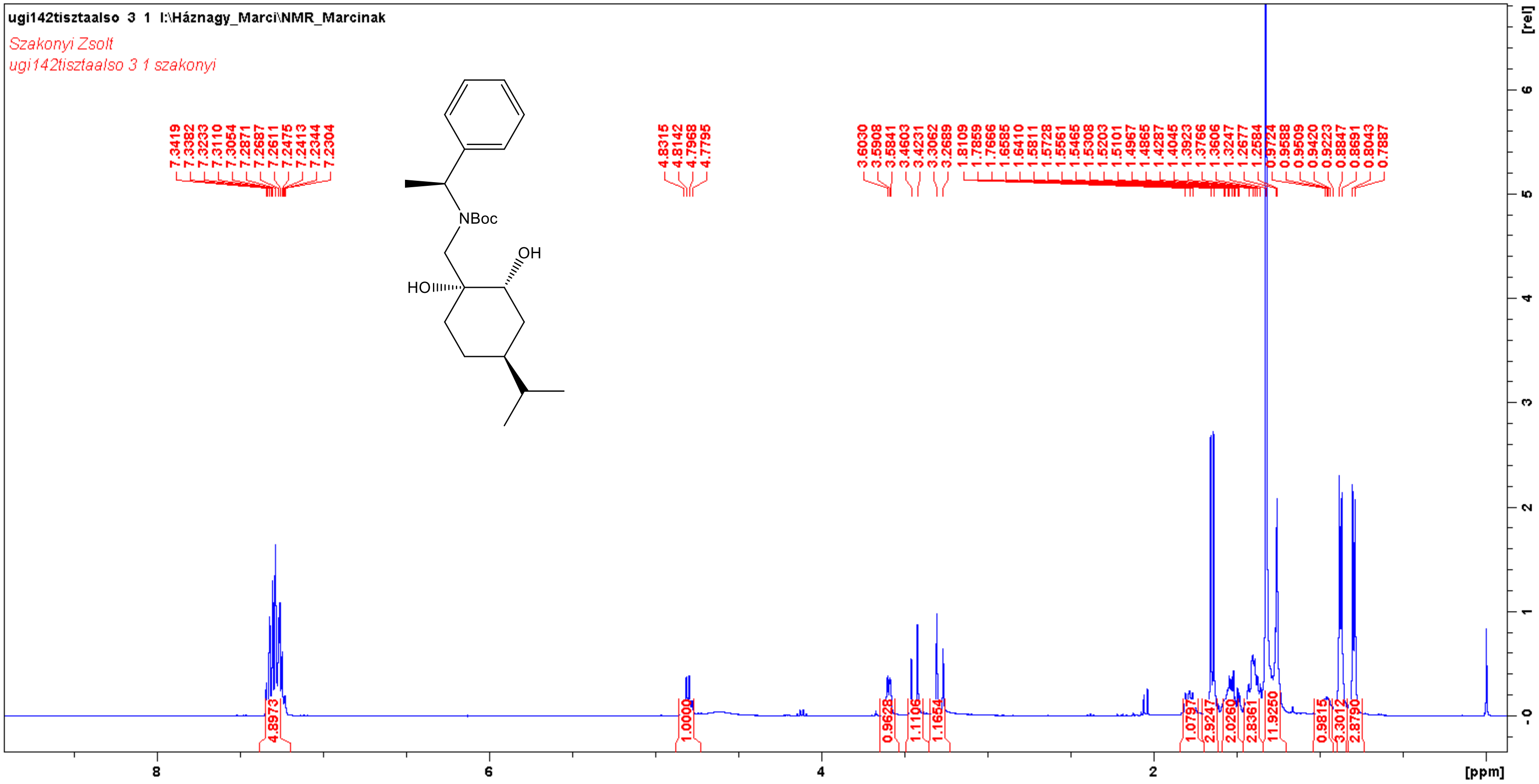

Figure S 33: ^{13}C-NMR of compound *tert*-butyl (((1*R*,2*R*,4*S*)-1,2-dihydroxy-4-isopropylcyclohexyl)methyl)((*S*)-1-phenylethyl)carbamate **6b**

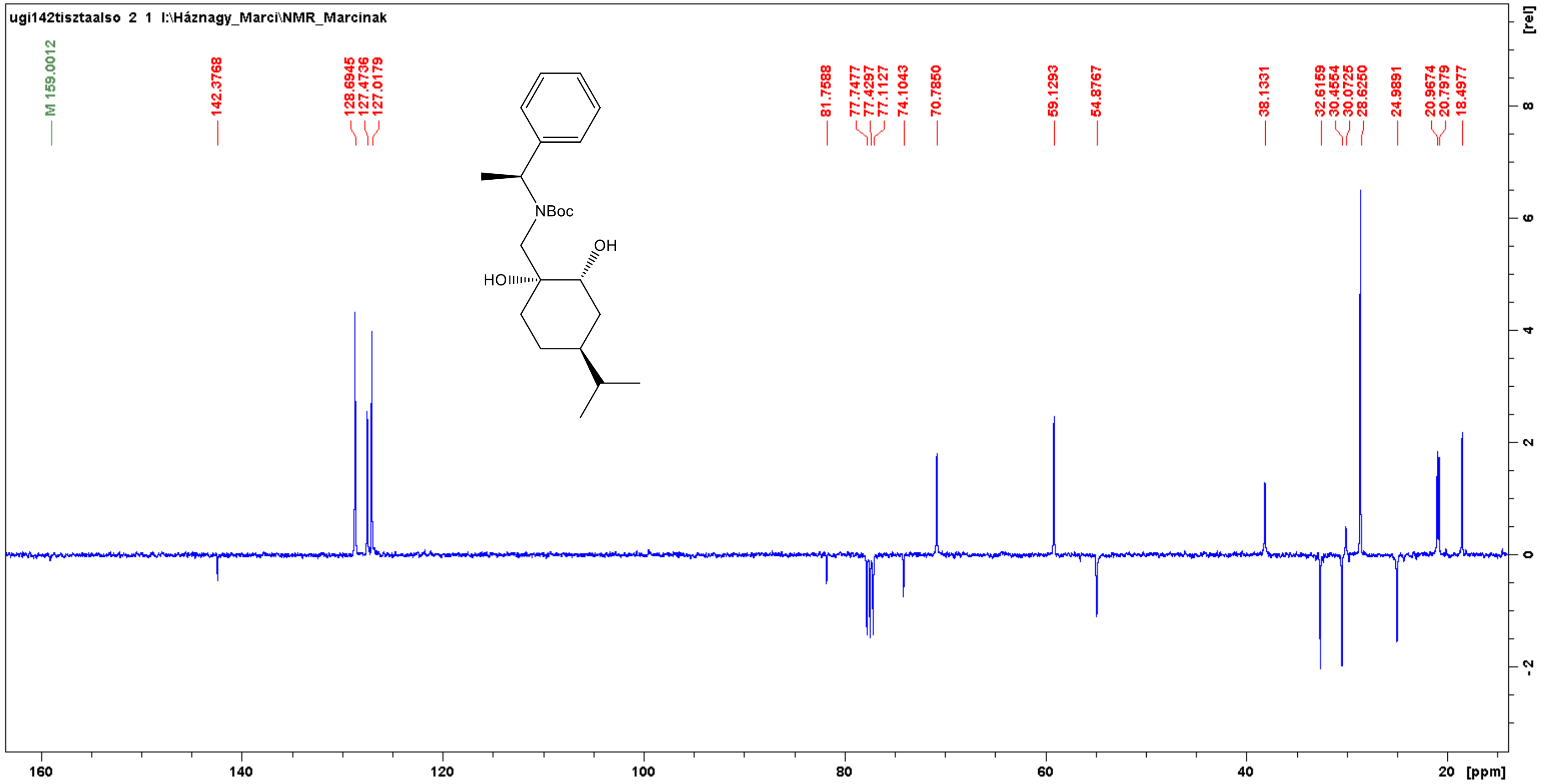

Figure S 34: ^{1}H-NMR of compound *tert*-butyl (((1*S*,2*S*,4*S*)-1,2-dihydroxy-4-isopropylcyclohexyl)methyl)((*R*)-1-phenylethyl)carbamate **5c**

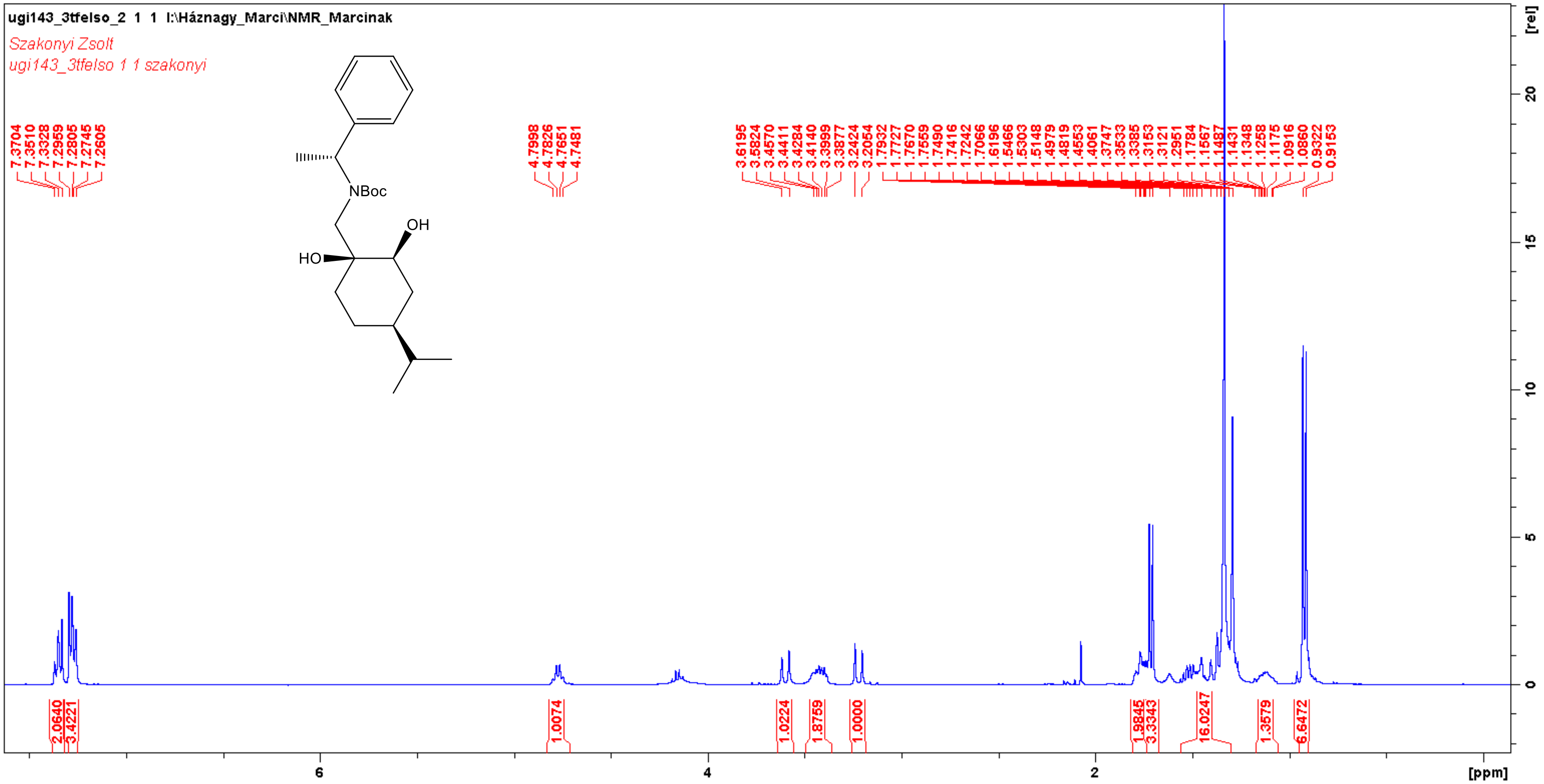

Figure S 35: ^{13}C-NMR of compound *tert*-butyl (((1*S*,2*S*,4*S*)-1,2-dihydroxy-4-isopropylcyclohexyl)methyl)((*R*)-1-phenylethyl)carbamate **5c**

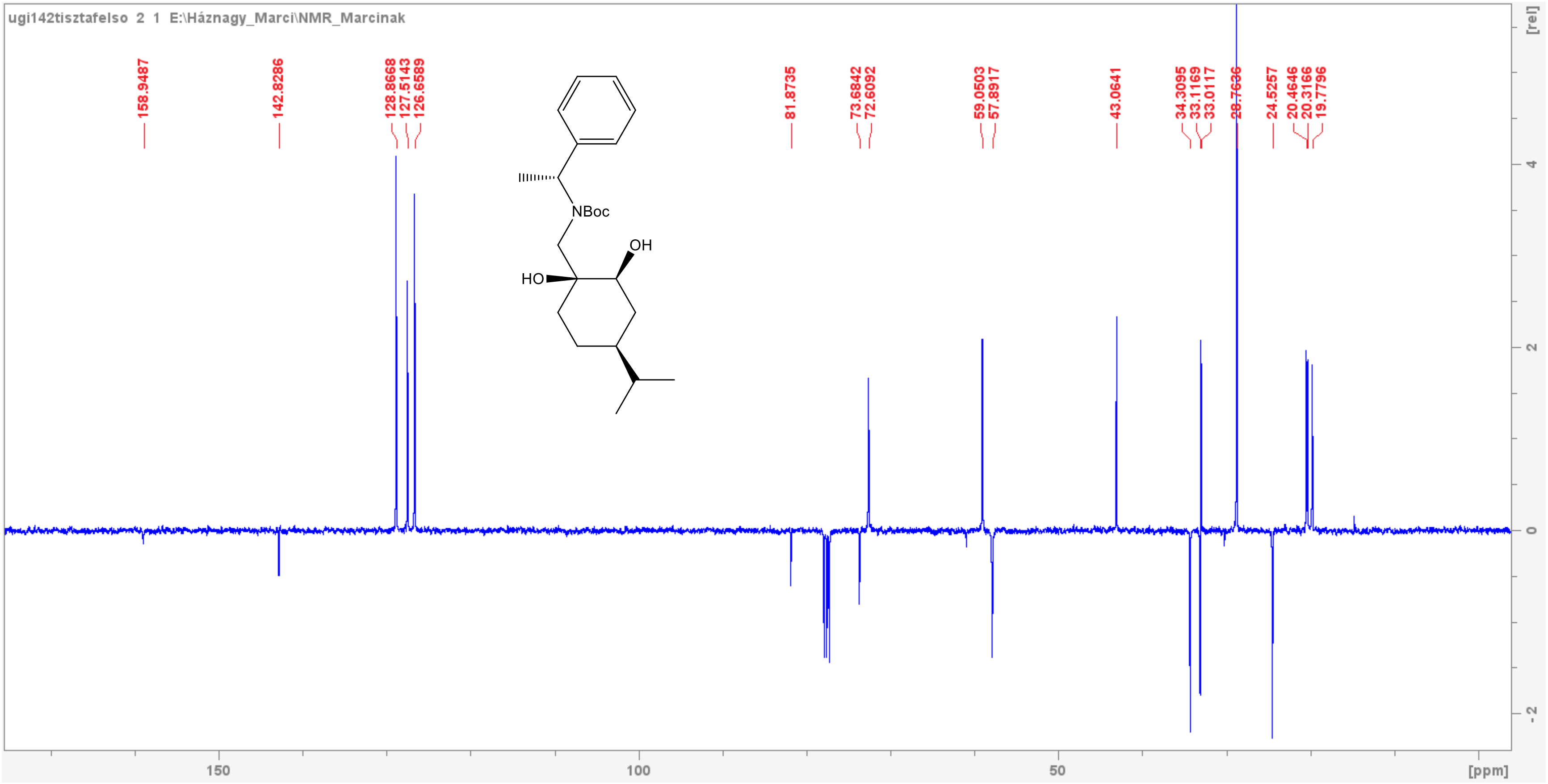

Figure S 36: ^{1}H-NMR of compound *tert*-butyl (((1*R*,2*R*,4*S*)-1,2-dihydroxy-4-isopropylcyclohexyl)methyl)((*R*)-1-phenylethyl)carbamate **6c**

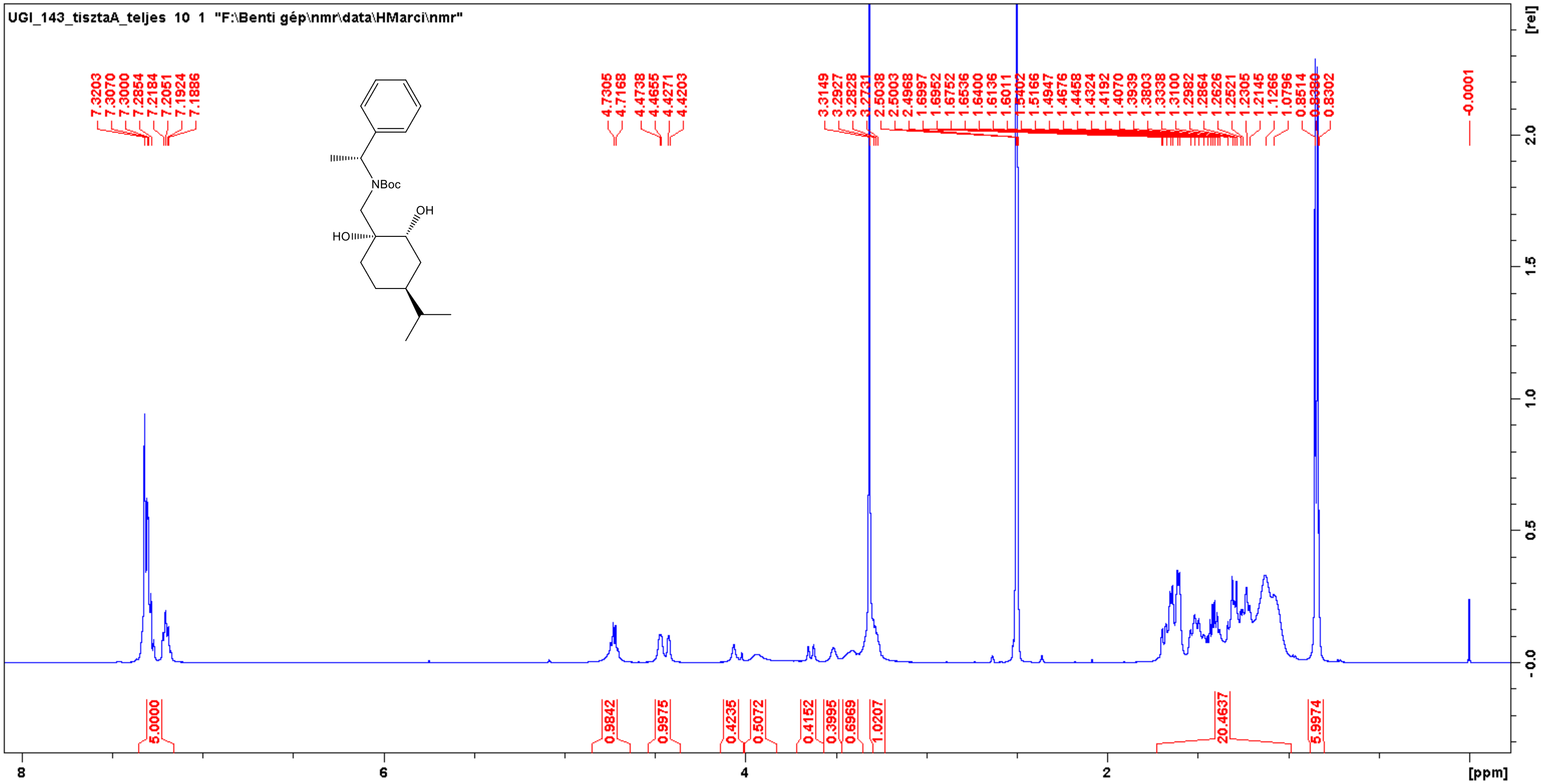

Figure S 37: ^{13}C-NMR of compound *tert*-butyl (((1*R*,2*R*,4*S*)-1,2-dihydroxy-4-isopropylcyclohexyl)methyl)((*R*)-1-phenylethyl)carbamate **6c**

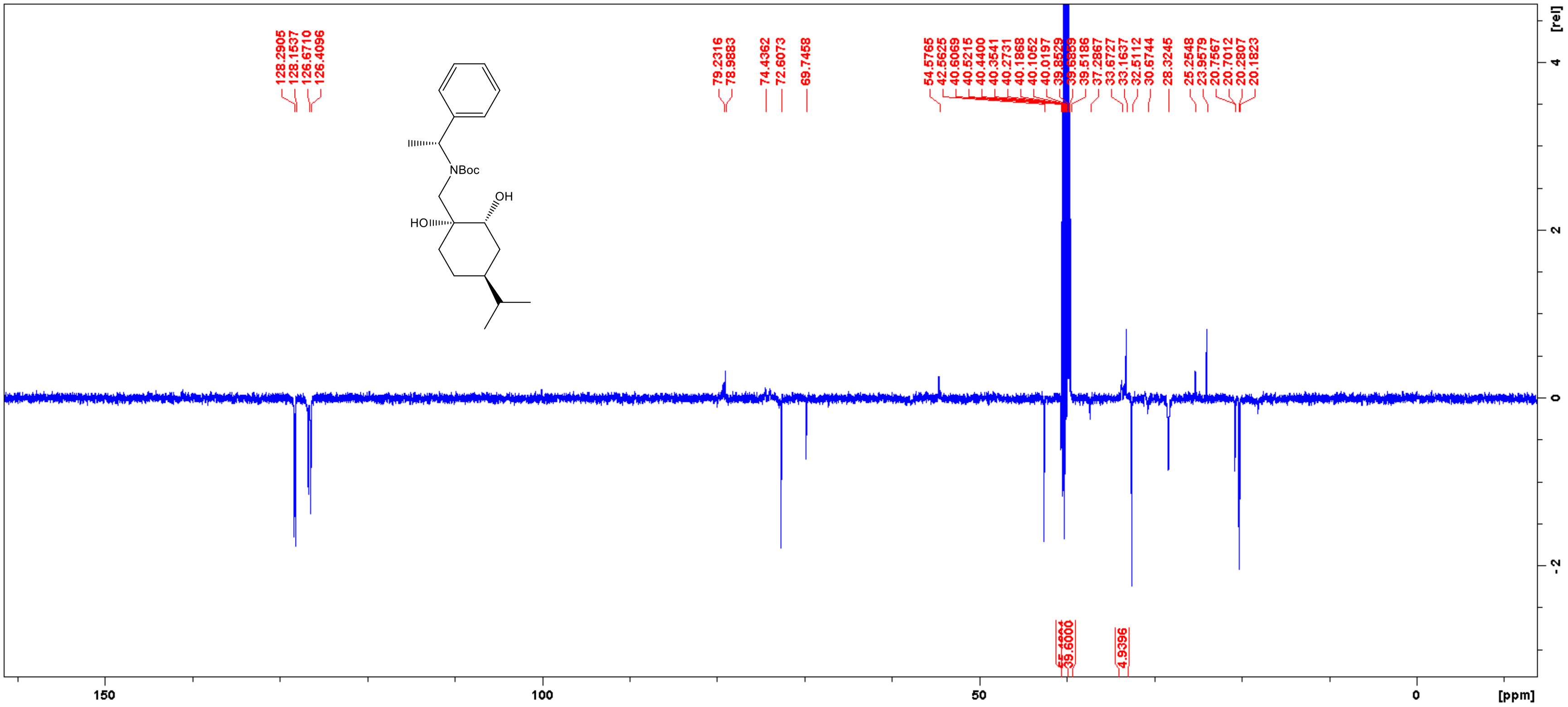

Figure S 38: HSQC NMR of compound *tert*-butyl (((1*R*,2*R*,4*S*)-1,2-dihydroxy-4-isopropylcyclohexyl)methyl)((*R*)-1-phenylethyl)carbamate **6c**

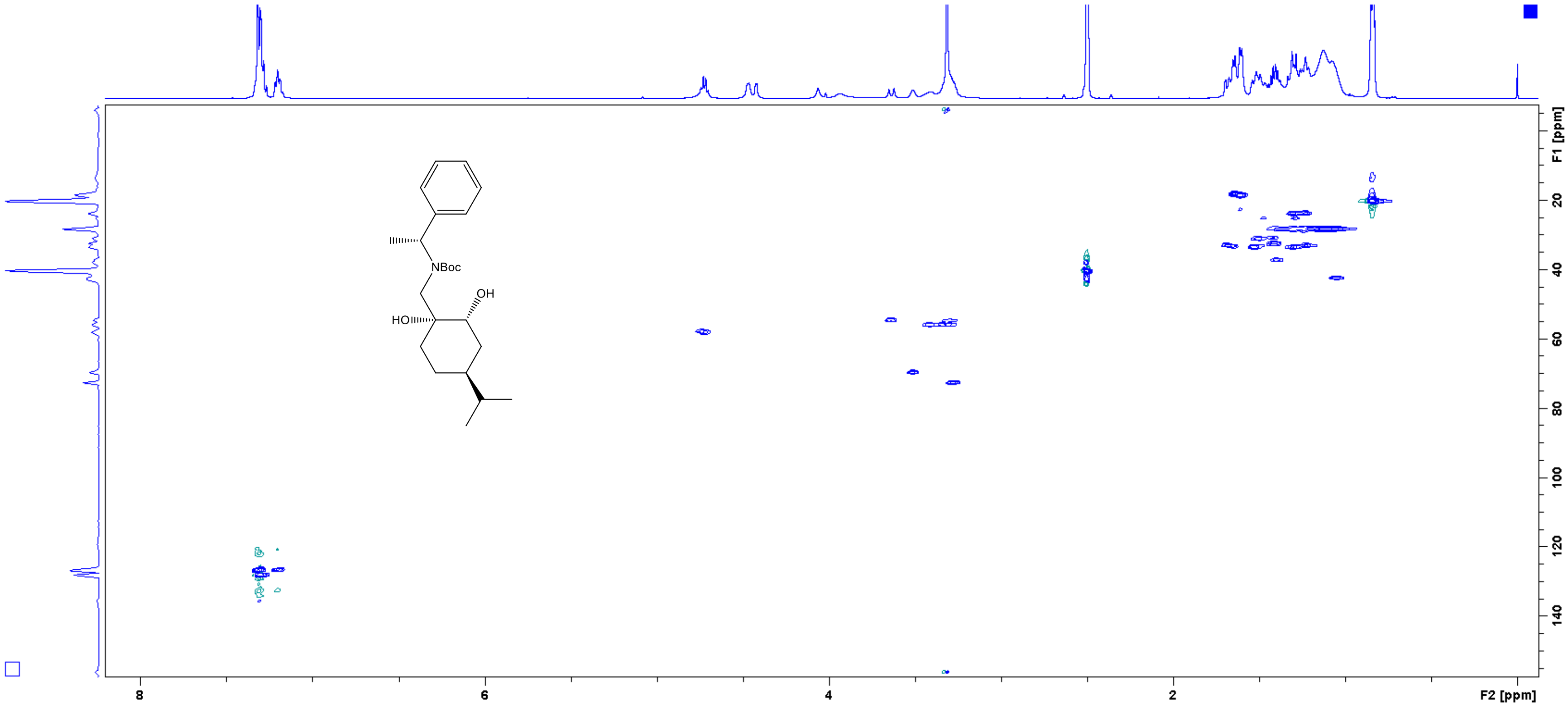

Figure S 39: HMBC NMR of compound *tert*-butyl (((1*R*,2*R*,4*S*)-1,2-dihydroxy-4-isopropylcyclohexyl)methyl)((*R*)-1-phenylethyl)carbamate **6c**

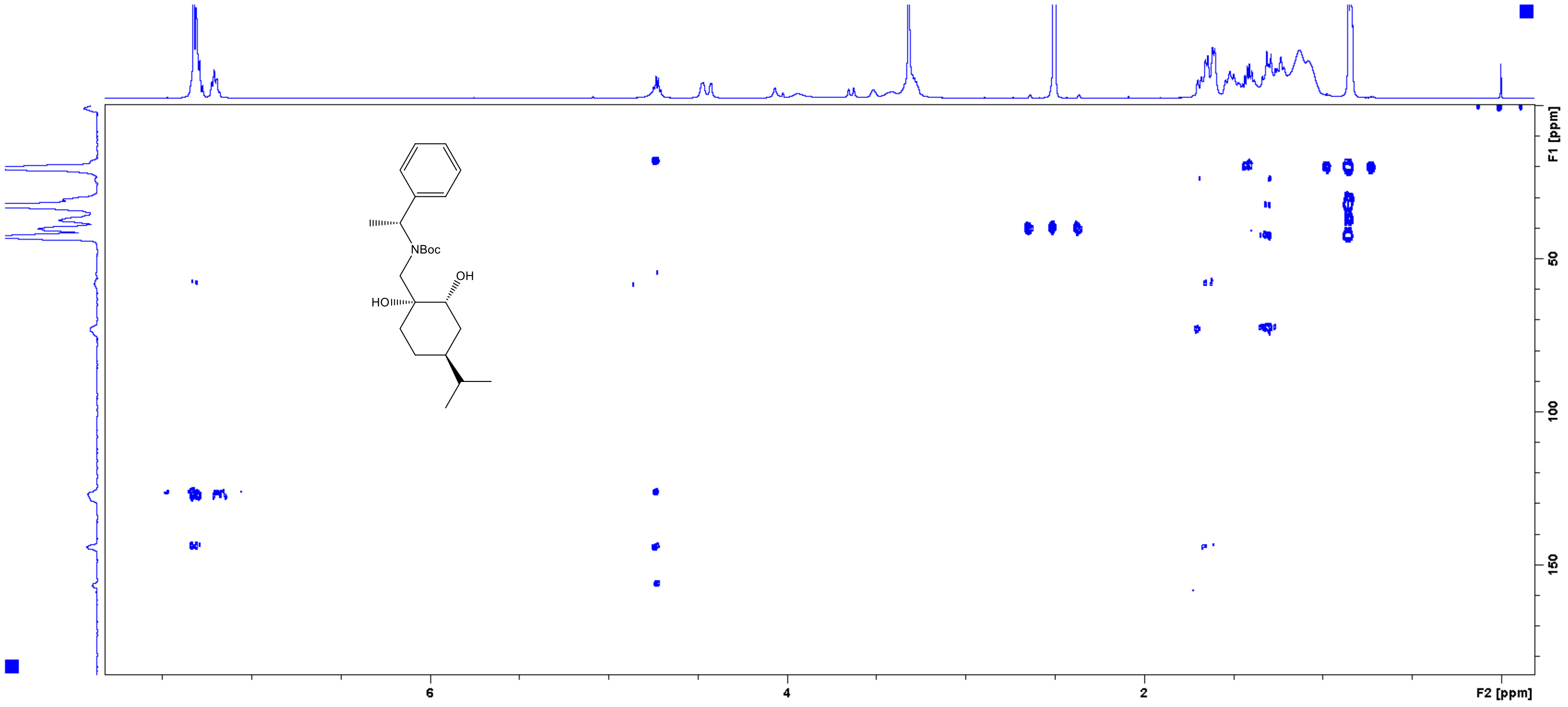

Figure S 40: ^{1}H-NMR of compound (1*S*,2*S*,4*S*)-1-((benzylamino)methyl)-4-isopropylcyclohexane-1,2-diol **7a**

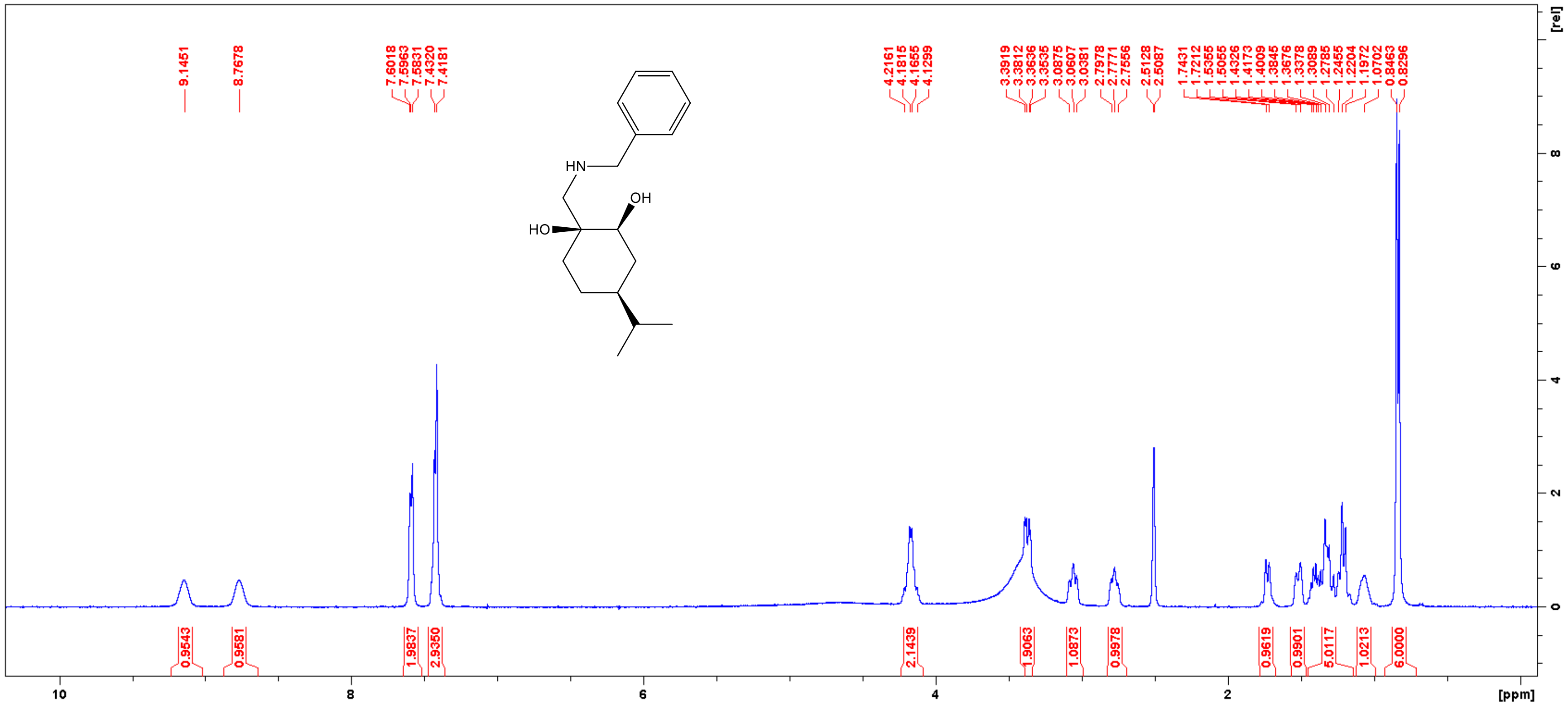

Figure S 41: **^{13}C-NMR of compound** (1*S*,2*S*,4*S*)-1-((benzylamino)methyl)-4-isopropylcyclohexane-1,2-diol **7a**

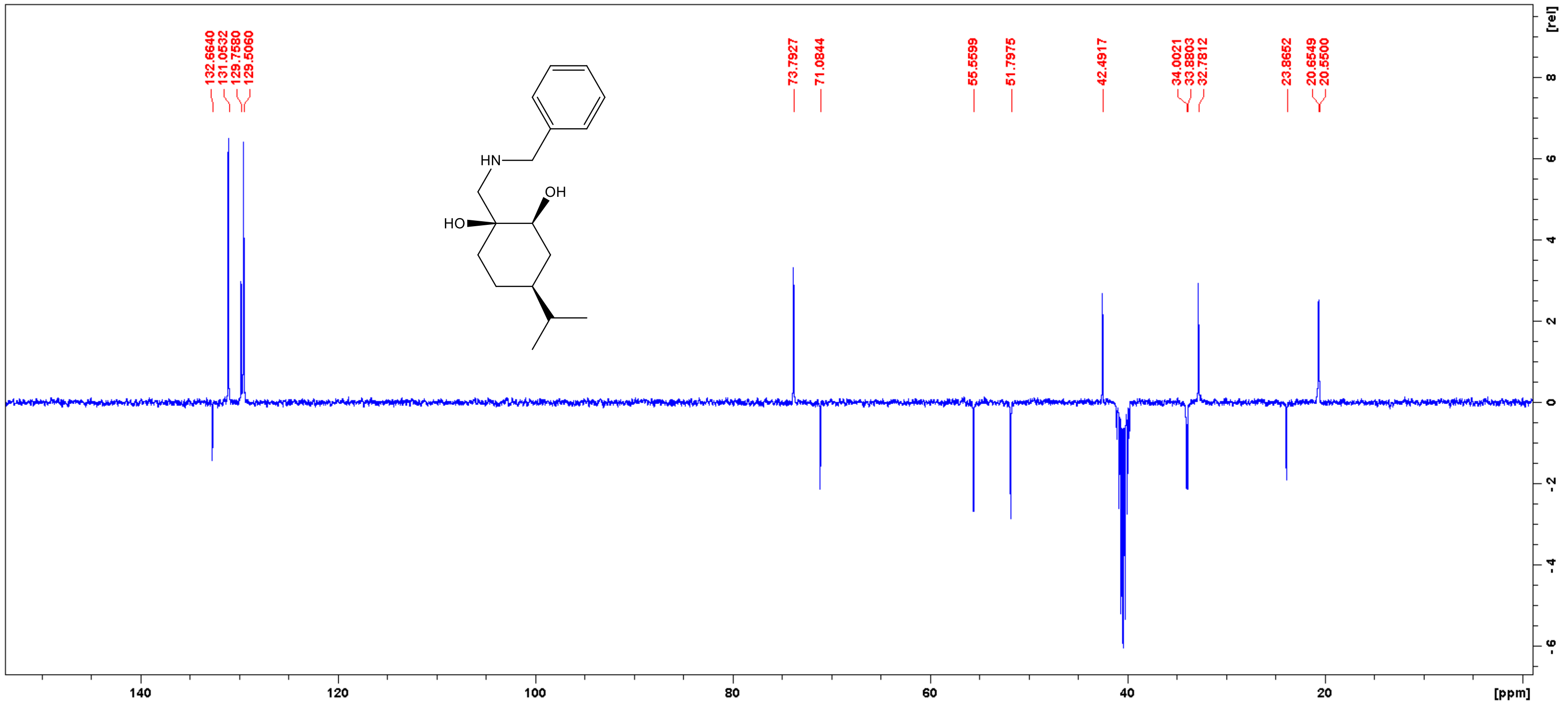

Figure S 42: COSY NMR of compound (1*S*,2*S*,4*S*)-1-((benzylamino)methyl)-4-isopropylcyclohexane-1,2-diol hydrochloride **7a**

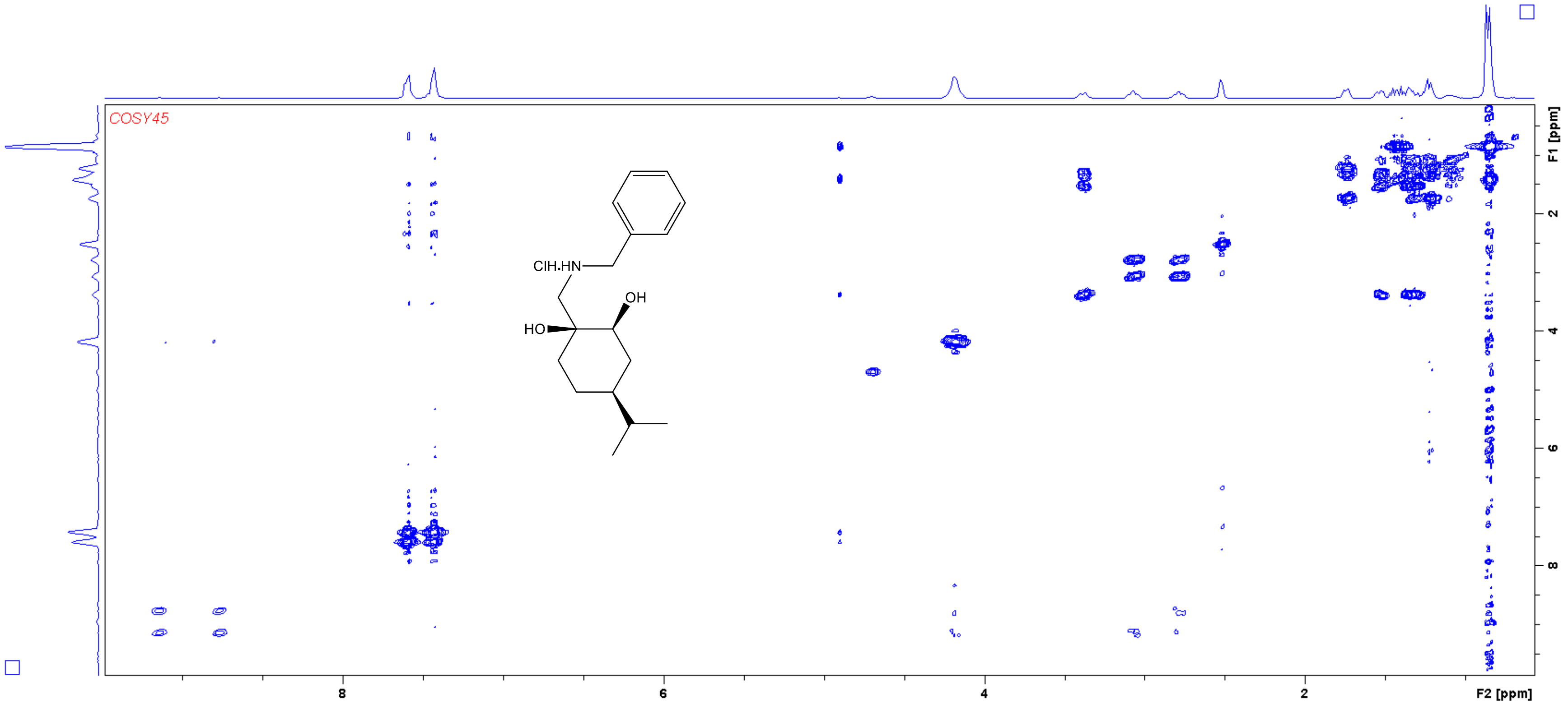

Figure S 43: NOESY NMR of compound (1*S*,2*S*,4*S*)-1-((benzylamino)methyl)-4-isopropylcyclohexane-1,2-diol hydrochloride **7a**

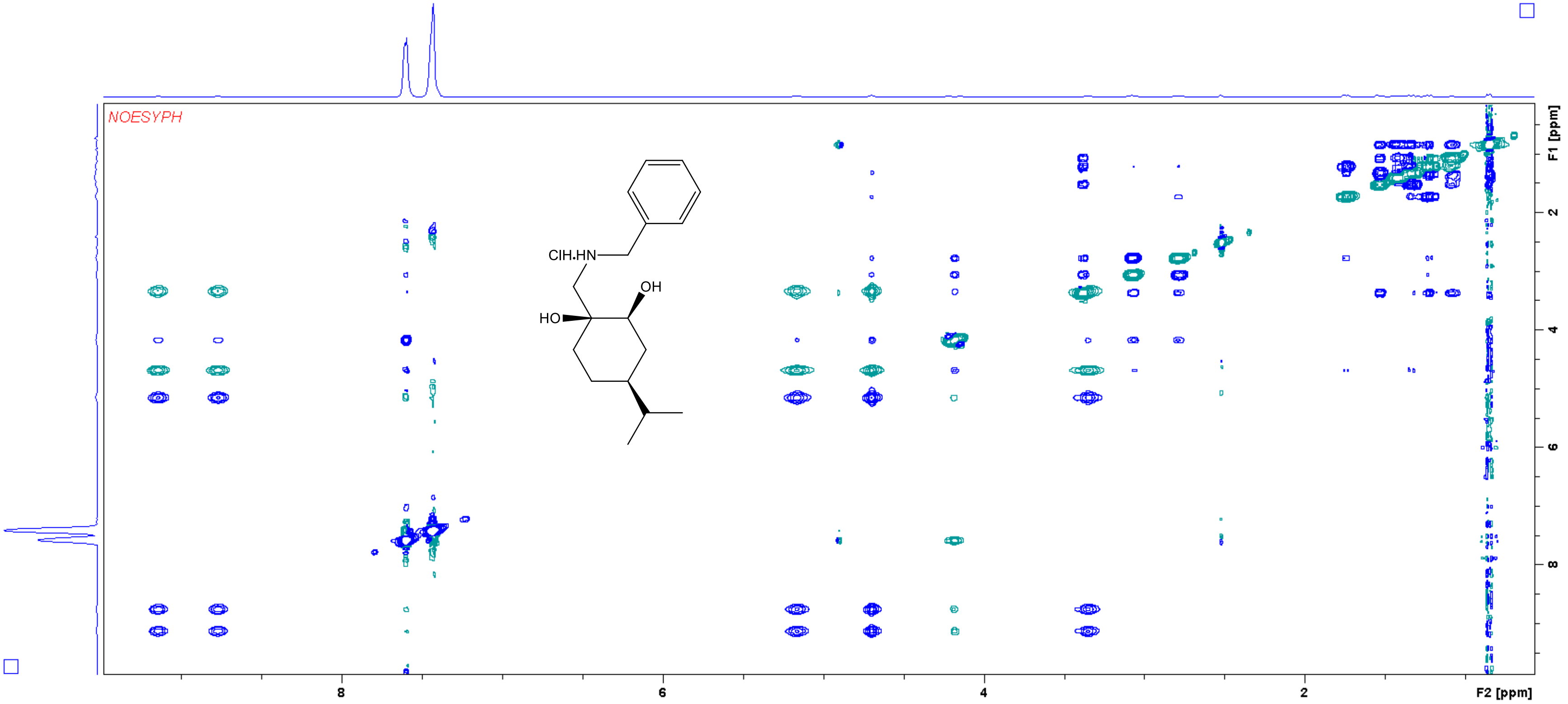

Figure S 44: HSQC NMR of compound (1*S*,2*S*,4*S*)-1-((benzylamino)methyl)-4-isopropylcyclohexane-1,2-diol hydrochloride **7a**

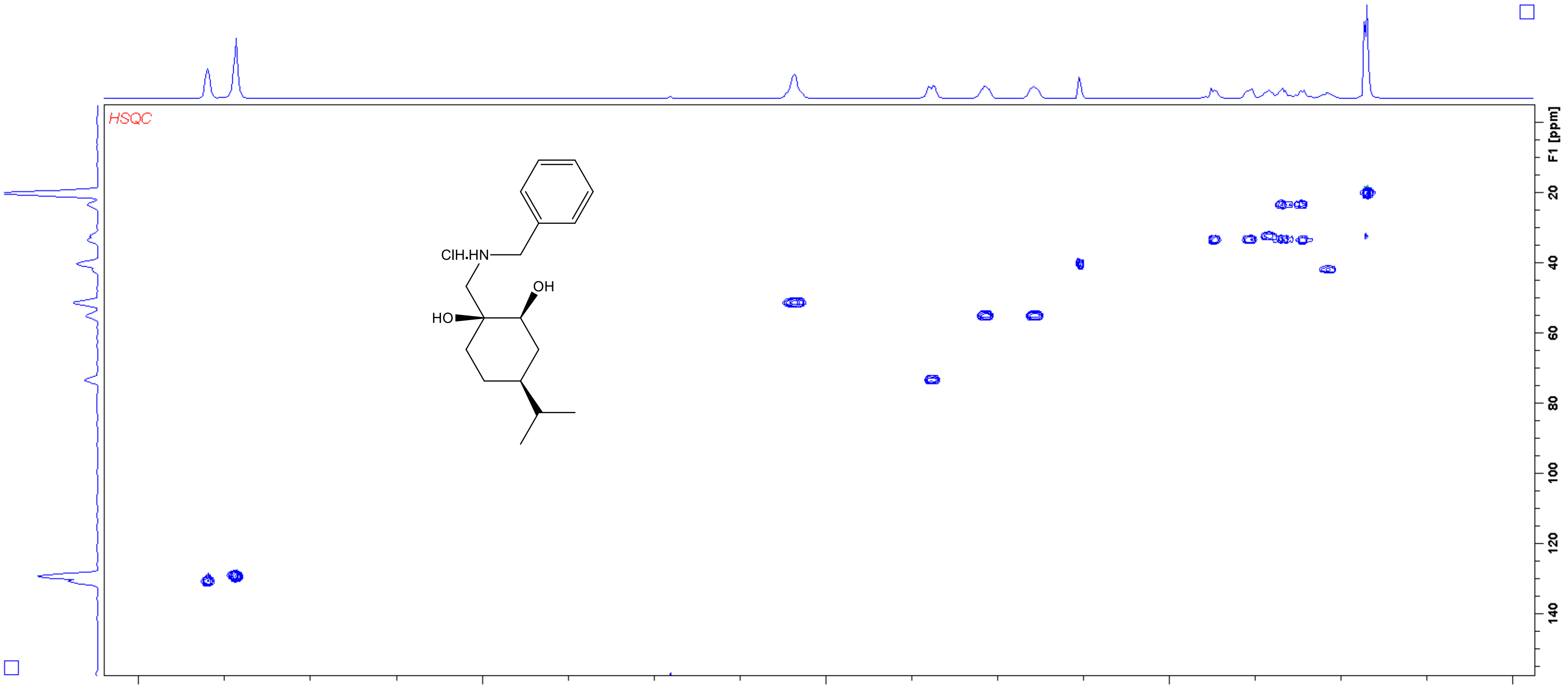

Figure S 45: ^{1}H-NMR of compound (1*S*,2*S*,4*S*)-4-isopropyl-1-((((*S*)-1-phenylethyl)amino)methyl)cyclohexane-1,2-diol hydrochloride **7b**

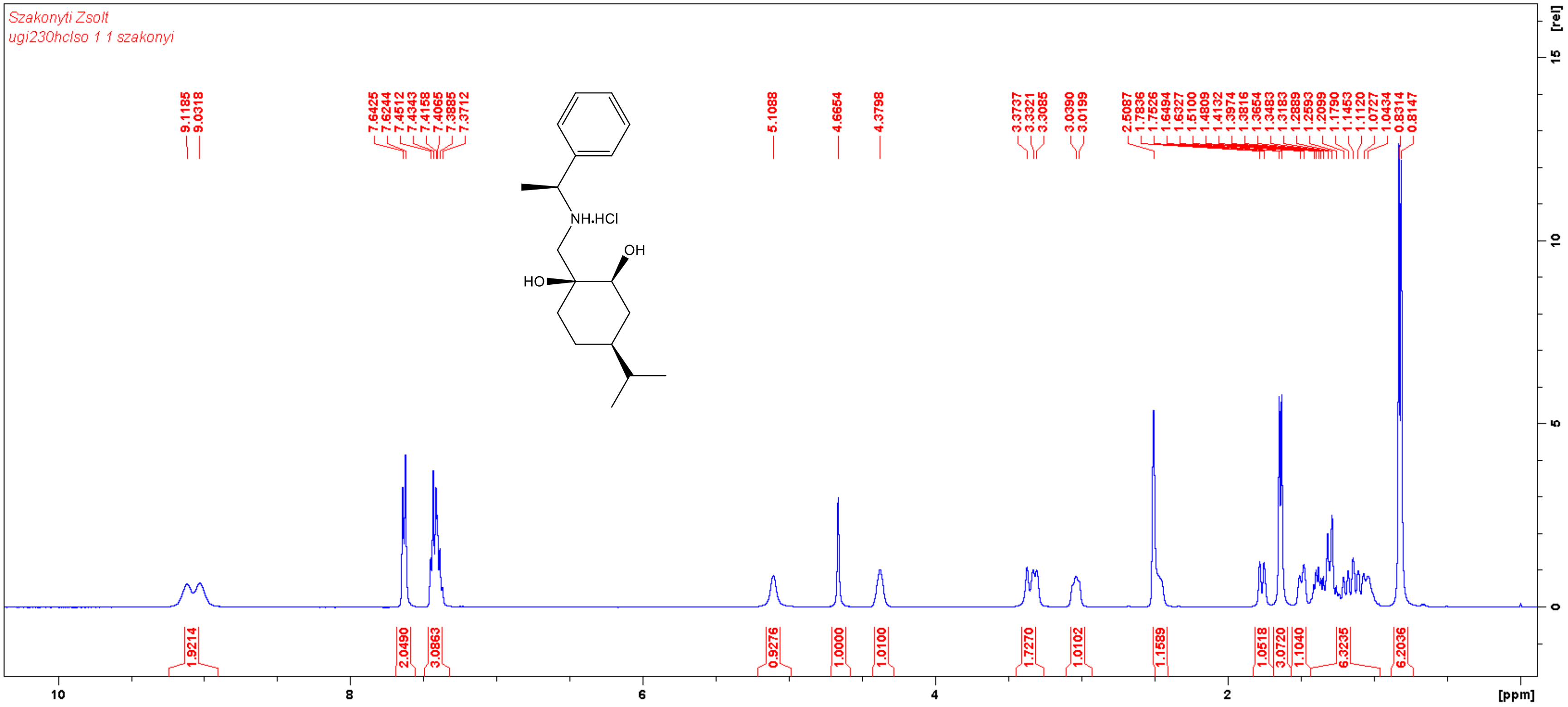

Figure S 46: ^{13}C-NMR of compound (1*S*,2*S*,4*S*)-4-isopropyl-1-((((*S*)-1-phenylethyl)amino)methyl)cyclohexane-1,2-diol hydrochloride **7b**

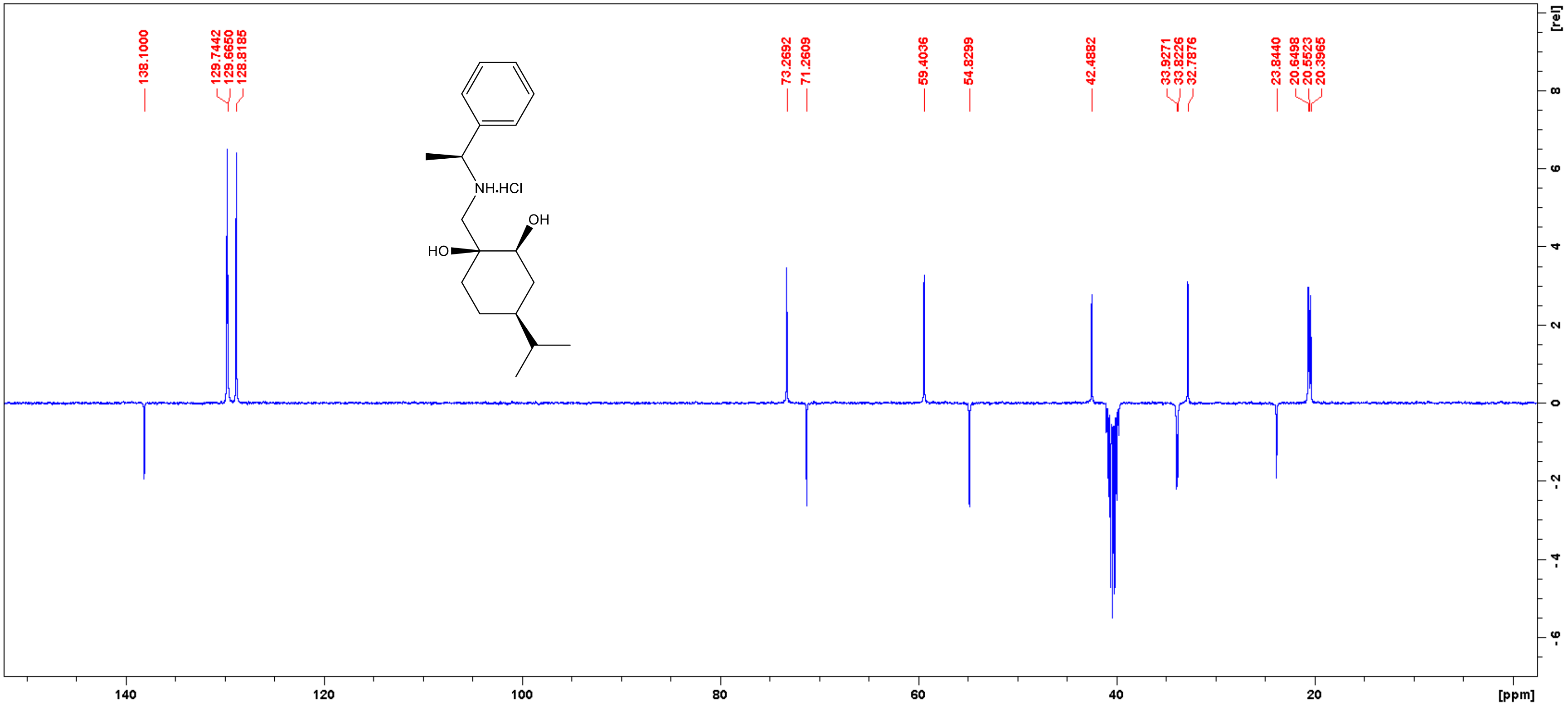

Figure S 47: ^{1}H-NMR of compound (1*S*,2*S*,4*S*)-4-Isopropyl-1-((((*R*)-1-phenylethyl)amino)methyl)cyclohexane-1,2-diol **7c**

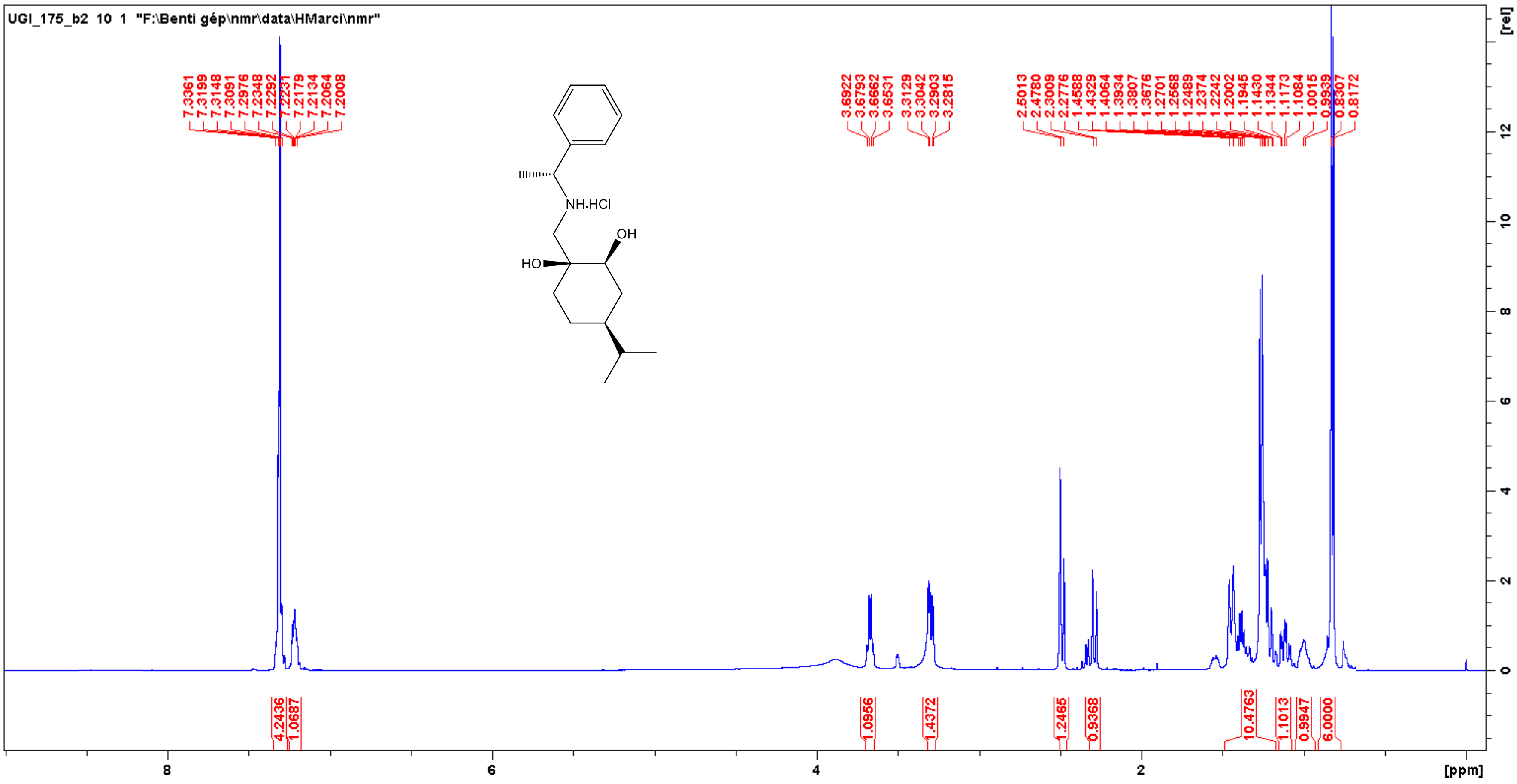

Figure S 48: ^{13}C-NMR of compound (1*S*,2*S*,4*S*)-4-Isopropyl-1-((((*R*)-1-phenylethyl)amino)methyl)cyclohexane-1,2-diol **7c**

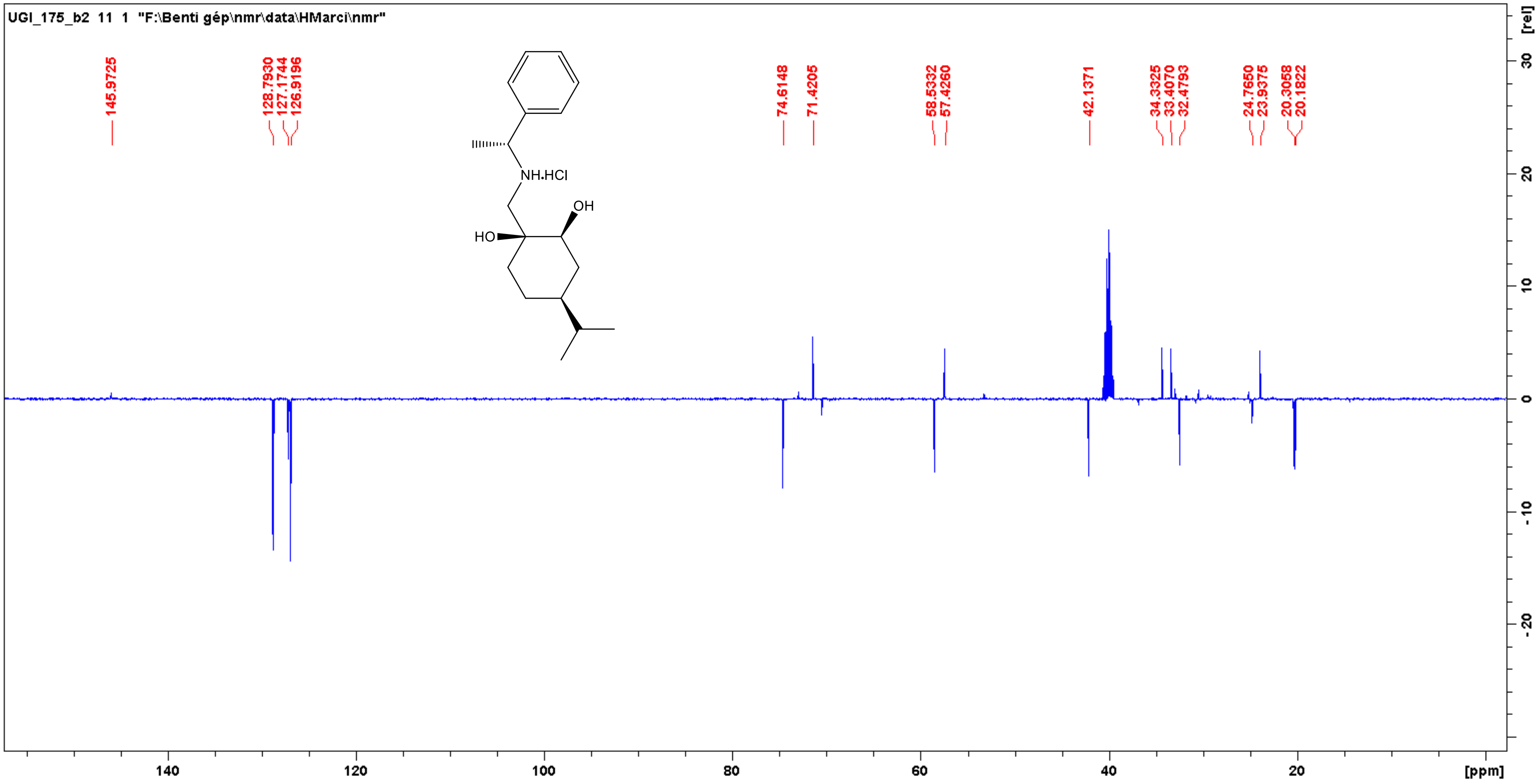

Figure S 49: ^{1}H-NMR of compound (1*R*,2*R*,4*S*)-1-((Benzylamino)methyl)-4-isopropylcyclohexane-1,2-diol hydrochloride **11a**

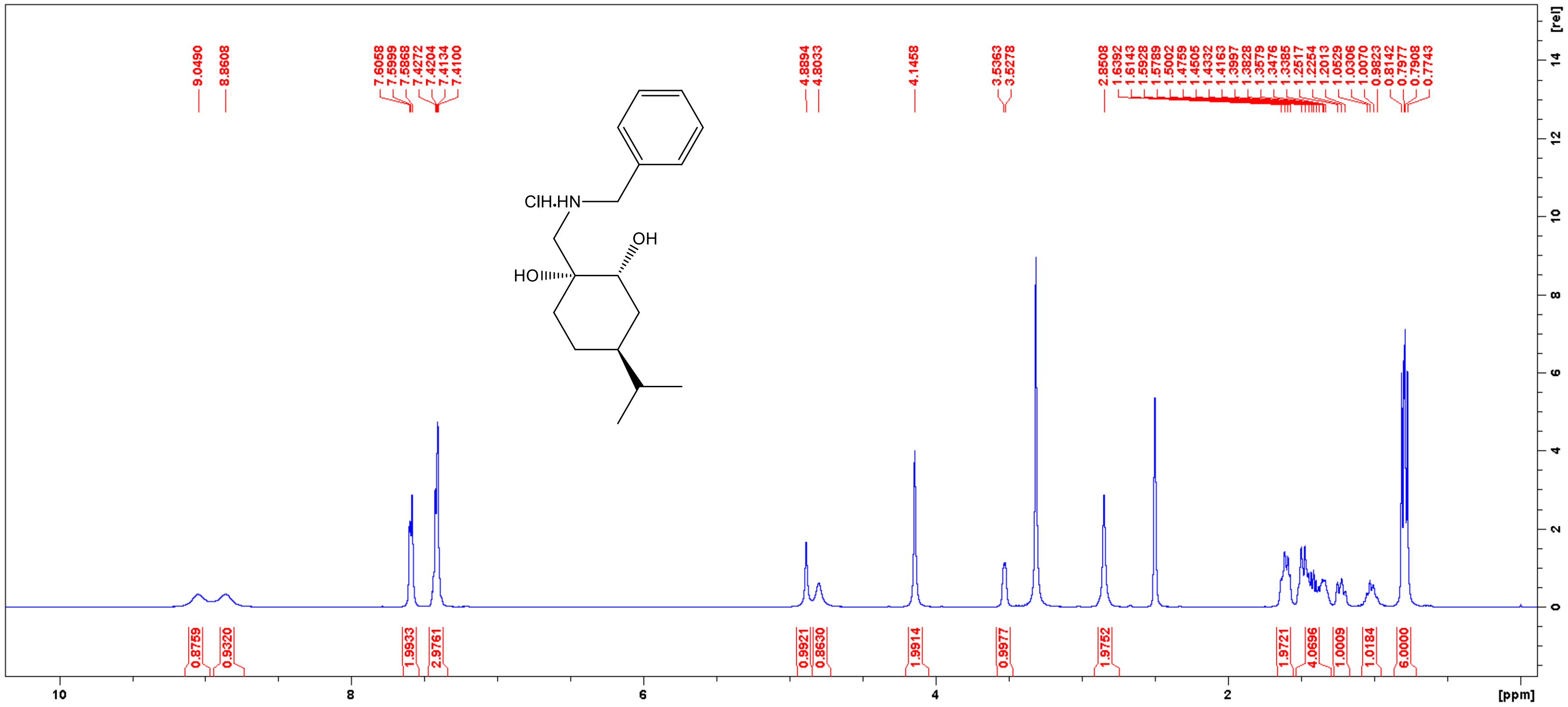

Figure S 50: ^{13}C-NMR of compound (1*R*,2*R*,4*S*)-1-((Benzylamino)methyl)-4-isopropylcyclohexane-1,2-diol hydrochloride **11a**

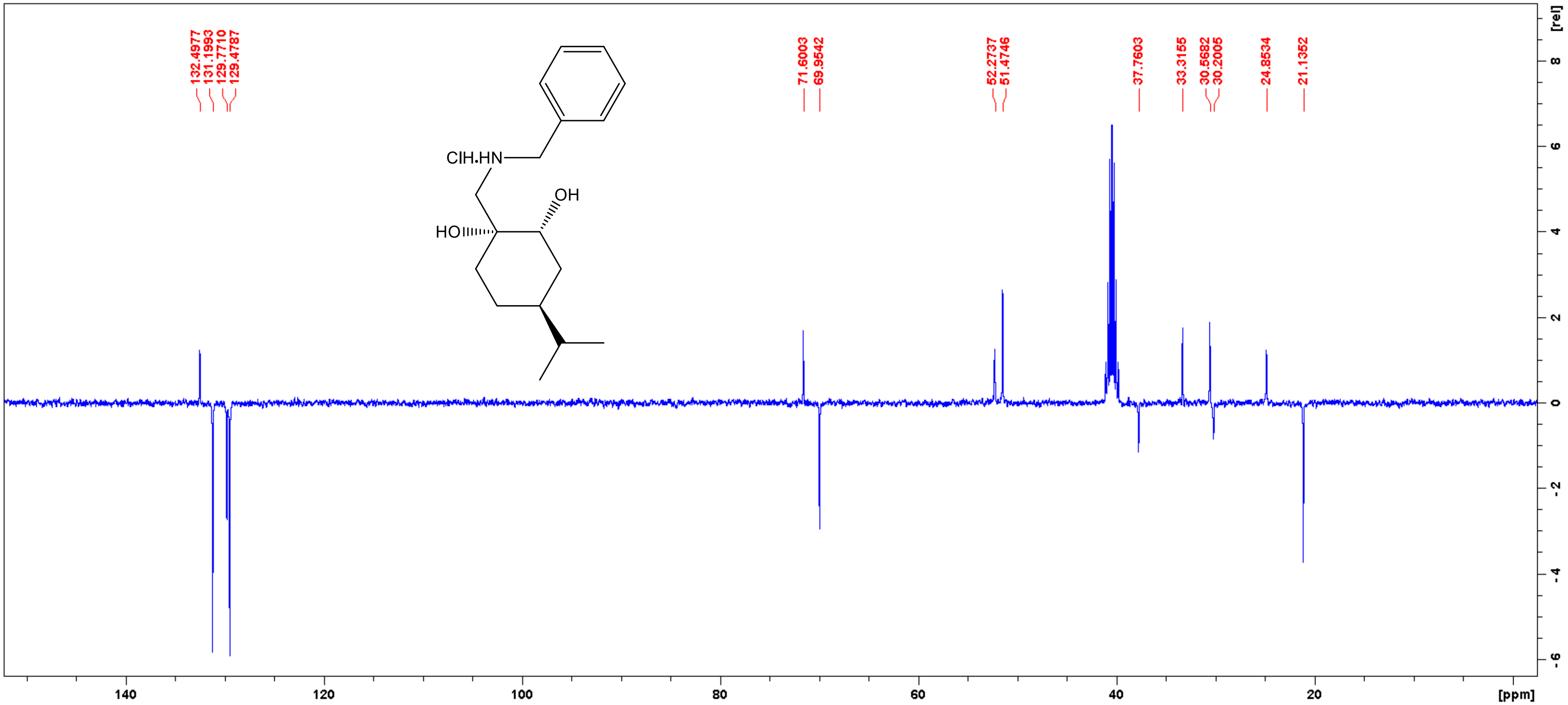

Figure S 51: COSY NMR of compound (1*R*,2*R*,4*S*)-1-((Benzylamino)methyl)-4-isopropylcyclohexane-1,2-diol hydrochloride **11a**

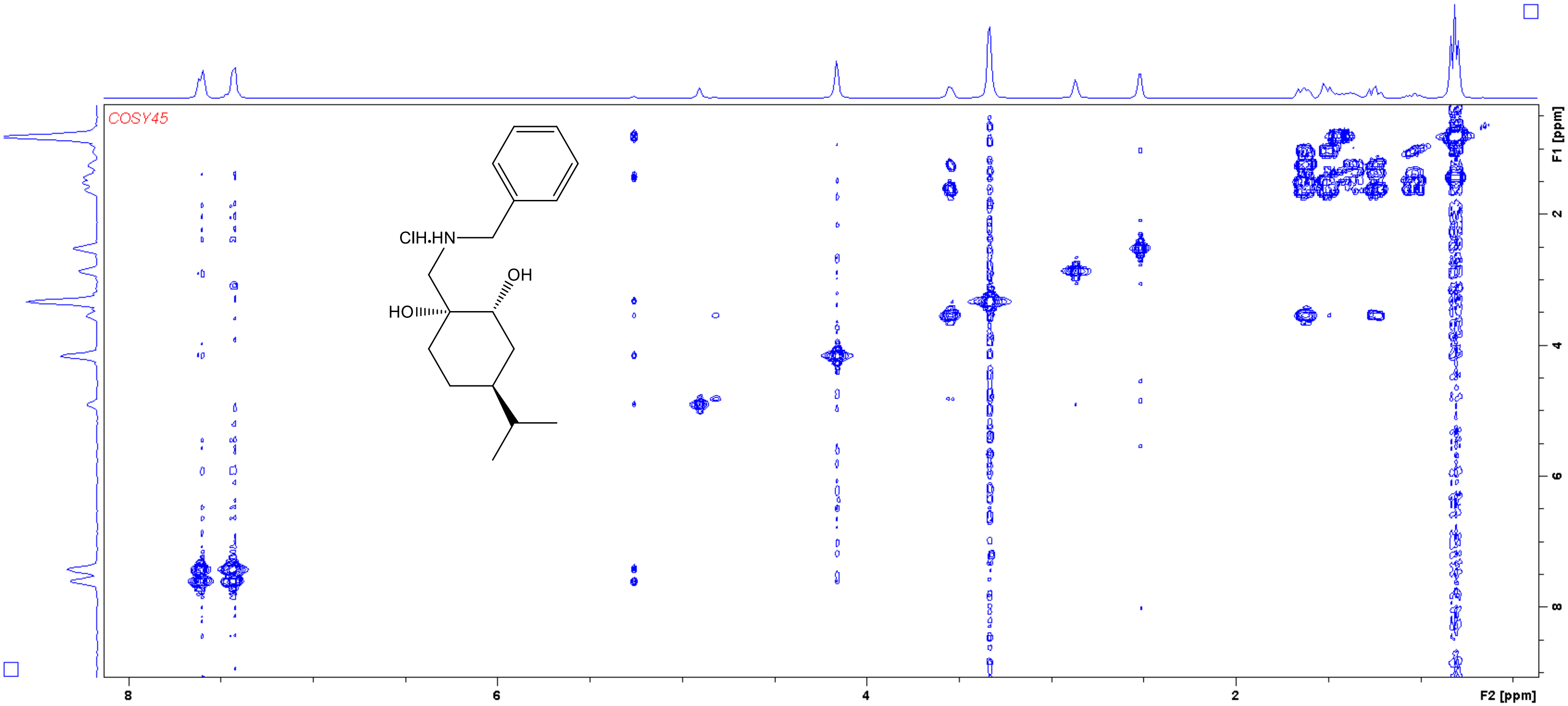

Figure S 52: NOESY NMR of compound (1*R*,2*R*,4*S*)-1-((Benzylamino)methyl)-4-isopropylcyclohexane-1,2-diol hydrochloride **11a**

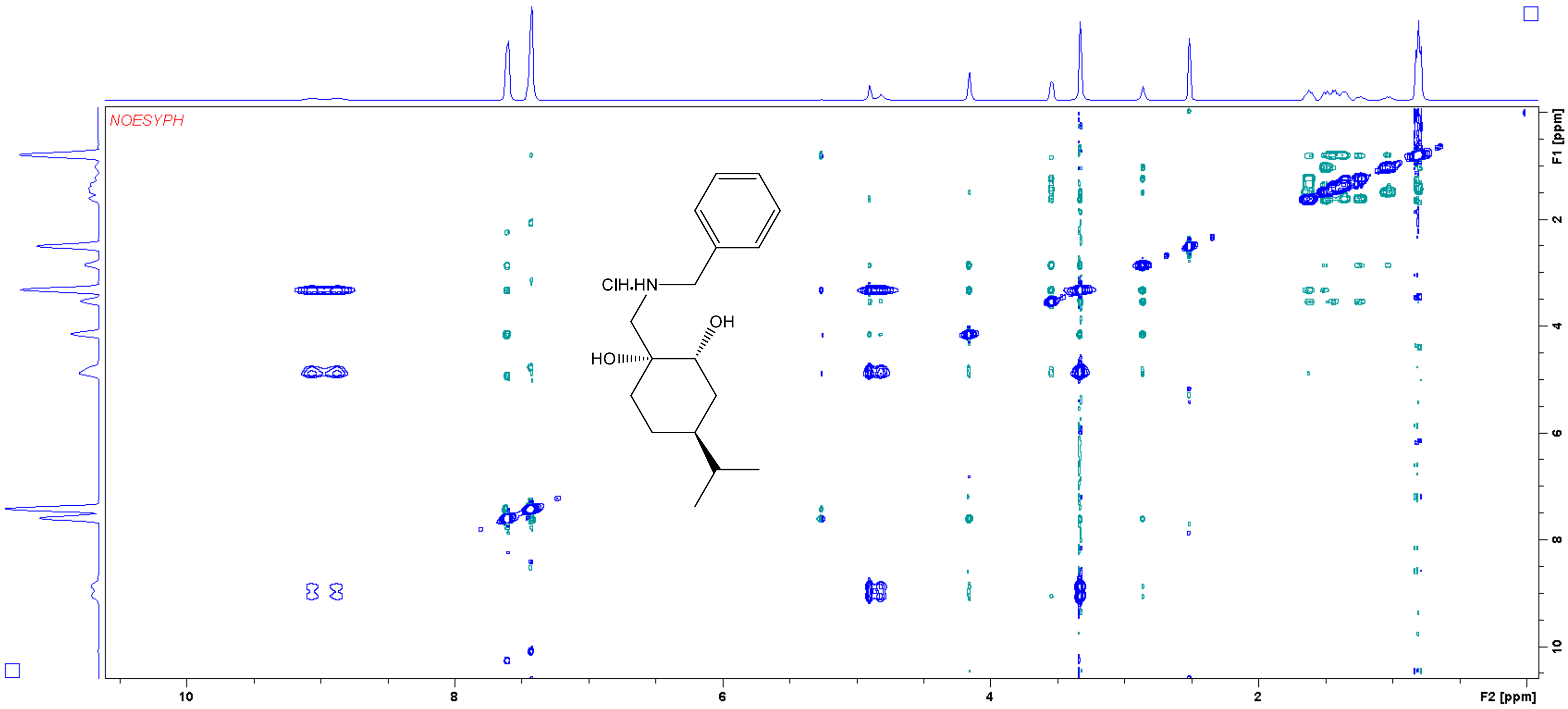

Figure S 53: HSQC NMR of compound (1*R*,2*R*,4*S*)-1-((Benzylamino)methyl)-4-isopropylcyclohexane-1,2-diol hydrochloride **11a**

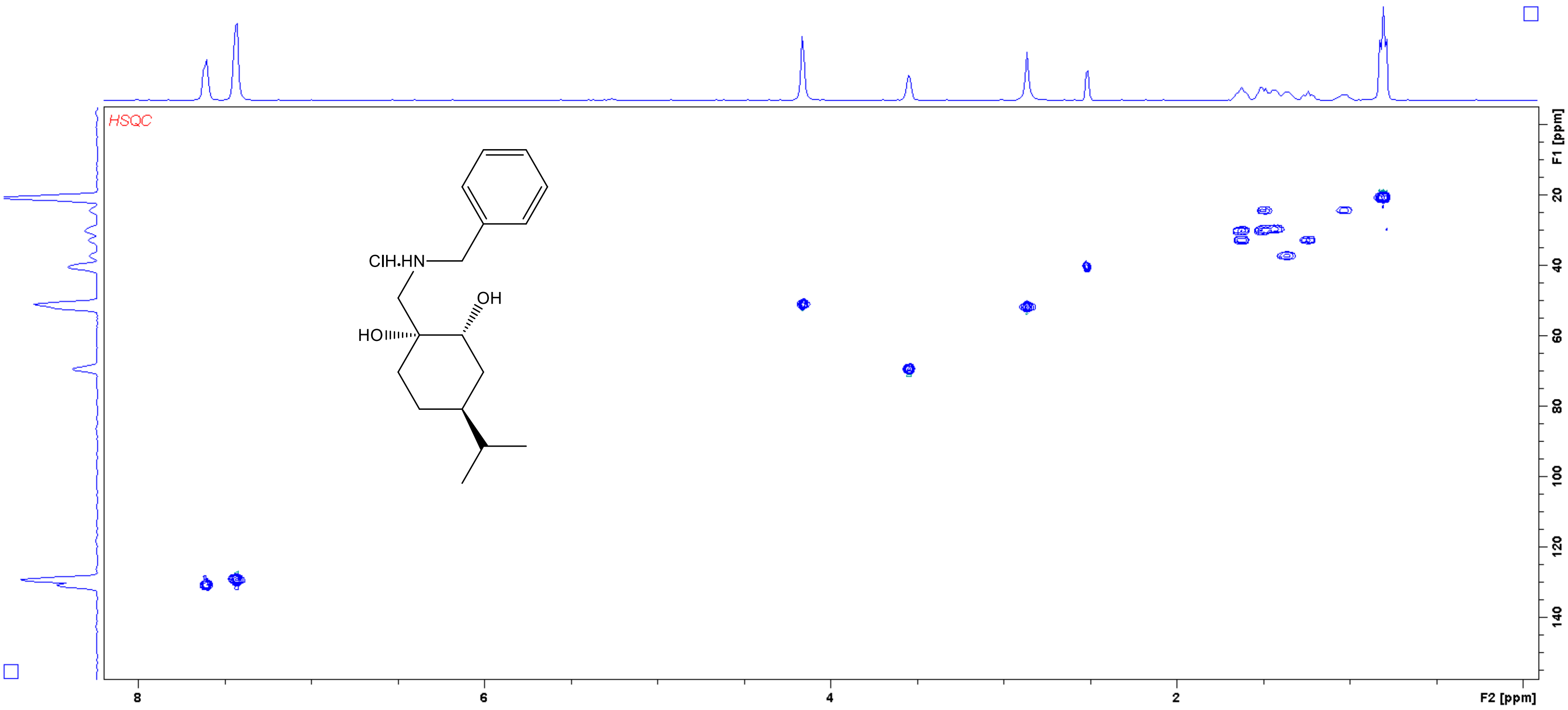

Figure S 54: HMBC NMR of compound (1*R*,2*R*,4*S*)-1-((Benzylamino)methyl)-4-isopropylcyclohexane-1,2-diol hydrochloride **11a**

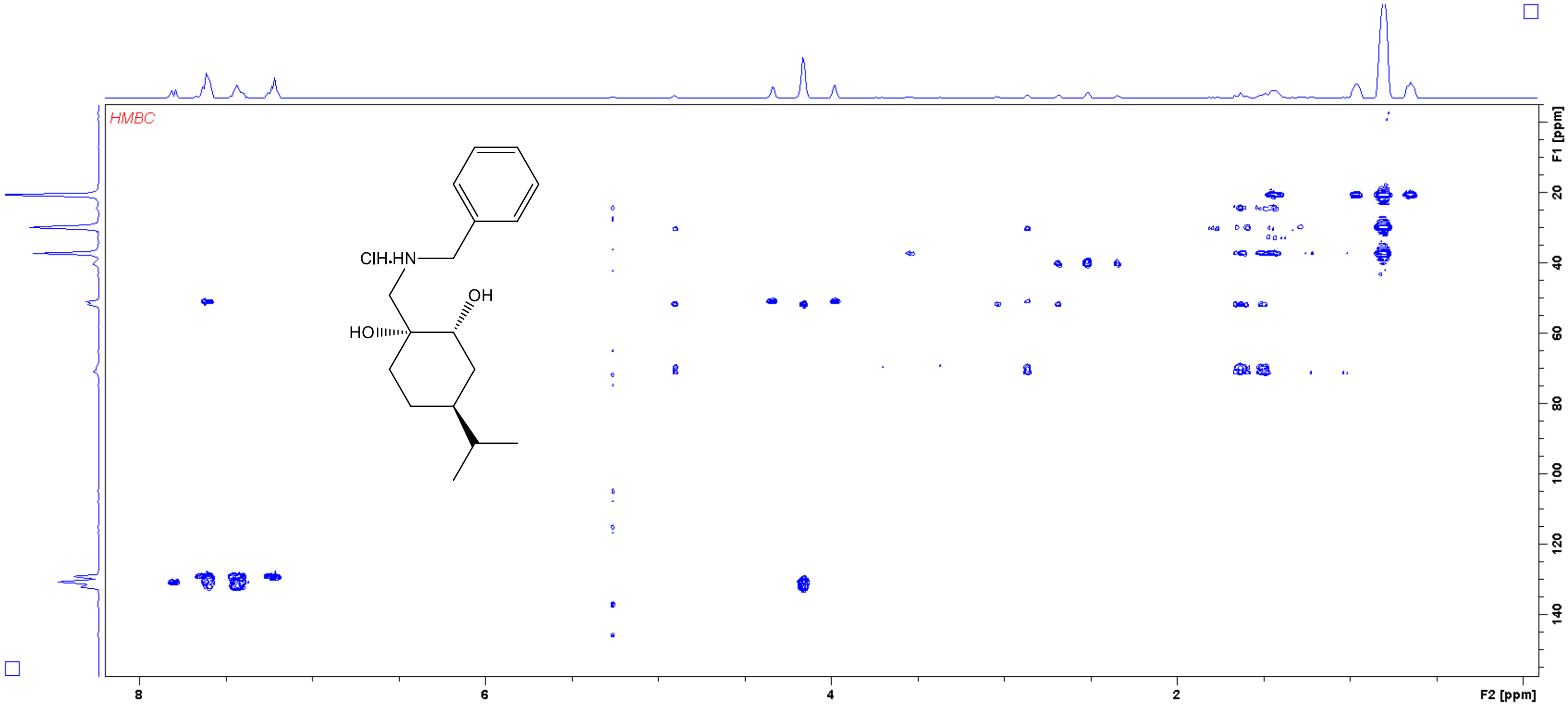

Figure S 55: ¹H-NMR of compound (1*R*,2*R*,4*S*)-4-Isopropyl-1-((((*S*)-1-phenylethyl)amino)methyl)cyclohexane-1,2-diol hydrochloride **11b**

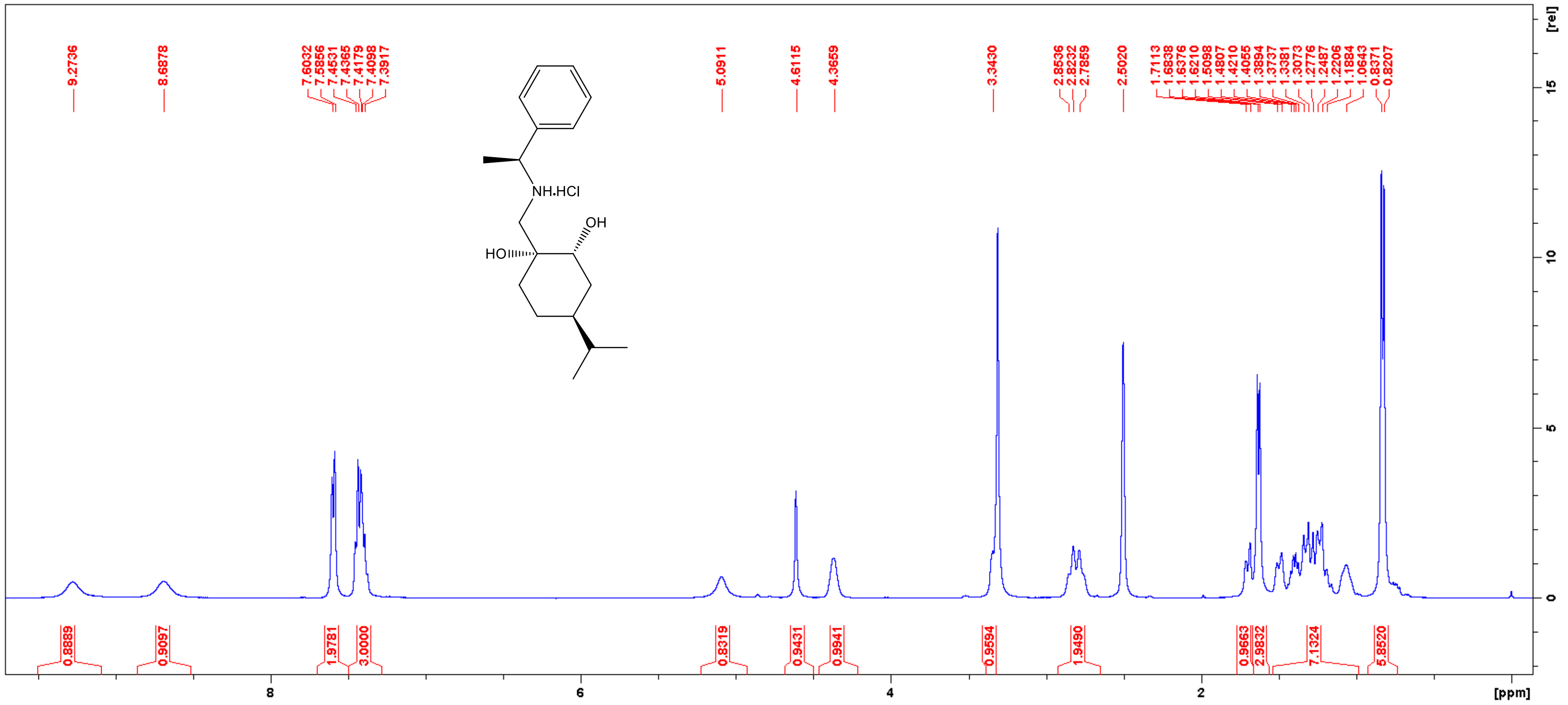

Figure S 56: ^{13}C-NMR of compound (1*R*,2*R*,4*S*)-4-Isopropyl-1-((((*S*)-1-phenylethyl)amino)methyl)cyclohexane-1,2-diol hydrochloride **11b**

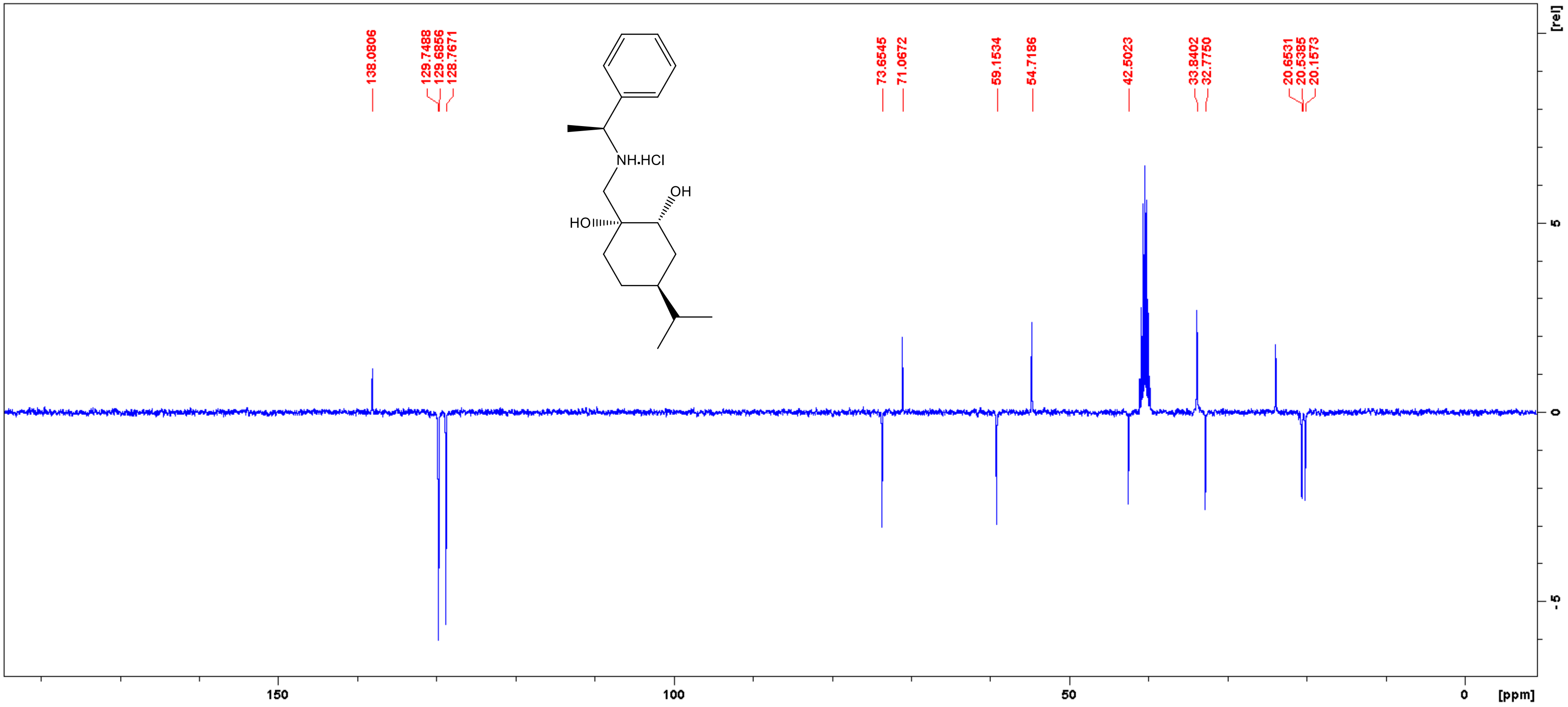

Figure S 57: COSY NMR of compound (1*R*,2*R*,4*S*)-4-Isopropyl-1-((((*S*)-1-phenylethyl)amino)methyl)cyclohexane-1,2-diol hydrochloride **11b**

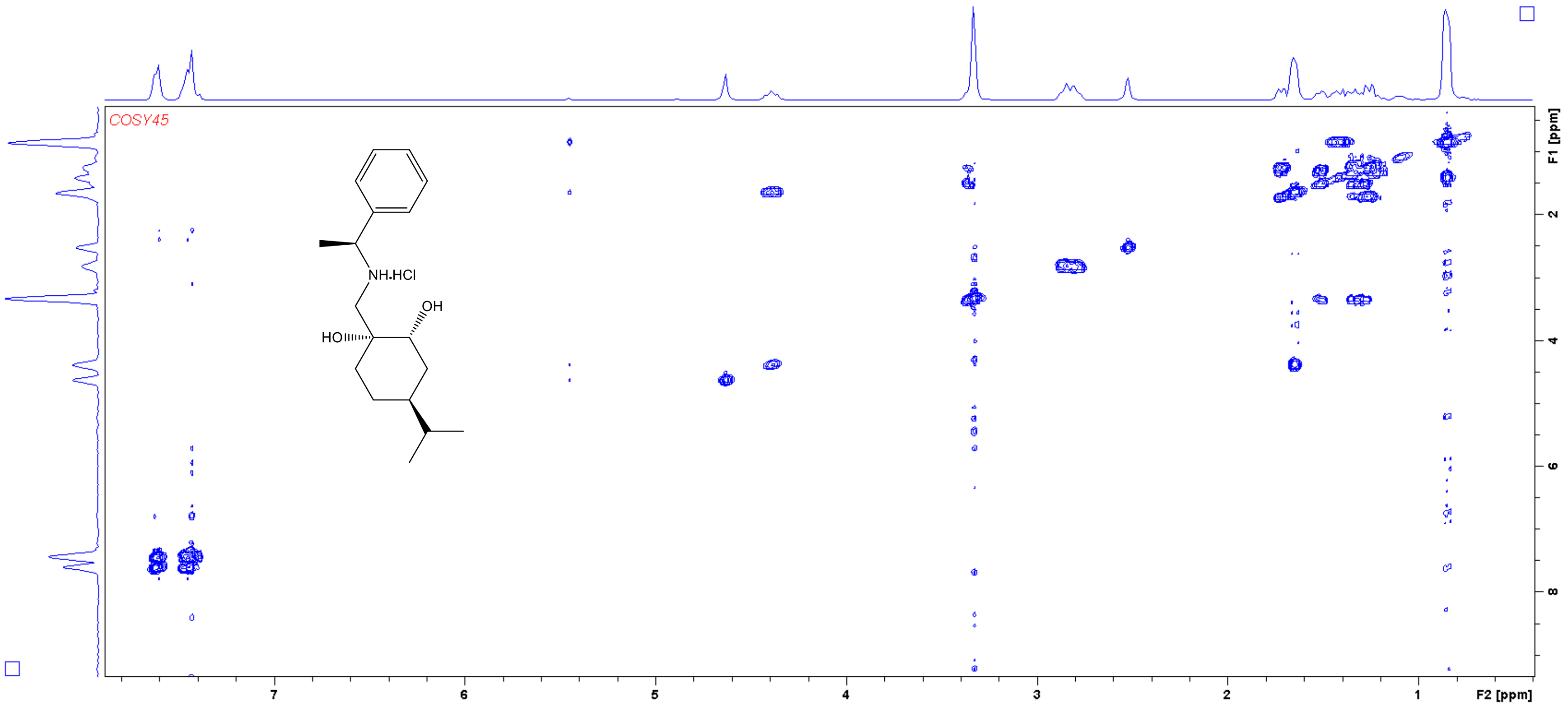

Figure S 58: NOESY NMR of compound (1*R*,2*R*,4*S*)-4-Isopropyl-1-((((*S*)-1-phenylethyl)amino)methyl)cyclohexane-1,2-diol hydrochloride **11b**

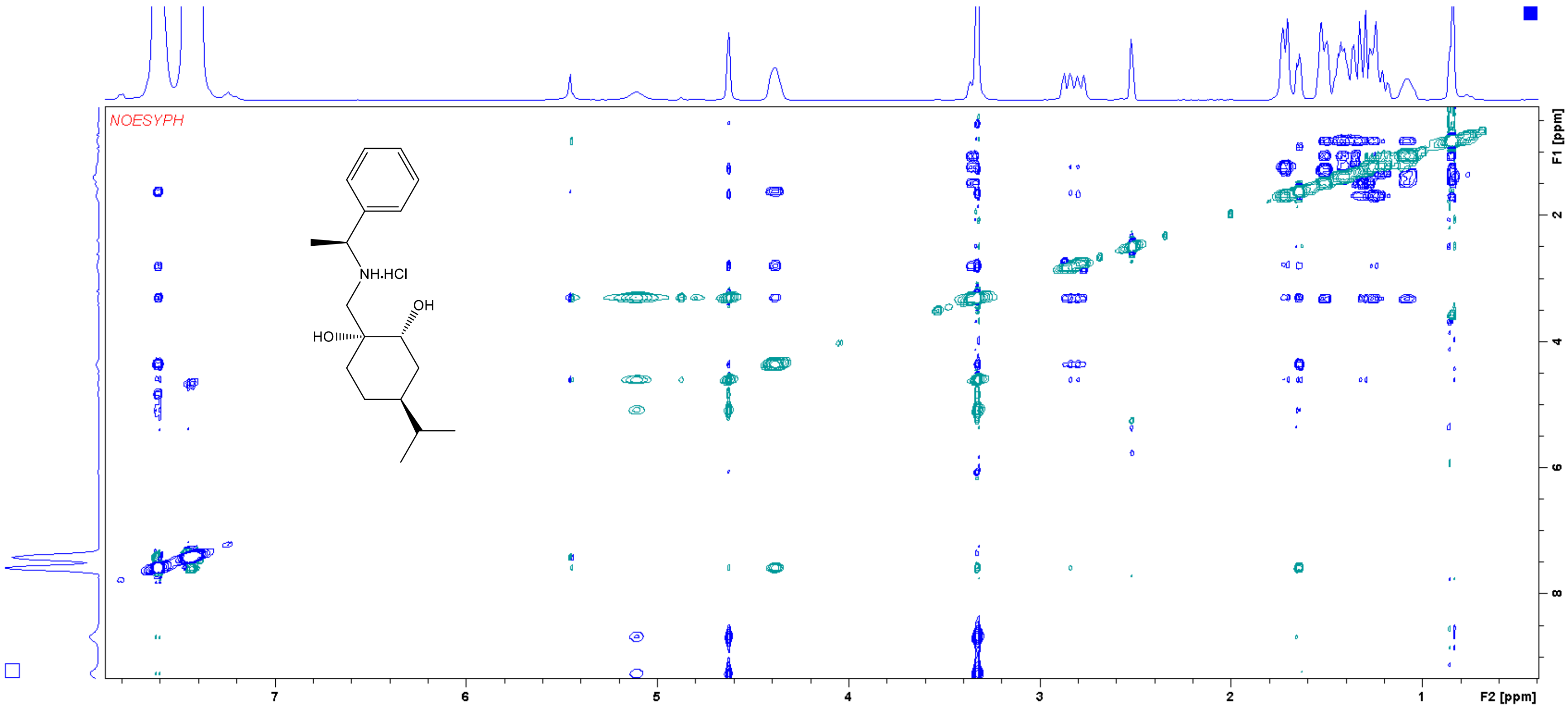

Figure S 59: HSQC NMR of compound (1*R*,2*R*,4*S*)-4-Isopropyl-1-((((*S*)-1-phenylethyl)amino)methyl)cyclohexane-1,2-diol hydrochloride **11b**

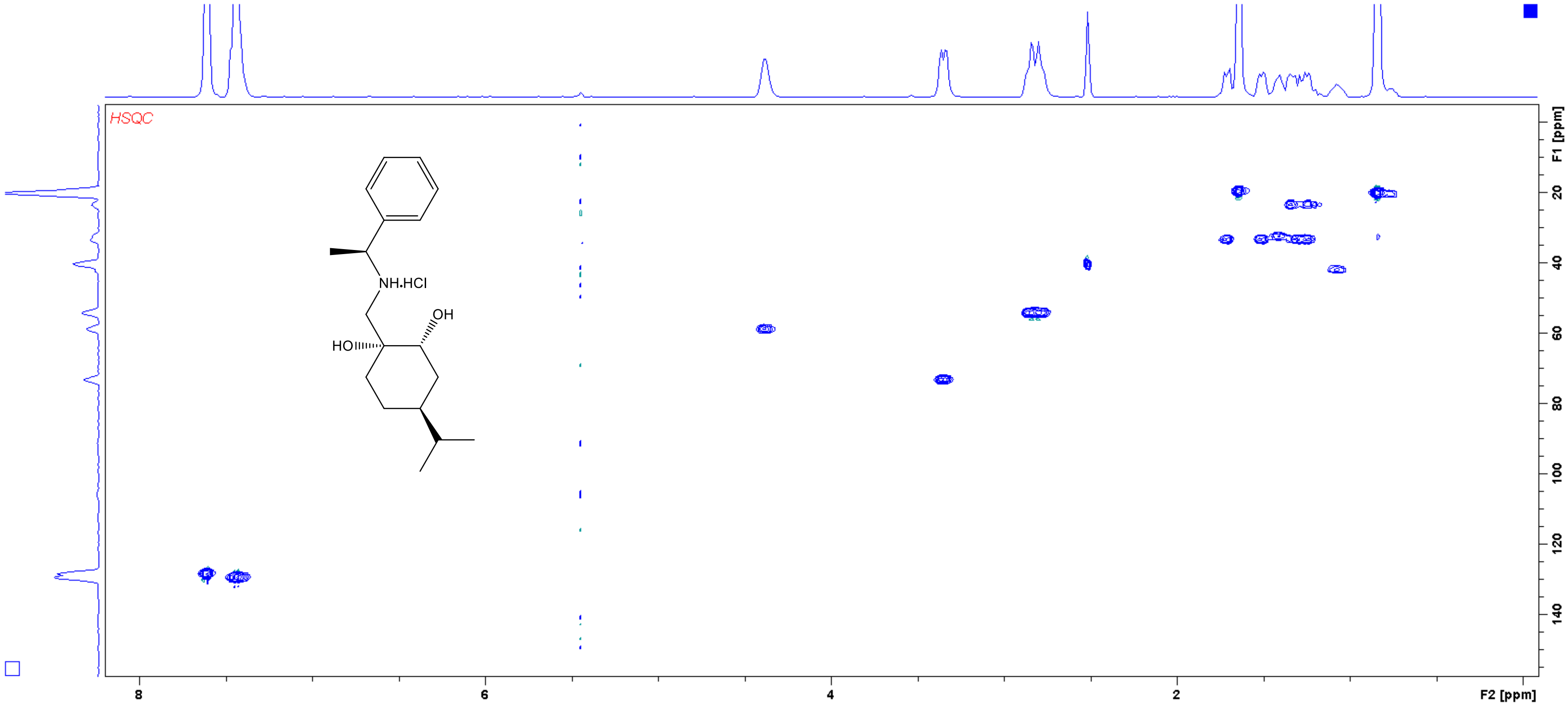

Figure S 60: ^{1}H-NMR of compound (1*R*,2*R*,4*S*)-4-Isopropyl-1-((((*R*)-1-phenylethyl)amino)methyl)cyclohexane-1,2-diol hydrochloride **11c**

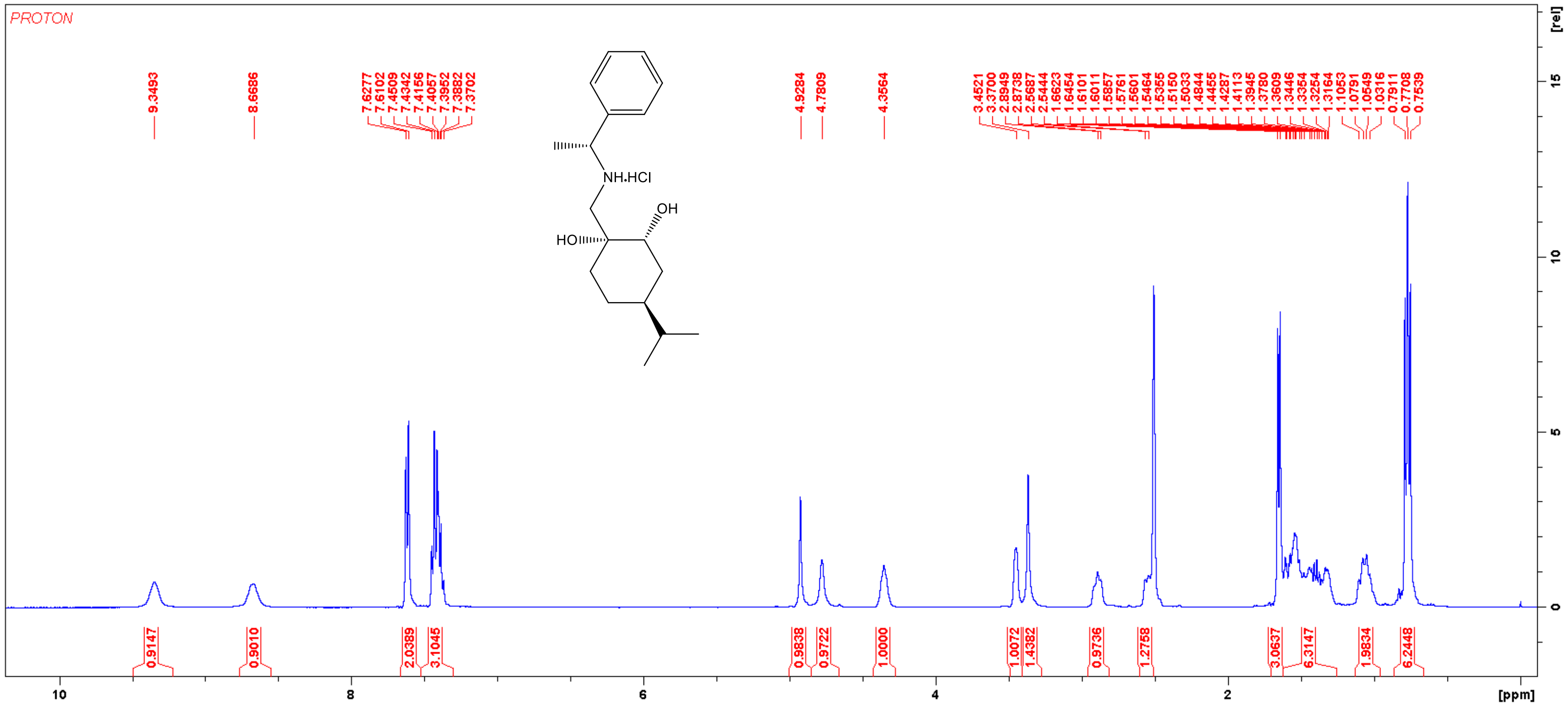

Figure S 61: ^{13}C-NMR of compound (1*R*,2*R*,4*S*)-4-Isopropyl-1-((((*R*)-1-phenylethyl)amino)methyl)cyclohexane-1,2-diol hydrochloride **11c**

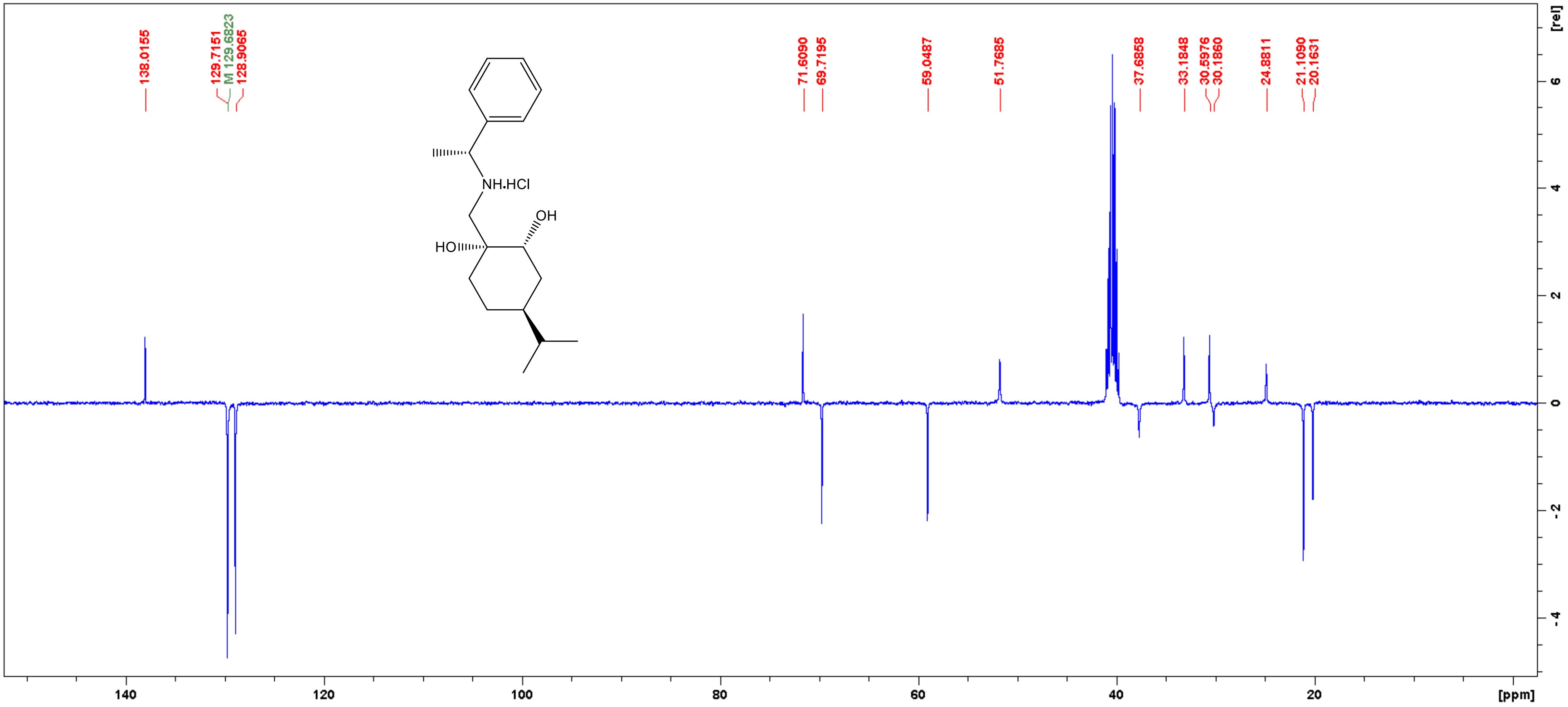

Figure S 62: ^{1}H-NMR of compound (1*S*,2*S*,4*S*)-1-(Aminomethyl)-4-isopropylcyclohexane-1,2-diol hydrochloride **8**

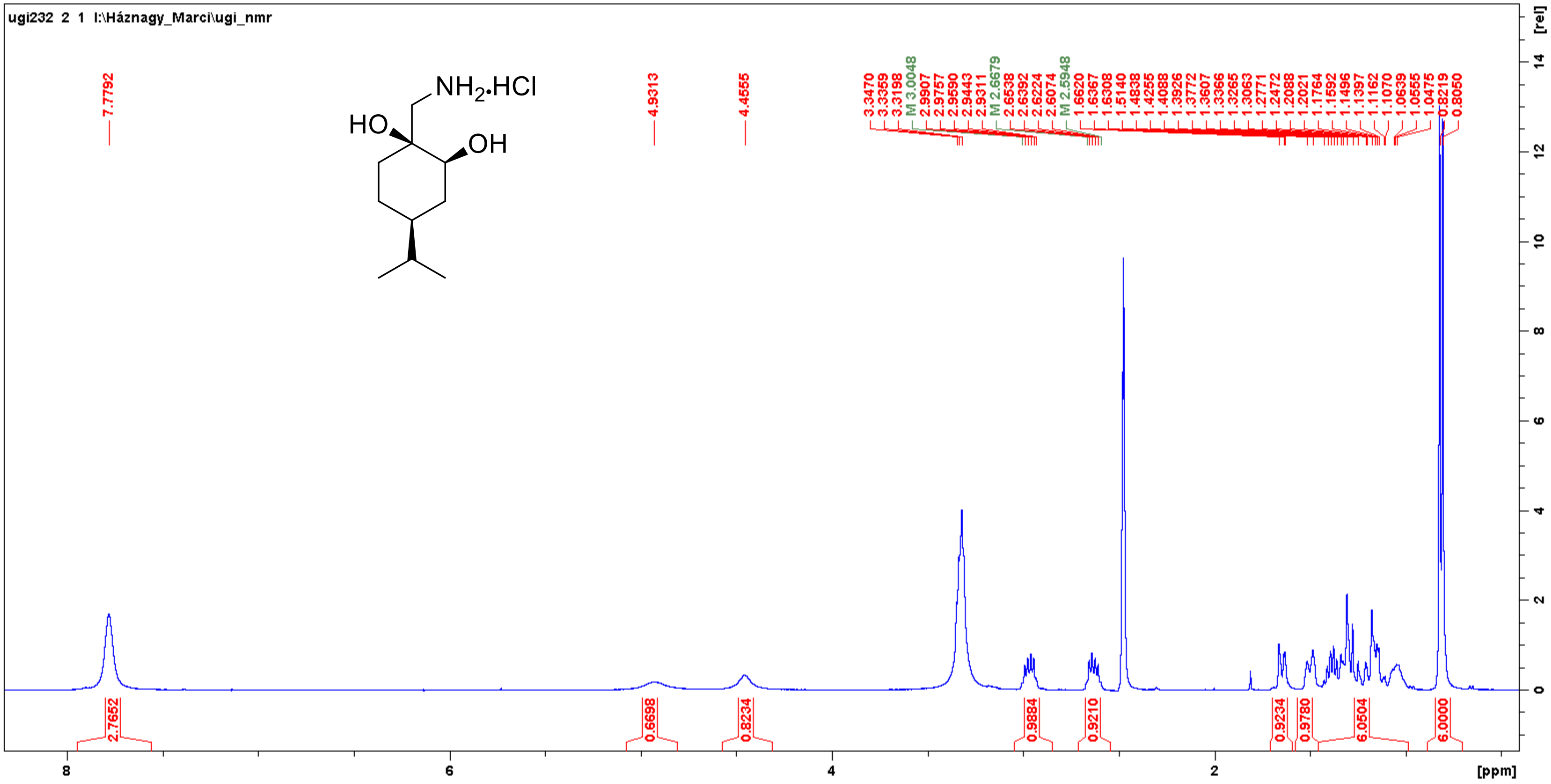

Figure S 63: ^{13}C-NMR of compound (1*S*,2*S*,4*S*)-1-(Aminomethyl)-4-isopropylcyclohexane-1,2-diol hydrochloride **8**

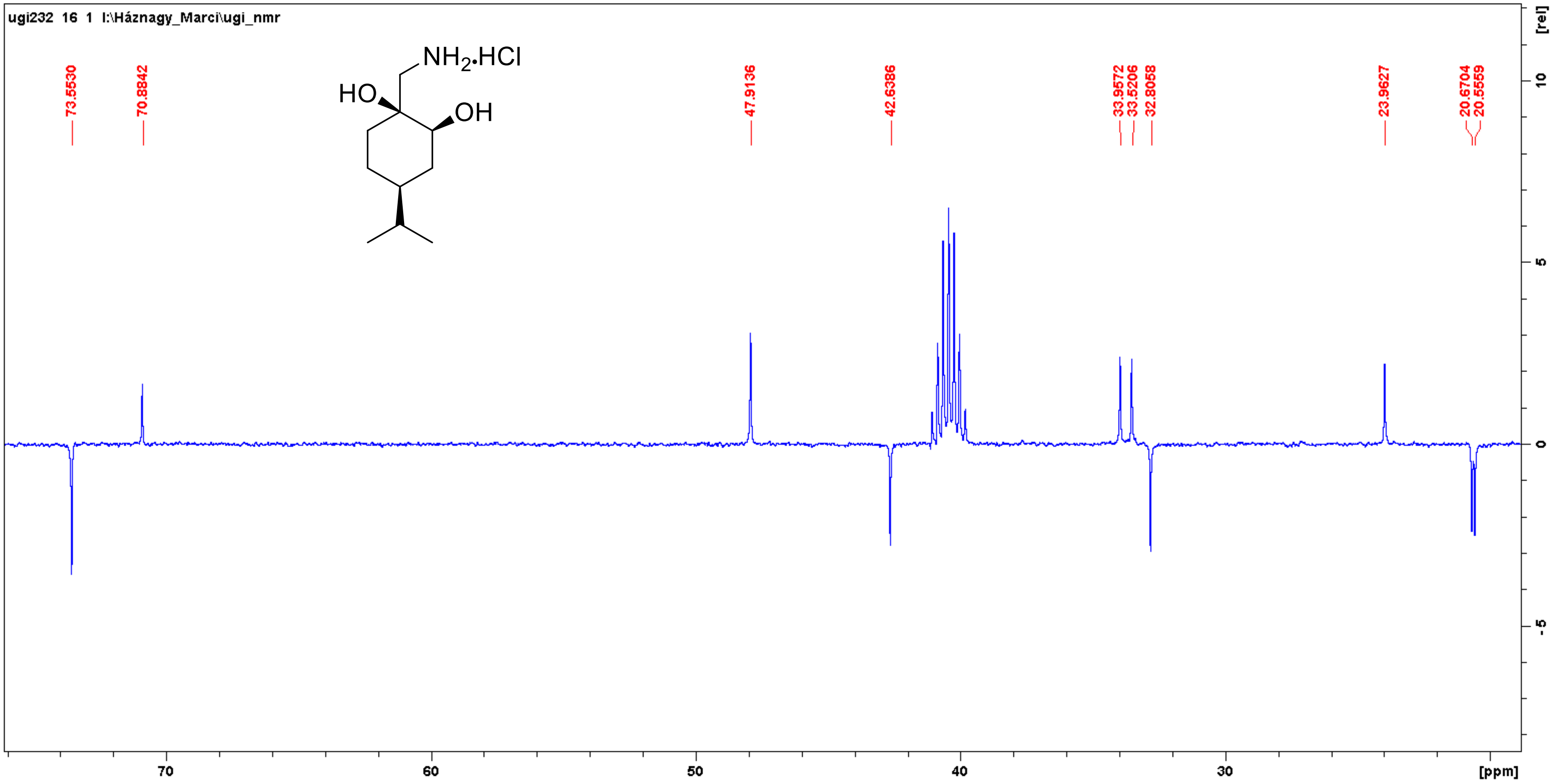

Figure S 64: COSY NMR of compound (1*S*,2*S*,4*S*)-1-(Aminomethyl)-4-isopropylcyclohexane-1,2-diol hydrochloride **8**

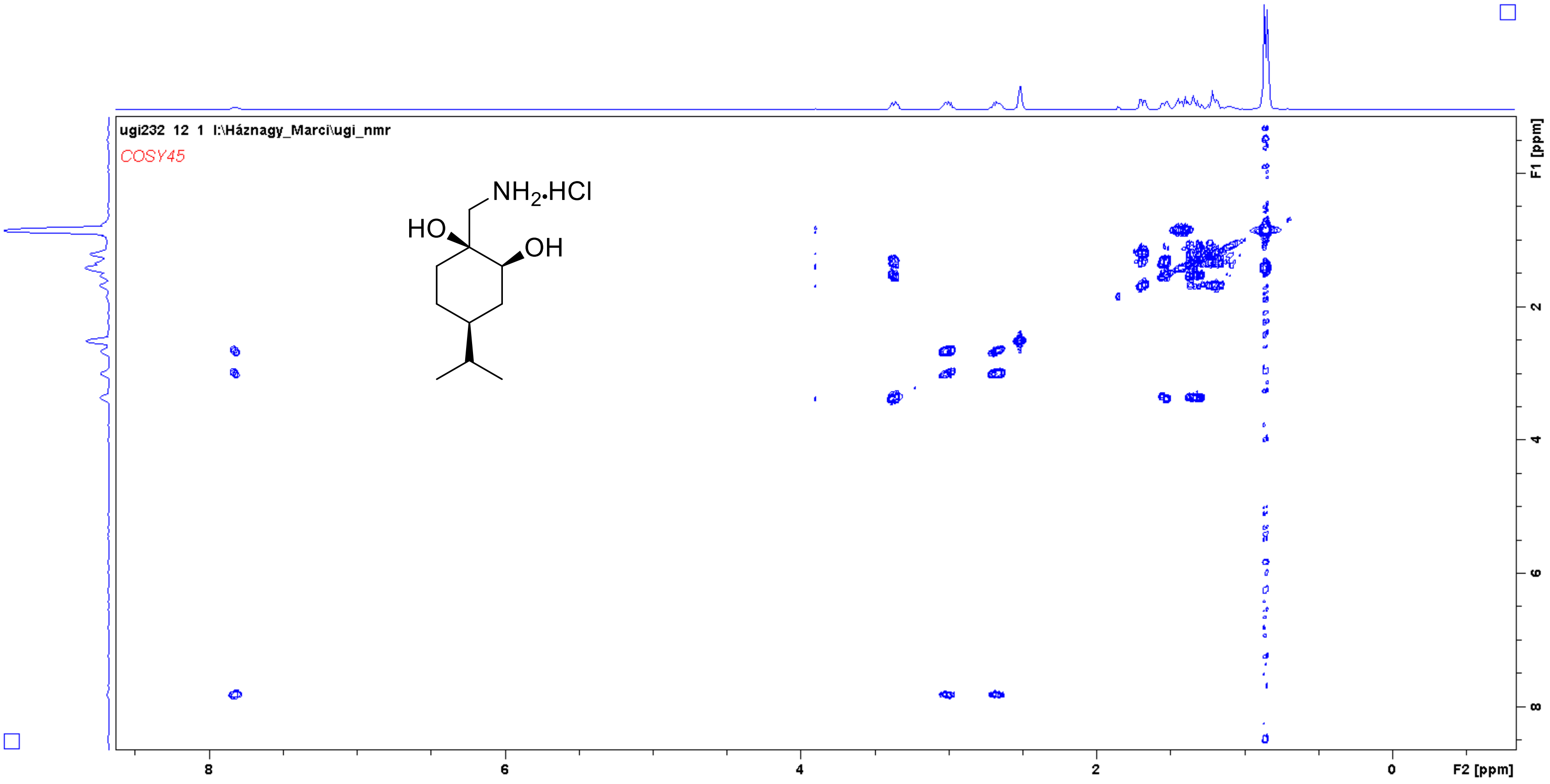

Figure S 65: HSQC NMR of compound (1*S*,2*S*,4*S*)-1-(Aminomethyl)-4-isopropylcyclohexane-1,2-diol hydrochloride **8**

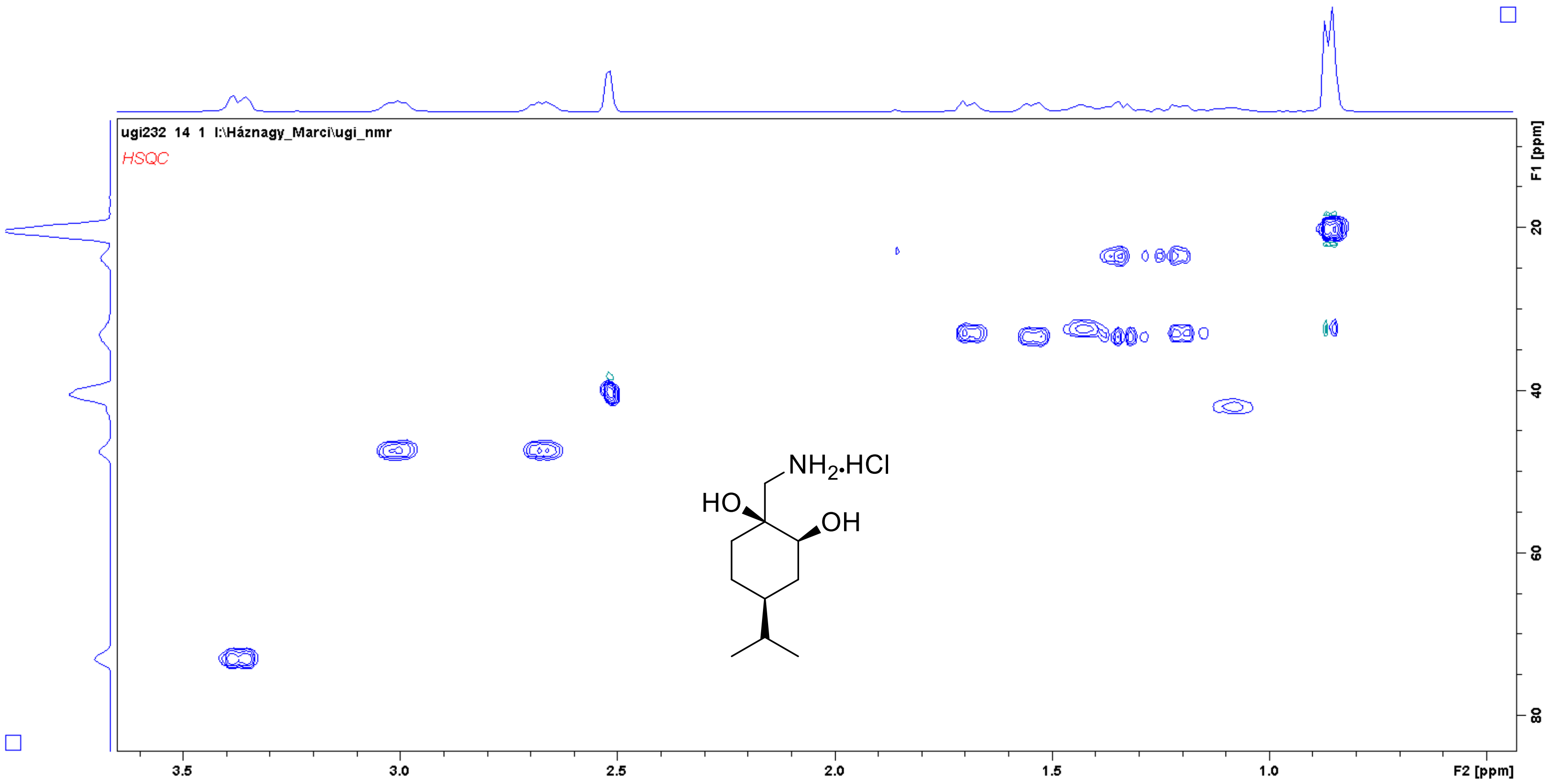

Figure S 66: HMBC NMR of compound (1*S*,2*S*,4*S*)-1-(Aminomethyl)-4-isopropylcyclohexane-1,2-diol hydrochloride **8**

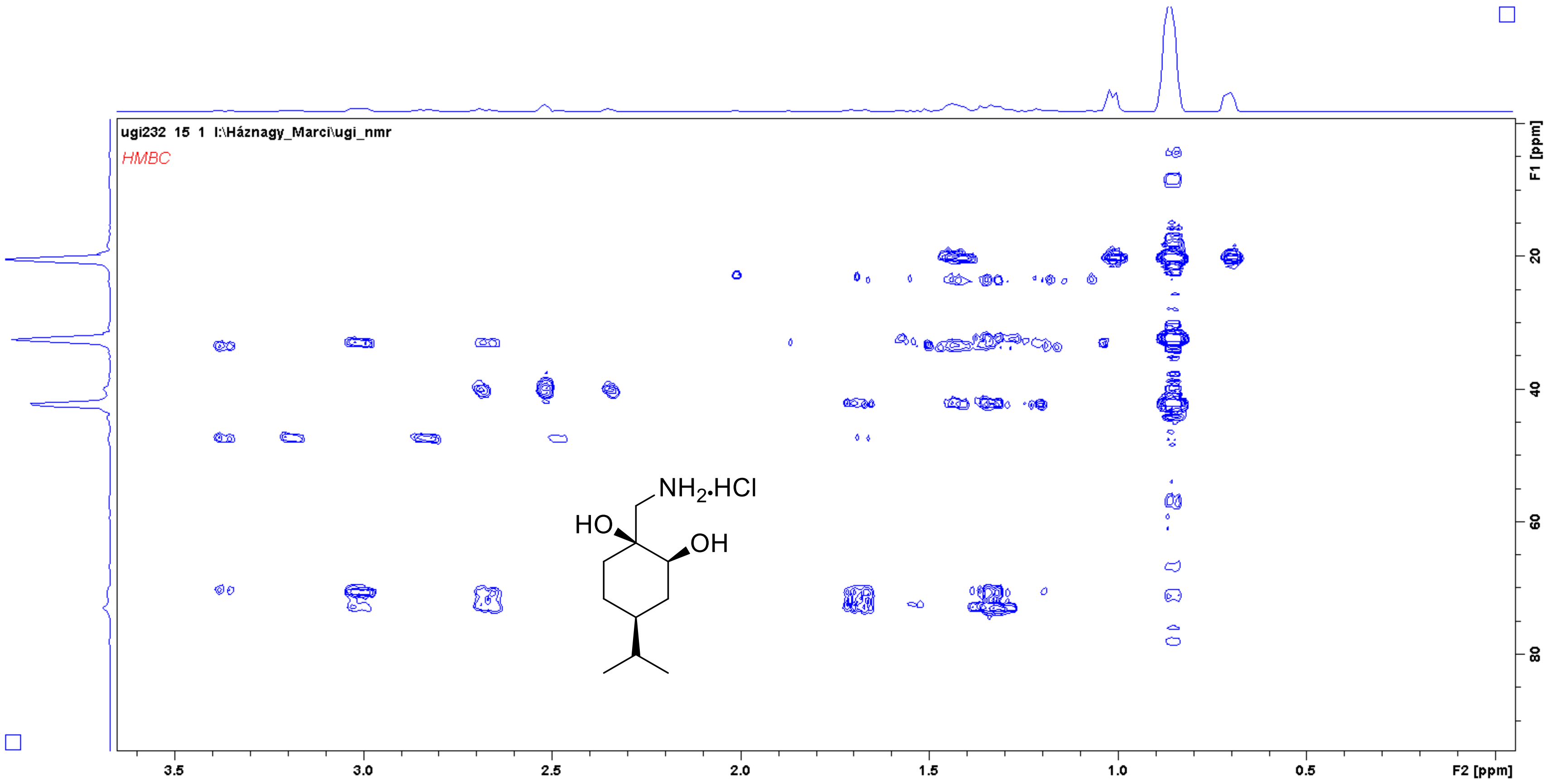

Figure S 67: ^{1}H-NMR of compound (1*R*,2*R*,4*S*)-1-(Aminomethyl)-4-isopropylcyclohexane-1,2-diol hydrochloride **12**

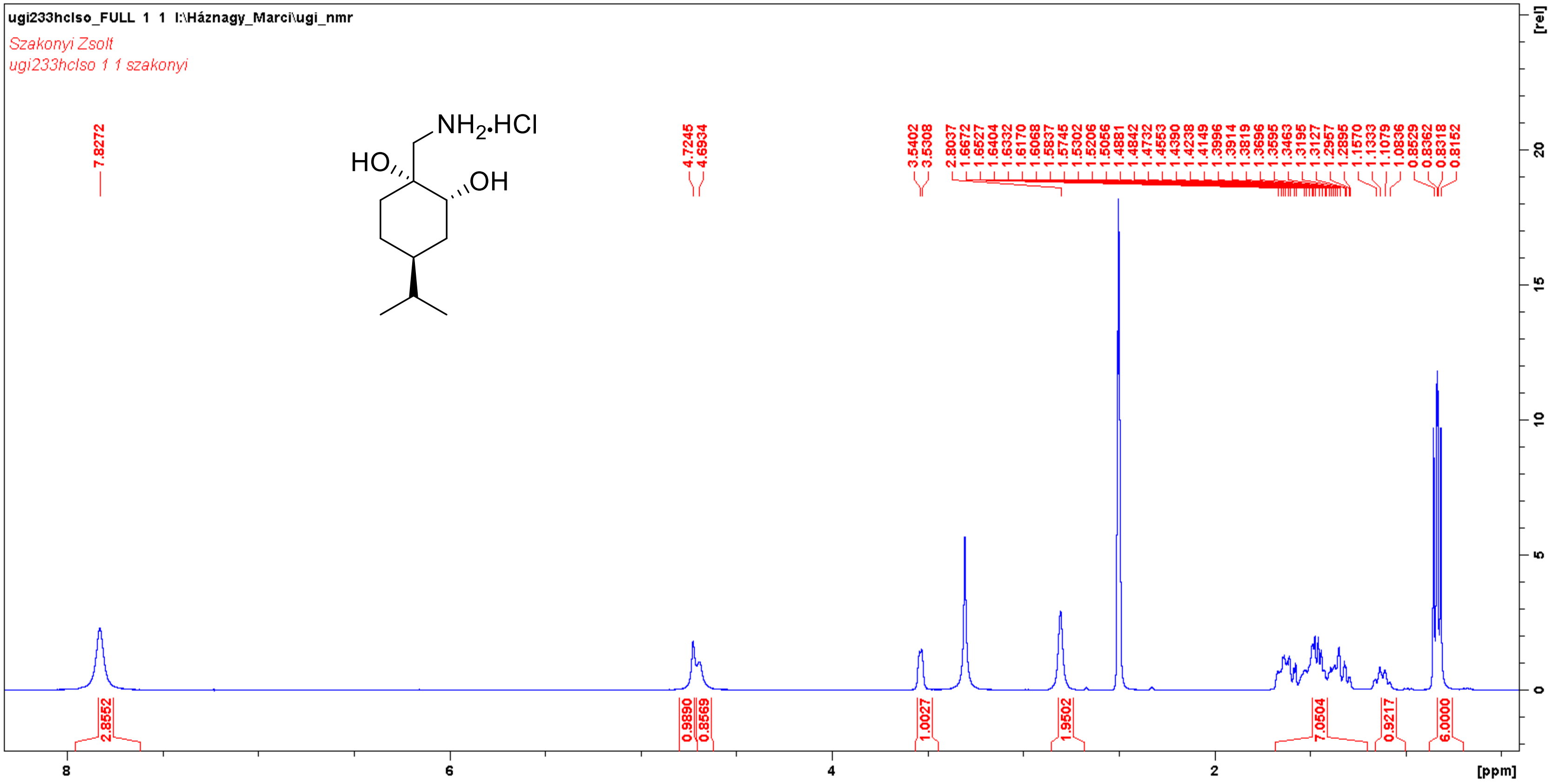

Figure S 68: ^{13}**C-NMR of compound** (1*R*,2*R*,4*S*)-1-(Aminomethyl)-4-isopropylcyclohexane-1,2-diol hydrochloride **12**

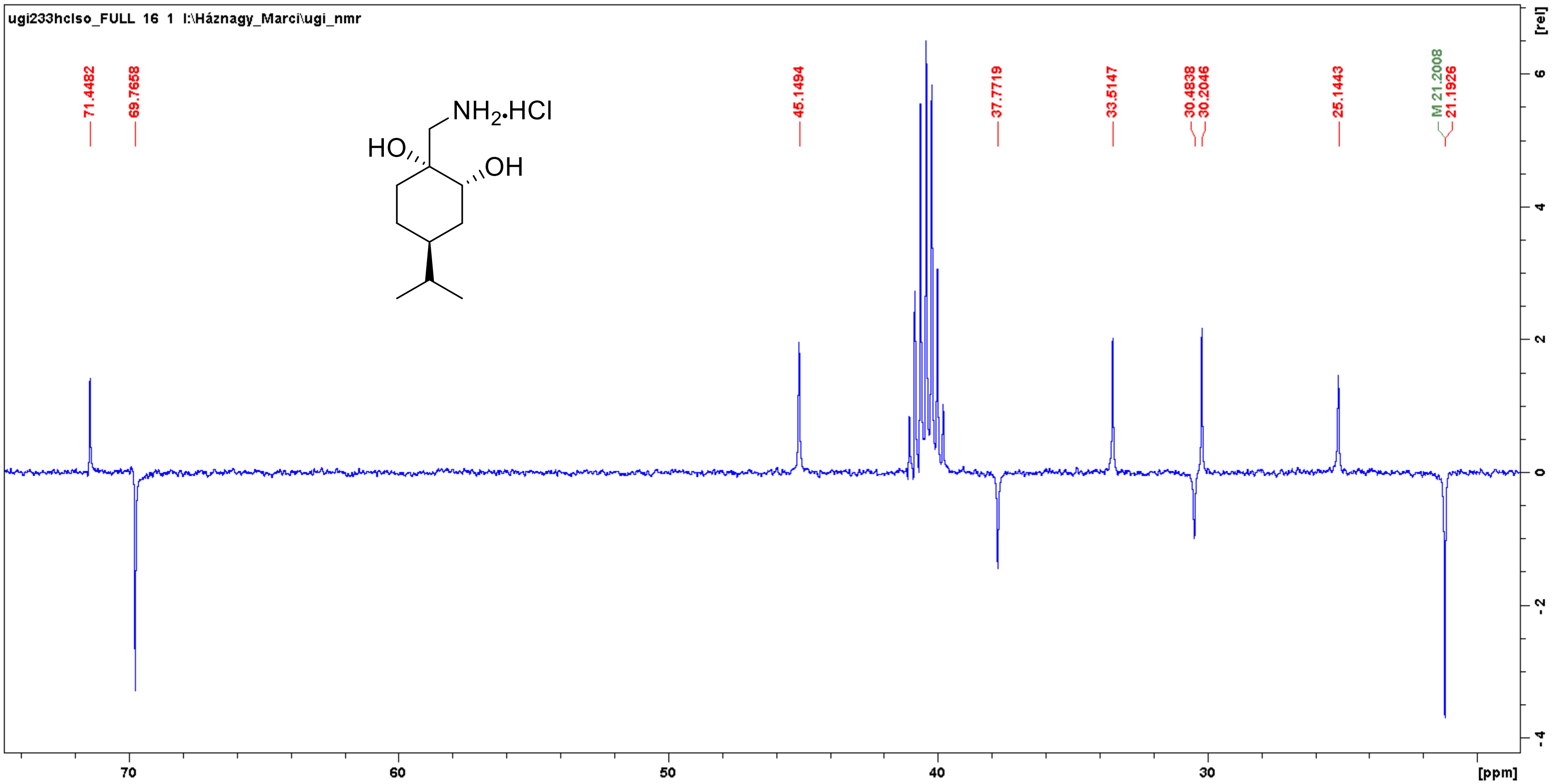

Figure S 69: COSY NMR of compound (1*R*,2*R*,4*S*)-1-(Aminomethyl)-4-isopropylcyclohexane-1,2-diol hydrochloride **12**

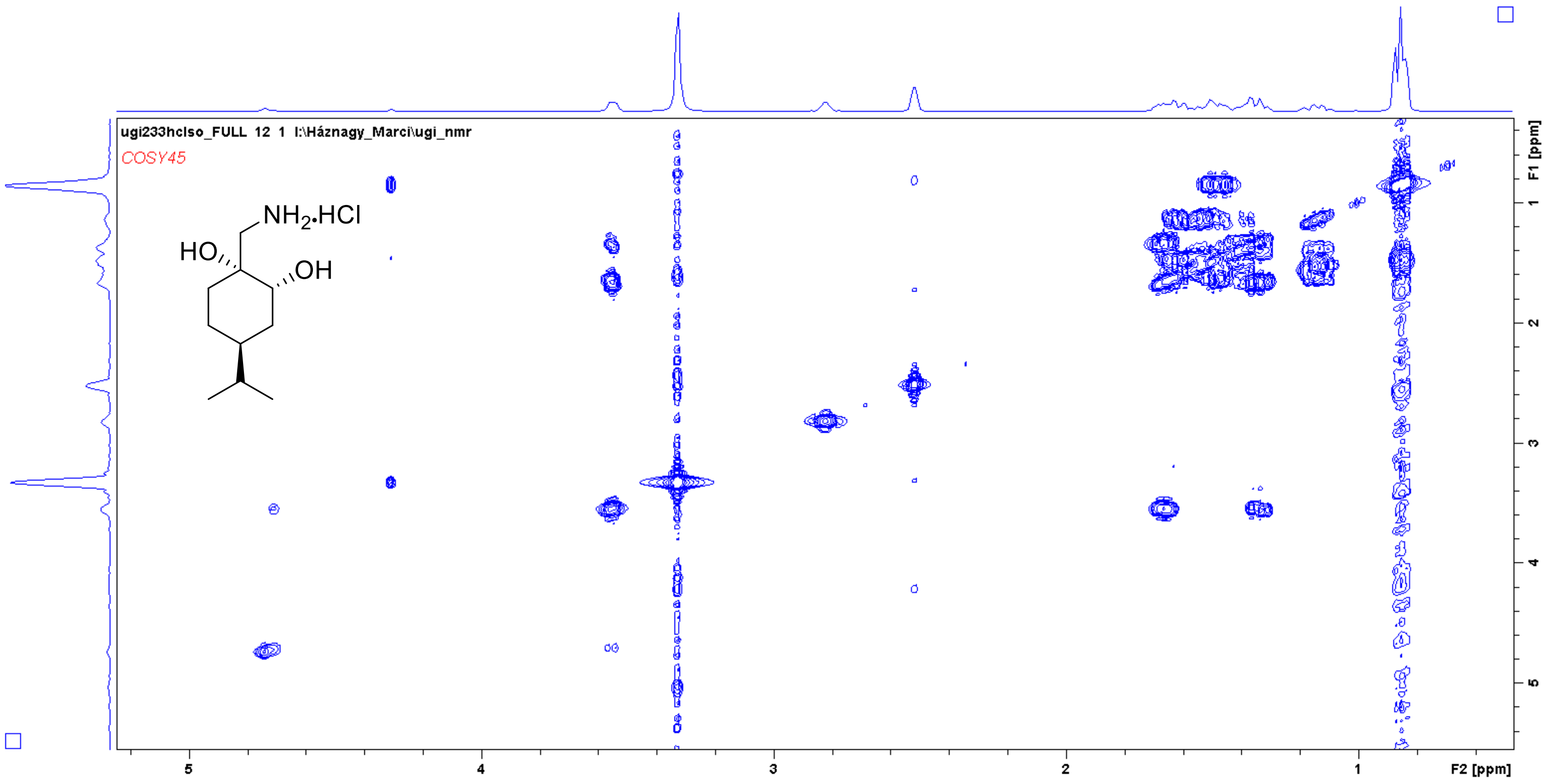

Figure S 70: HSQC NMR of compound (1*R*,2*R*,4*S*)-1-(Aminomethyl)-4-isopropylcyclohexane-1,2-diol hydrochloride **12**

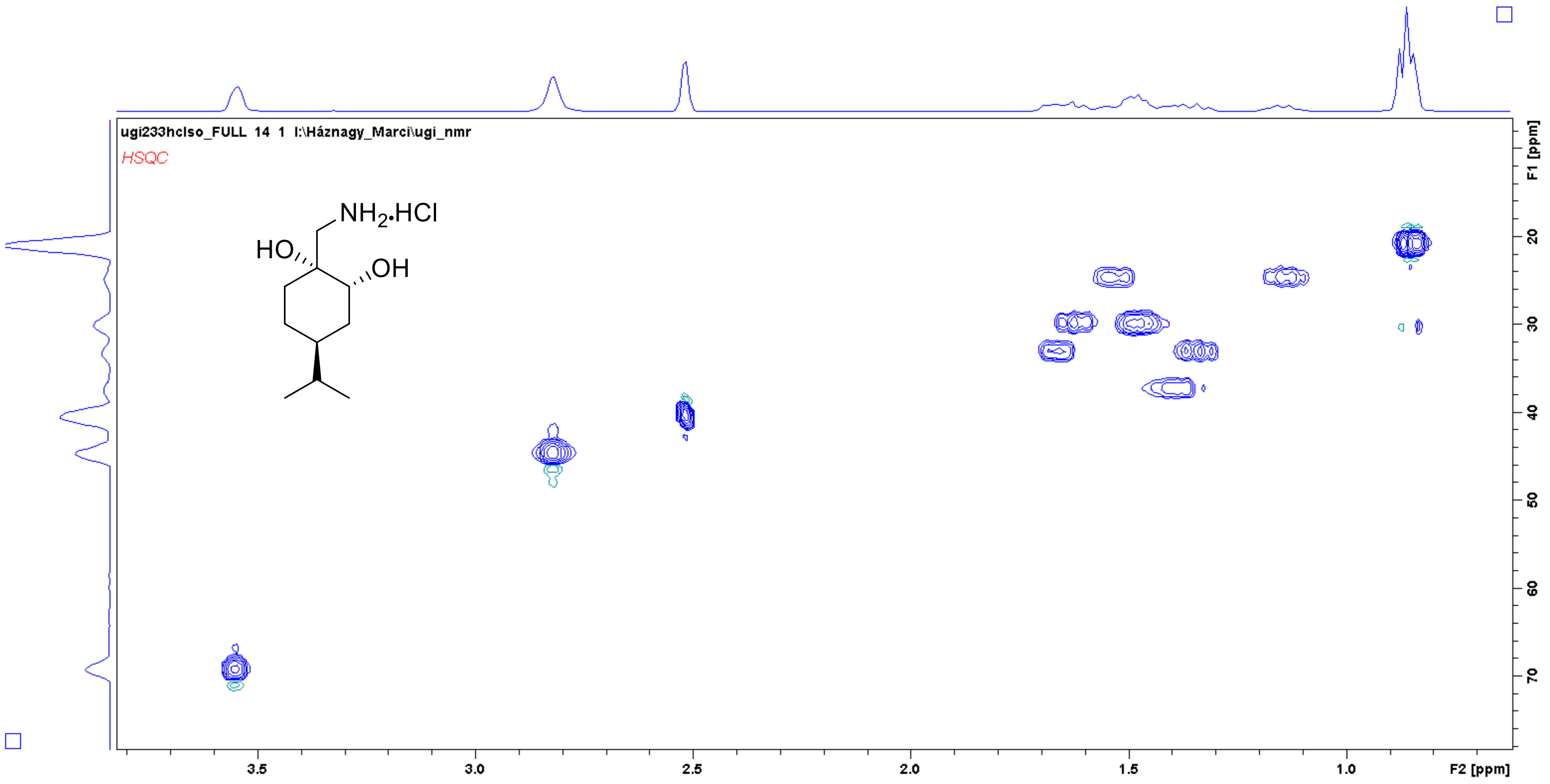

Figure S 71: HMBC NMR of compound (1*R*,2*R*,4*S*)-1-(Aminomethyl)-4-isopropylcyclohexane-1,2-diol hydrochloride **12**

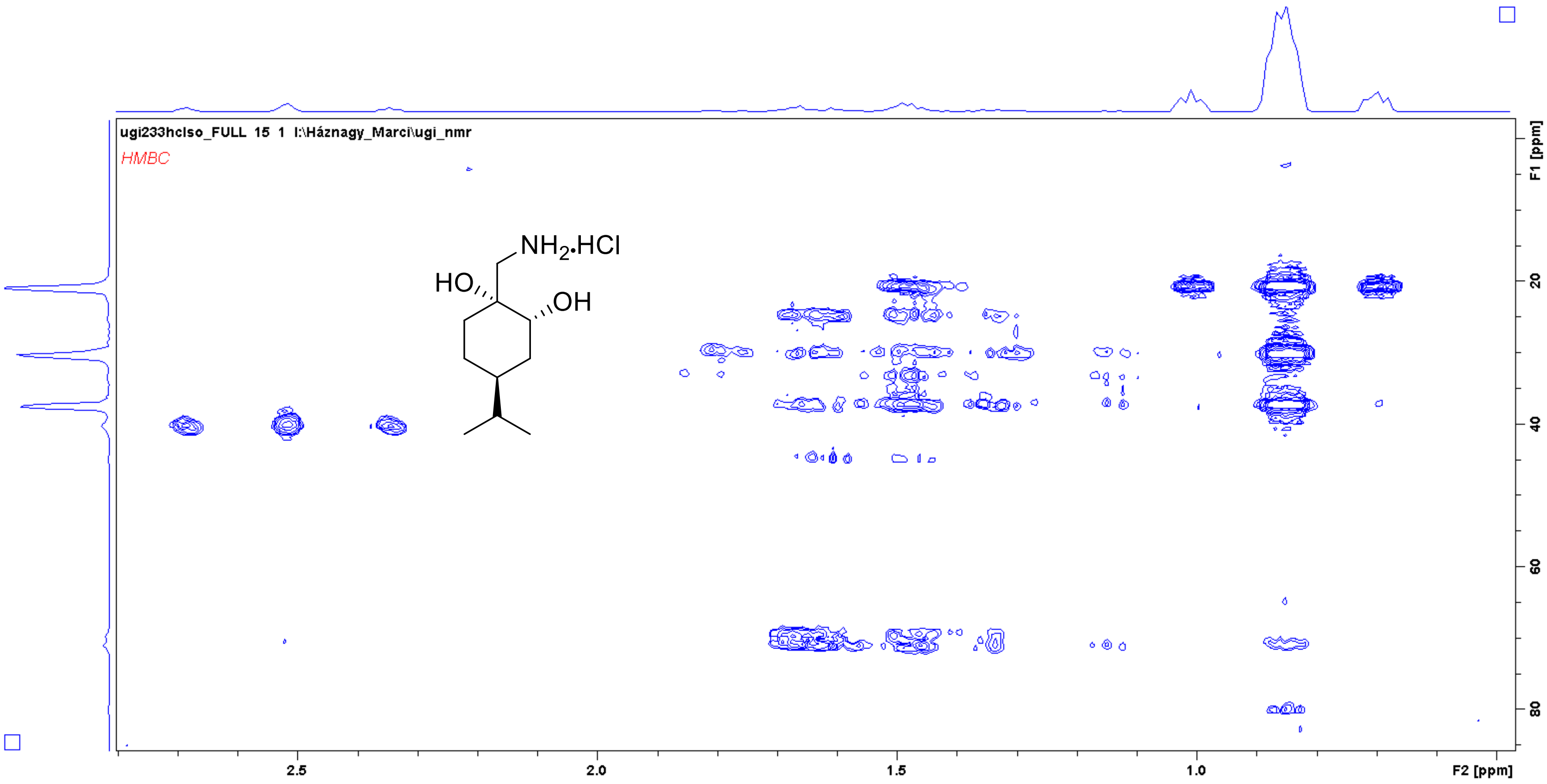

Figure S 72: ^{1}H-NMR of compound (1*S*,2*S*,4*S*)-1-((Benzyl(methyl)amino)methyl)-4-isopropylcyclohexane-1,2-diol **9**

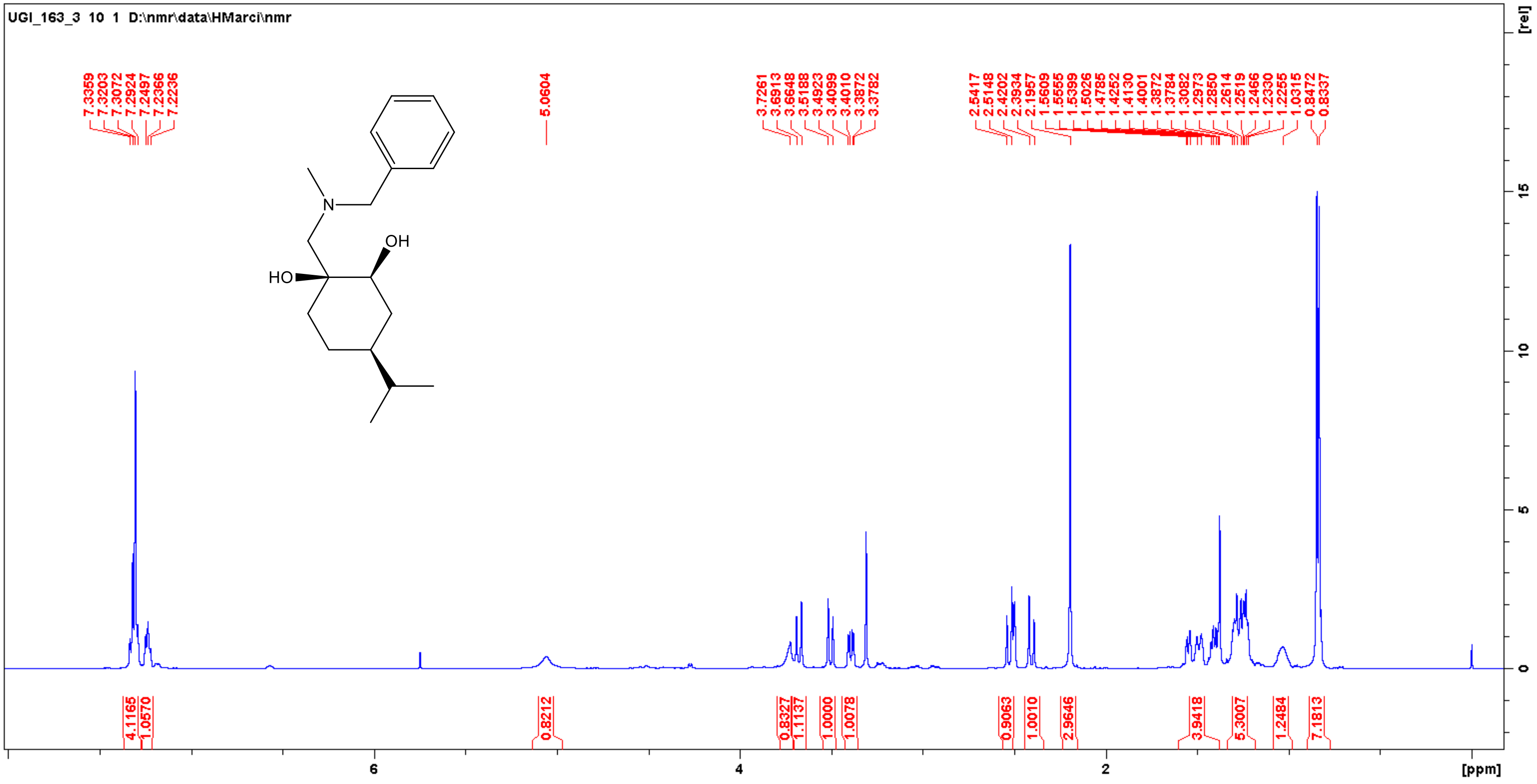

Figure S 73: ^{13}C-NMR of compound (1*S*,2*S*,4*S*)-1-((Benzyl(methyl)amino)methyl)-4-isopropylcyclohexane-1,2-diol **9**

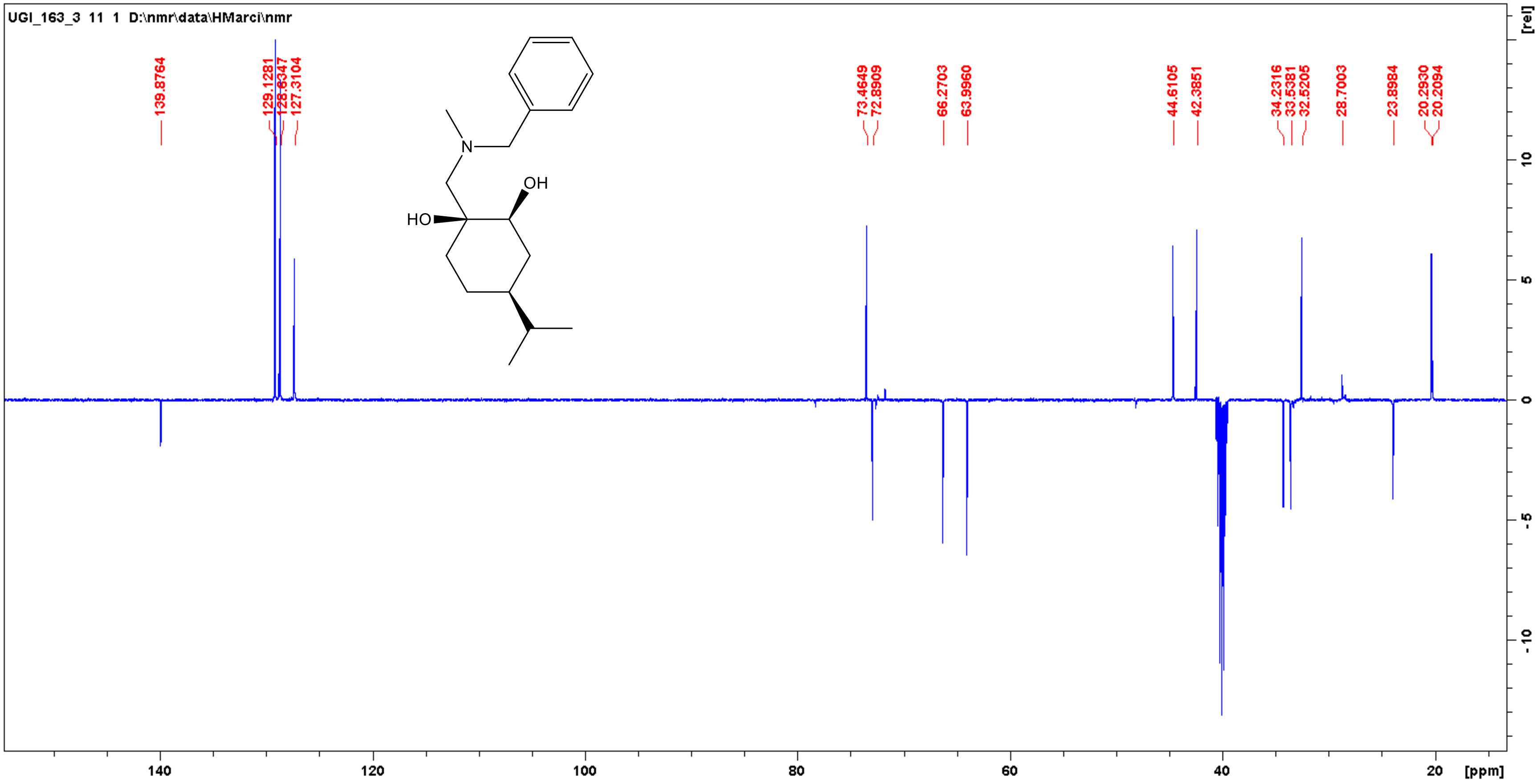

Figure S 74: ^{1}H-NMR of compound (1*R*,2*R*,4*S*)-1-((Benzyl(methyl)amino)methyl)-4-isopropylcyclohexane-1,2-diol **13**

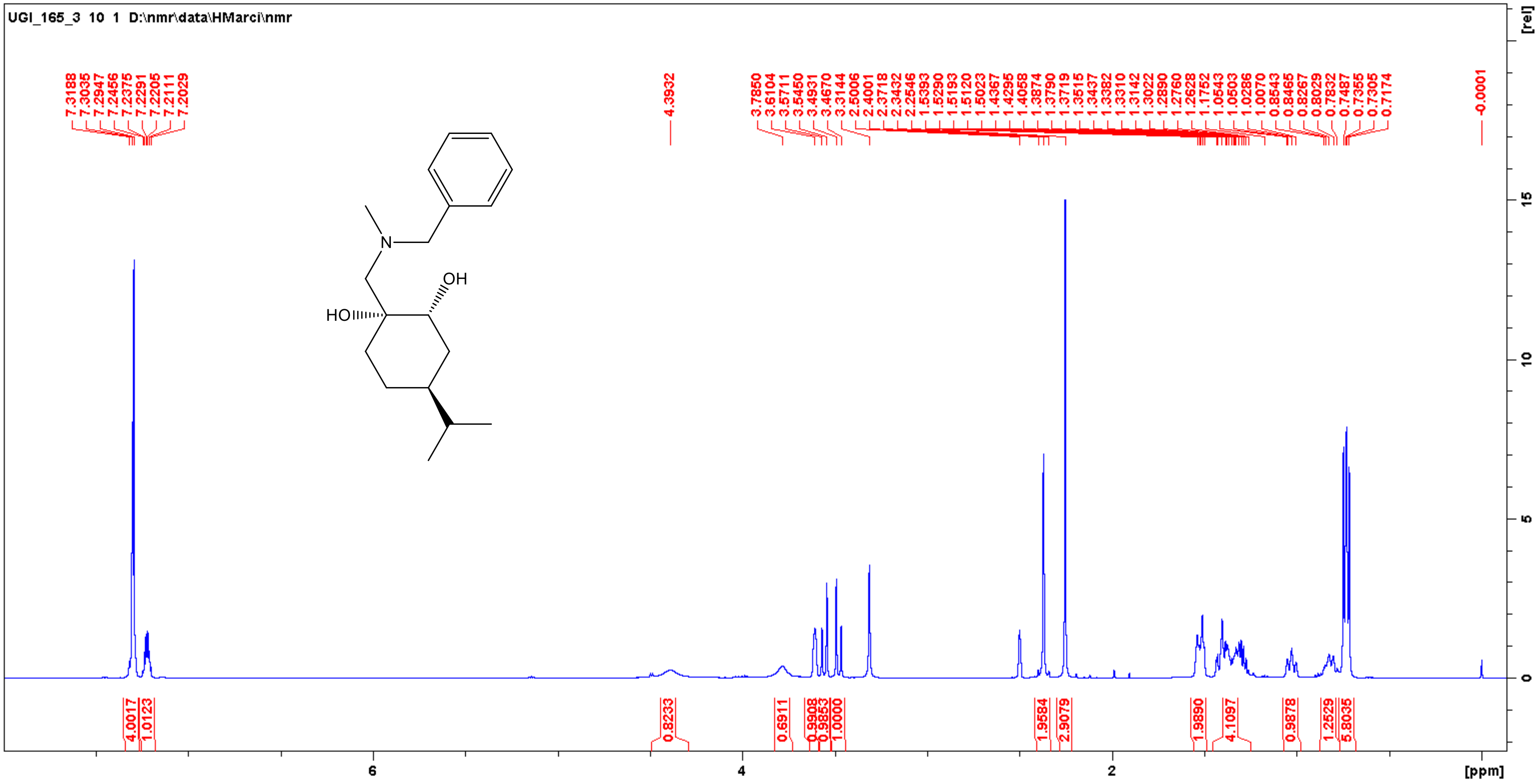

Figure S 75: ^{13}C-NMR of compound (1*R*,2*R*,4*S*)-1-((Benzyl(methyl)amino)methyl)-4-isopropylcyclohexane-1,2-diol **13**

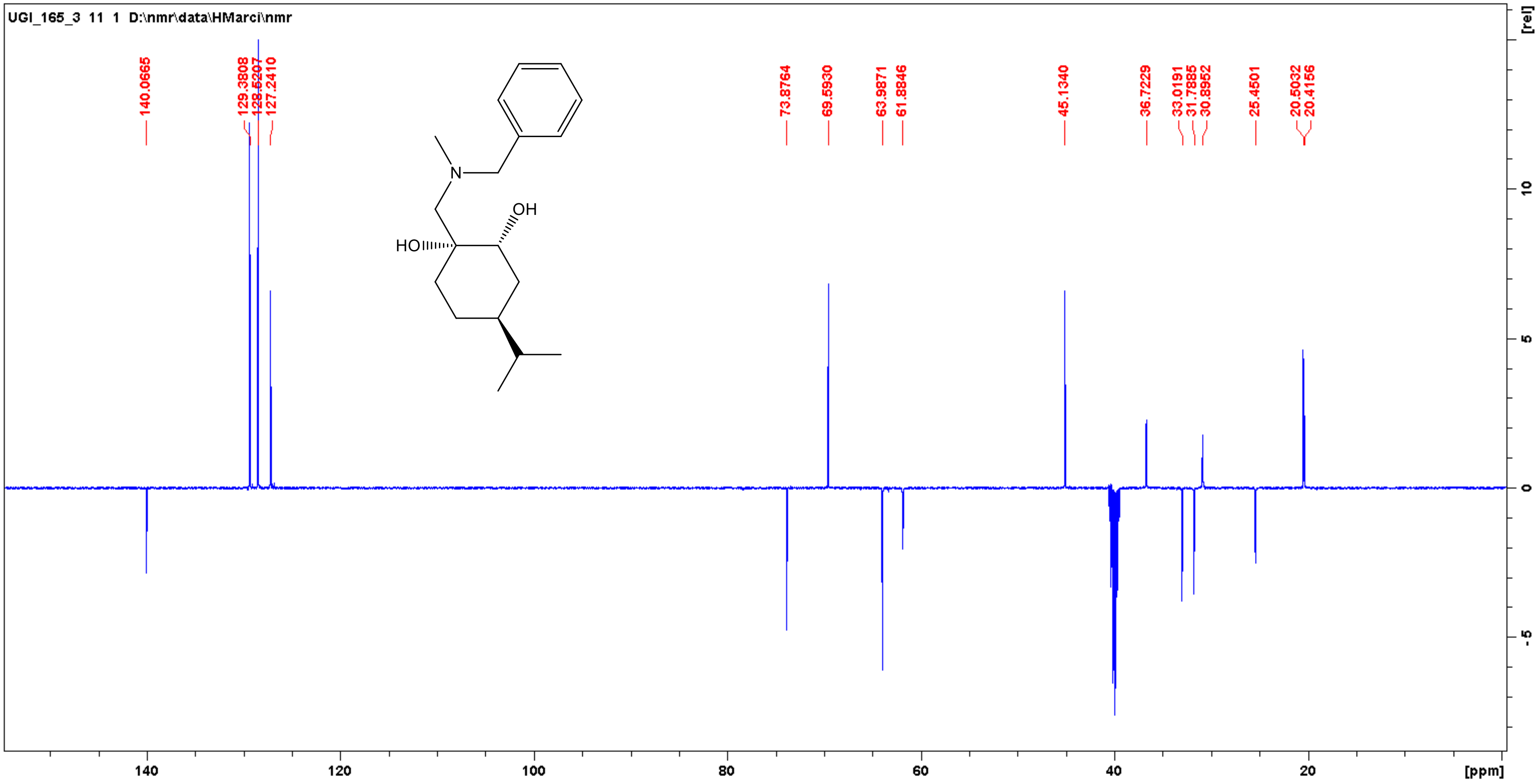

Figure S 76: ¹H-NMR of compound (4a*S*,7*S*,8a*S*)-3-Benzyl-7-isopropyloctahydro-2*H*-benzo[*e*][1,3]oxazin-4a-ol **10a**

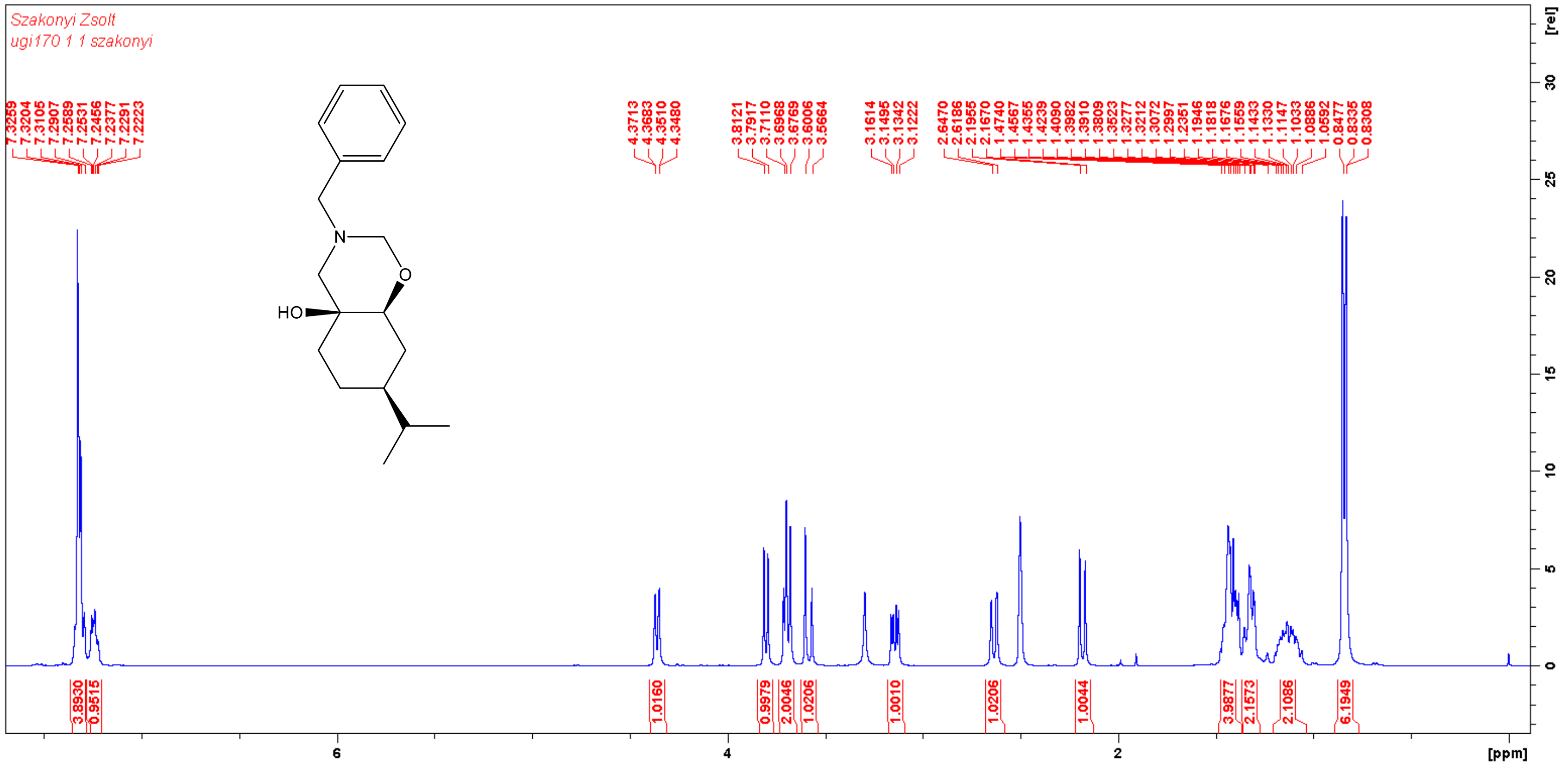

Figure S 77: ^{13}C-NMR of compound (4a*S*,7*S*,8a*S*)-3-Benzyl-7-isopropyloctahydro-2*H*-benzo[*e*][1,3]oxazin-4a-ol **10a**

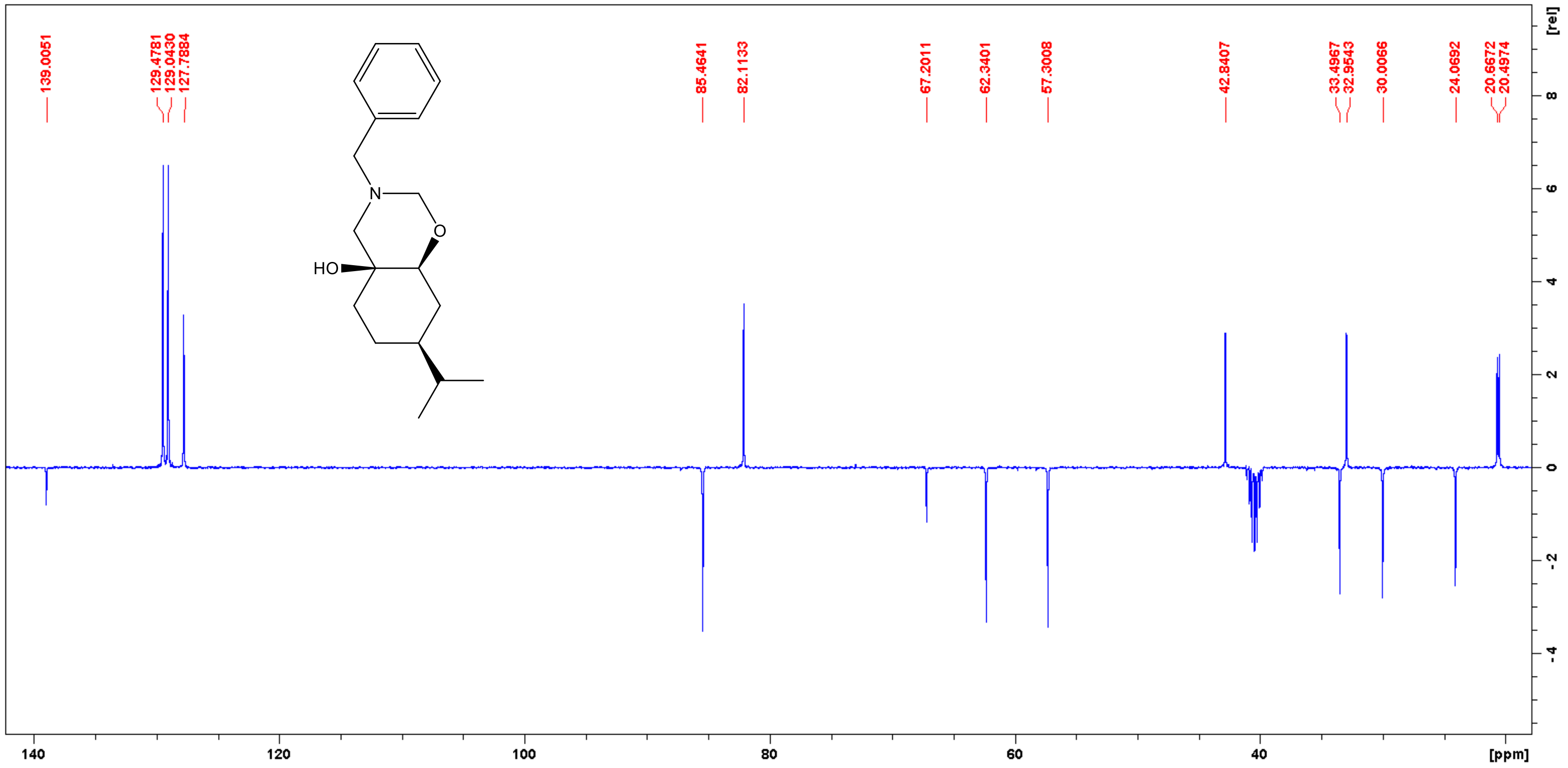

Figure S 78: COSY NMR of compound (4a*S*,7*S*,8a*S*)-3-Benzyl-7-isopropyloctahydro-2*H*-benzo[*e*][1,3]oxazin-4a-ol **10a**

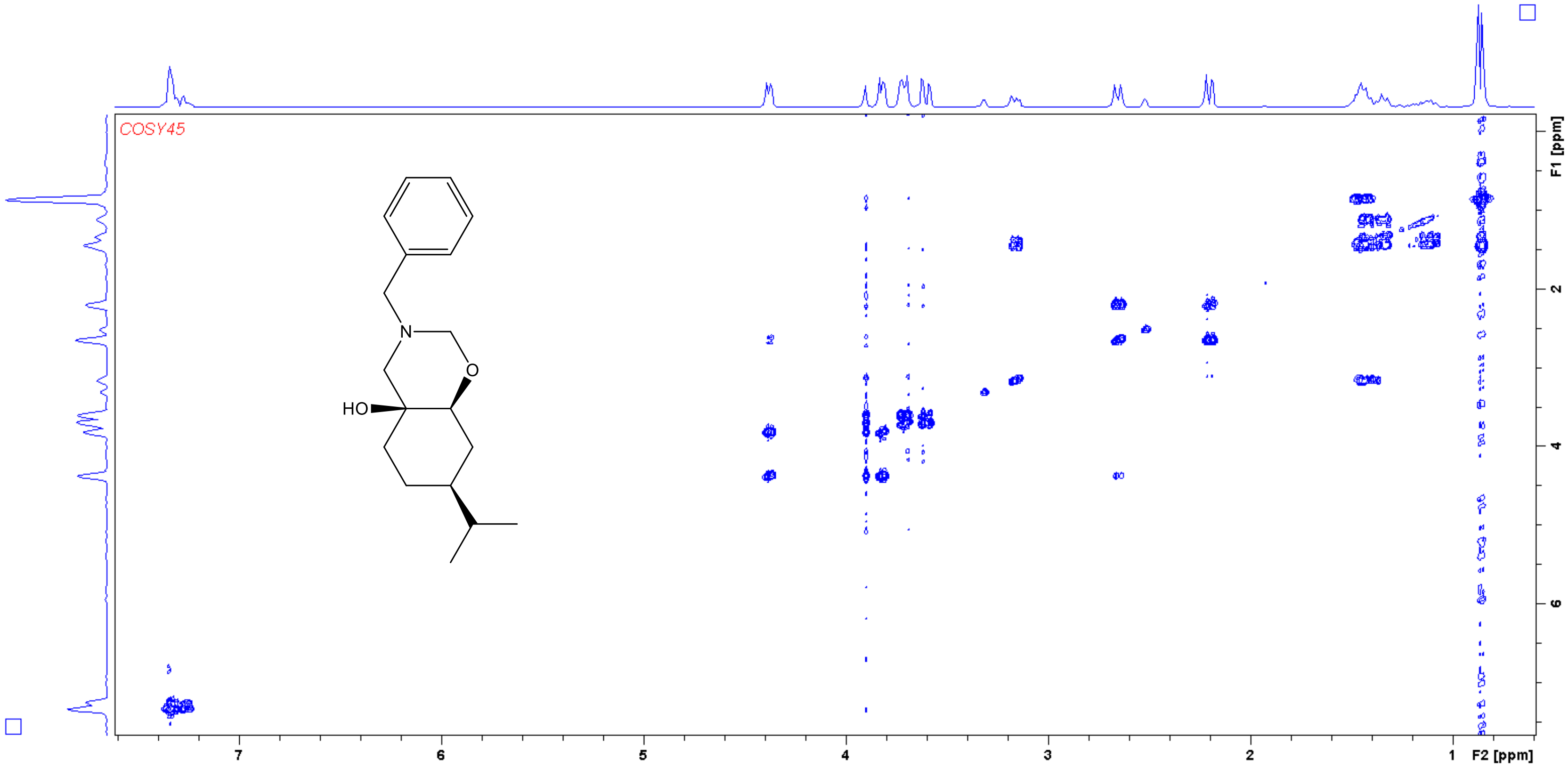

Figure S 79: NOESY NMR of compound (4a*S*,7*S*,8a*S*)-3-Benzyl-7-isopropyloctahydro-2*H*-benzo[*e*][1,3]oxazin-4a-ol **10a**

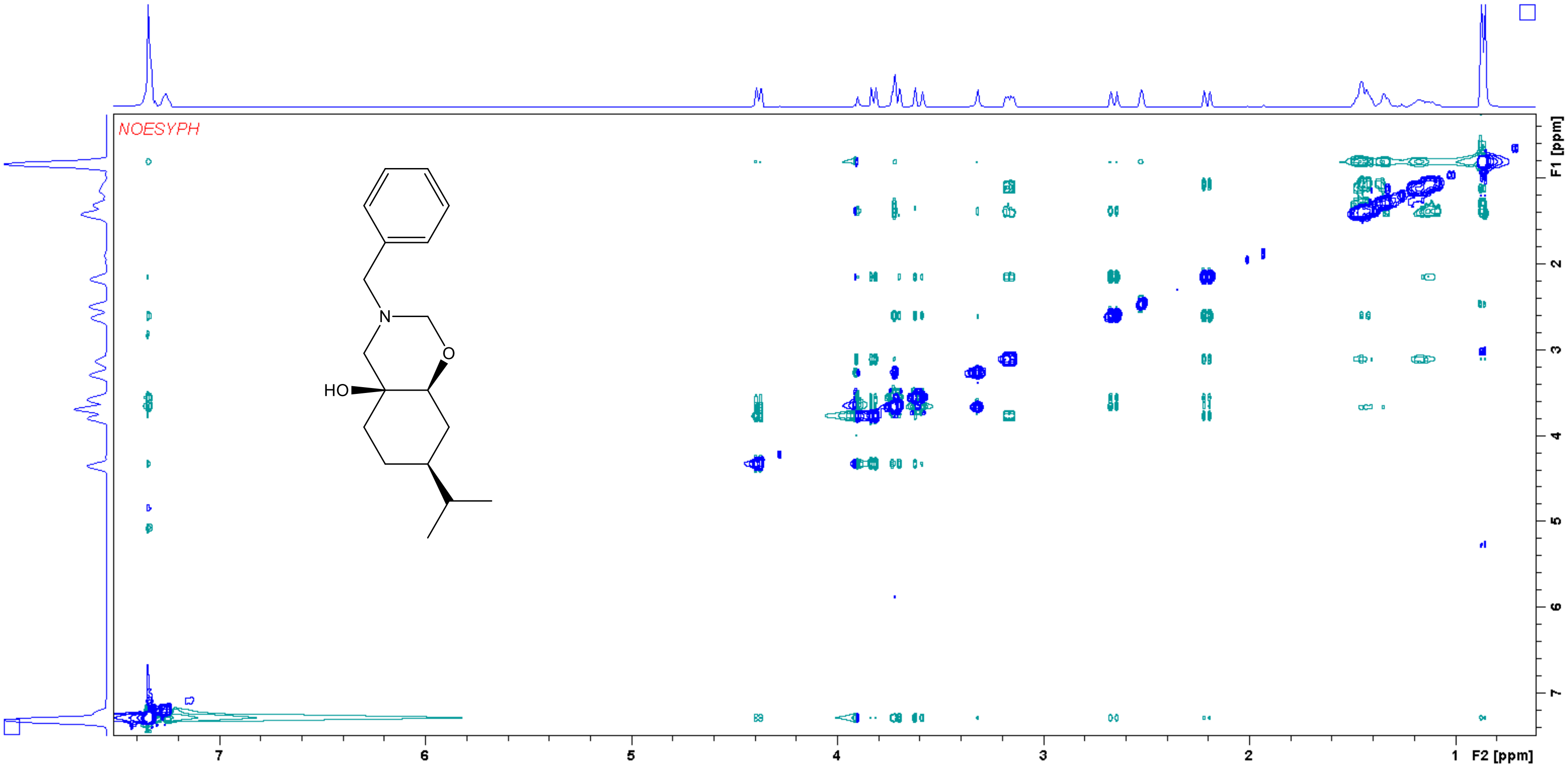

Figure S 80: HSQC NMR of compound (4a*S*,7*S*,8a*S*)-3-Benzyl-7-isopropyloctahydro-2*H*-benzo[*e*][1,3]oxazin-4a-ol **10a**

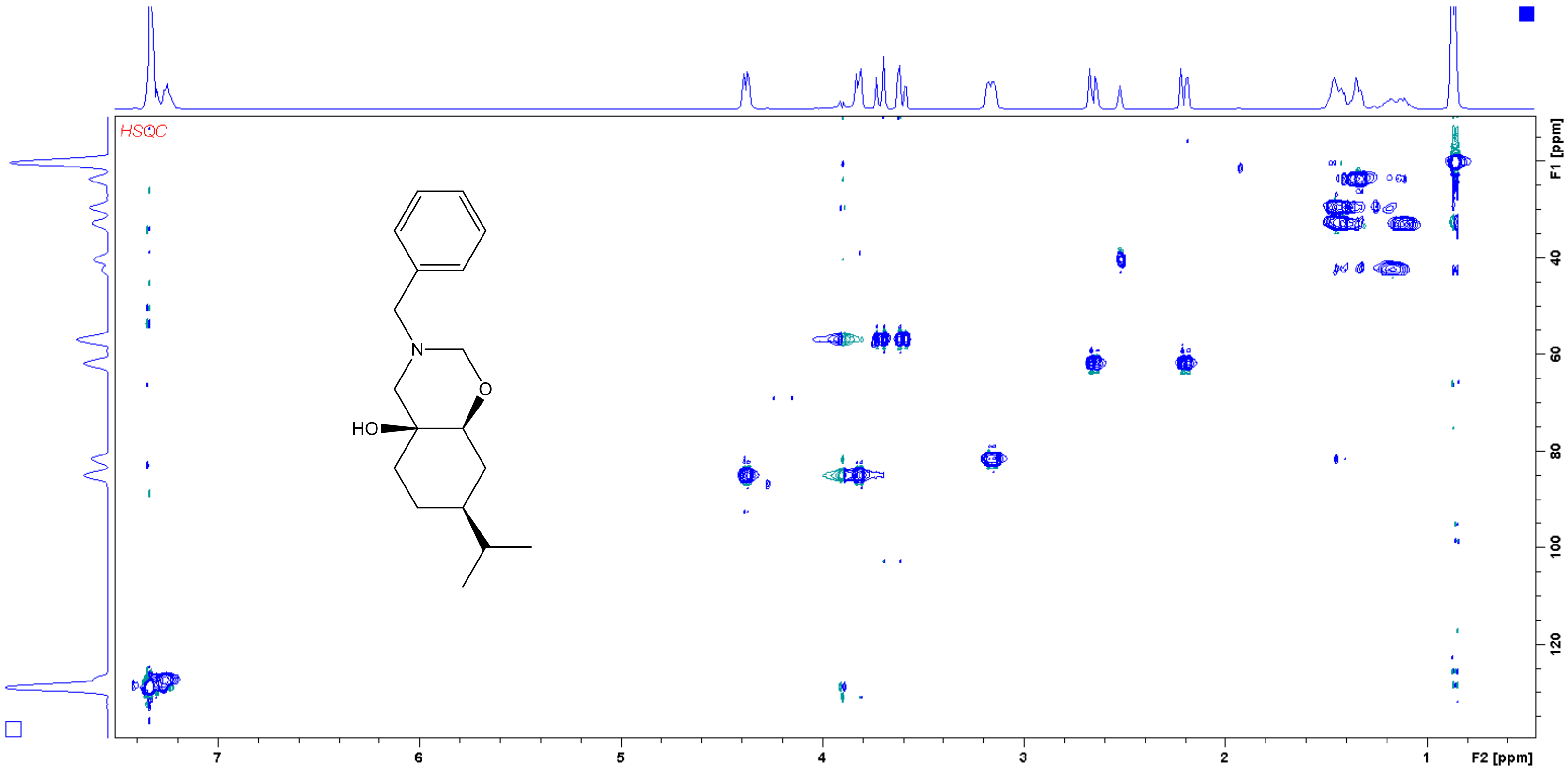

Figure S 81: HMBC NMR of compound (4a*S*,7*S*,8a*S*)-3-Benzyl-7-isopropyloctahydro-2*H*-benzo[*e*][1,3]oxazin-4a-ol **10a**

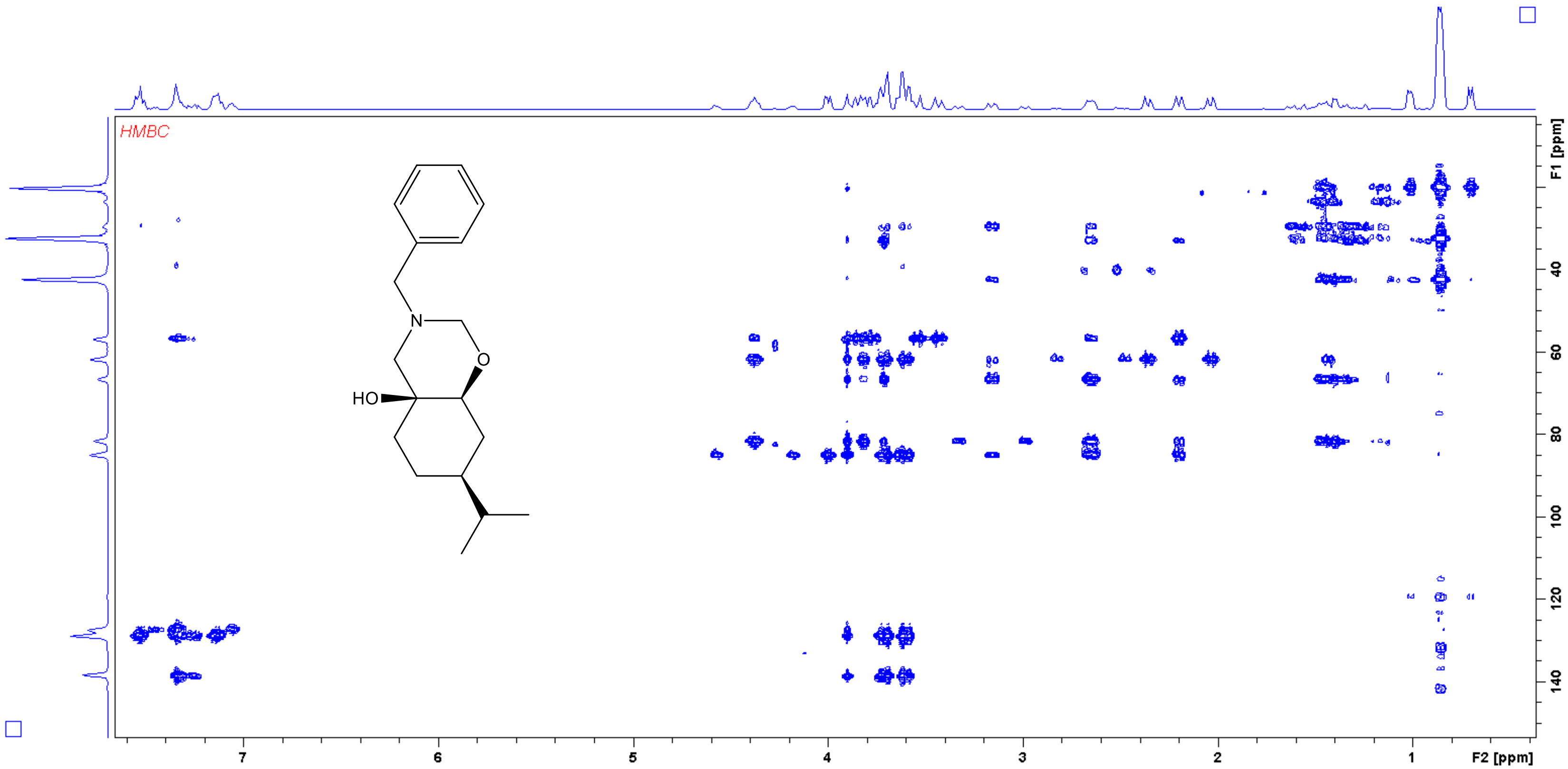

Figure S 82: ^{1}H-NMR of compound (4a*S*,7*S*,8a*S*)-7-Isopropyl-3-((*S*)-1-phenylethyl)octahydro-2*H*-benzo[*e*][1,3]oxazin-4a-ol **10b**

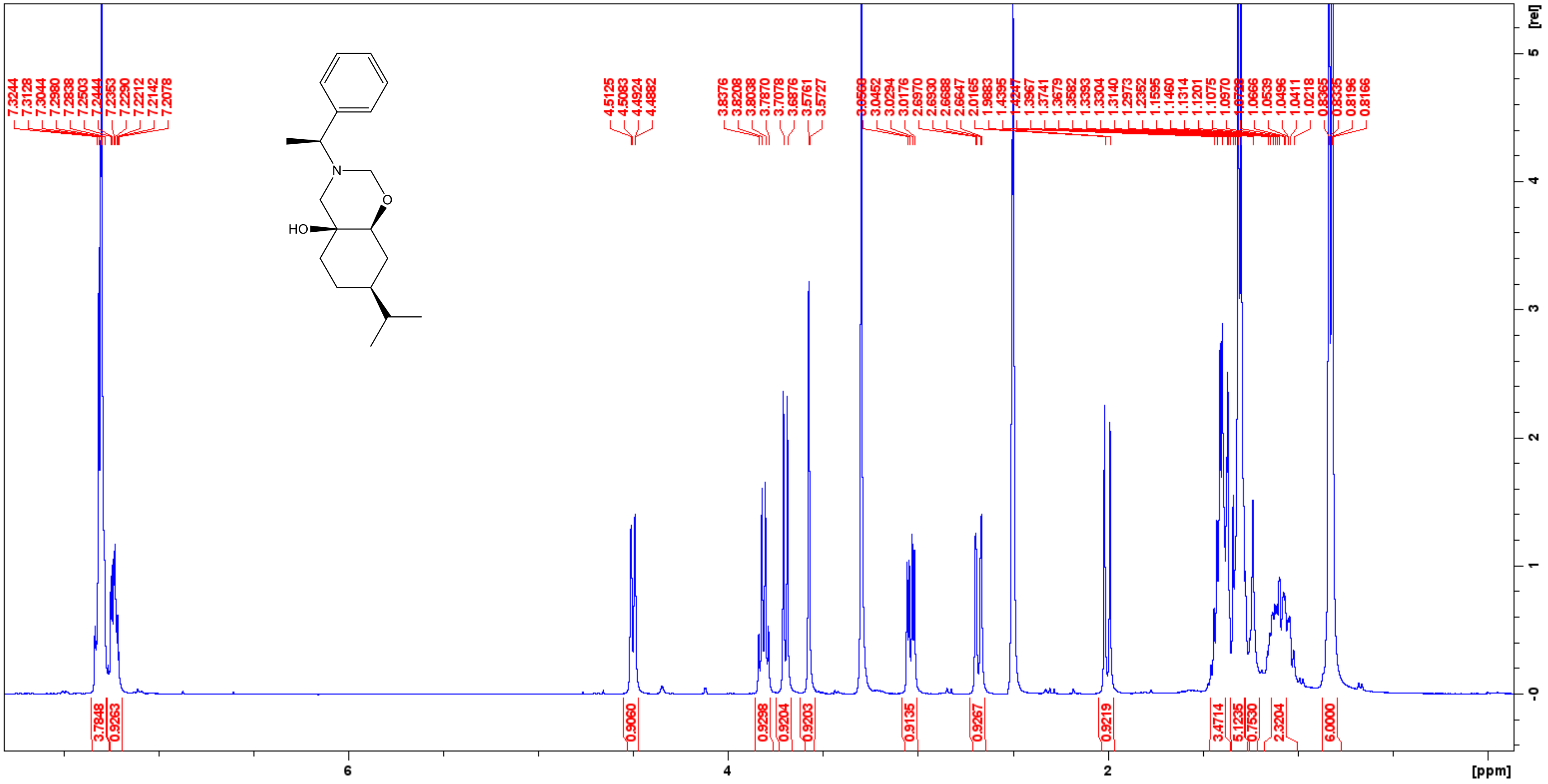

Figure S 83: ^{13}C-NMR of compound (4a*S*,7*S*,8a*S*)-7-Isopropyl-3-((*S*)-1-phenylethyl)octahydro-2*H*-benzo[*e*][1,3]oxazin-4a-ol **10b**

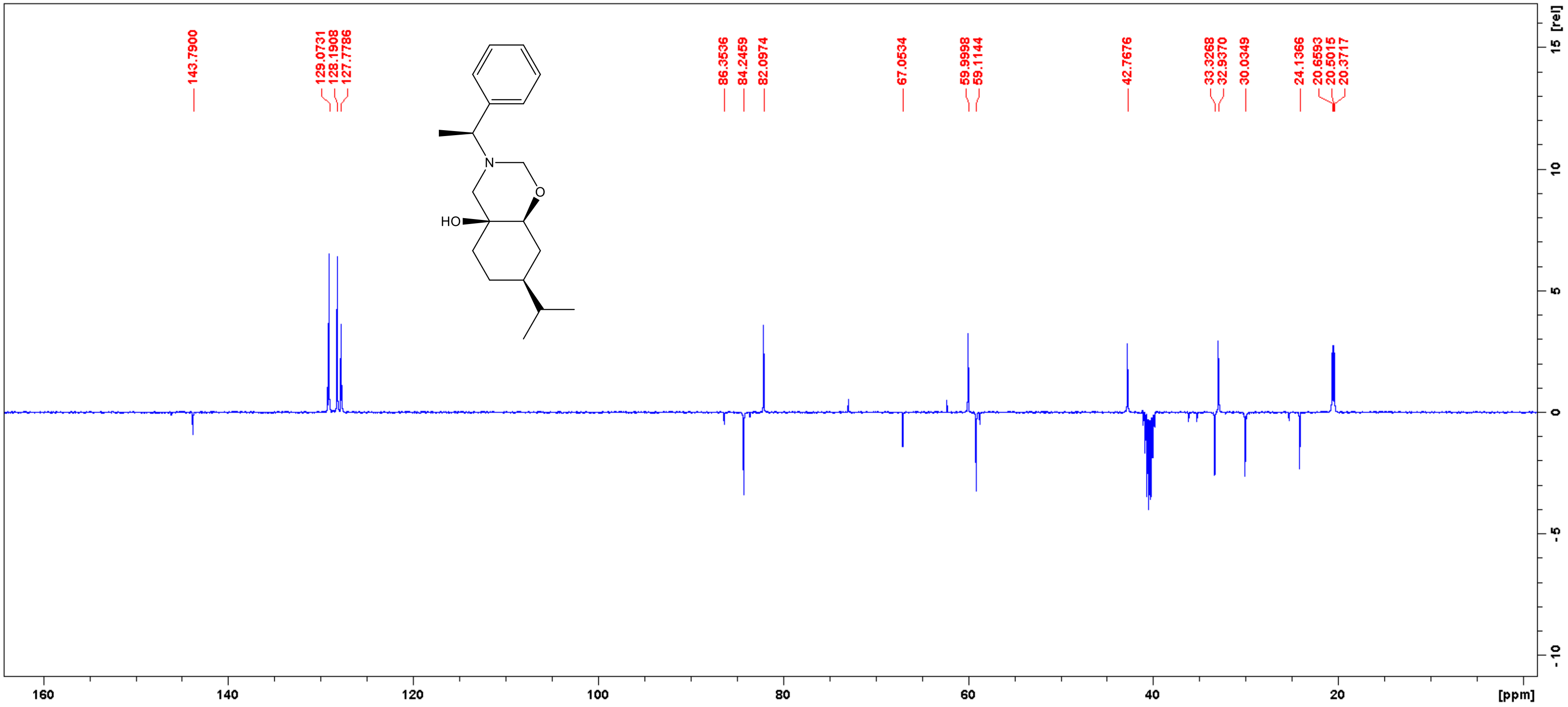

Figure S 84: COSY NMR of compound (4a*S*,7*S*,8a*S*)-7-Isopropyl-3-((*S*)-1-phenylethyl)octahydro-2*H*-benzo[*e*][1,3]oxazin-4a-ol **10b**

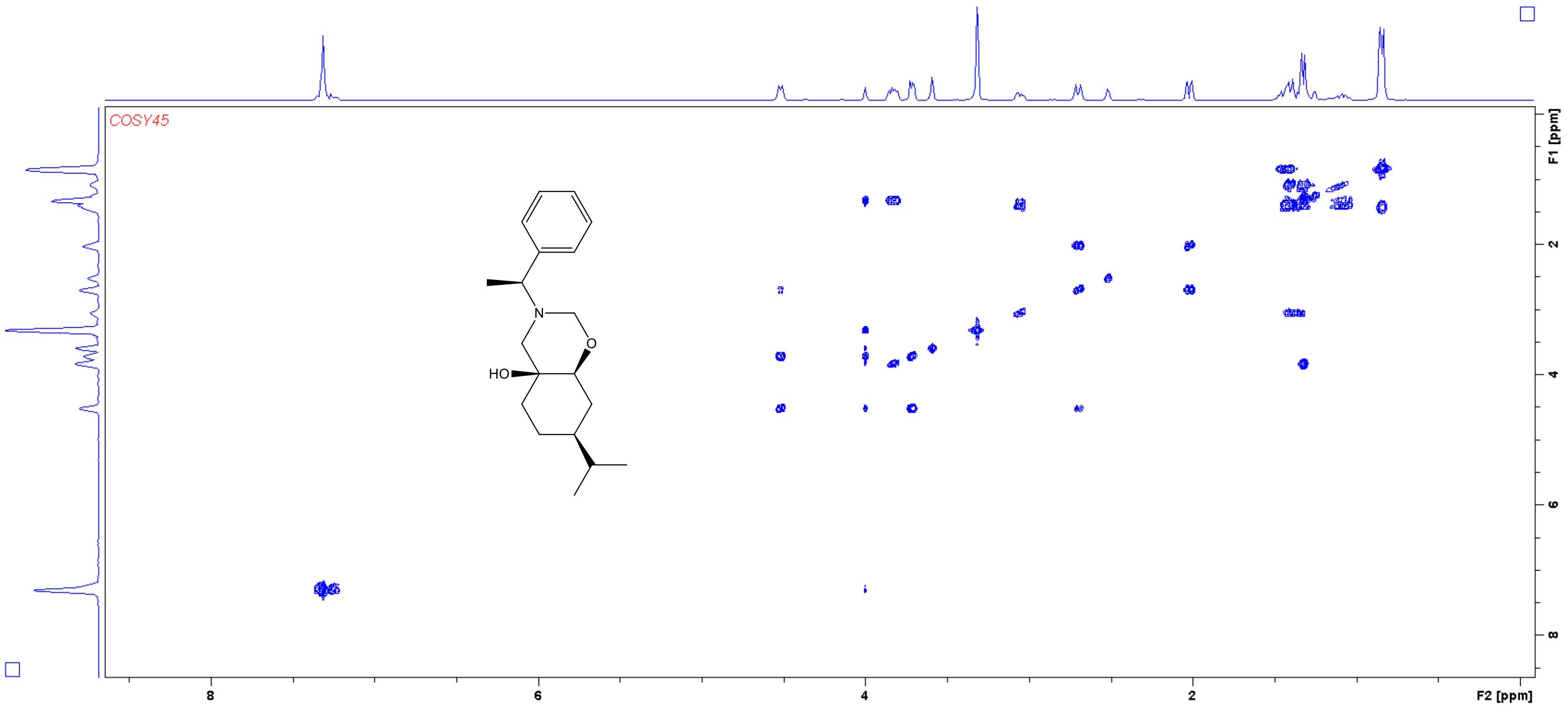

Figure S 85: HSQC NMR of compound (4a*S*,7*S*,8a*S*)-7-Isopropyl-3-((*S*)-1-phenylethyl)octahydro-2*H*-benzo[*e*][1,3]oxazin-4a-ol **10b**

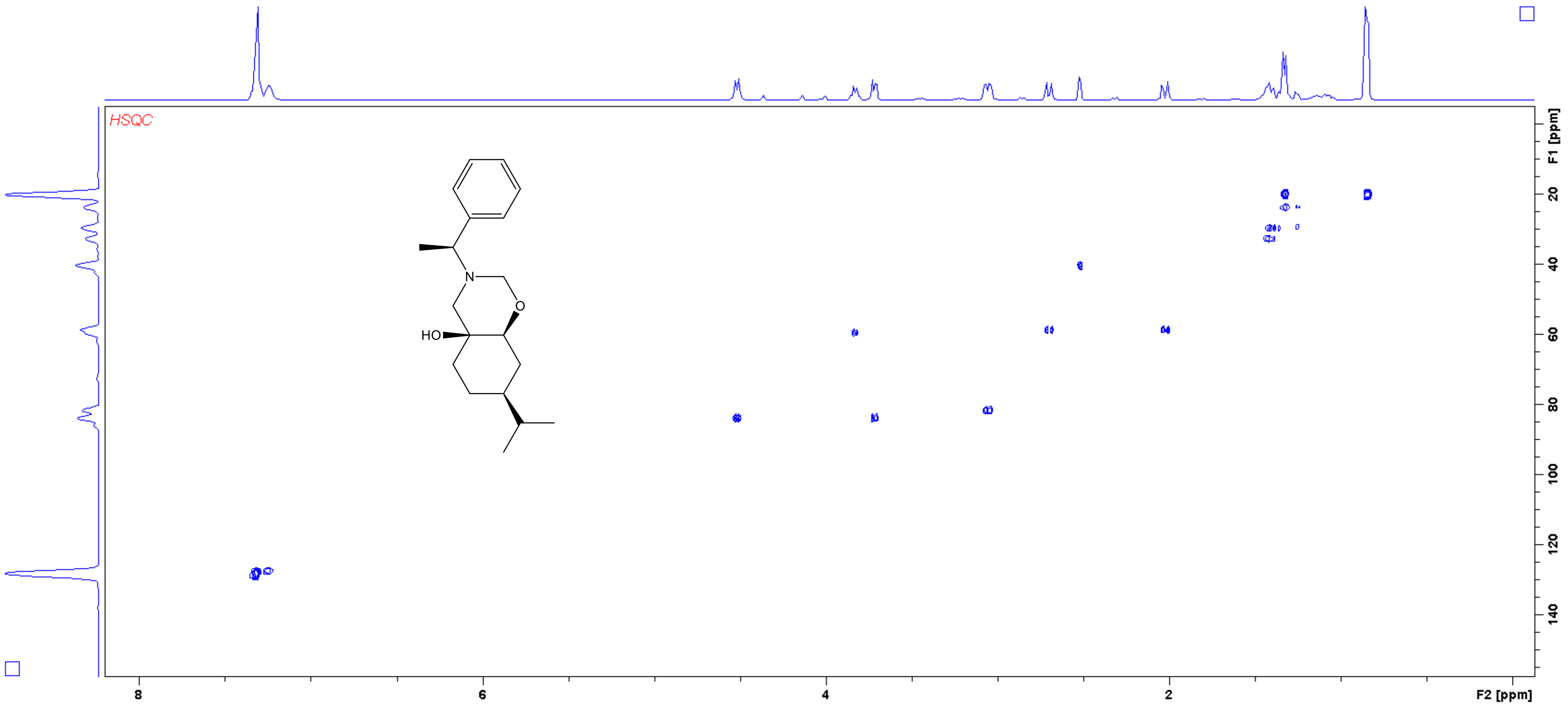

Figure S 86: HMBC NMR of compound (4a*S*,7*S*,8a*S*)-7-Isopropyl-3-((*S*)-1-phenylethyl)octahydro-2*H*-benzo[*e*][1,3]oxazin-4a-ol **10b**

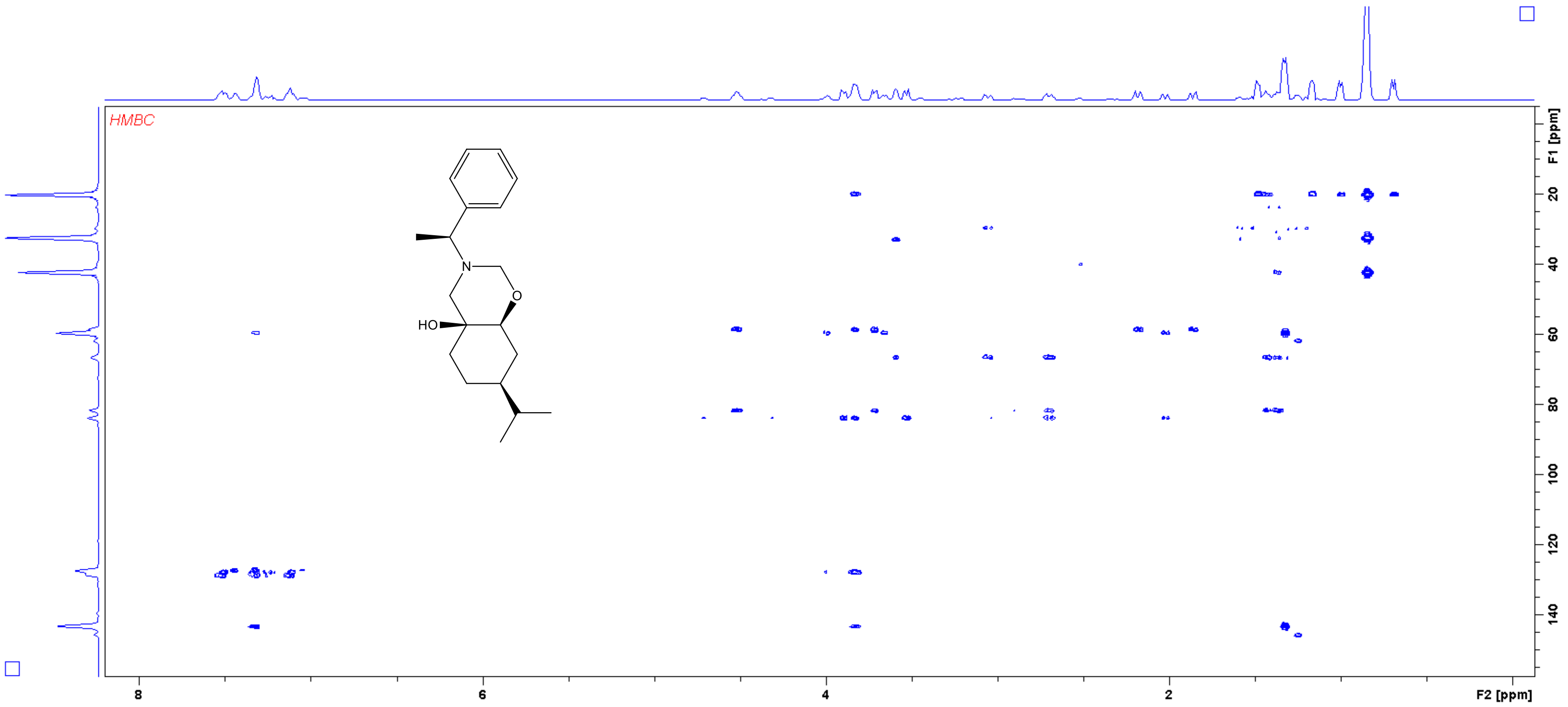

Figure S 87: ^{1}H-NMR of compound (4a*S*,7*S*,8a*S*)-7-Isopropyl-3-((*R*)-1-phenylethyl)octahydro-2*H*-benzo[*e*][1,3]oxazin-4a-ol **10c**

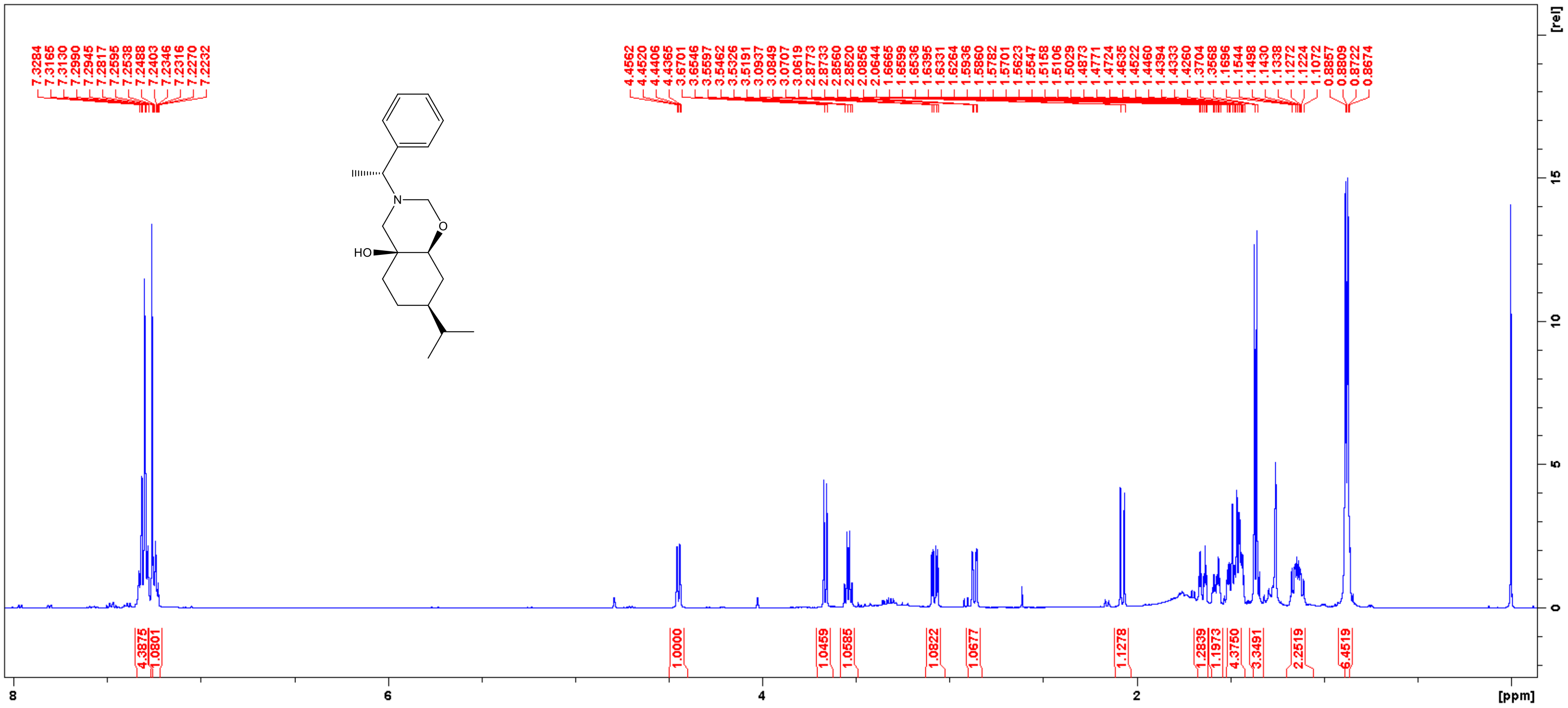

Figure S 88: ^{13}C-NMR of compound (4a*S*,7*S*,8a*S*)-7-Isopropyl-3-((*R*)-1-phenylethyl)octahydro-2*H*-benzo[*e*][1,3]oxazin-4a-ol **10c**

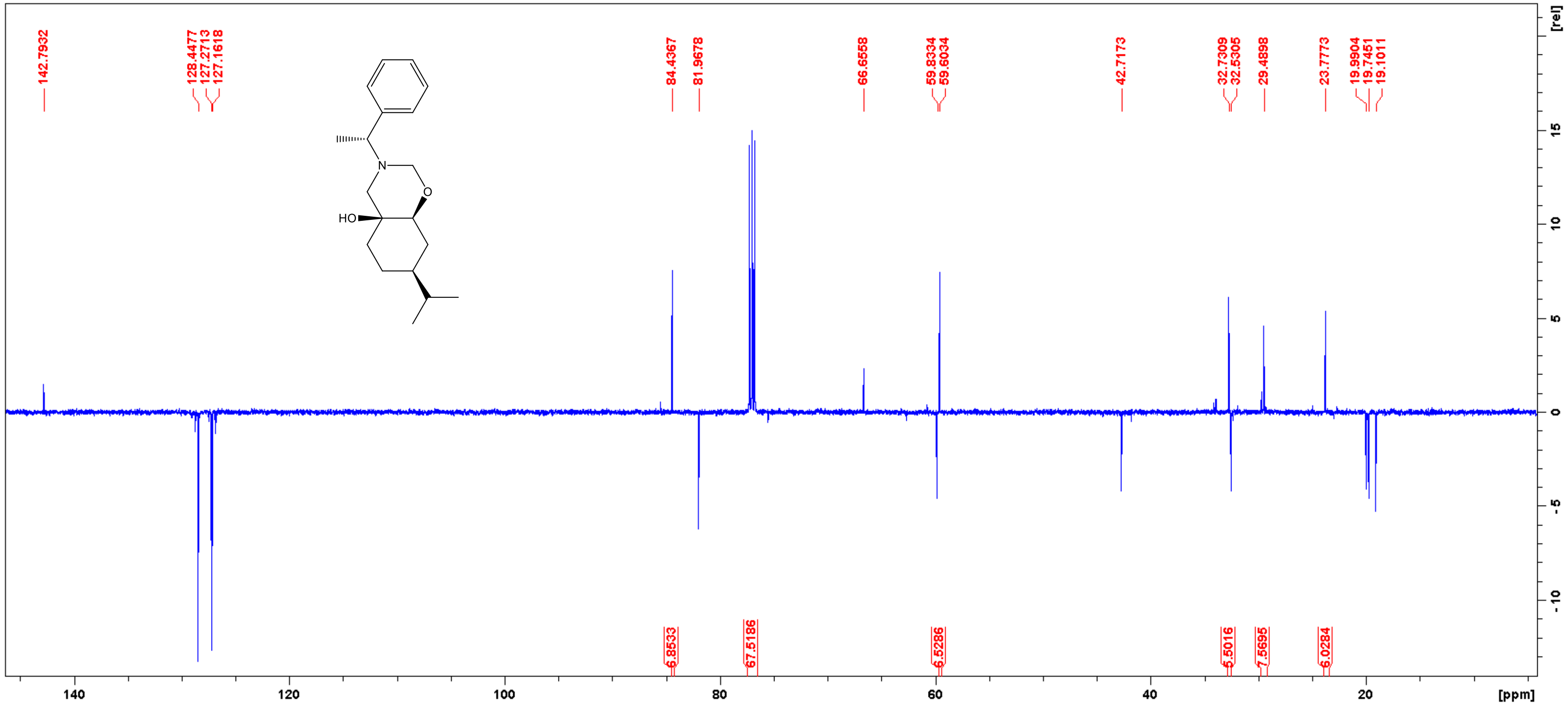

Figure S 89: COSY NMR of compound (4a*S*,7*S*,8a*S*)-7-Isopropyl-3-((*R*)-1-phenylethyl)octahydro-2*H*-benzo[*e*][1,3]oxazin-4a-ol **10c**

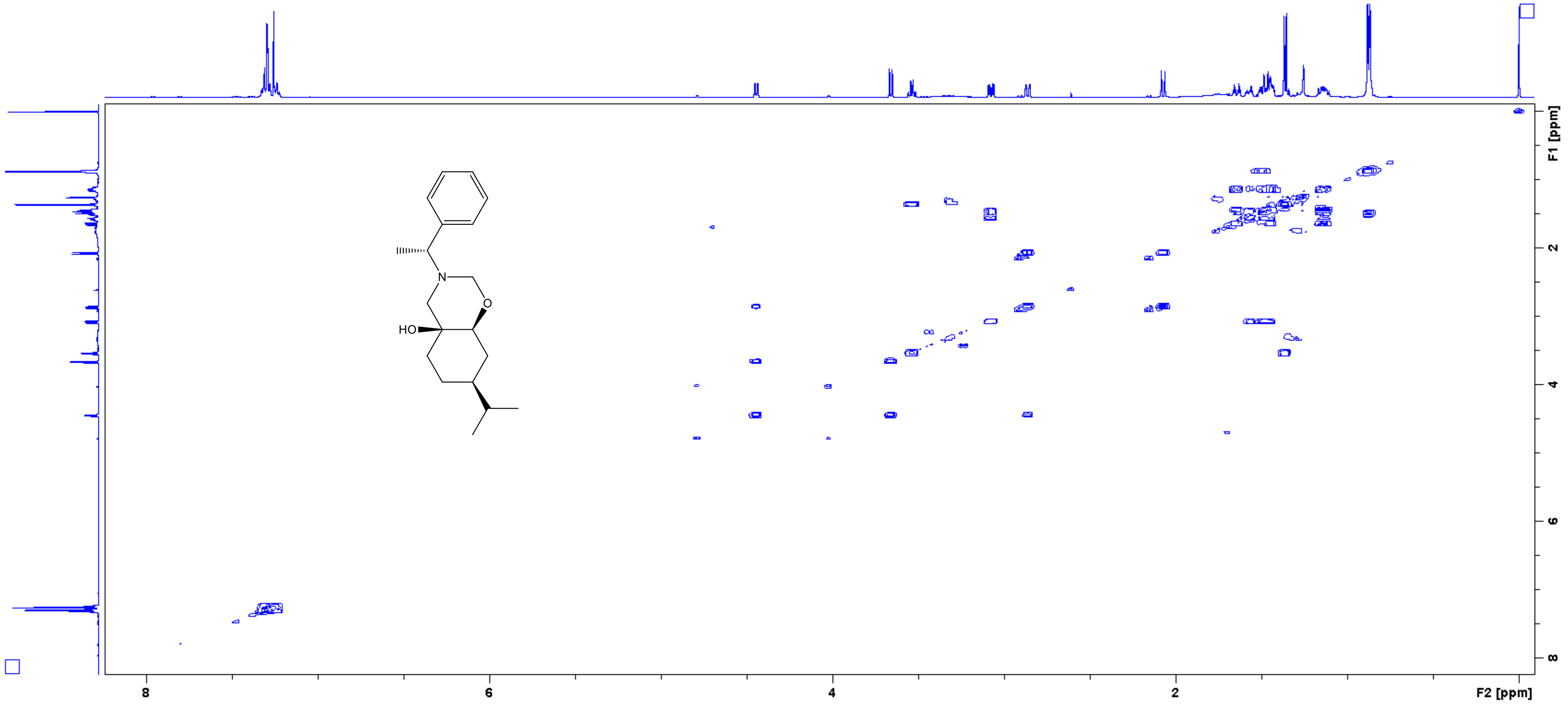

Figure S 90: HSQC NMR of compound (4a*S*,7*S*,8a*S*)-7-Isopropyl-3-((*R*)-1-phenylethyl)octahydro-2*H*-benzo[*e*][1,3]oxazin-4a-ol **10c**

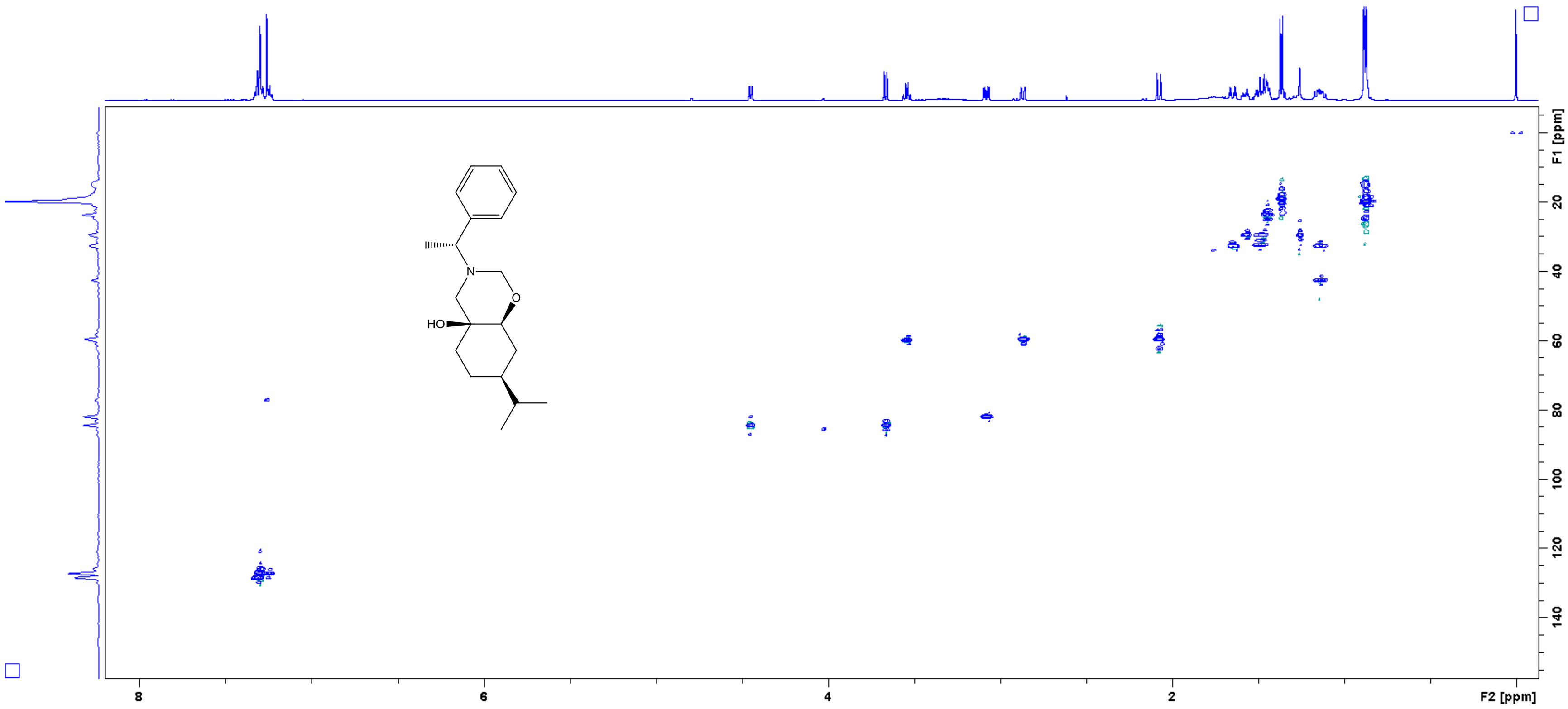

Figure S 91: HMBC NMR of compound (4a*S*,7*S*,8a*S*)-7-Isopropyl-3-((*R*)-1-phenylethyl)octahydro-2*H*-benzo[*e*][1,3]oxazin-4a-ol **10c**

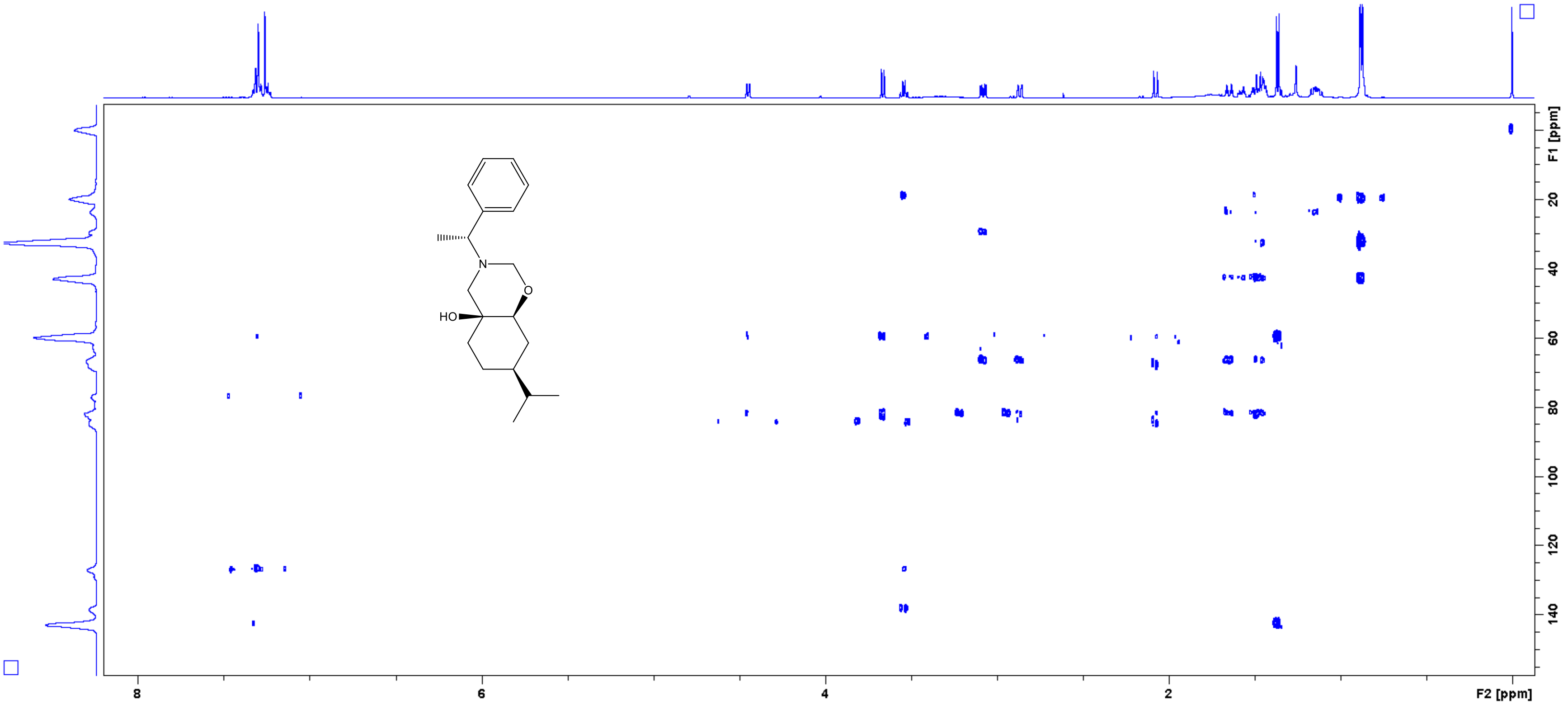

Figure S 92: ^{1}H-NMR of compound 2,2,2-Trichloro-*N*-((1*R*,5*S*)-5-isopropyl-2-metilenecyclohexyl)acetamide (intermediate) **16a** and 2,2,2-trichloro-*N*-((1*S*,5*S*)-5-isopropyl-2-methylenecyclohexyl)acetamide **16b**

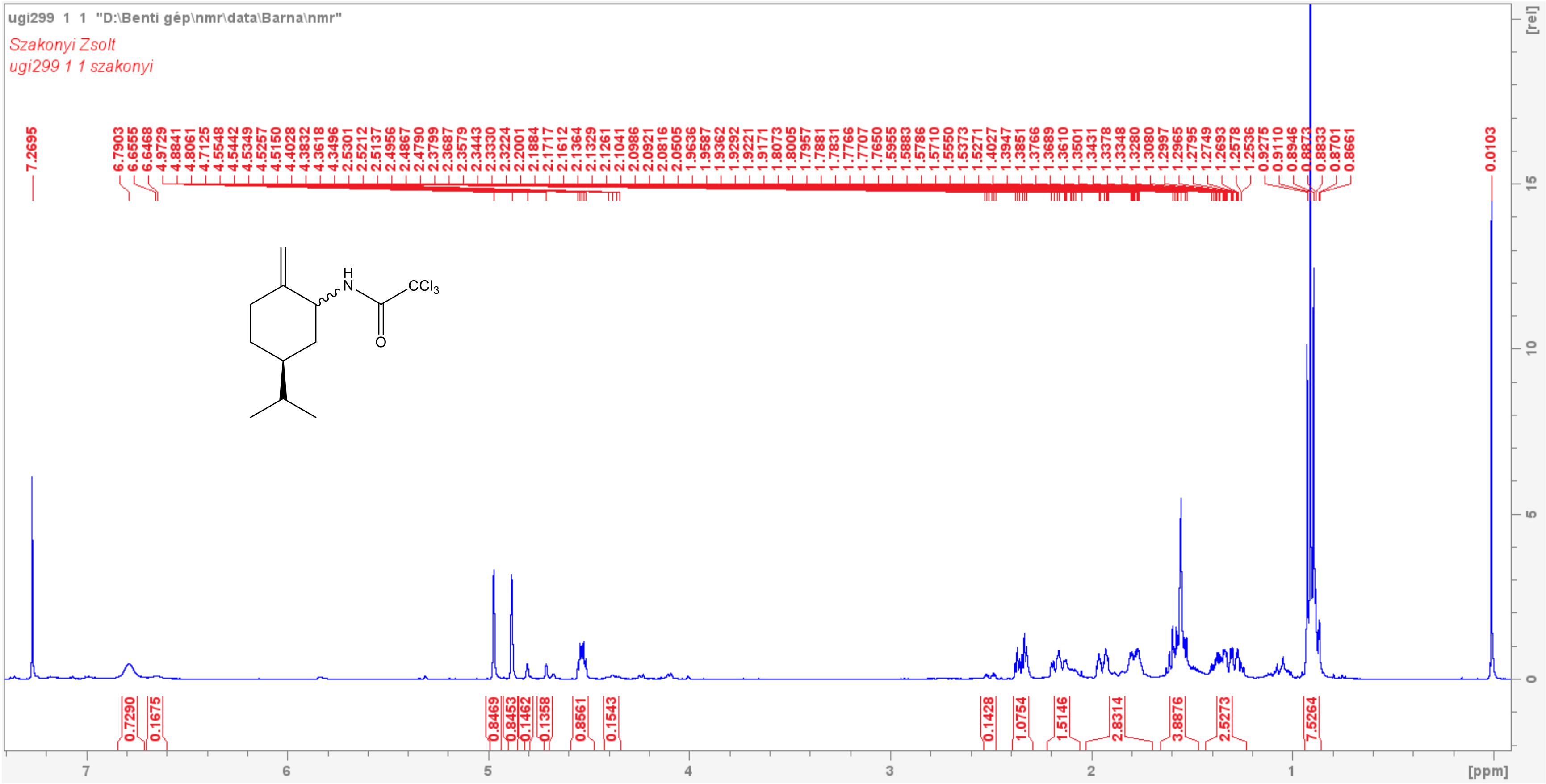

Figure S 93: ^{13}C-NMR of compound 2,2,2-Trichloro-*N*-((1*R*,5*S*)-5-isopropyl-2-metilenecyclohexyl)acetamide (intermediate) **16a** and 2,2,2-trichloro-*N*-((1*S*,5*S*)-5-isopropyl-2-methylenecyclohexyl)acetamide **16b**

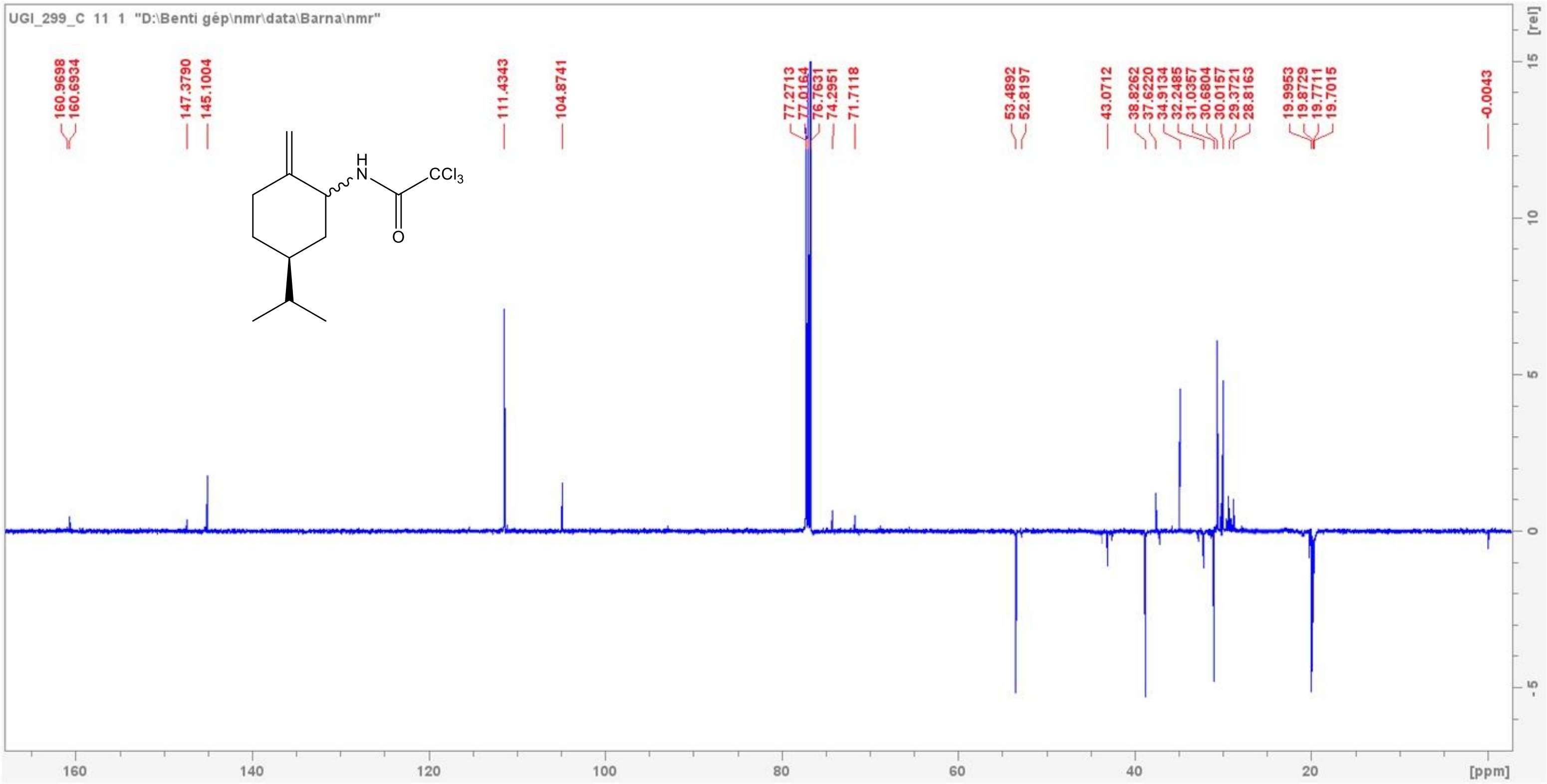

Figure S 94: ^{1}H-NMR of compound *tert*-Butyl ((1*R*,5*S*)-5-isopropyl-2-methylenecyclohexyl)carbamate **18a** and *tert*-butyl ((1*S*,5*S*)-5-isopropyl-2-methylenecyclohexyl)carbamate **18b**

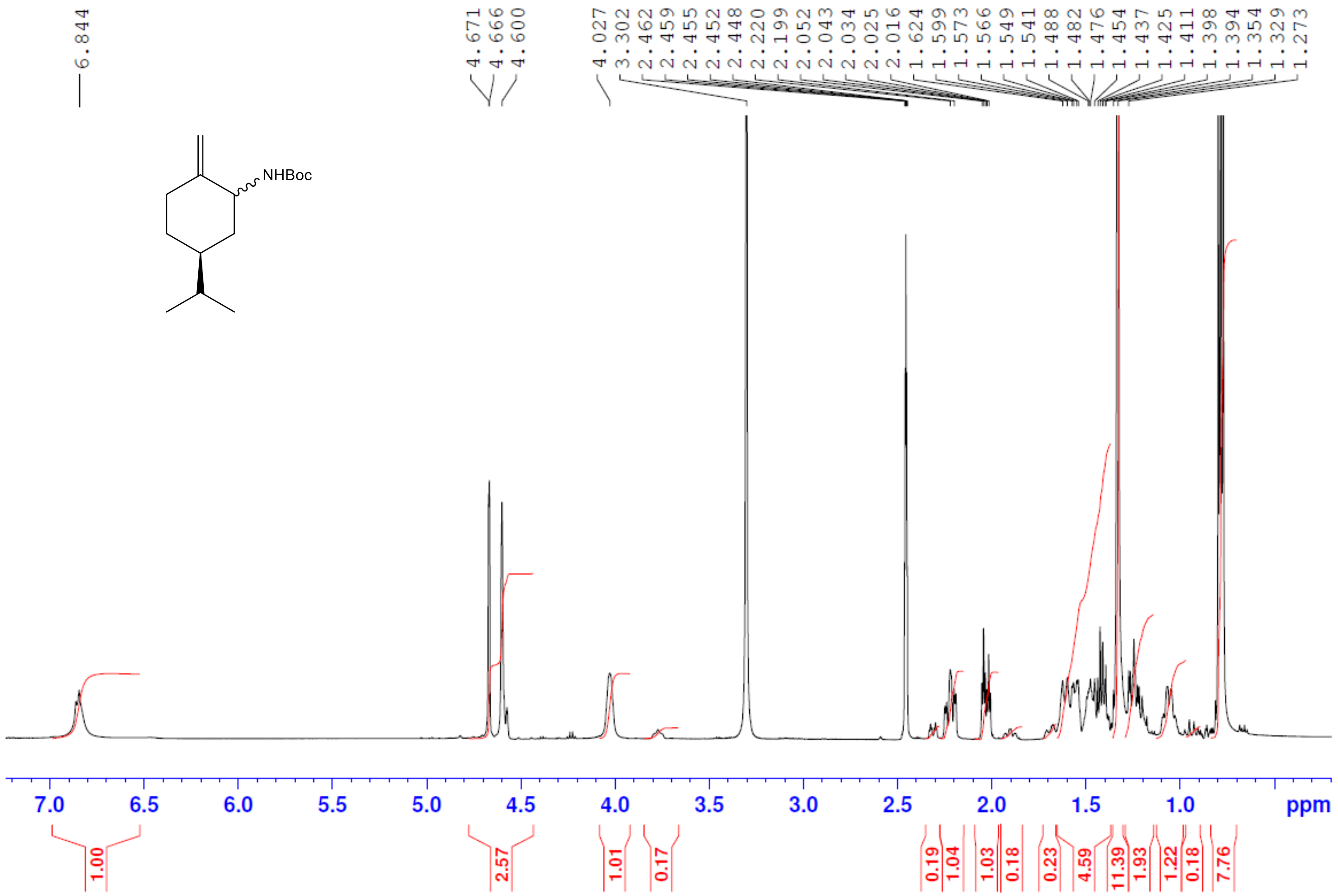

Figure S 95: **^{13}C-NMR of compound** *tert*-Butyl ((1*R*,5*S*)-5-isopropyl-2-methylenecyclohexyl)carbamate **18a** and *tert*-butyl ((1*S*,5*S*)-5-isopropyl-2-methylenecyclohexyl)carbamate **18b**

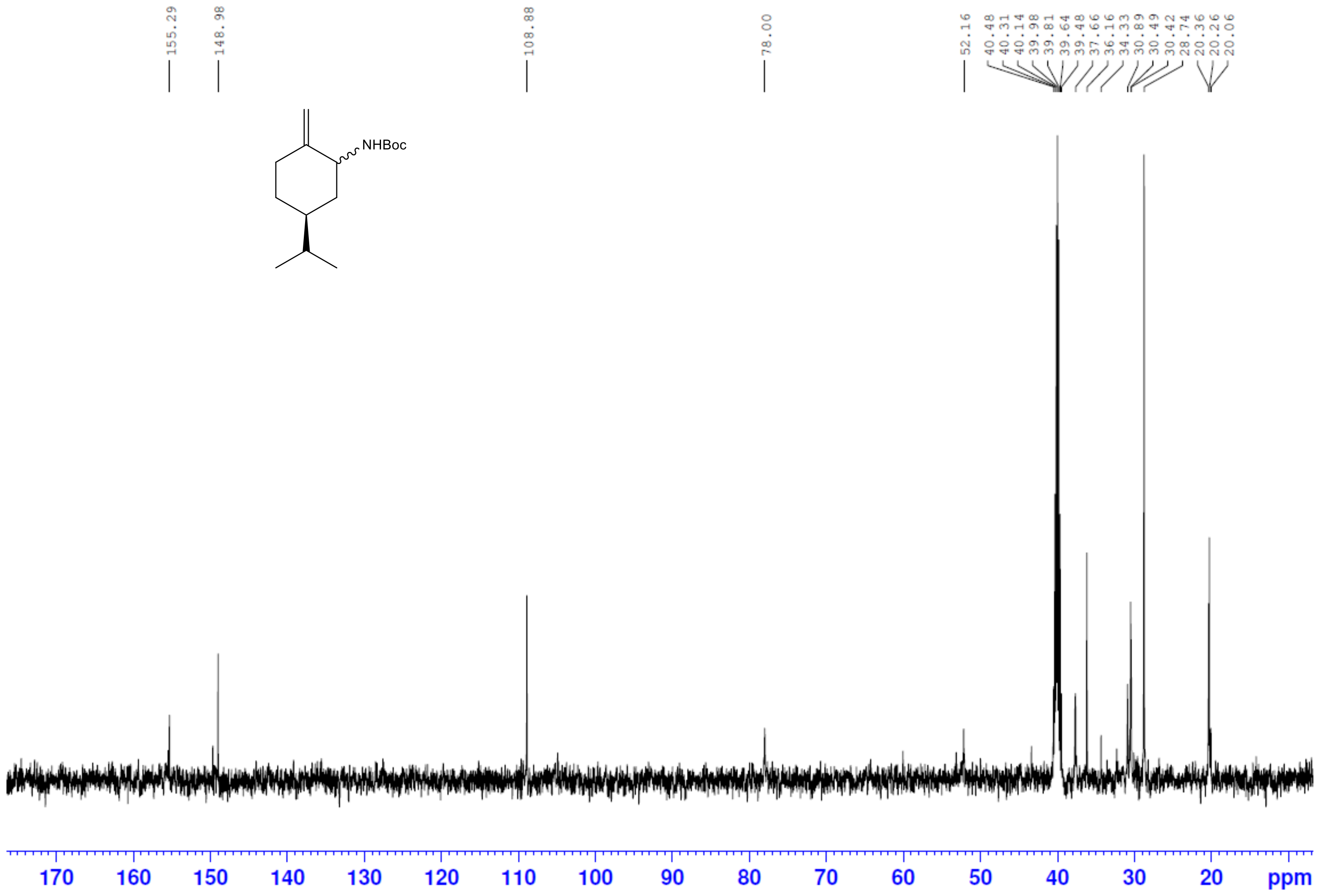

Figure S 96: NOESY-NMR of compound *tert*-Butyl ((1*R*,5*S*)-5-isopropyl-2-methylenecyclohexyl)carbamate **18a** and *tert*-butyl ((1*S*,5*S*)-5-isopropyl-2-methylenecyclohexyl)carbamate **18b**

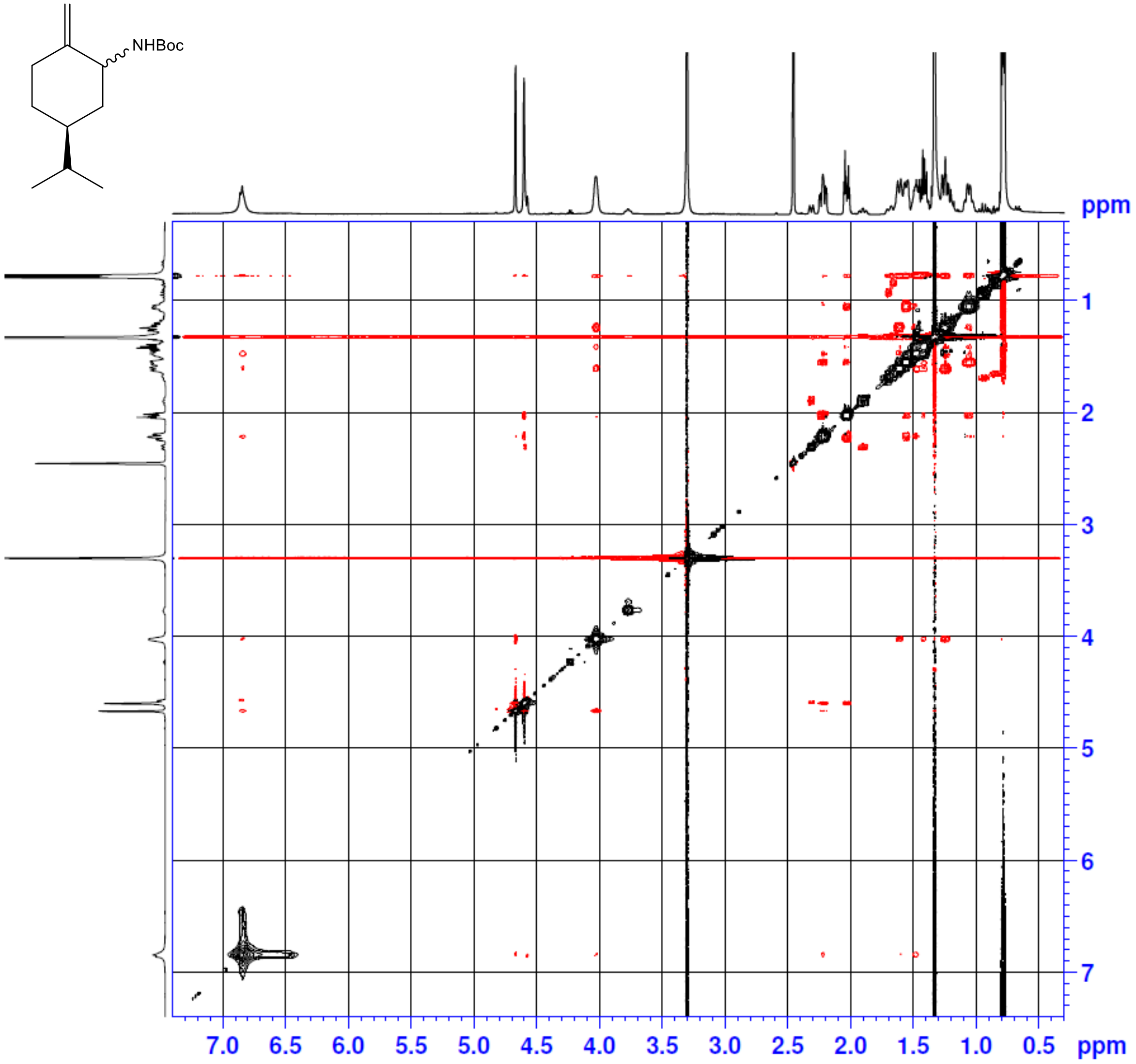

Figure S 96: **^{1}H-NMR of compound** *tert*-Butyl ((1*R*,2*S*,5*S*)-2-hydroxy-2-hydroxymethyl-5-isopropylcyclohexyl)-carbamate **19a**

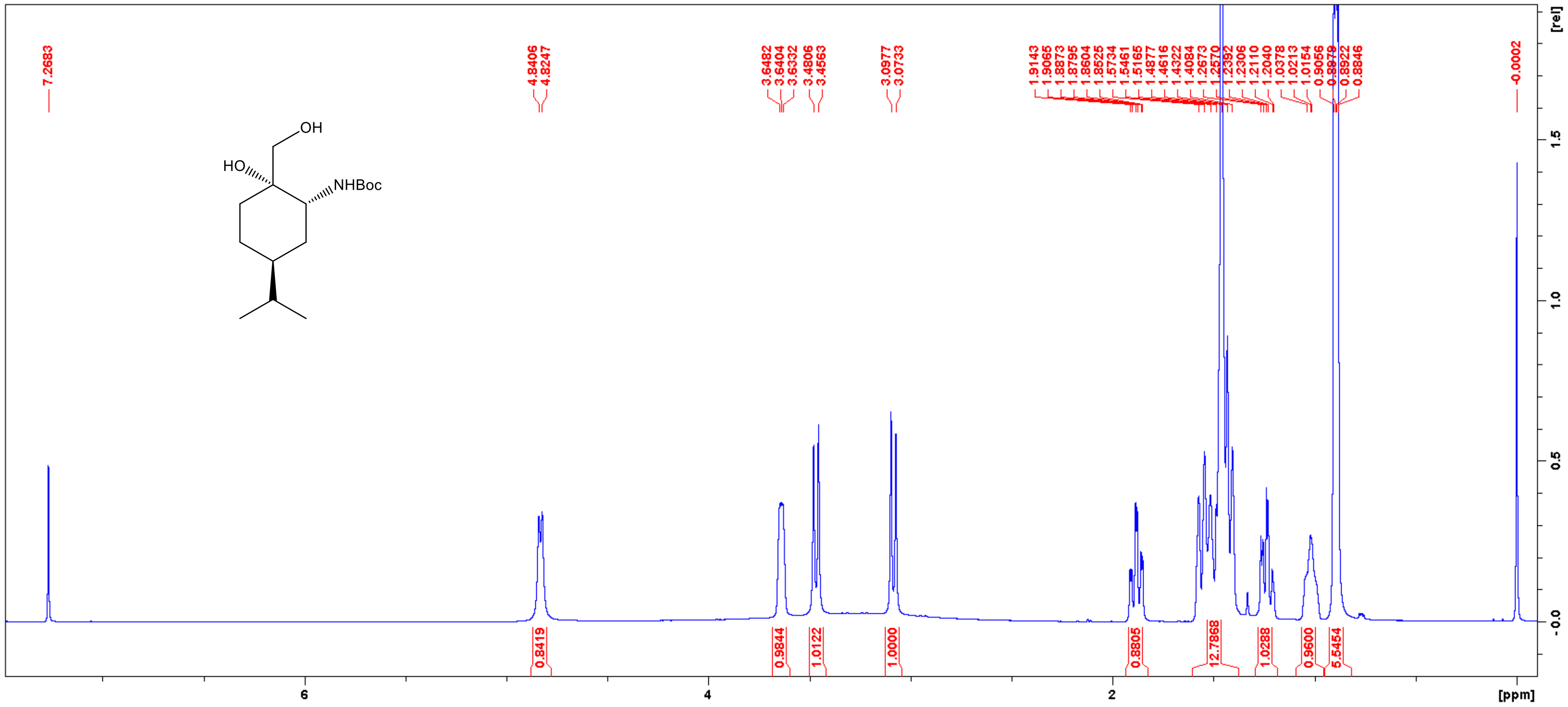

Figure S 97: ^{13}C-NMR of compound *tert*-Butyl ((1*R*,2*S*,5*S*)-2-hydroxy-2-hydroxymethyl-5-isopropylcyclohexyl)-carbamate **19a**

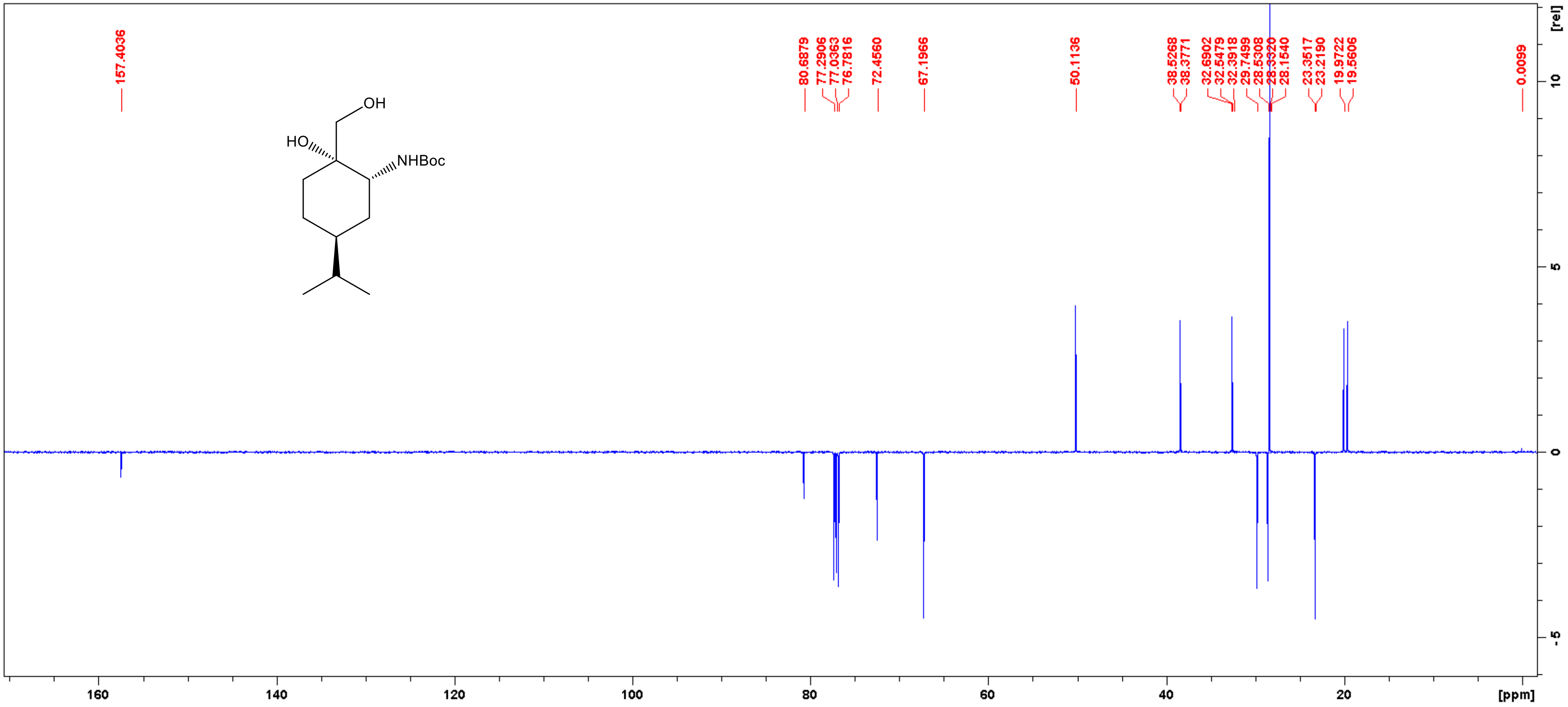

Figure S 98: HSQC NMR of compound *tert*-Butyl ((1*R*,2*S*,5*S*)-2-hydroxy-2-hydroxymethyl-5-isopropylcyclohexyl)-carbamate **19a**

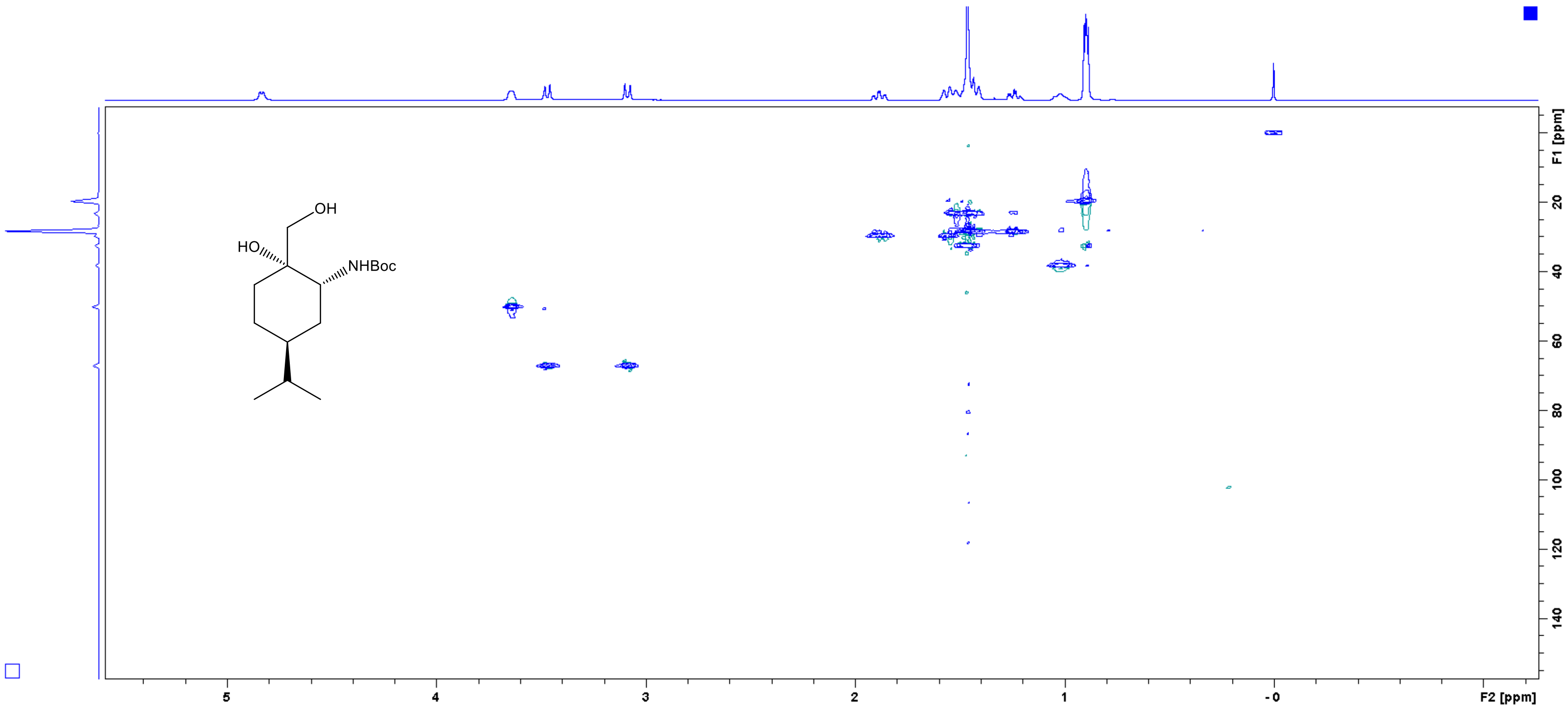

Figure S 99: HMBC NMR of compound *tert*-Butyl ((1*R*,2*S*,5*S*)-2-hydroxy-2-hydroxymethyl-5-isopropylcyclohexyl)-carbamate **19a**

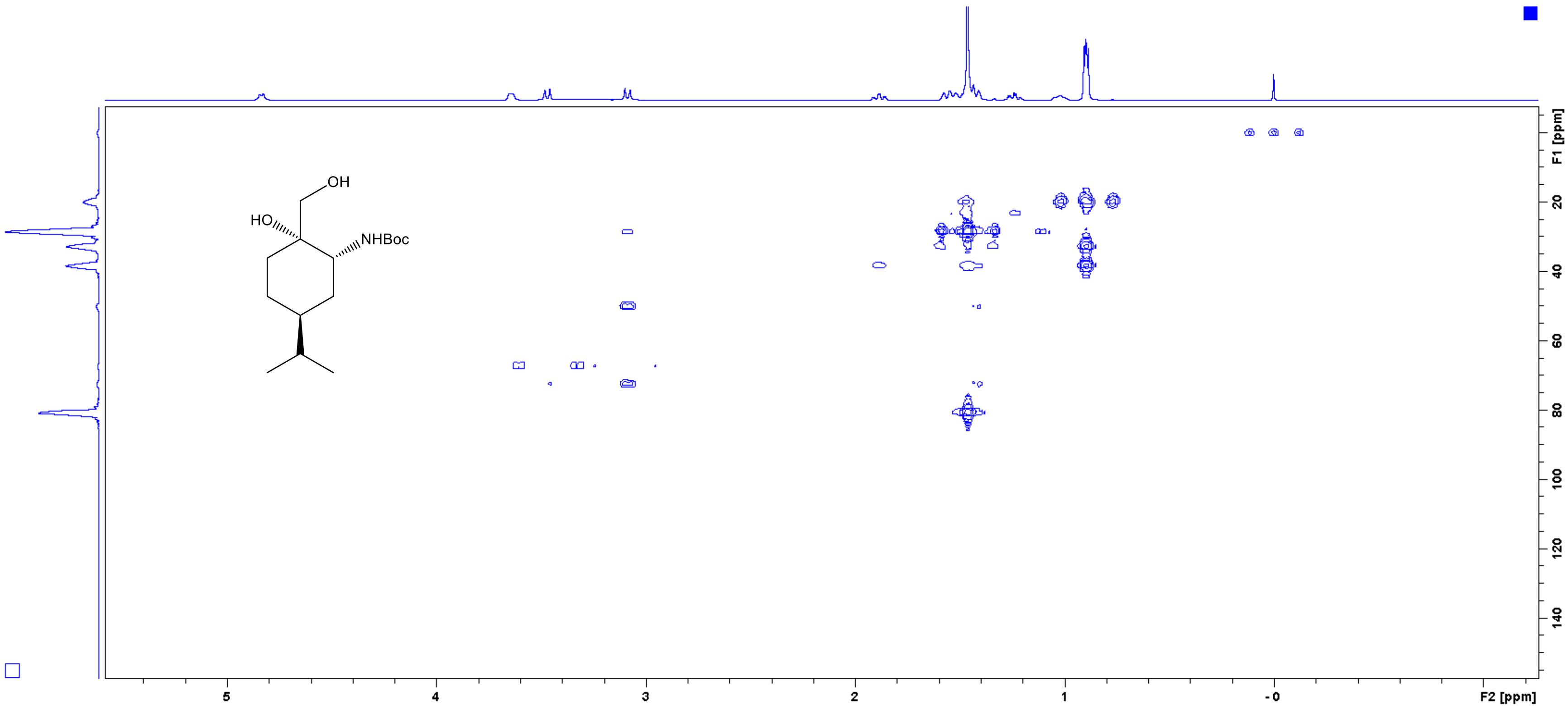

Figure S 100: ¹H-NMR of compound (1*R*,2*R*,5*S*)- and (1*S*,2*R*,5*S*)-*tert*-Butyl 2-hydroxy-2-hydroxymethyl-5-isopropylcyclohexyl)carbamate mixture **19b- c**

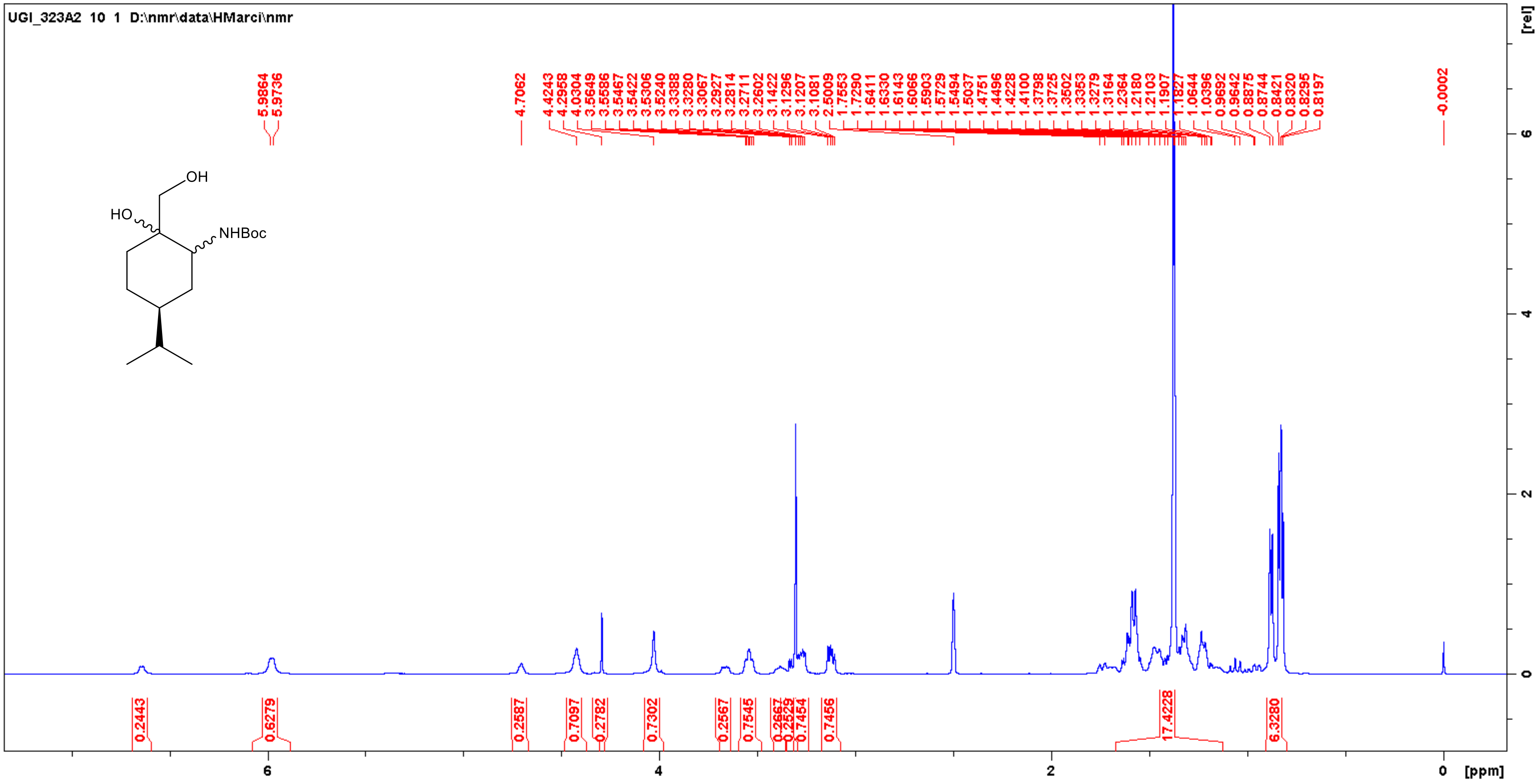

Figure S 101: ^{13}C-NMR of compound (1*R*,2*R*,5*S*)- and (1*S*,2*R*,5*S*)-*tert*-Butyl 2-hydroxy-2-hydroxymethyl-5-isopropylcyclohexyl)carbamate mixture **19b- c**

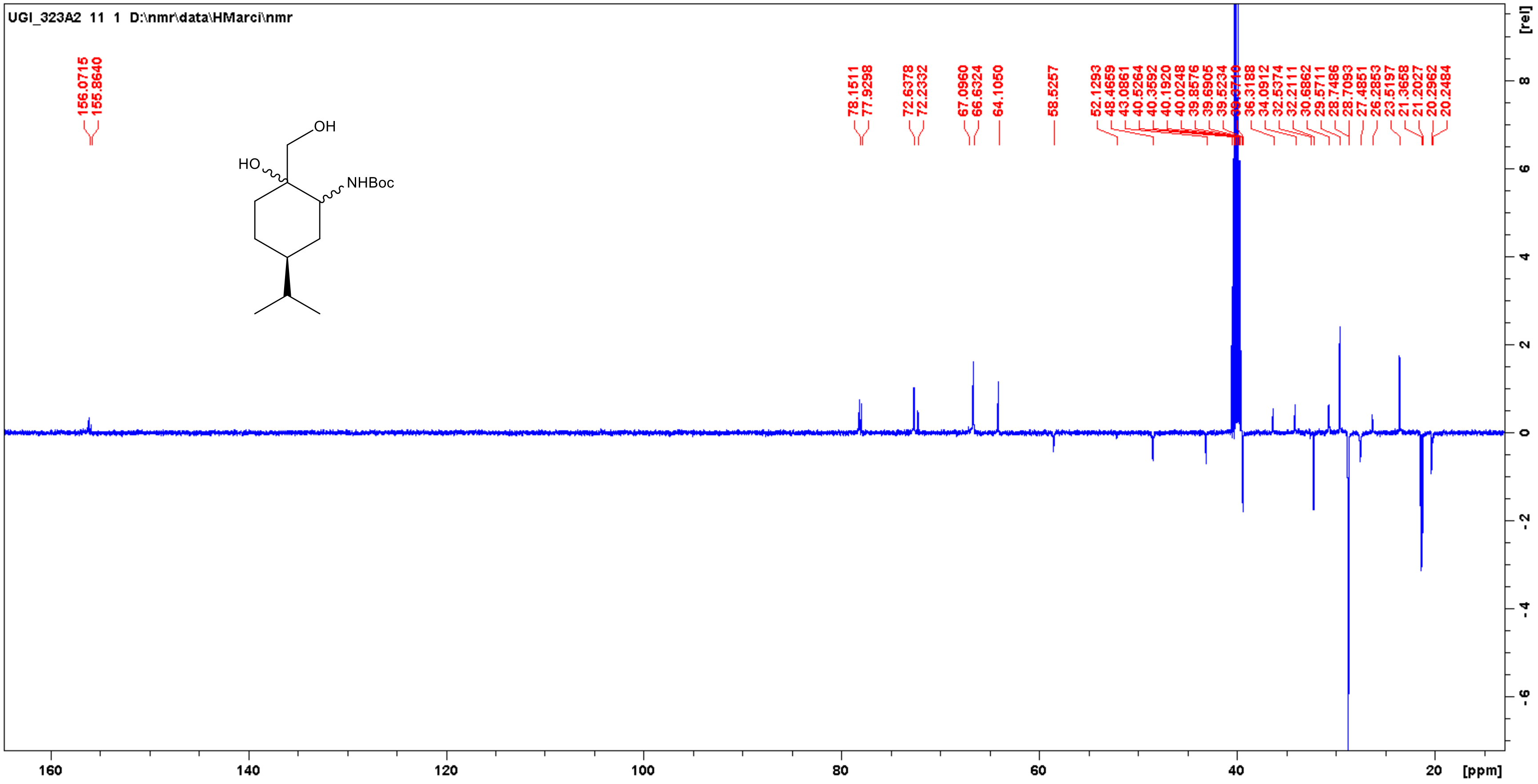

Figure S 102: COSY-NMR of compound (1*R*,2*R*,5*S*)- and (1*S*,2*R*,5*S*)-*tert*-Butyl 2-hydroxy-2-hydroxymethyl-5-isopropylcyclohexyl)carbamate mixture **19b- c**

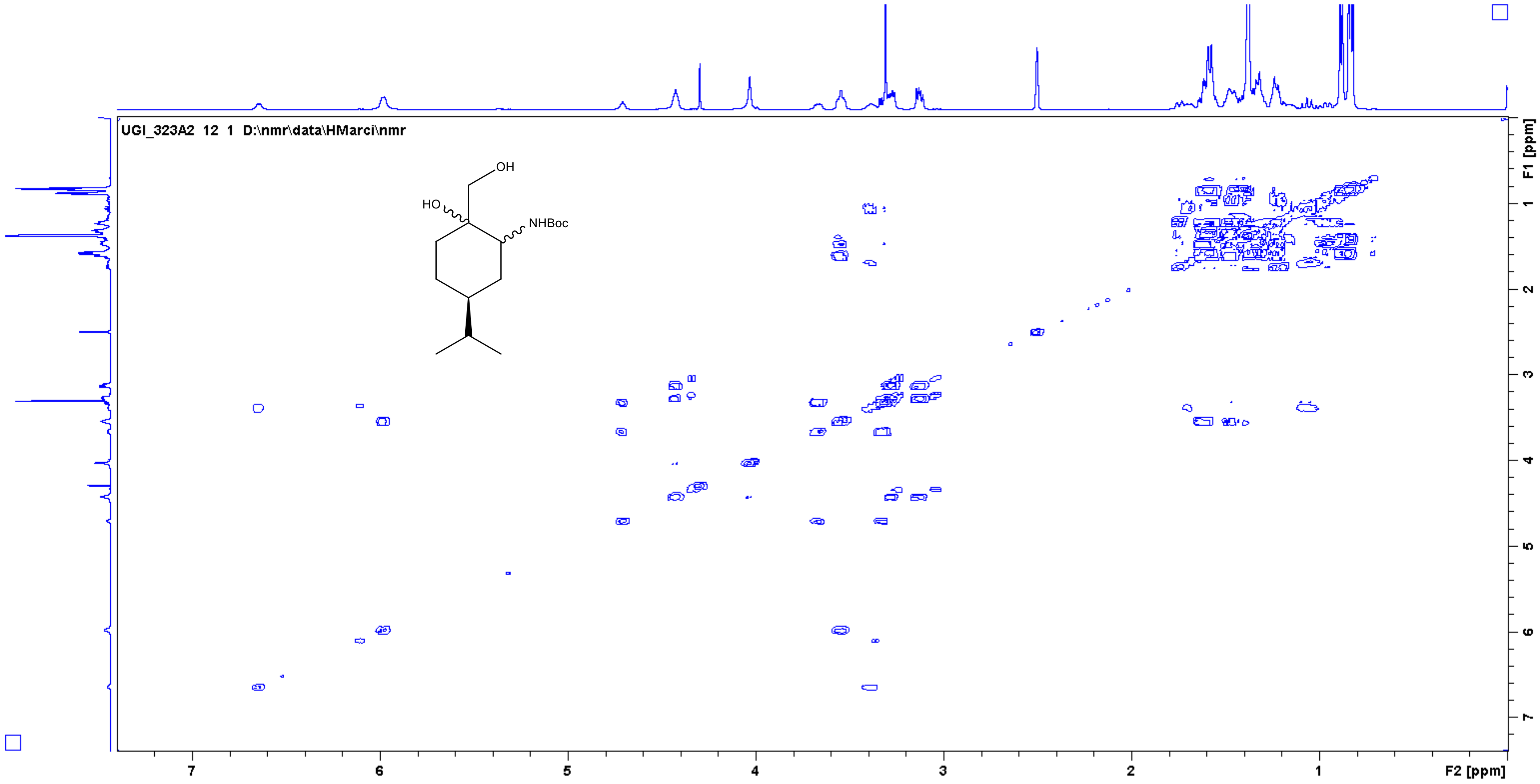

Figure S 103: ¹H-NMR of compound *tert*-Butyl ((5*R*,6*R*,8*S*)-8-isopropyl-2,2-dimethyl-1,3-dioxaspiro[4,5]decane-6-yl)carbamate **20b**

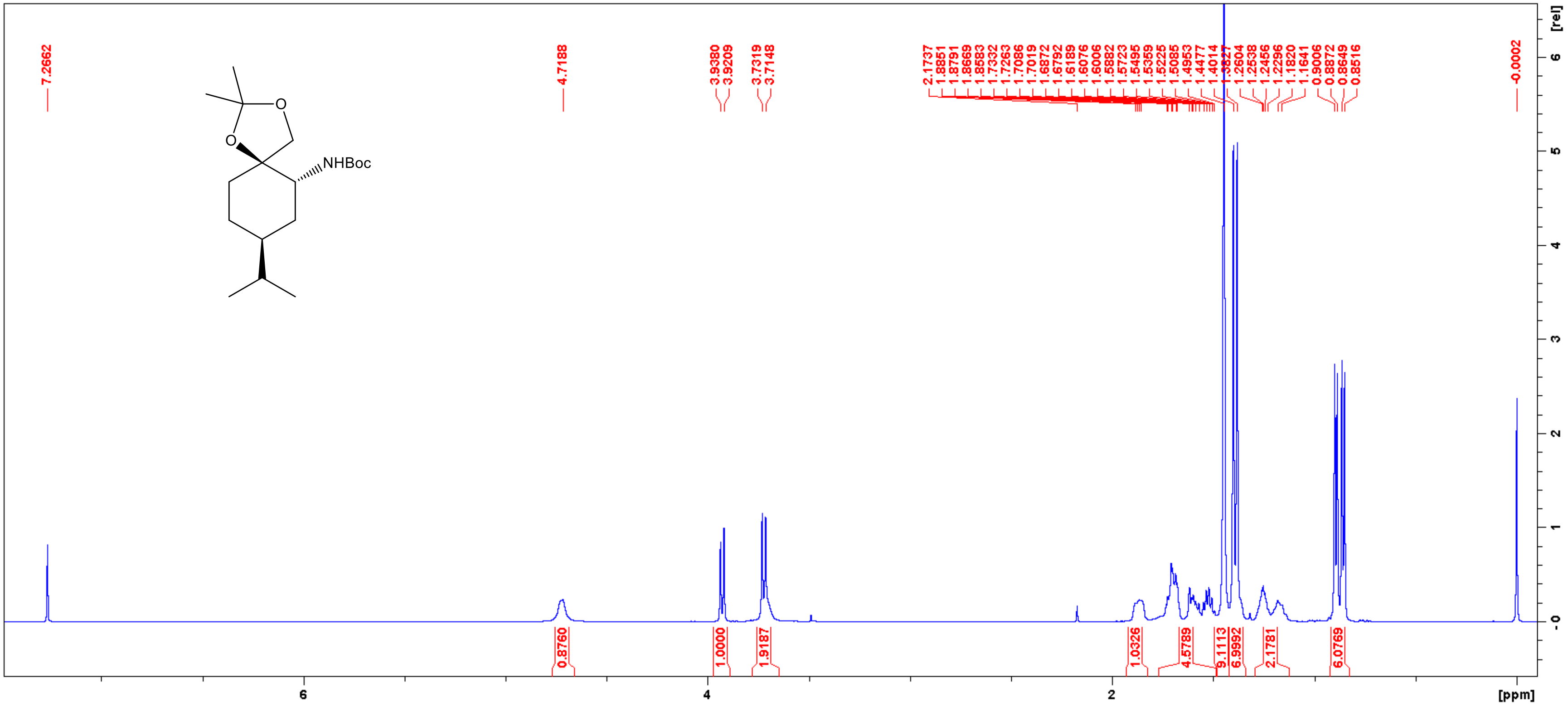

Figure S 104: ^{13}C-NMR of compound *tert*-Butyl ((5*R*,6*R*,8*S*)-8-isopropyl-2,2-dimethyl-1,3-dioxaspiro[4,5]decane-6-yl)carbamate **20b**

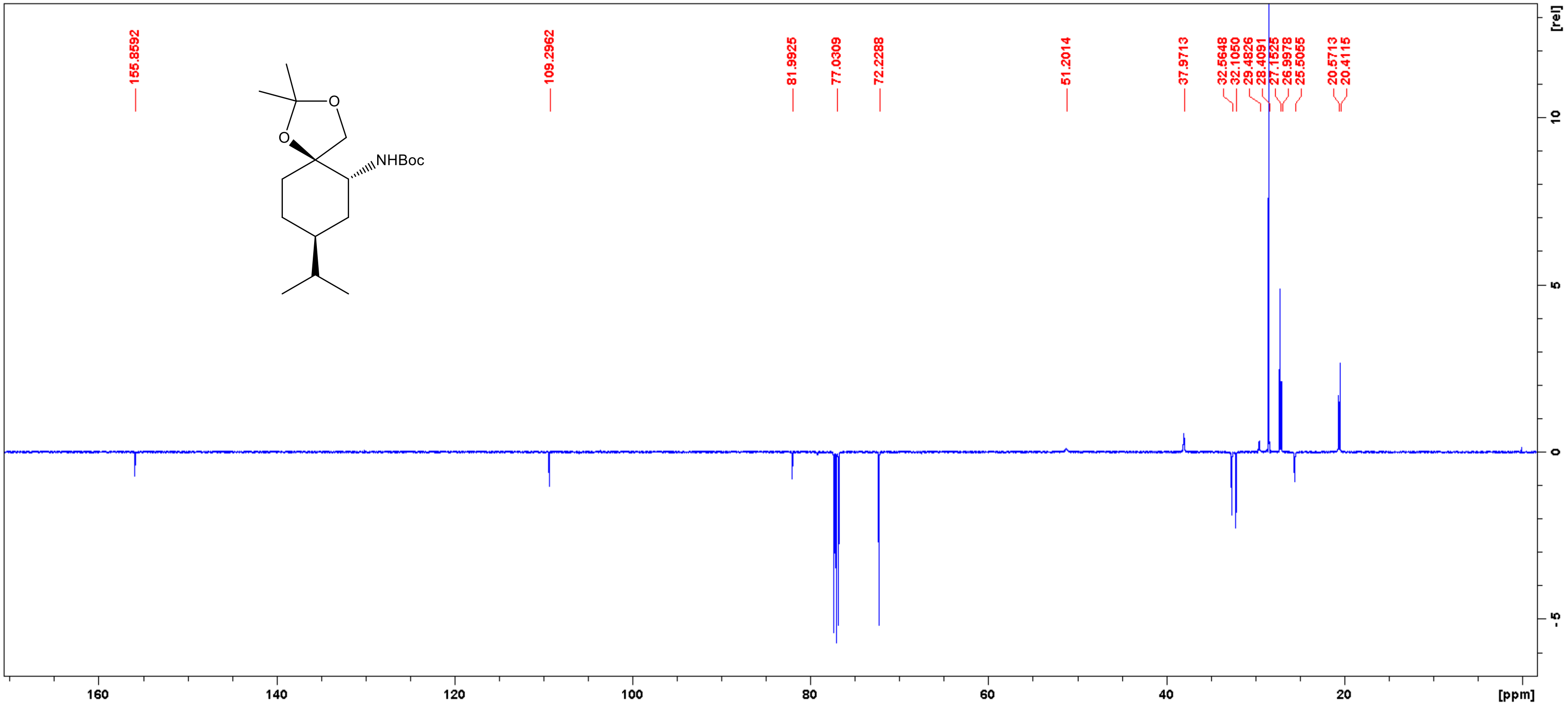

Figure S 105: HSQC NMR of *tert*-Butyl ((5*R*,6*R*,8*S*)-8-isopropyl-2,2-dimethyl-1,3-dioxaspiro[4,5]decane-6-yl)carbamate **20b**

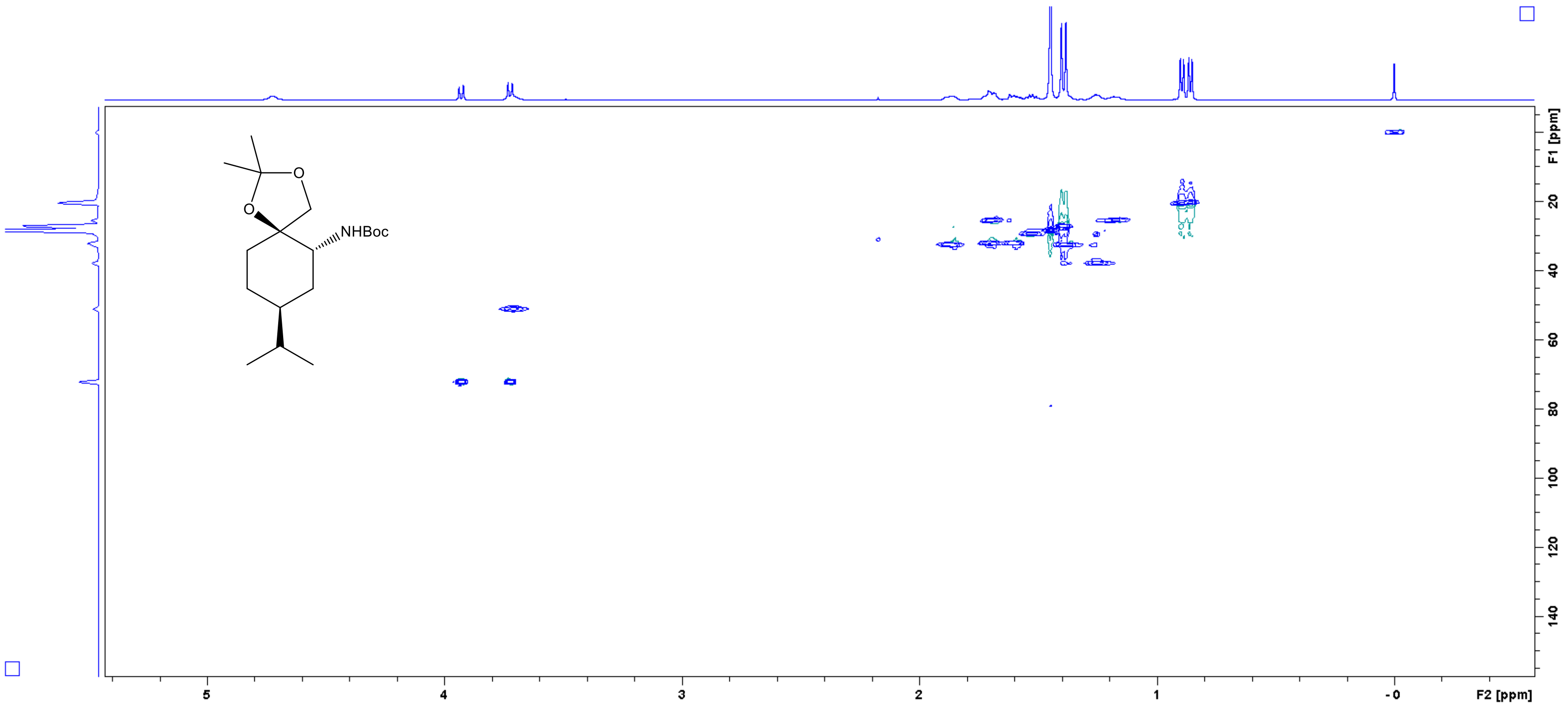

Figure S 106: HMBC NMR of compound *tert*-Butyl ((5*R*,6*R*,8*S*)-8-isopropyl-2,2-dimethyl-1,3-dioxaspiro[4,5]decane-6-yl)carbamate **20b**

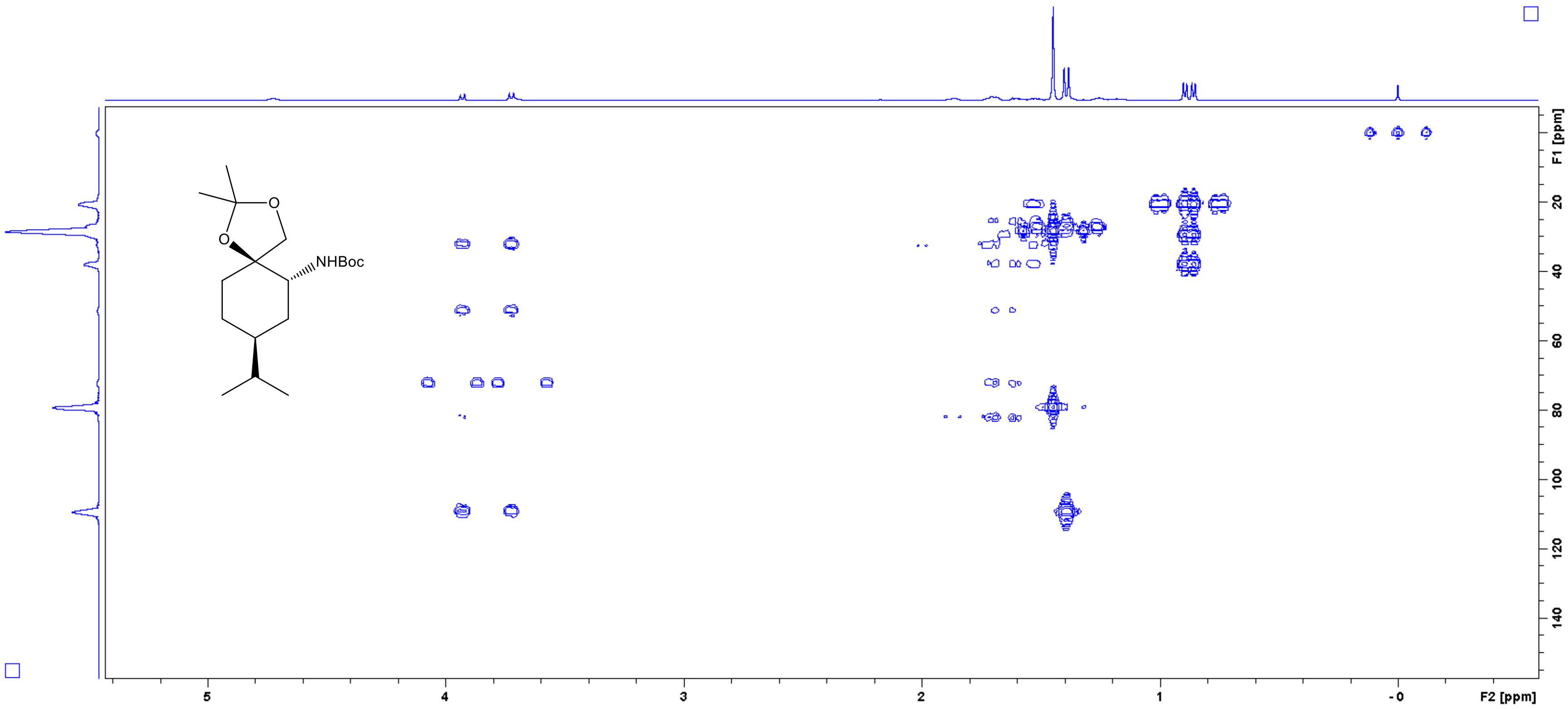

Figure S 107: ^{1}H-NMR of compound *tert*-Butyl ((5*R*,6*S*,8*S*)-8-isopropyl-2,2-dimethyl-1,3-dioxaspiro[4,5]decane-6-yl)carbamate **20c**

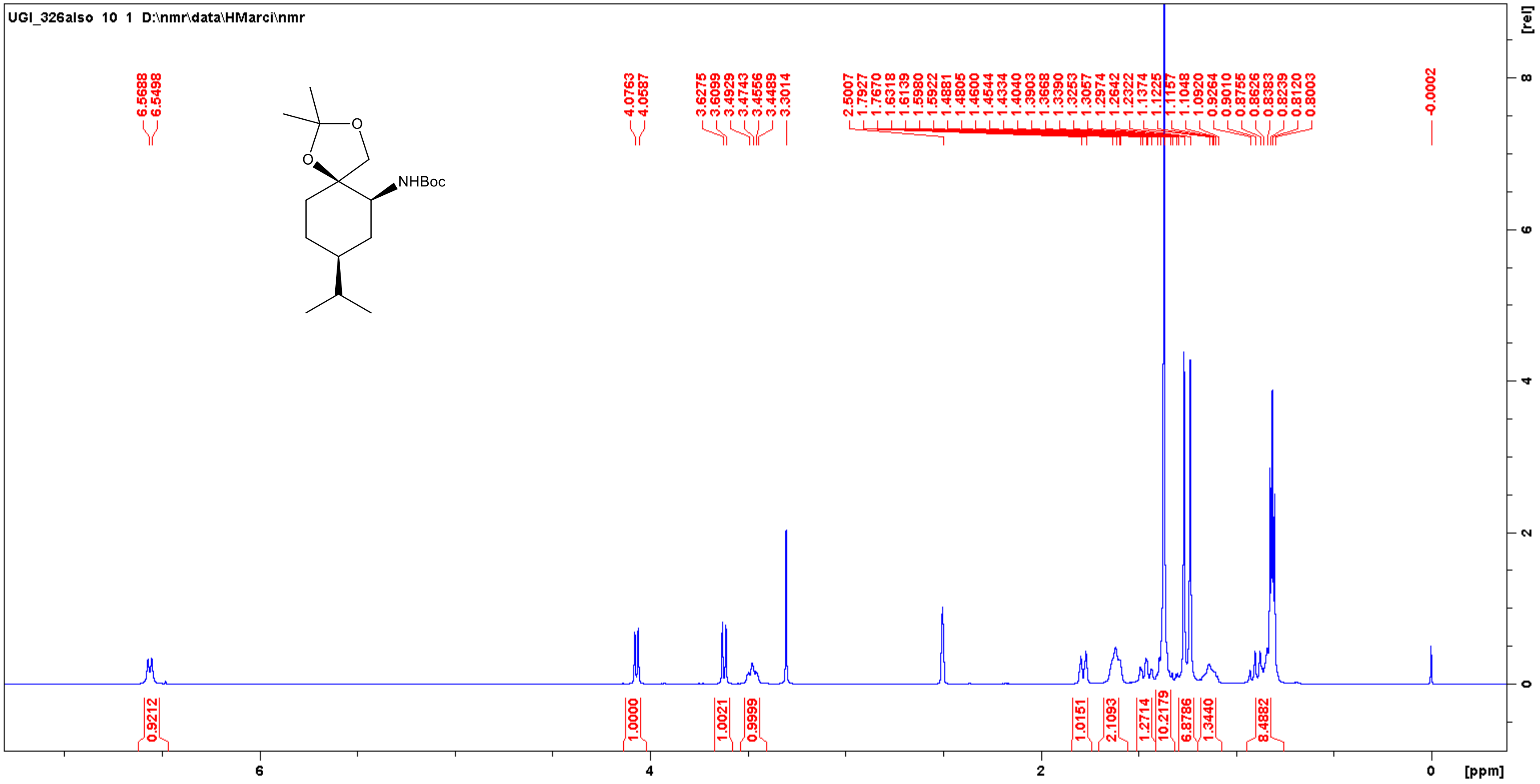

Figure S 108: ^{13}C-NMR of compound *tert*-Butyl ((5*R*,6*S*,8*S*)-8-isopropyl-2,2-dimethyl-1,3-dioxaspiro[4,5]decane-6-yl)carbamate **20c**

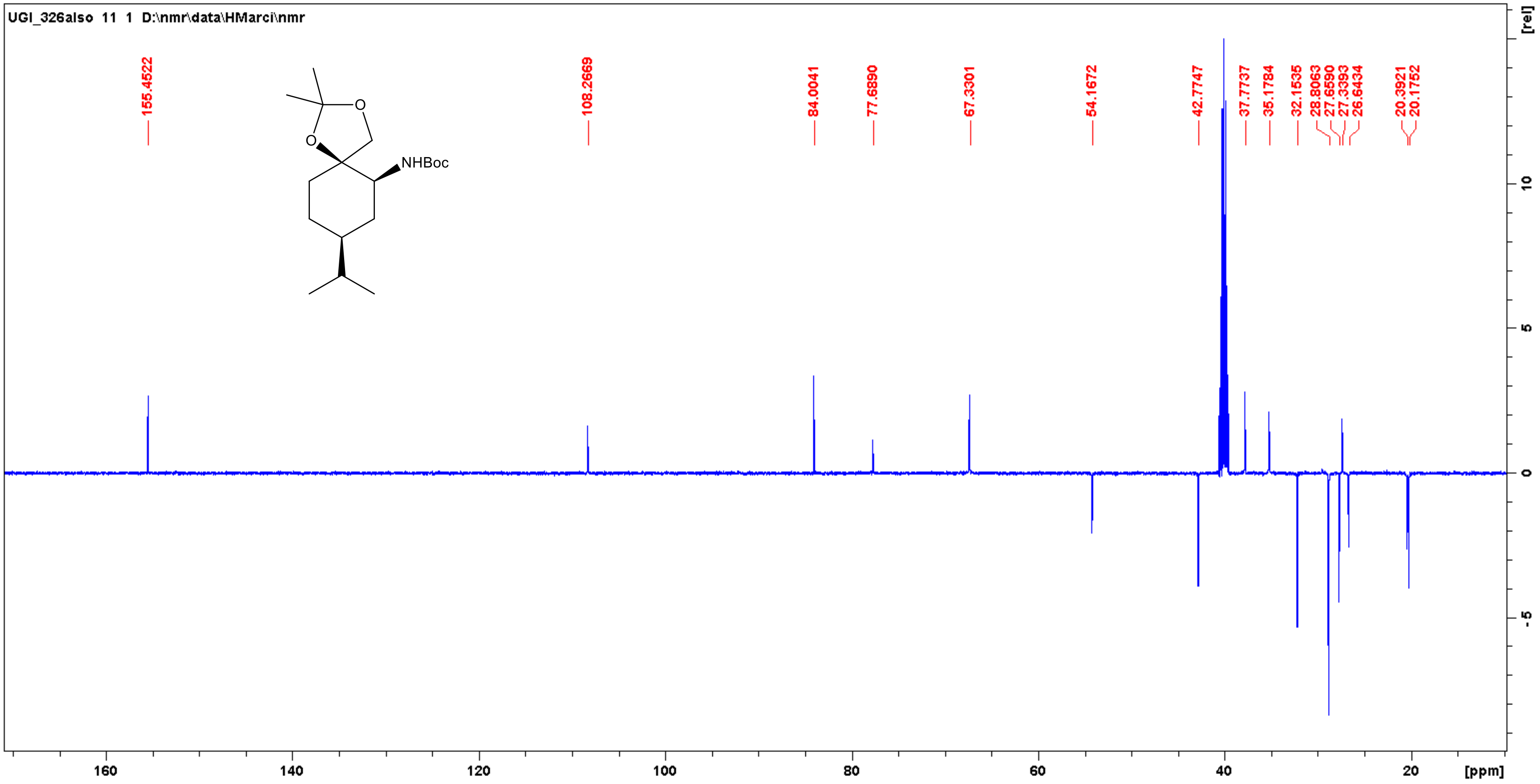

Figure S 109: COSY NMR of compound *tert*-Butyl ((5*R*,6*S*,8*S*)-8-isopropyl-2,2-dimethyl-1,3-dioxaspiro[4,5]decane-6-yl)carbamate **20c**

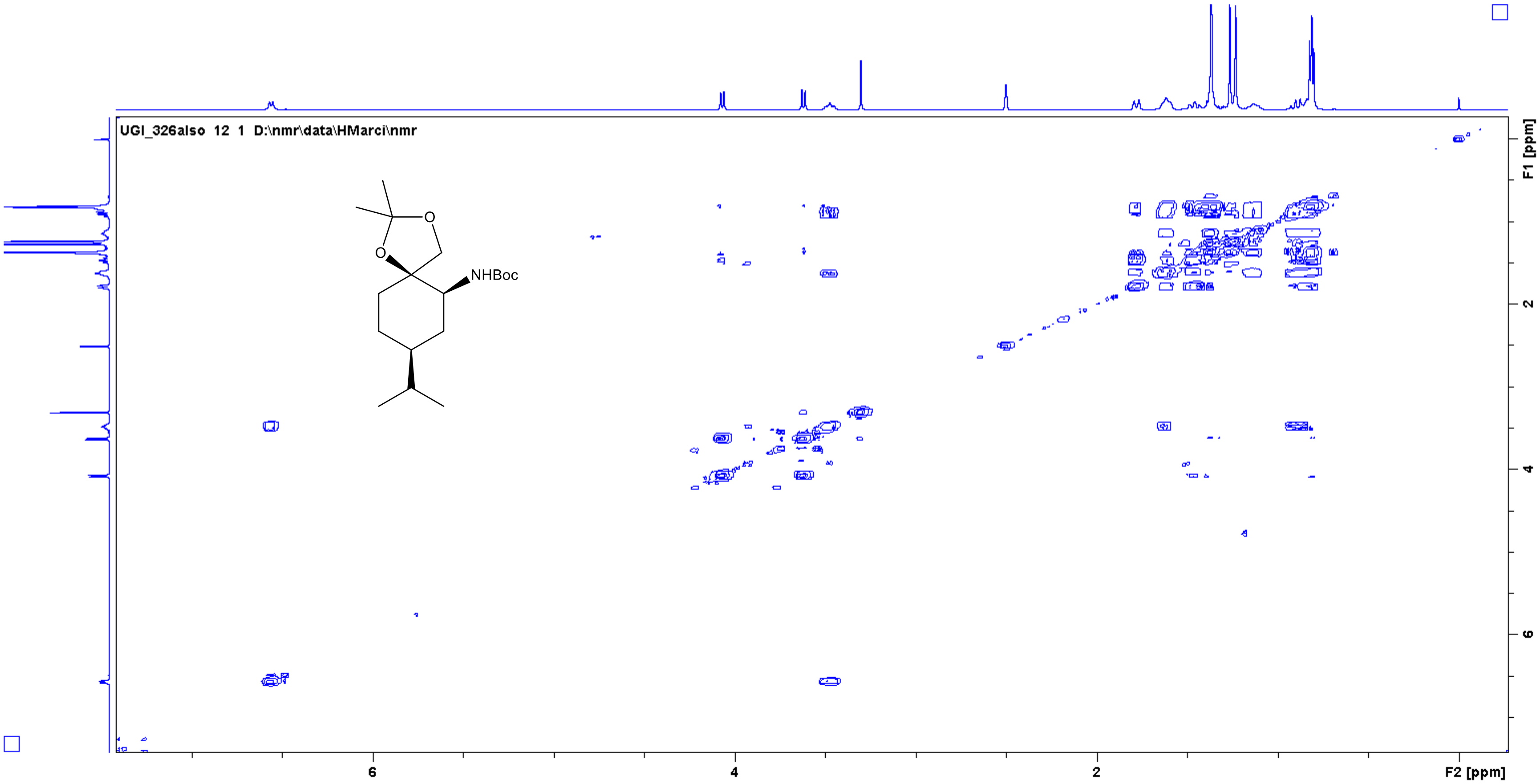

Figure S 110: HSQC NMR of compound *tert*-Butyl ((5*R*,6*S*,8*S*)-8-isopropyl-2,2-dimethyl-1,3-dioxaspiro[4,5]decane-6-yl)carbamate **20c**

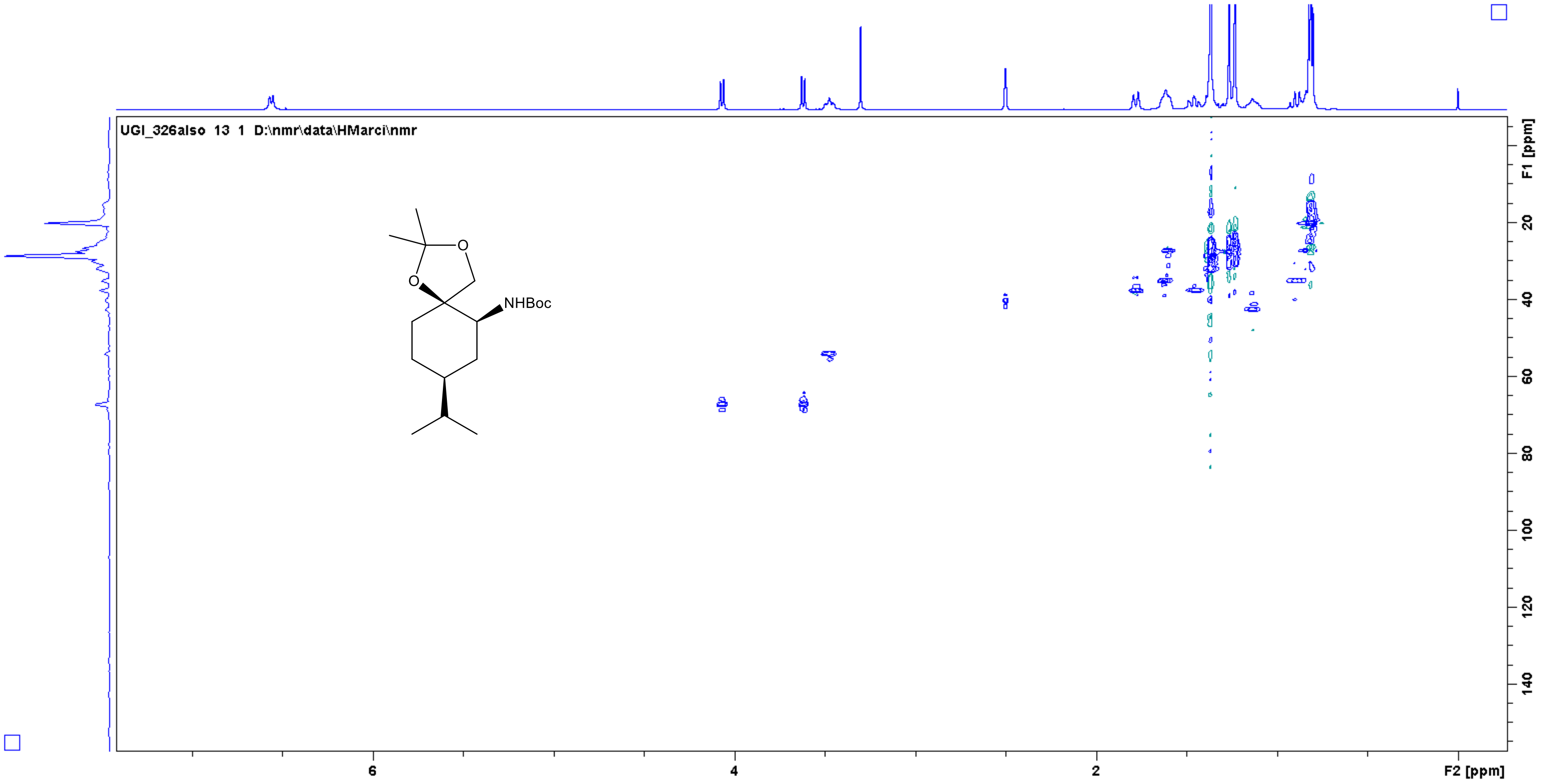

Figure S 111: ^{1}H-NMR of compound (1*S*,2*R*,4*S*)-2-Amino-1-hydroxymethyl-4-isopropylcyclohexanol hydrochloride **21a**

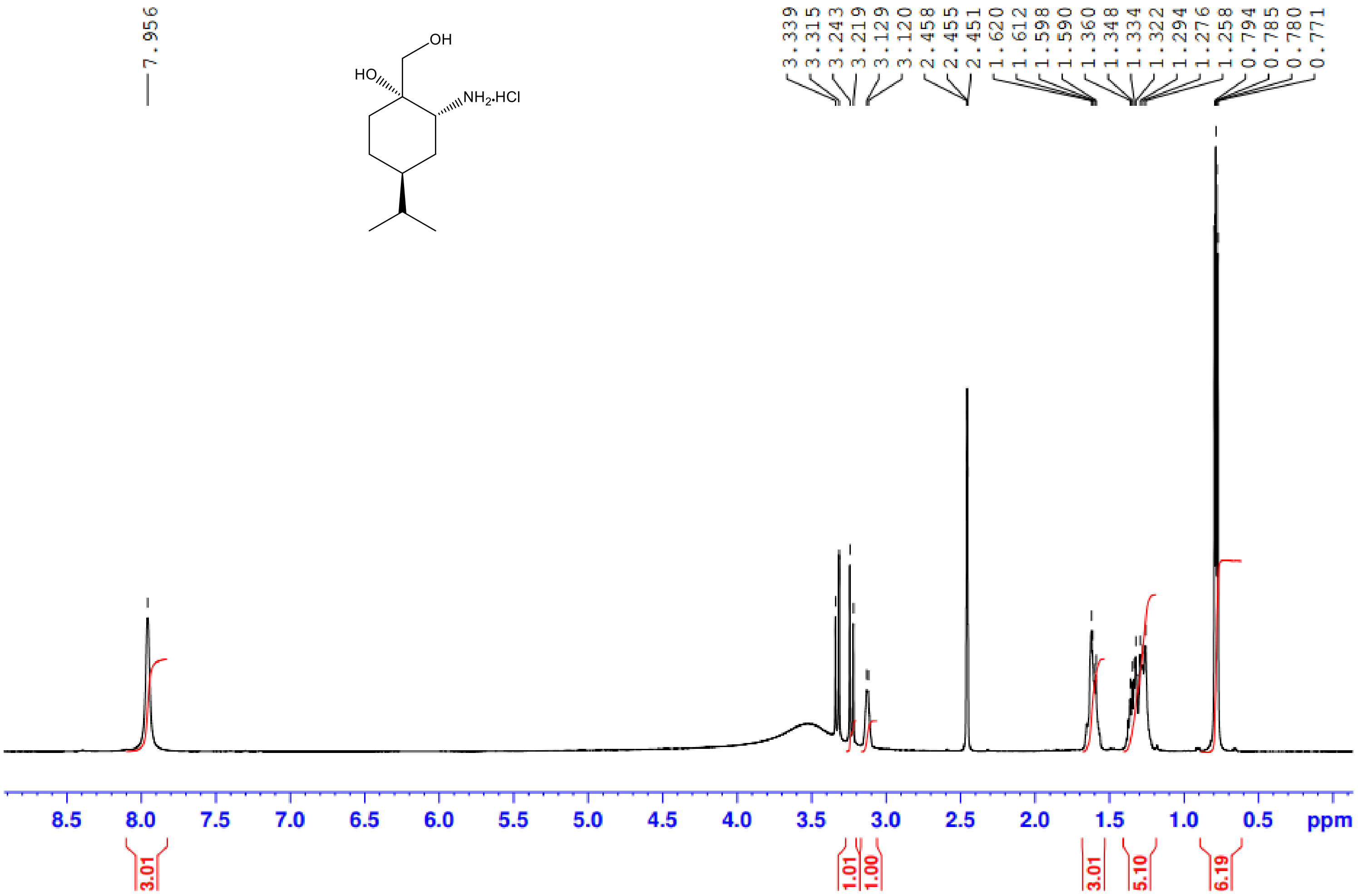

Figure S 112: ^{13}C-NMR of compound (1*S*,2*R*,4*S*)-2-Amino-1-hydroxymethyl-4-isopropylcyclohexanol hydrochloride **21a**

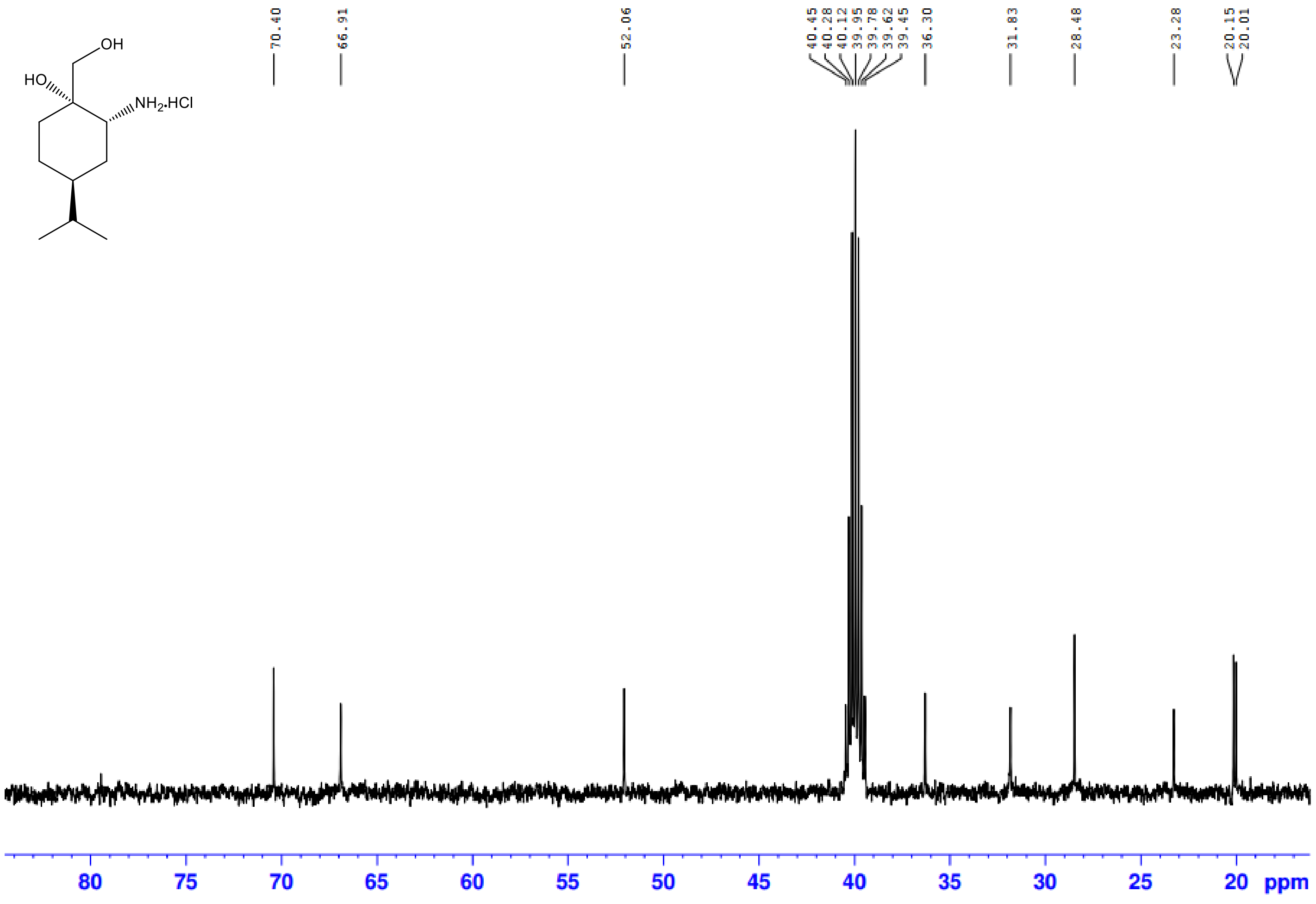

Figure S 113: NOESY-NMR of compound (1*S*,2*R*,4*S*)-2-Amino-1-hydroxymethyl-4-isopropylcyclohexanol hydrochloride **21a**

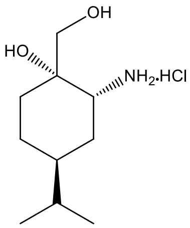

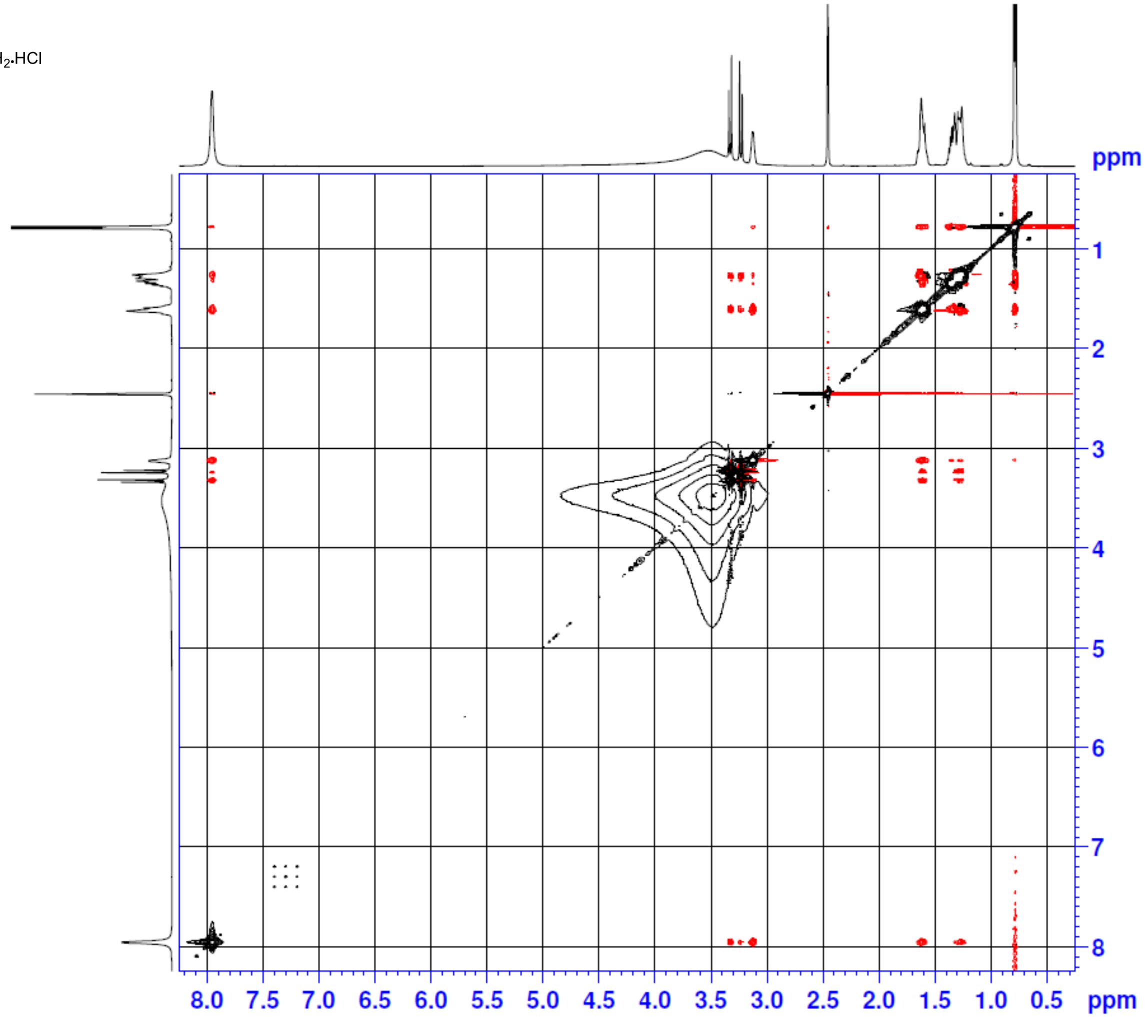

Figure S 114: ^{1}H-NMR of compound (1*R*,2*R*,4*S*)-2-Amino-1-hydroxymethyl-4-isopropylcyclohexanol hydrochloride **21b**

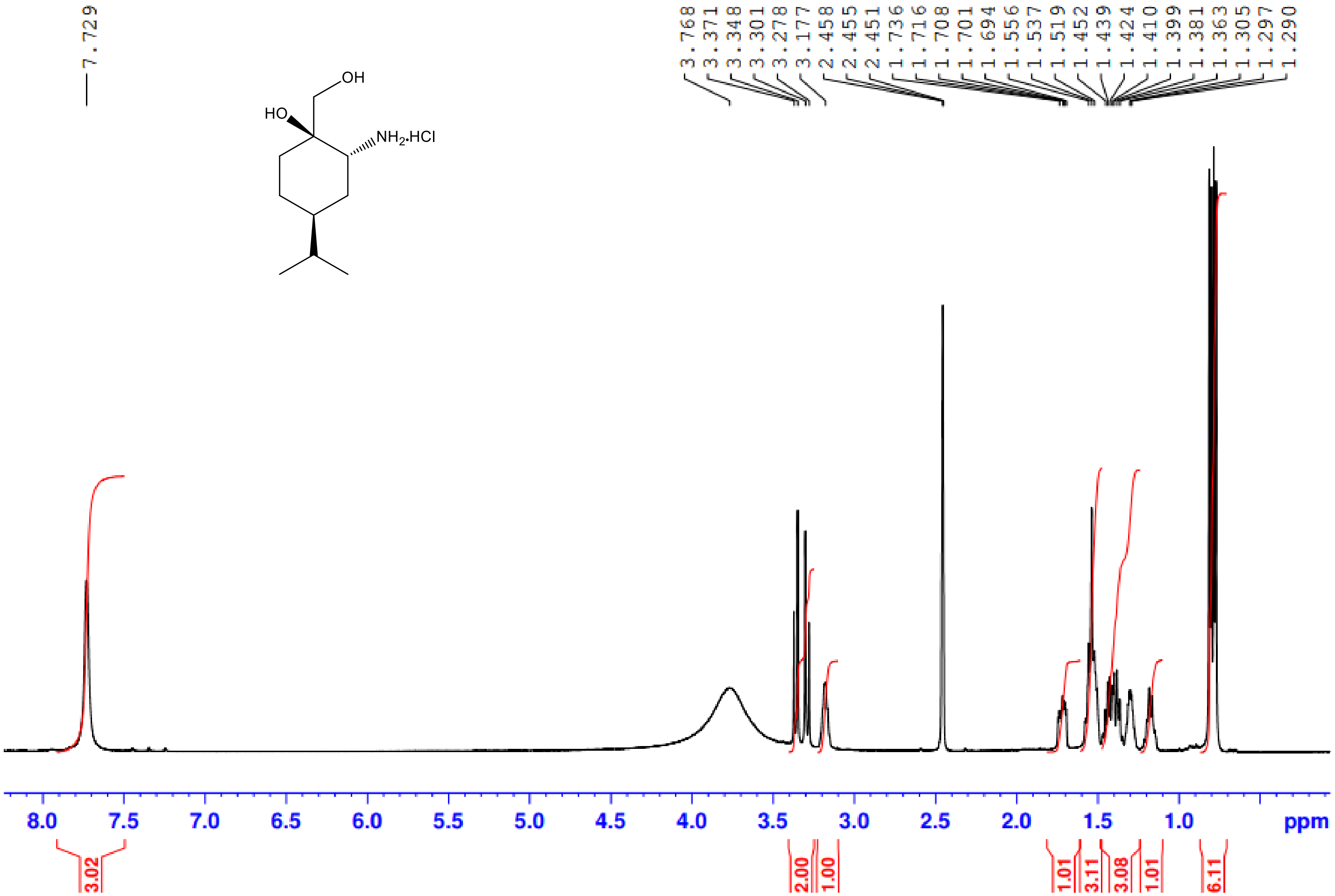

Figure S 115: ^{13}C-NMR of compound (1*R*,2*R*,4*S*)-2-Amino-1-hydroxymethyl-4-isopropylcyclohexanol hydrochloride **21b**

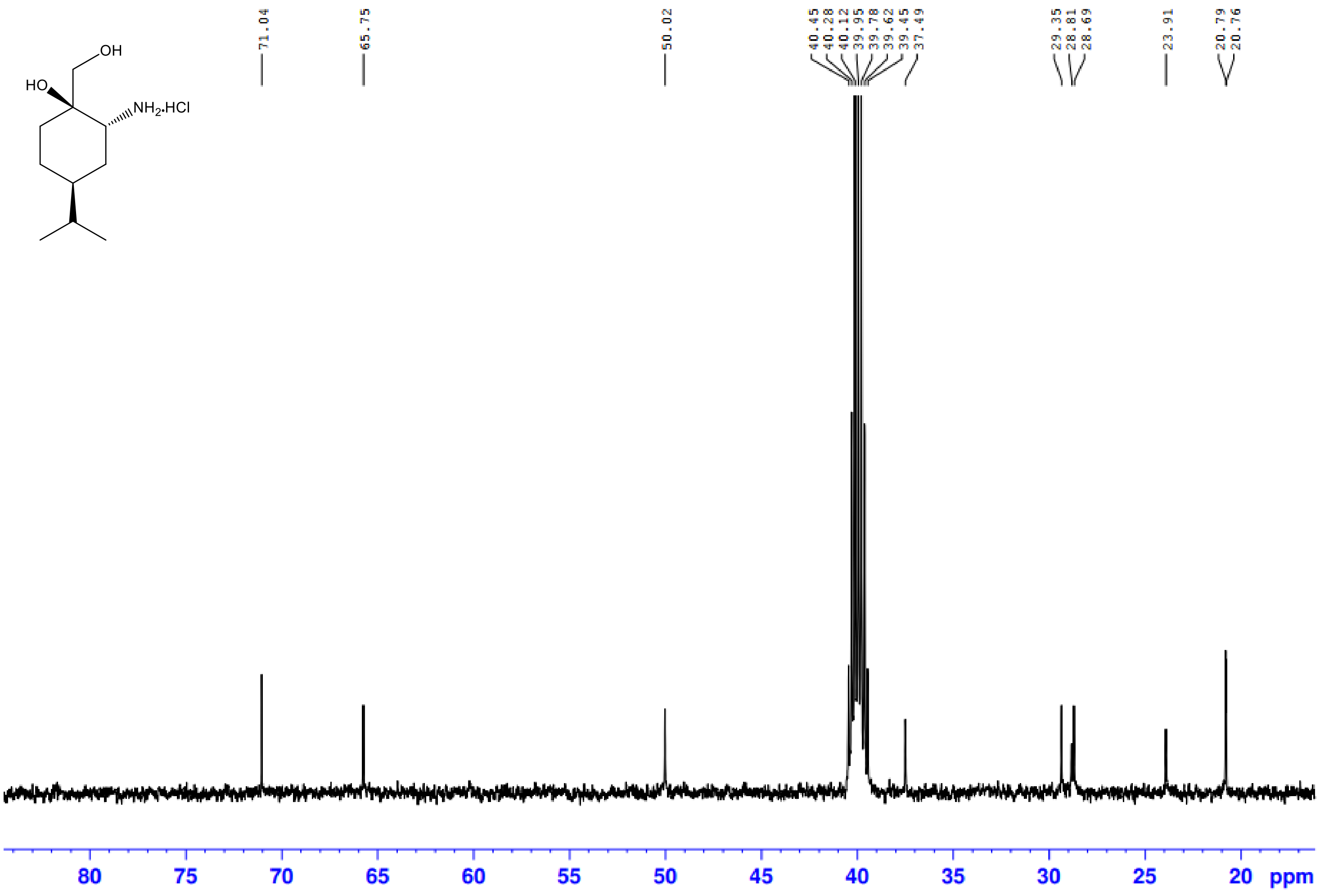

Figure S 116: NOESY-NMR of compound (1*R*,2*R*,4*S*)-2-Amino-1-hydroxymethyl-4-isopropylcyclohexanol hydrochloride **21b**

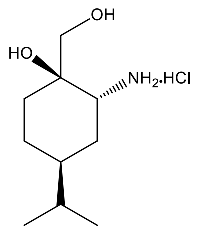

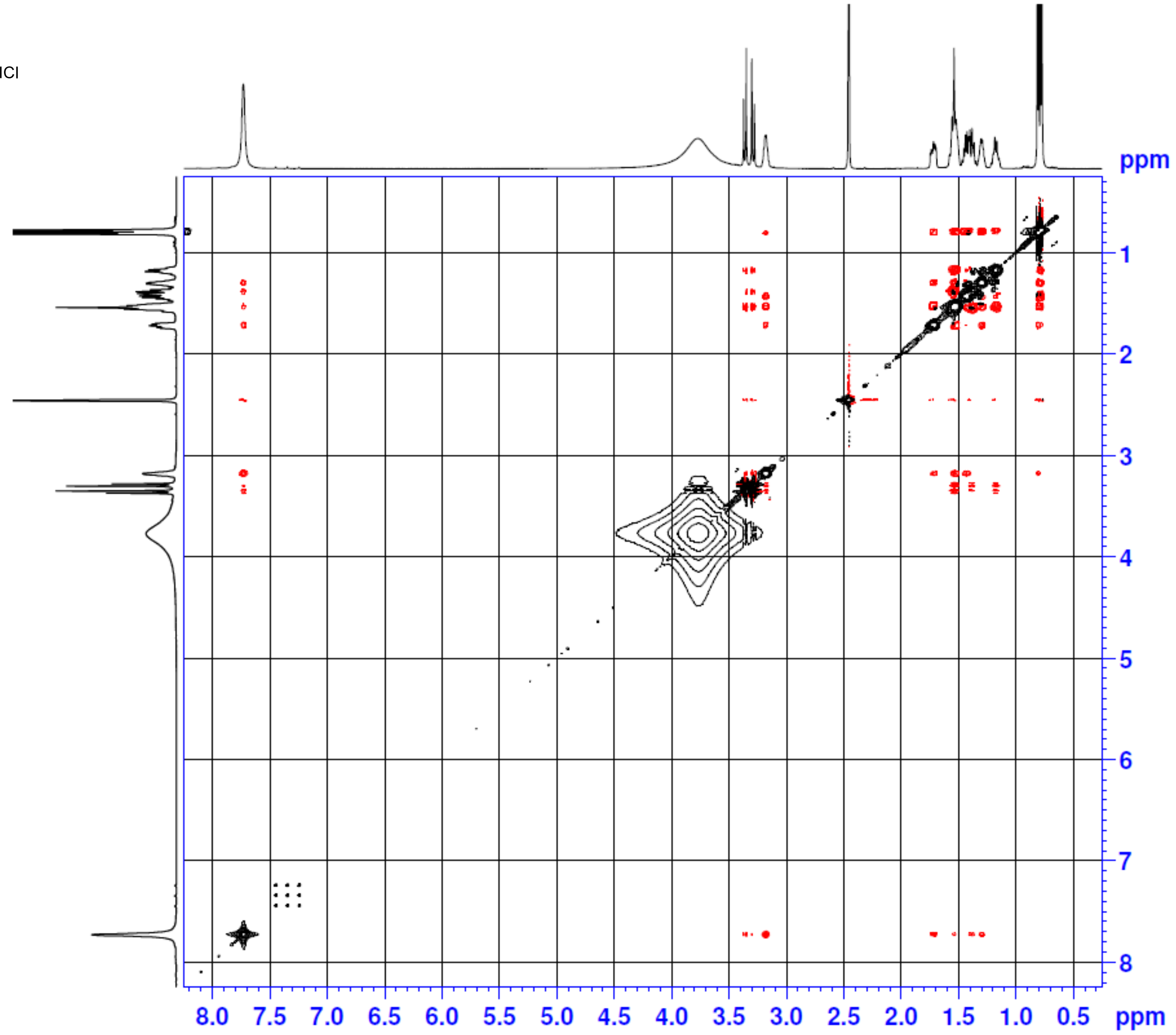

Figure S 117: ¹H-NMR of compound (1*R*,2*S*,4*S*)-2-Amino-1-hydroxymethyl-4-isopropylcyclohexanol hydrochloride **21c**

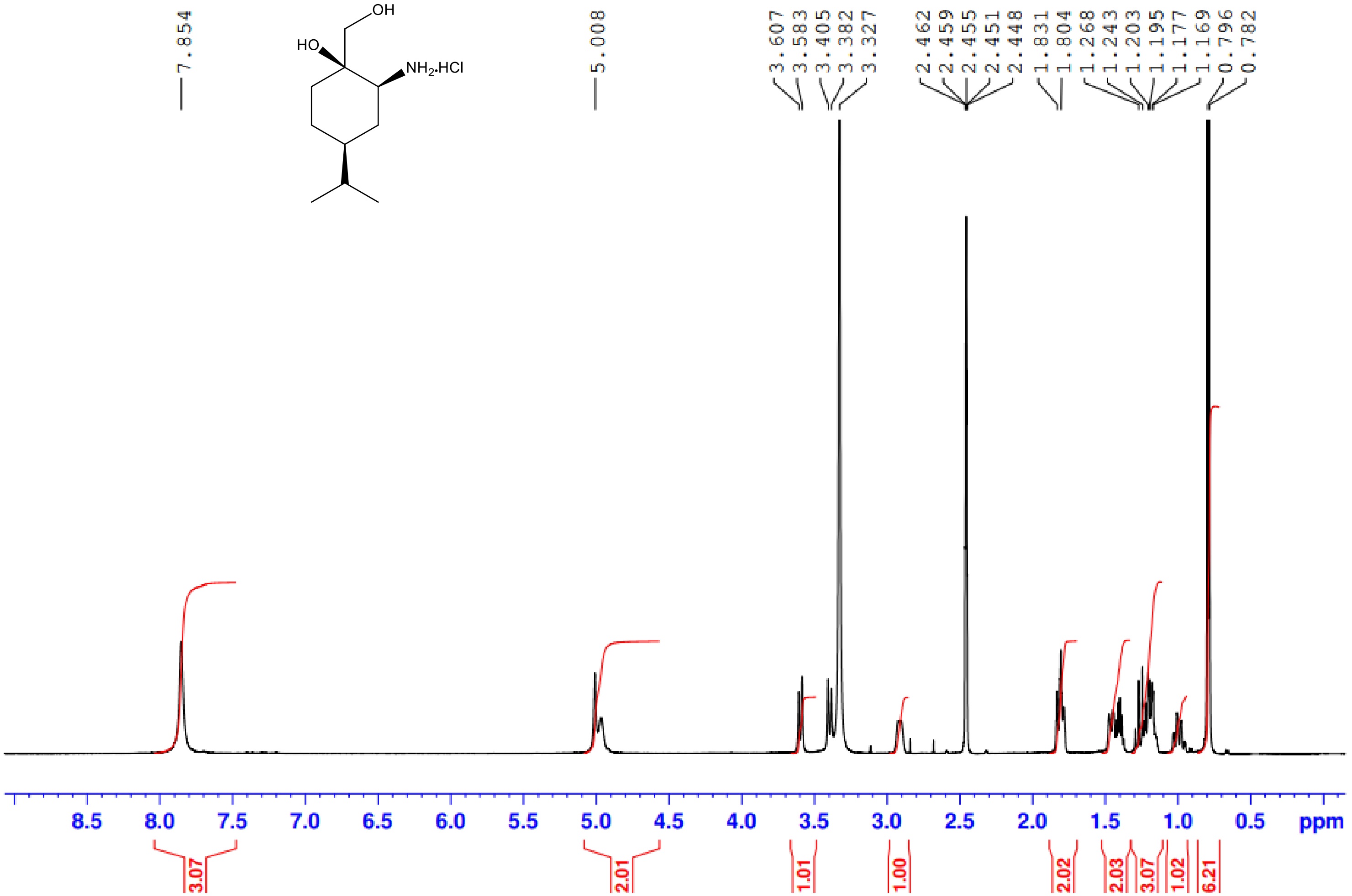

Figure S 118: ^{13}C-NMR of compound (1*R*,2*S*,4*S*)-2-Amino-1-hydroxymethyl-4-isopropylcyclohexanol hydrochloride **21c**

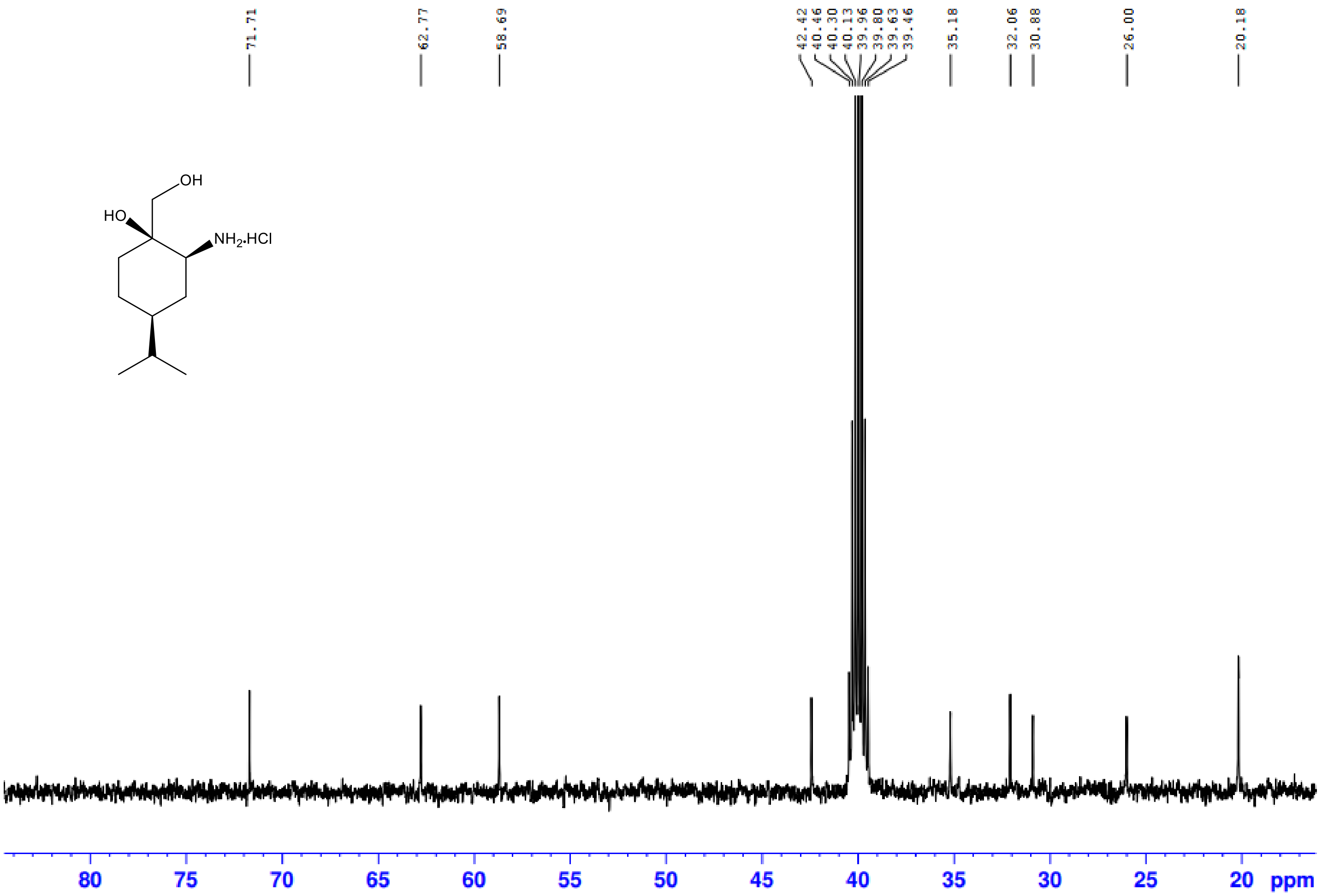

Figure S 119: NOESY-NMR of compound (1*R*,2*S*,4*S*)-2-Amino-1-hydroxymethyl-4-isopropylcyclohexanol hydrochloride **21c**

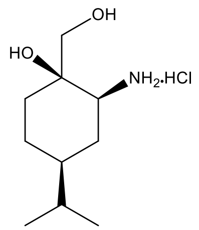

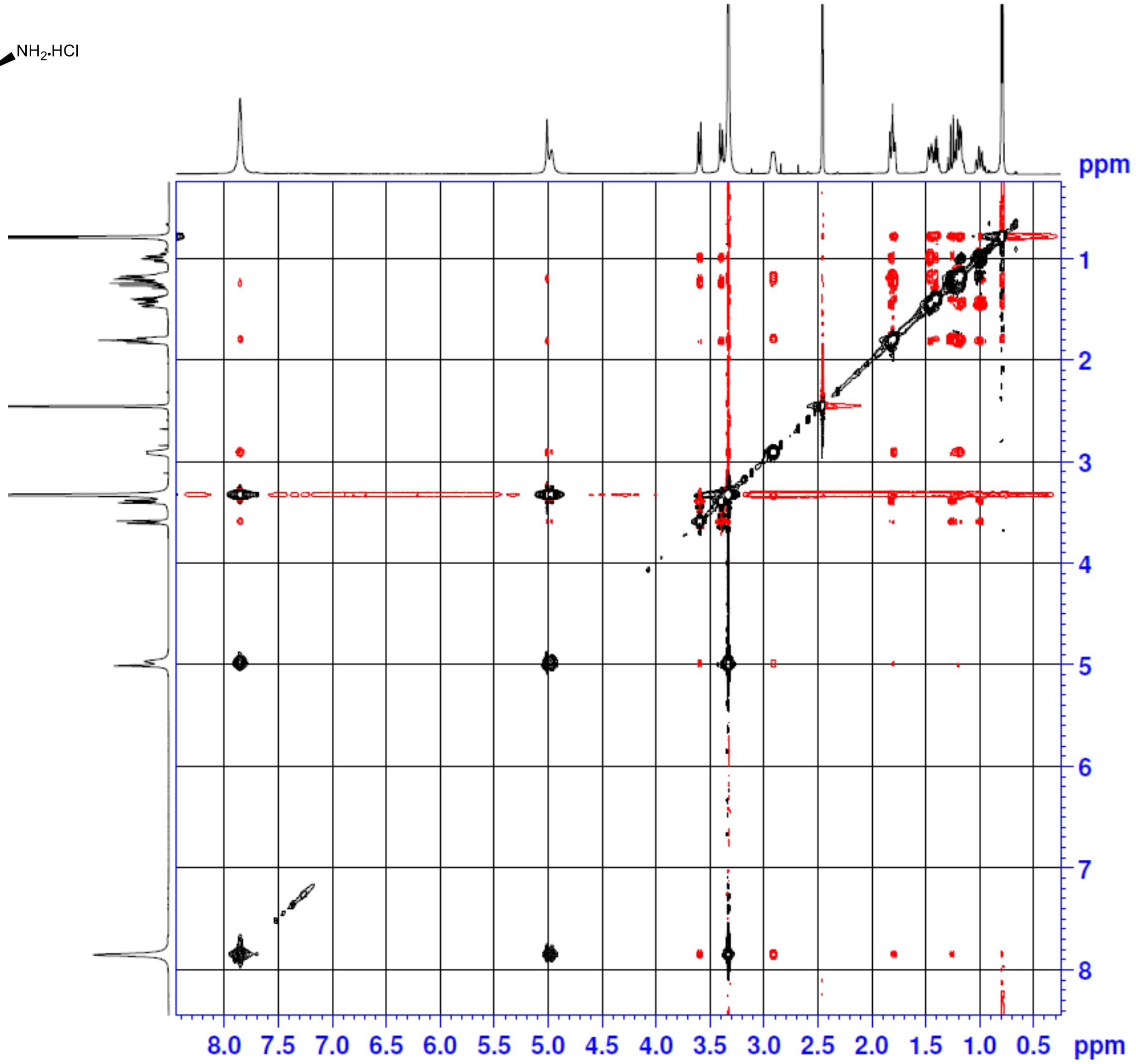

Figure S 120: ^{1}H-NMR of compound (1*S*,2*R*,4*S*)-2-Benzylamino-1-hydroxymethyl-4-isopropylcyclohexanol **23a**

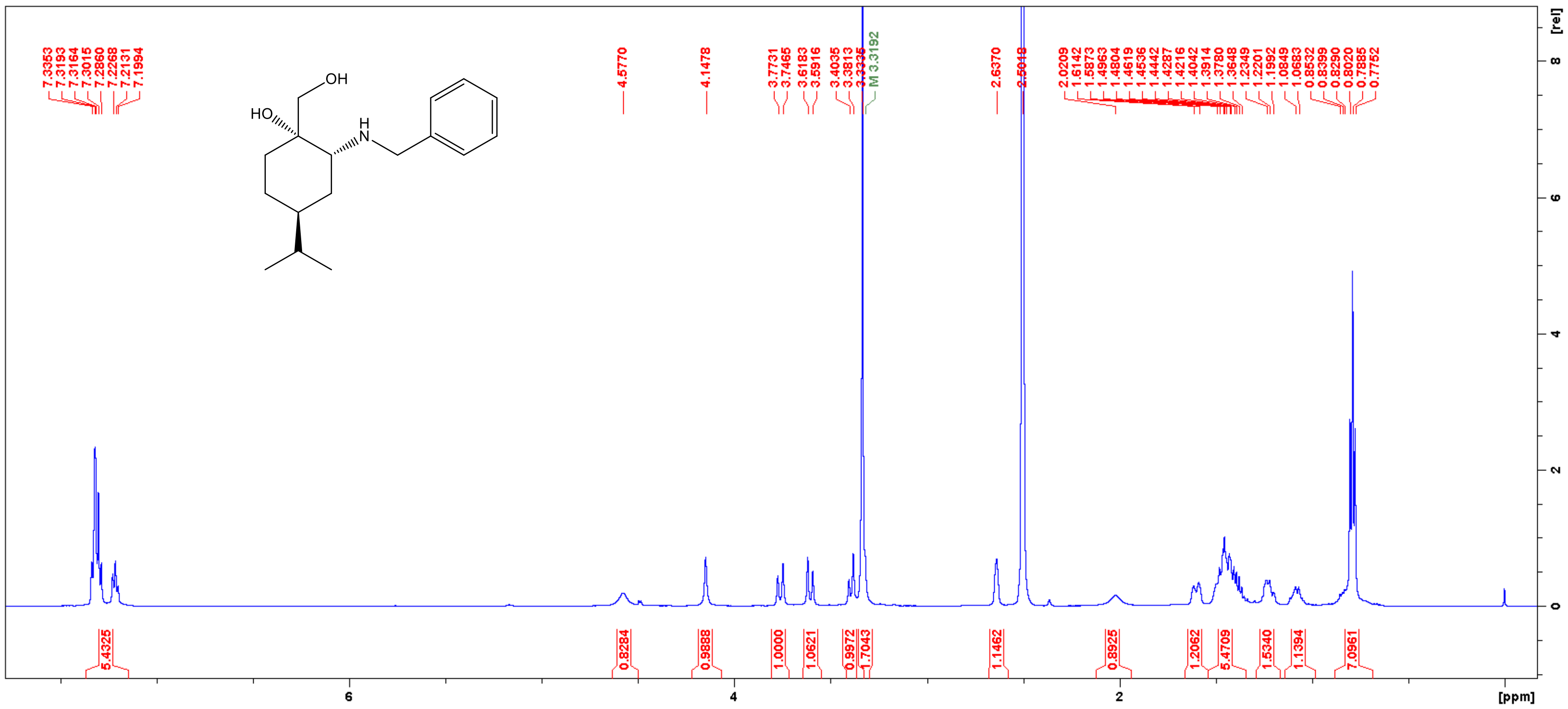

Figure S 121: ^{13}C-NMR of compound (1*S*,2*R*,4*S*)-2-Benzylamino-1-hydroxymethyl-4-isopropylcyclohexanol **23a**

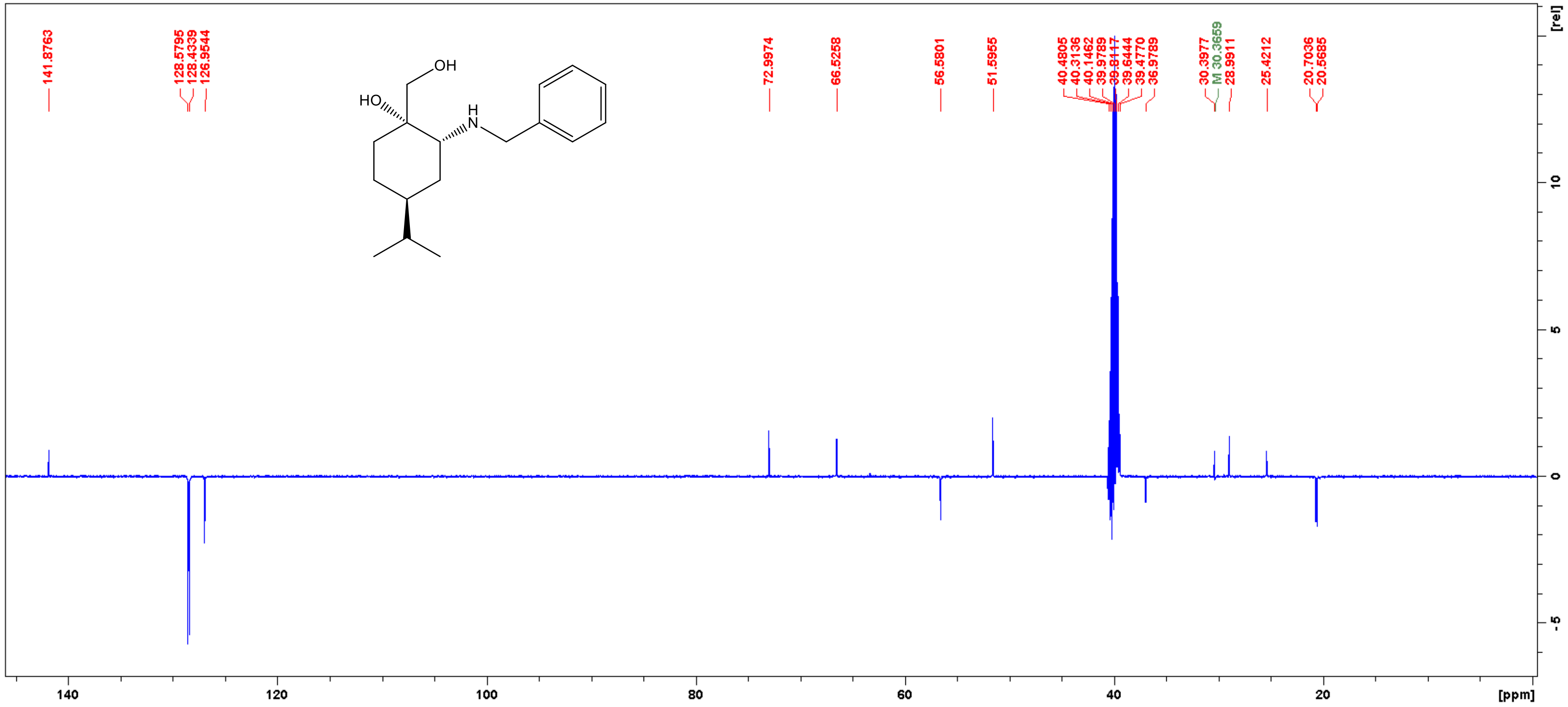

Figure S 122: COSY NMR of compound (1*S*,2*R*,4*S*)-2-Benzylamino-1-hydroxymethyl-4-isopropylcyclohexanol **23a**

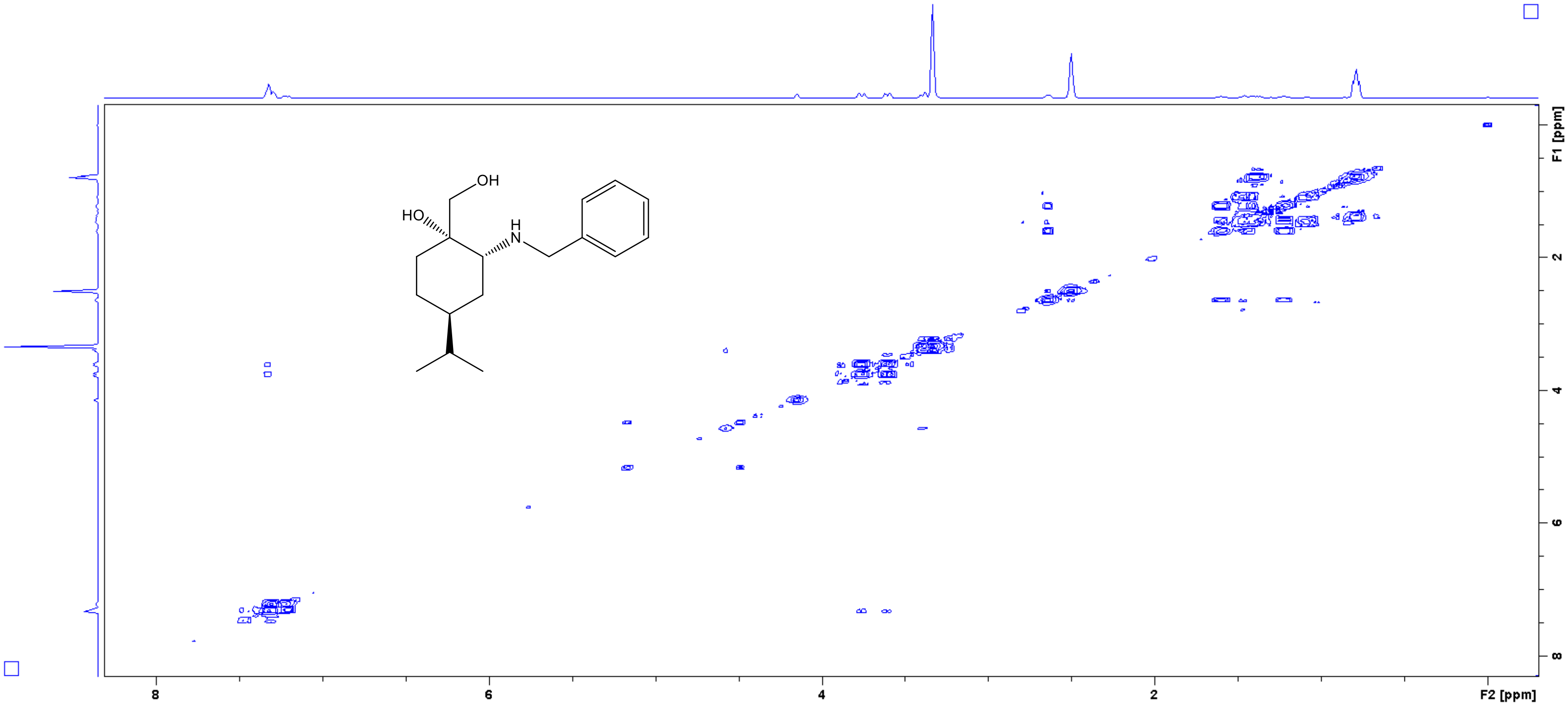

Figure S 123: NOESY NMR of compound (1*S*,2*R*,4*S*)-2-Benzylamino-1-hydroxymethyl-4-isopropylcyclohexanol **23a**

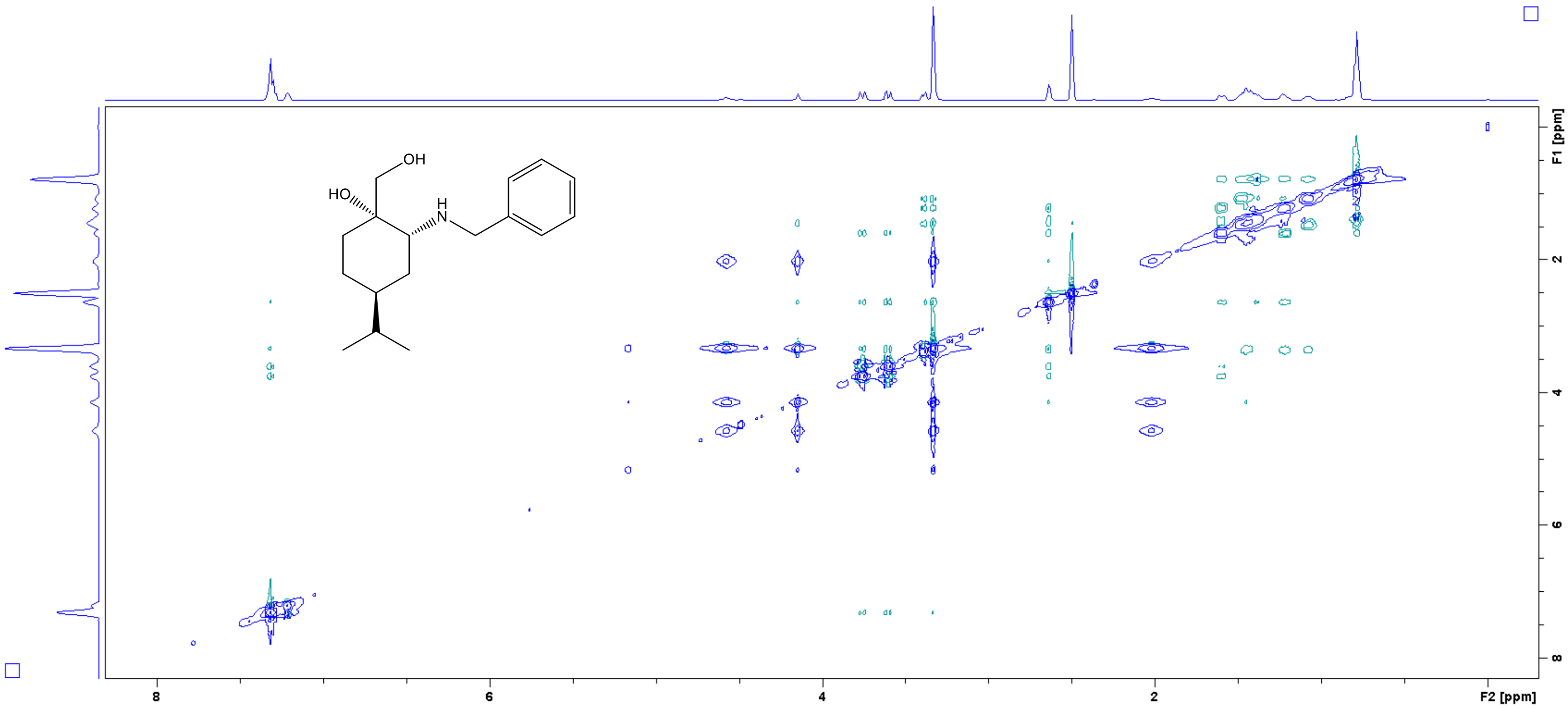

Figure S 124: HSQC NMR of compound (1*S*,2*R*,4*S*)-2-Benzylamino-1-hydroxymethyl-4-isopropylcyclohexanol **23a**

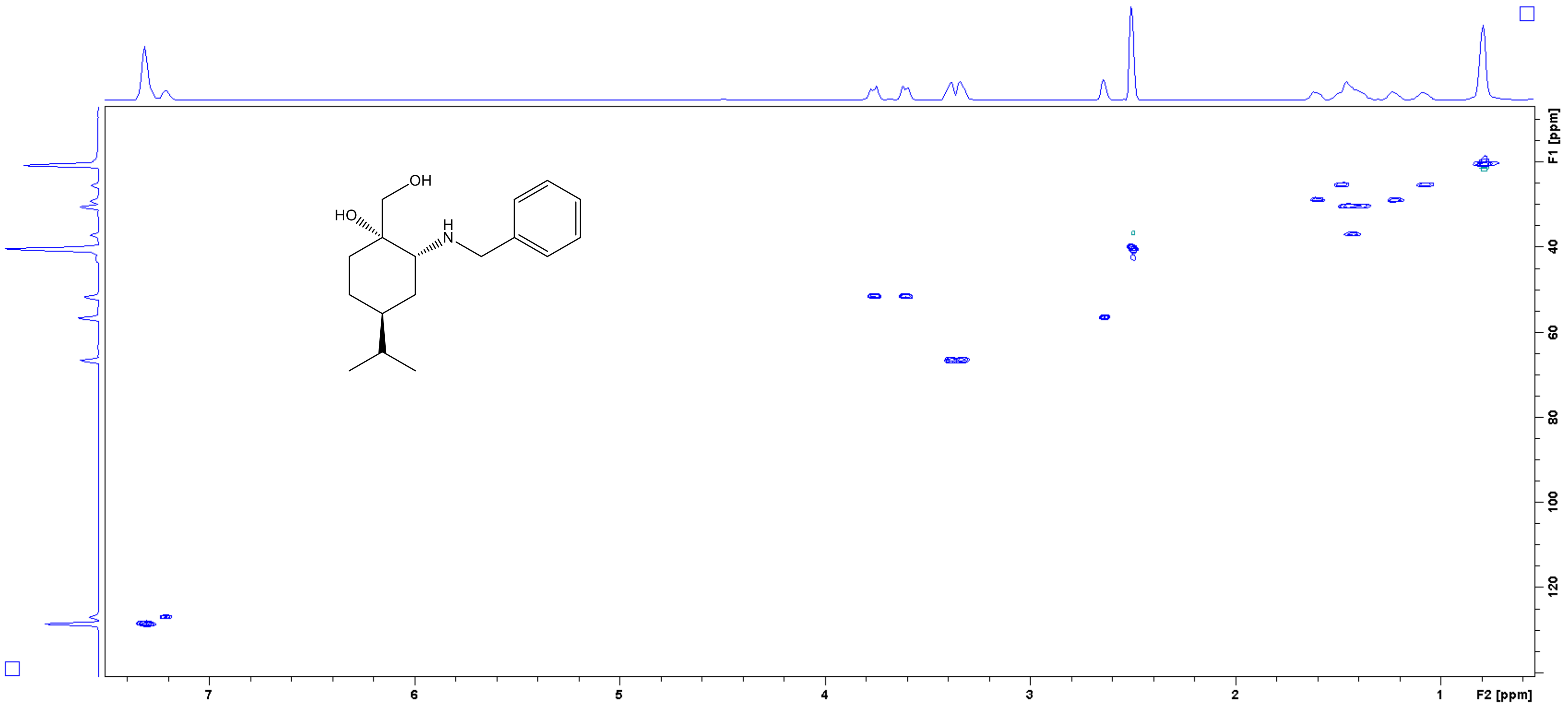

Figure S 125: ^{1}H-NMR of compound (1*R*,2*R*,4*S*)-2-Benzylamino-1-hydroxymethyl-4-isopropylcyclohexanol **23b**

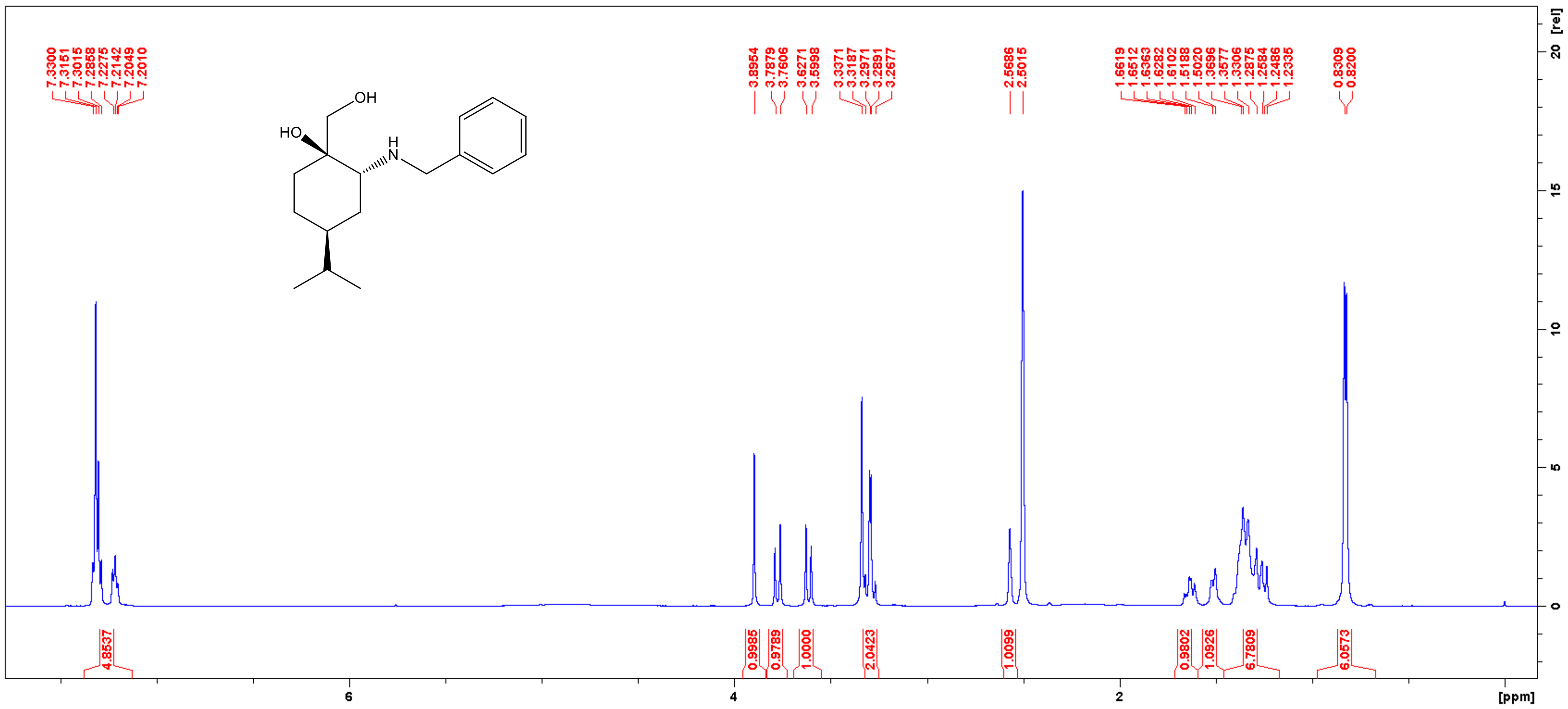

Figure S 125: ^{13}C-NMR of compound (1*R*,2*R*,4*S*)-2-Benzylamino-1-hydroxymethyl-4-isopropylcyclohexanol **23b**

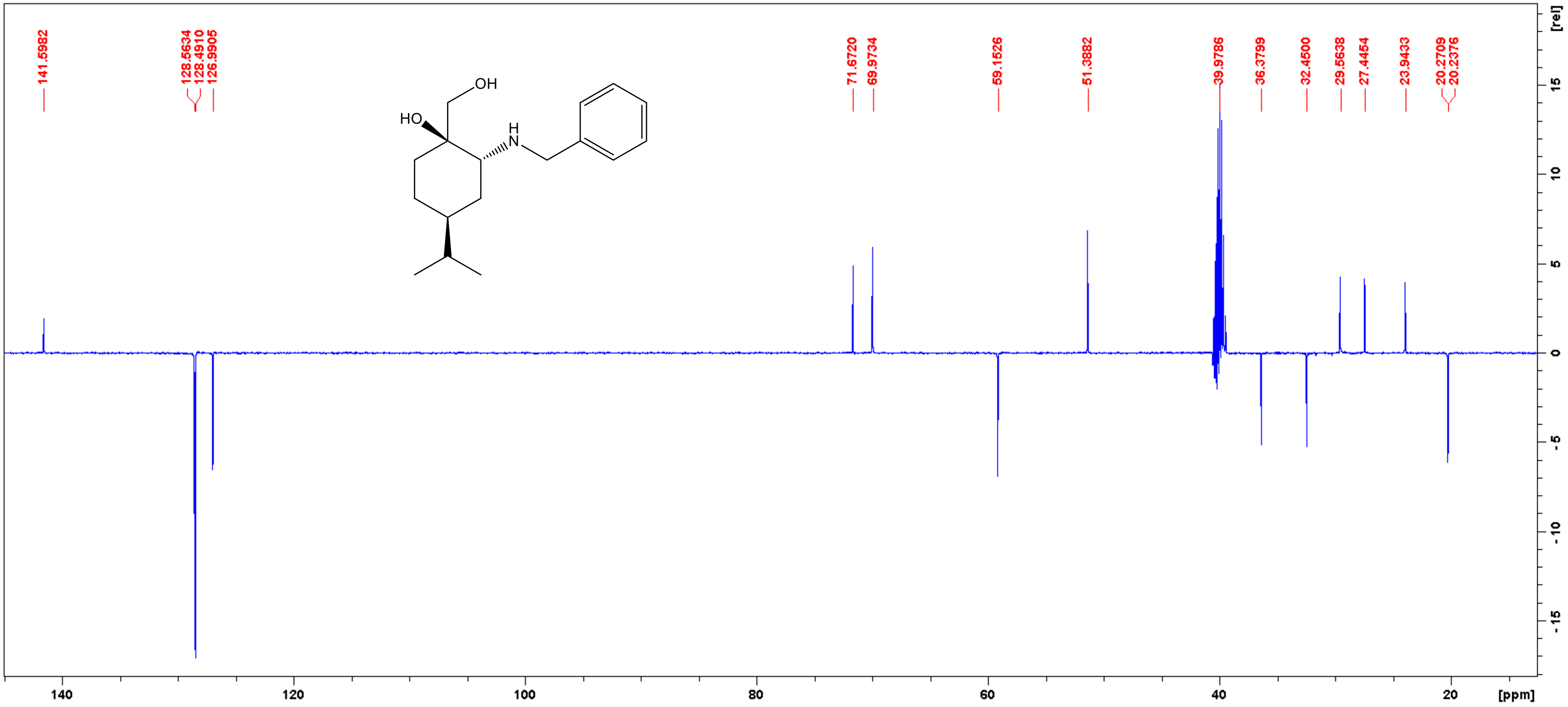

Figure S 127: COSY NMR of compound (1*R*,2*R*,4*S*)-2-Benzylamino-1-hydroxymethyl-4-isopropylcyclohexanol **23b**

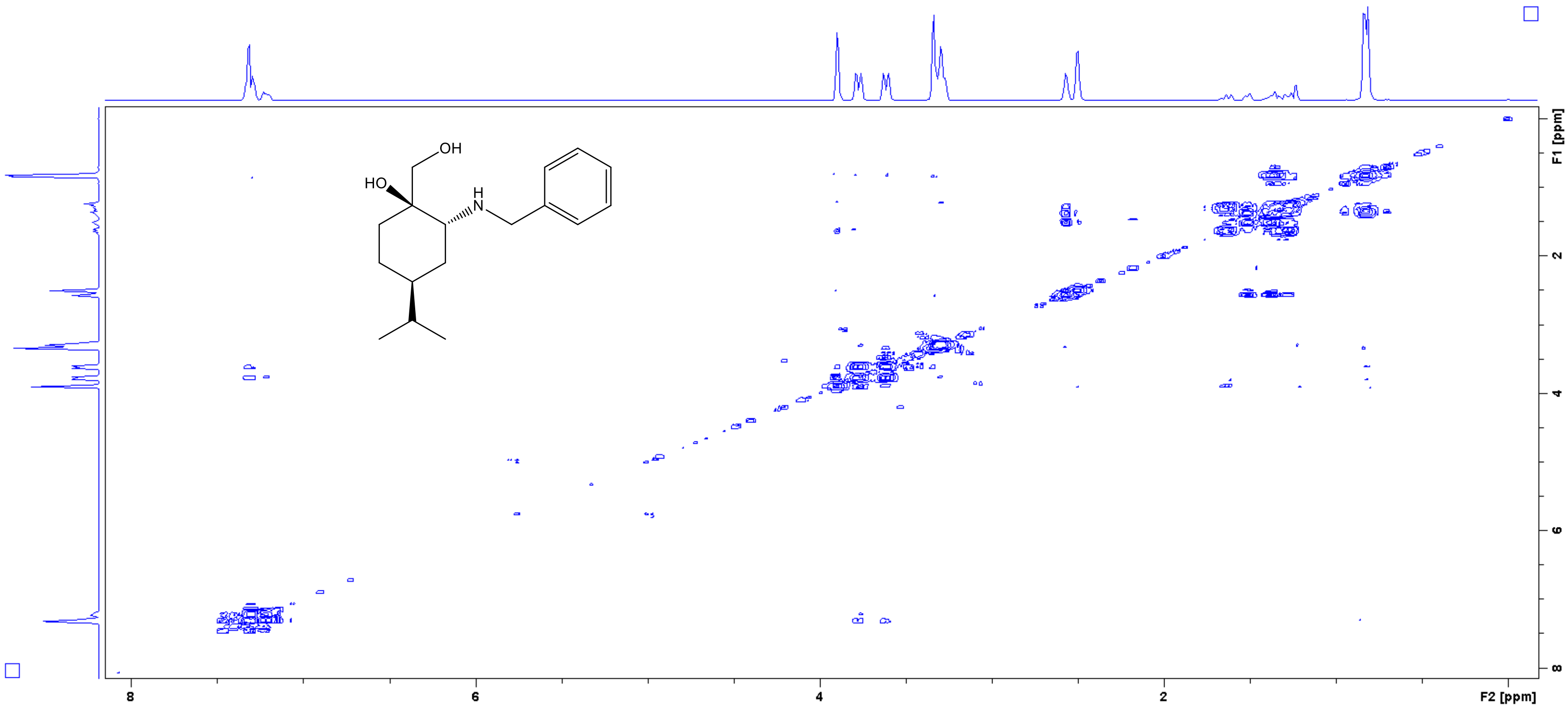

Figure S 128: NOESY NMR of compound (1*R*,2*R*,4*S*)-2-Benzylamino-1-hydroxymethyl-4-isopropylcyclohexanol **23b**

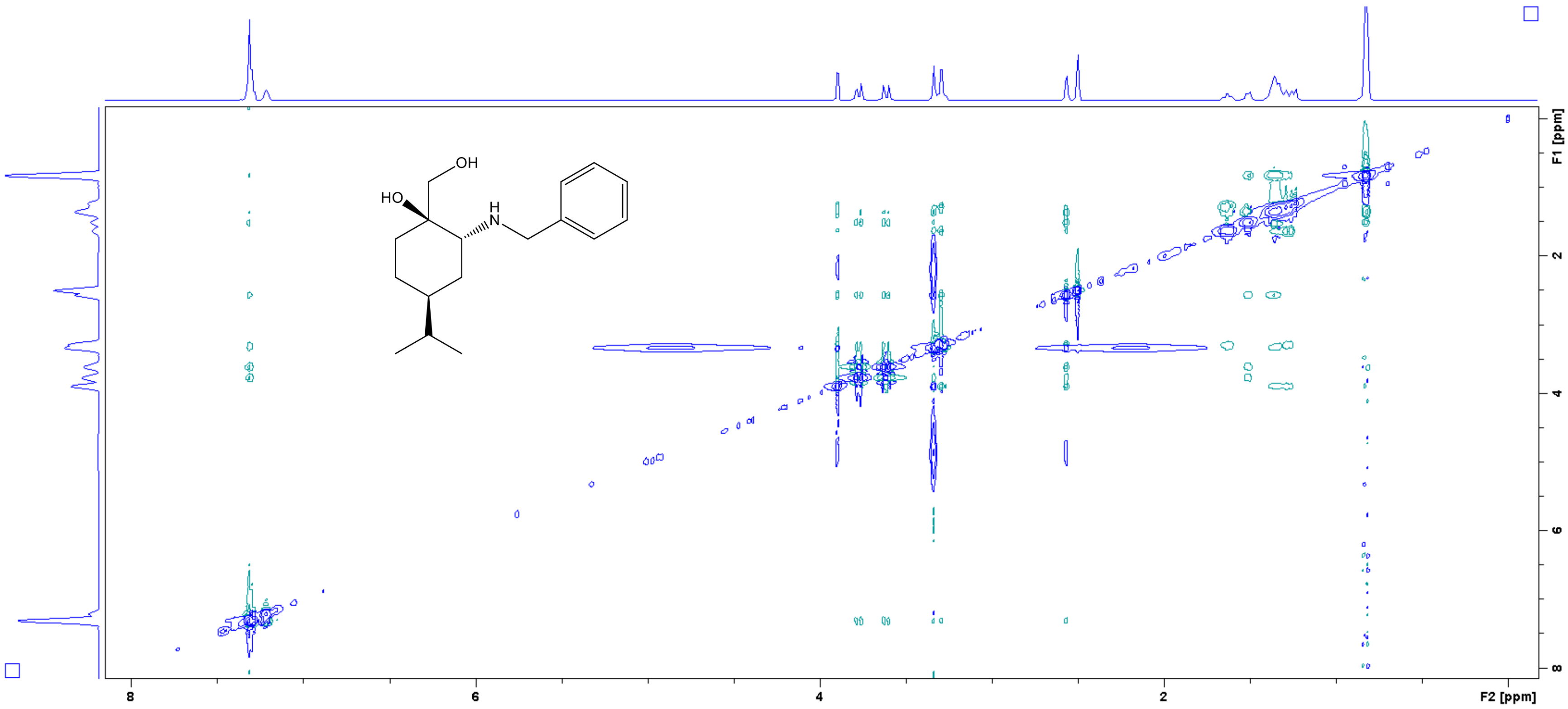

Figure S 129: HSQC NMR of compound (1*R*,2*R*,4*S*)-2-Benzylamino-1-hydroxymethyl-4-isopropylcyclohexanol **23b**

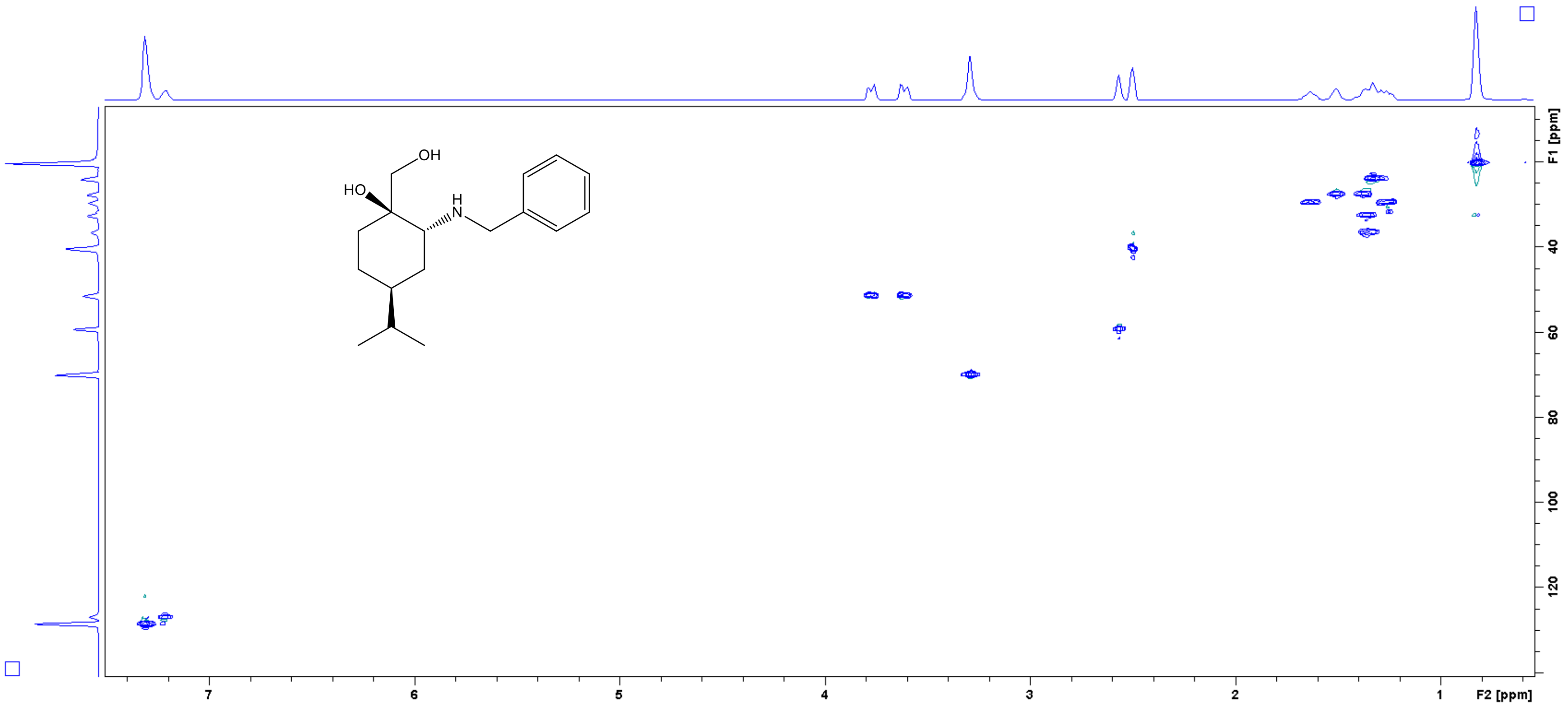

Figure S 130: HMBC NMR of compound (1*R*,2*R*,4*S*)-2-Benzylamino-1-hydroxymethyl-4-isopropylcyclohexanol **23b**

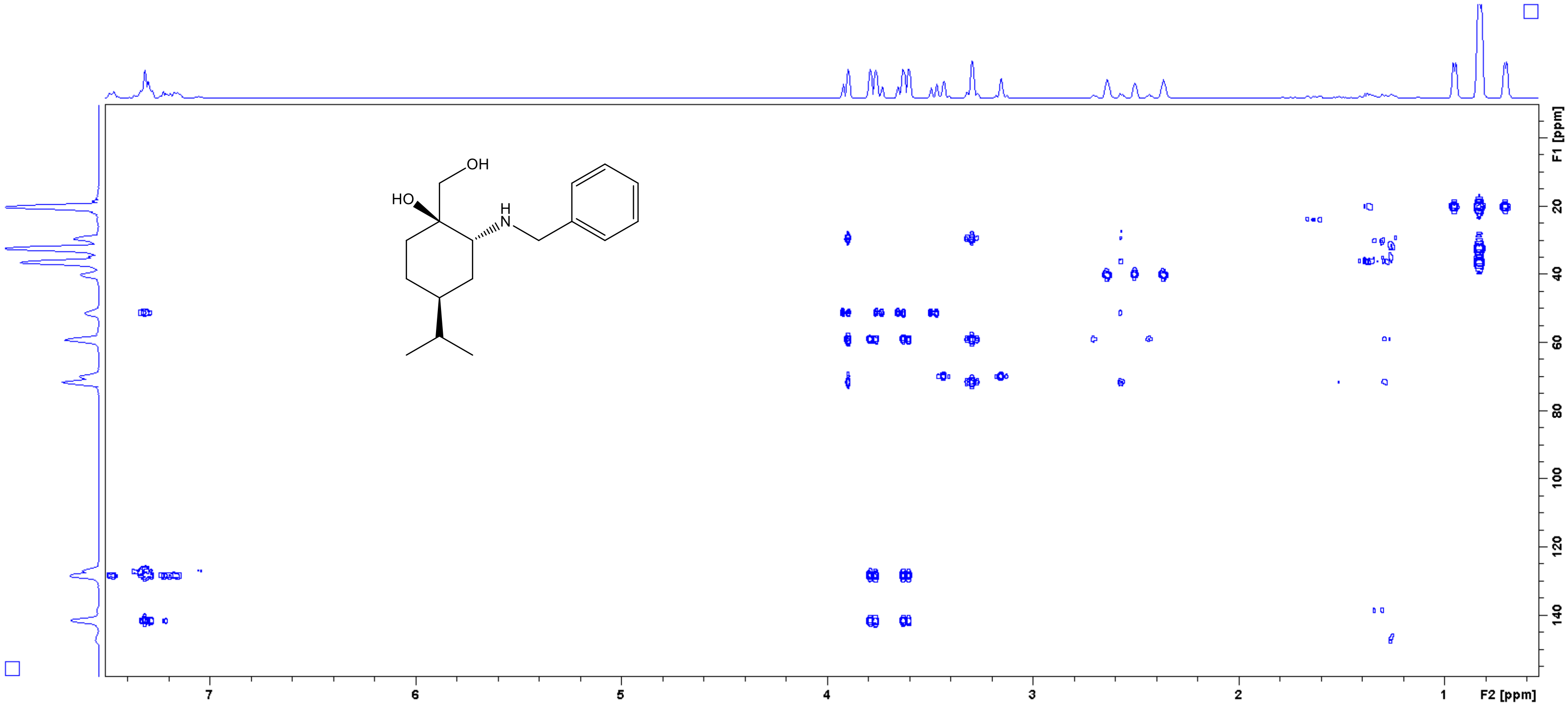

Figure S 131: ^{1}H-NMR of compound (1*S*,2*R*,4*S*)-1-(Hydroxymethyl)-4-isopropyl-2-(methylamino)cyclohexanol hydrocloride **22**

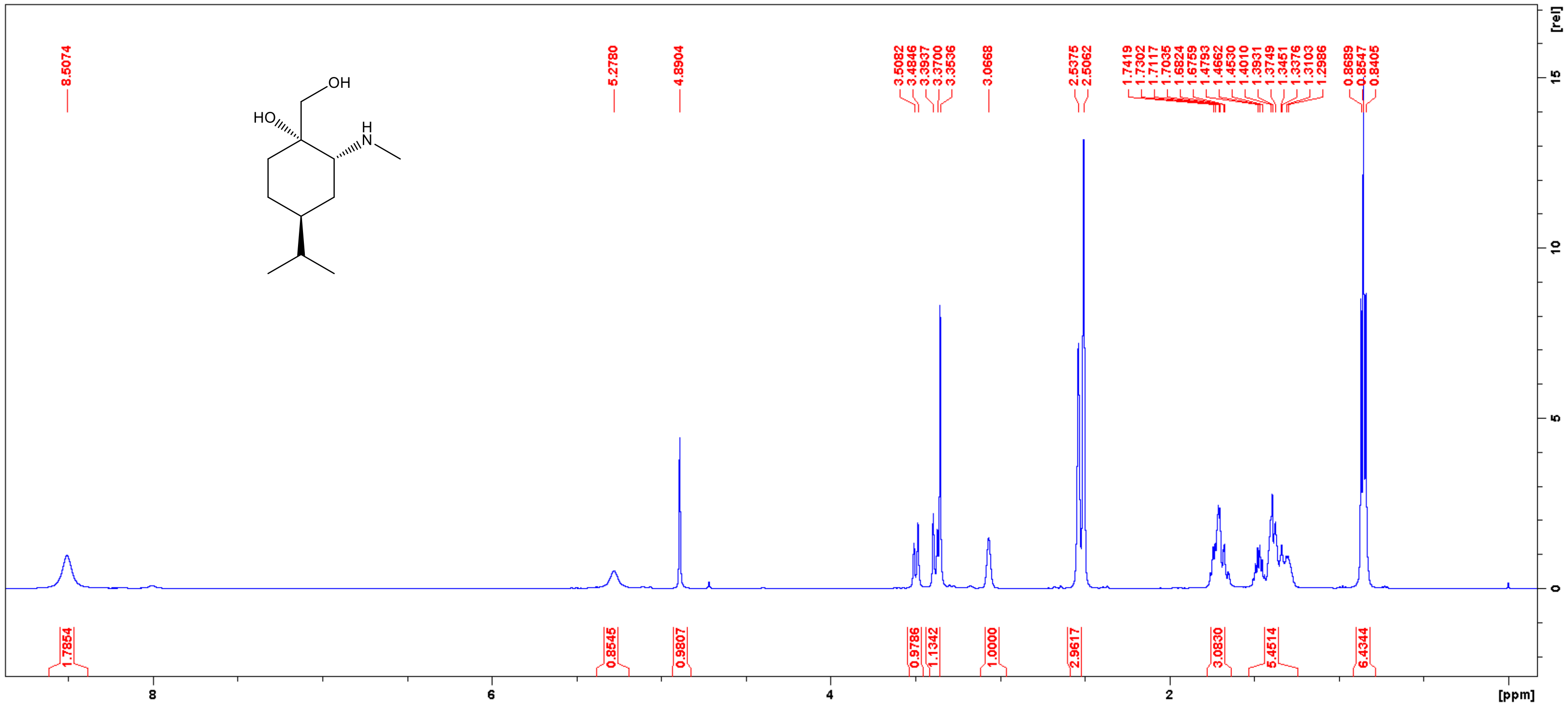

Figure S 132: ^{13}C-NMR of compound (1*S*,2*R*,4*S*)-1-(Hydroxymethyl)-4-isopropyl-2-(methylamino)cyclohexanol hydrocloride **22**

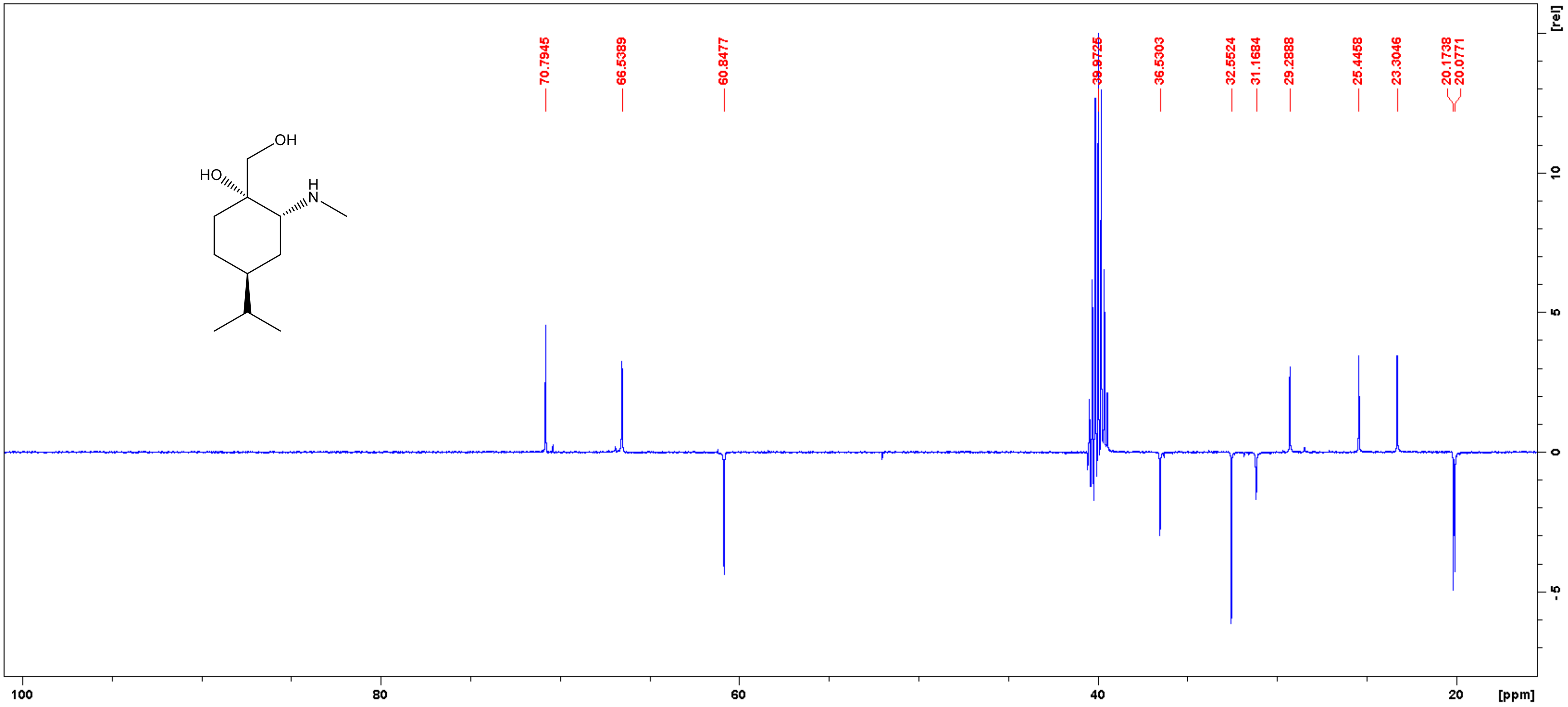

Figure S 133: COSY NMR of compound (1*S*,2*R*,4*S*)-1-(Hydroxymethyl)-4-isopropyl-2-(methylamino)cyclohexanol hydrocloride **22**

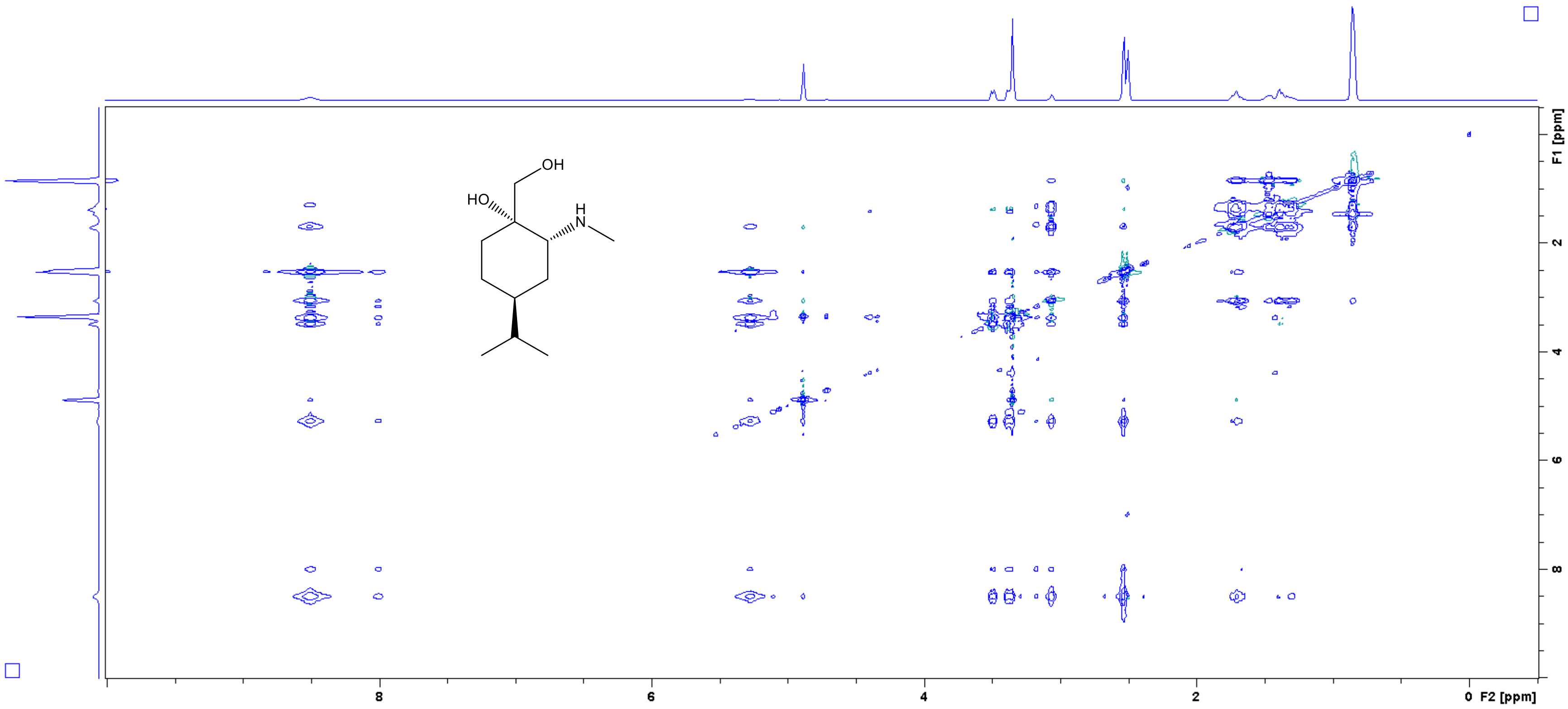

Figure S 134: HSQC NMR of compound (1*S*,2*R*,4*S*)-1-(Hydroxymethyl)-4-isopropyl-2-(methylamino)cyclohexanol hydrocloride **22**

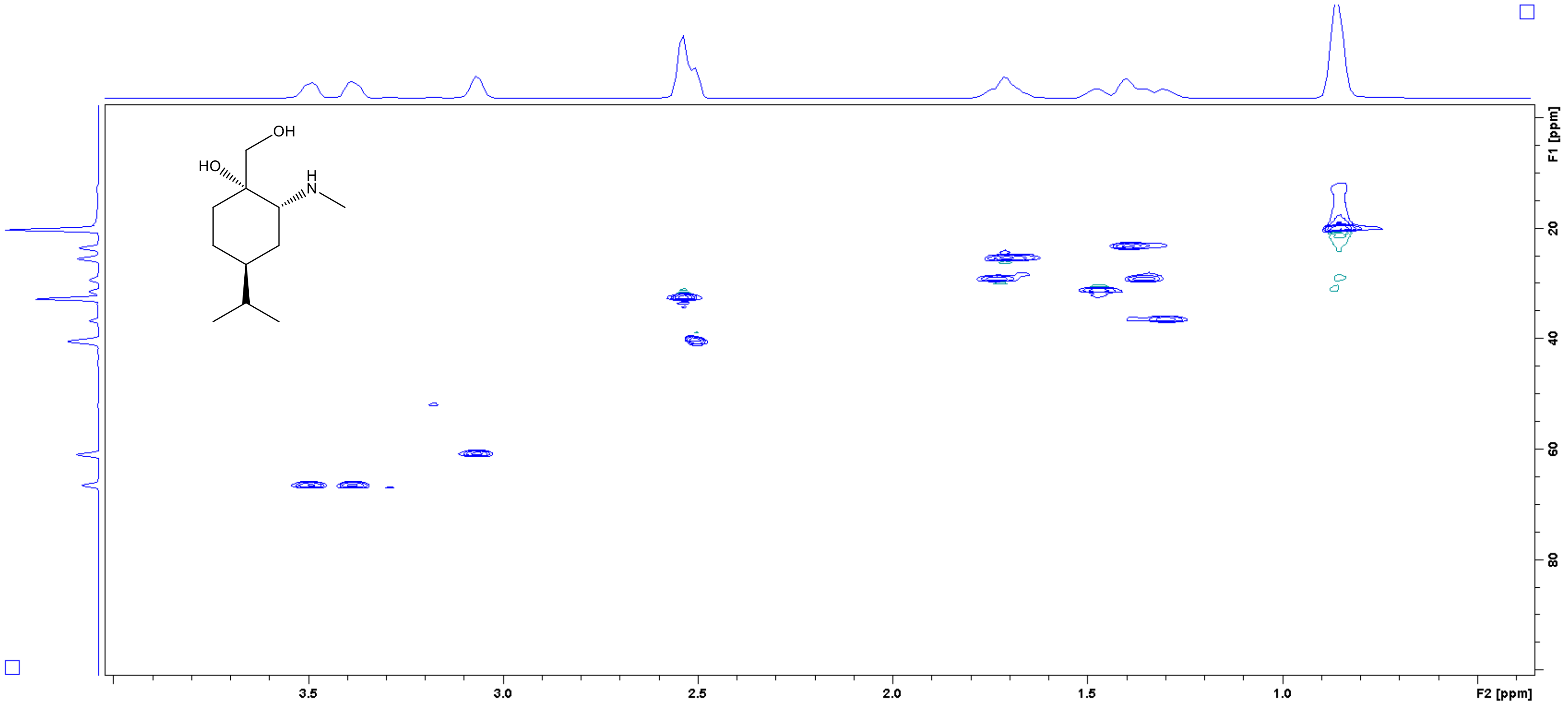

Figure S 135: ^{1}H-NMR of compound (4a*S*,7*S*,8a*R*)-1-Benzyl-7-isopropyloctahydro-1*H*-benzo[d][1,3]oxazine-4a-ol **25a**

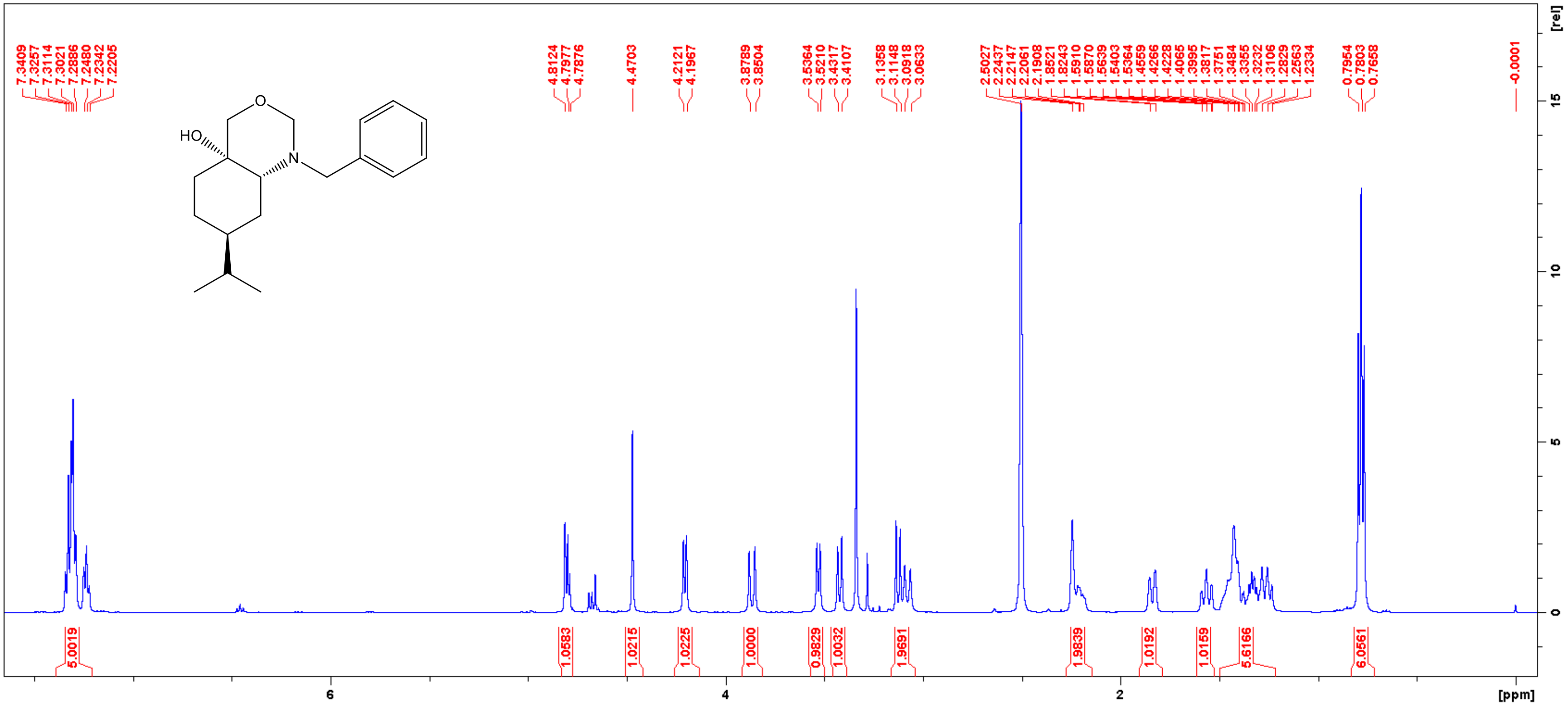

Figure S 136: ^{13}C-NMR of compound (4a*S*,7*S*,8a*R*)-1-Benzyl-7-isopropyloctahydro-1*H*-benzo[d][1,3]oxazine-4a-ol **25a**

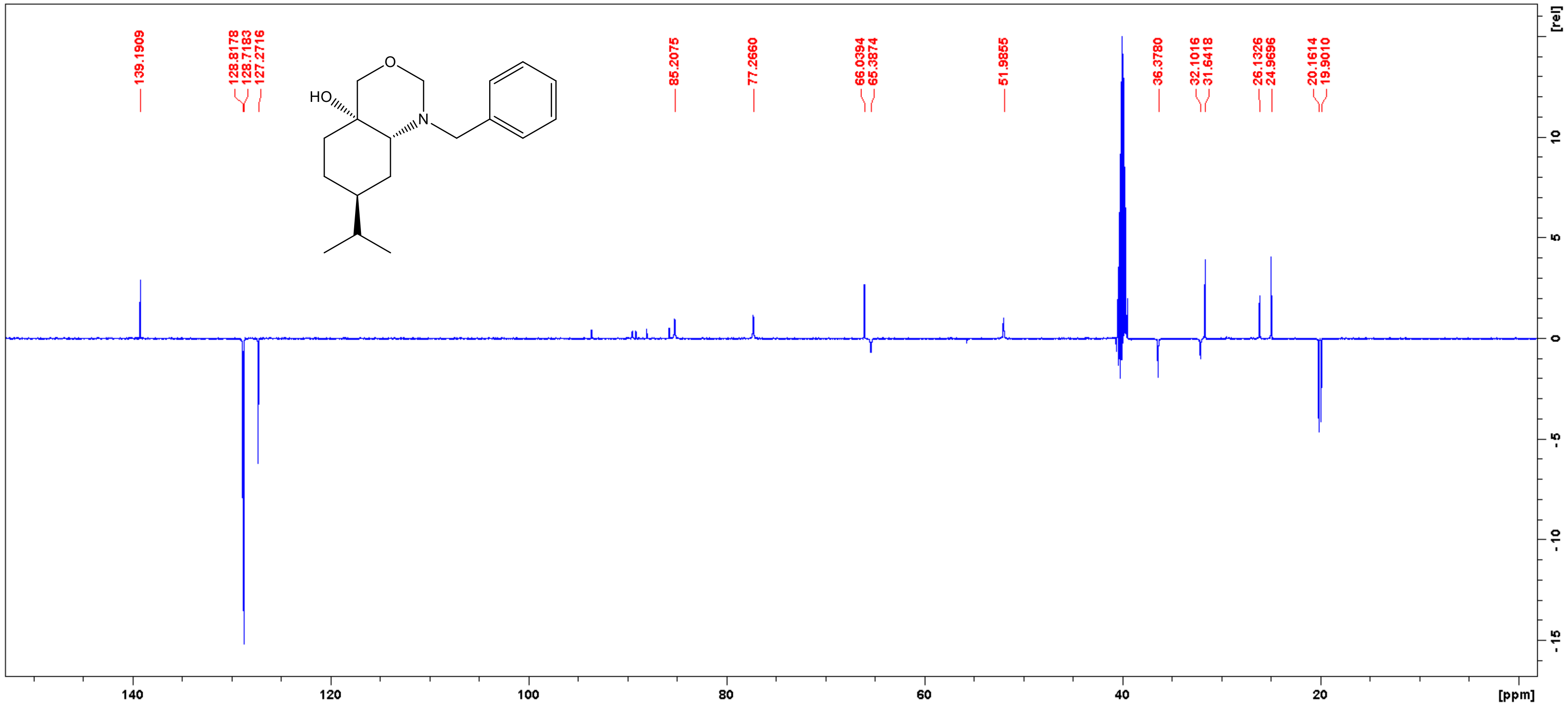

Figure S 137: COSY NMR of compound (4a*S*,7*S*,8a*R*)-1-Benzyl-7-isopropyloctahydro-1*H*-benzo[d][1,3]oxazine-4a-ol **25a**

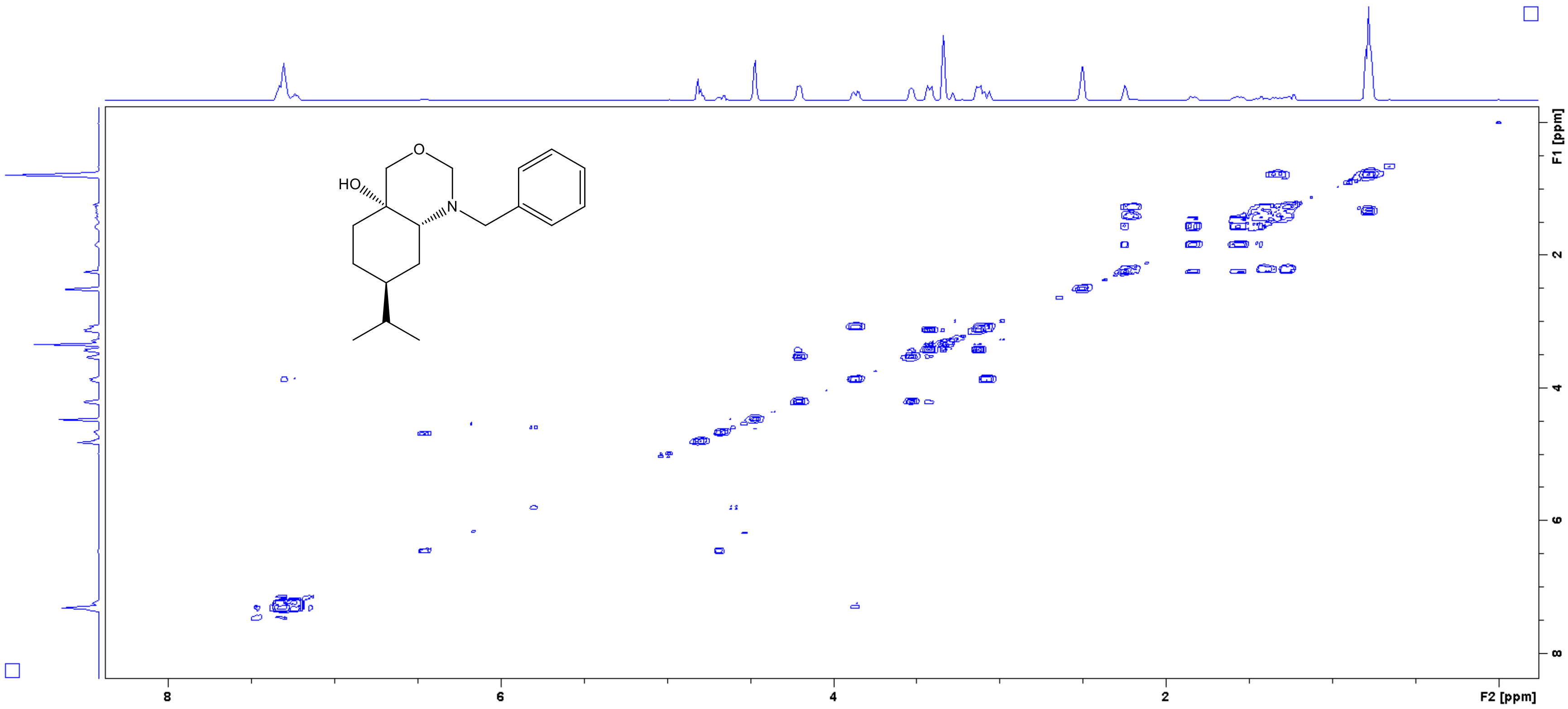

Figure S 138: HSQC NMR of compound (4a*S*,7*S*,8a*R*)-1-Benzyl-7-isopropyloctahydro-1*H*-benzo[d][1,3]oxazine-4a-ol **25a**

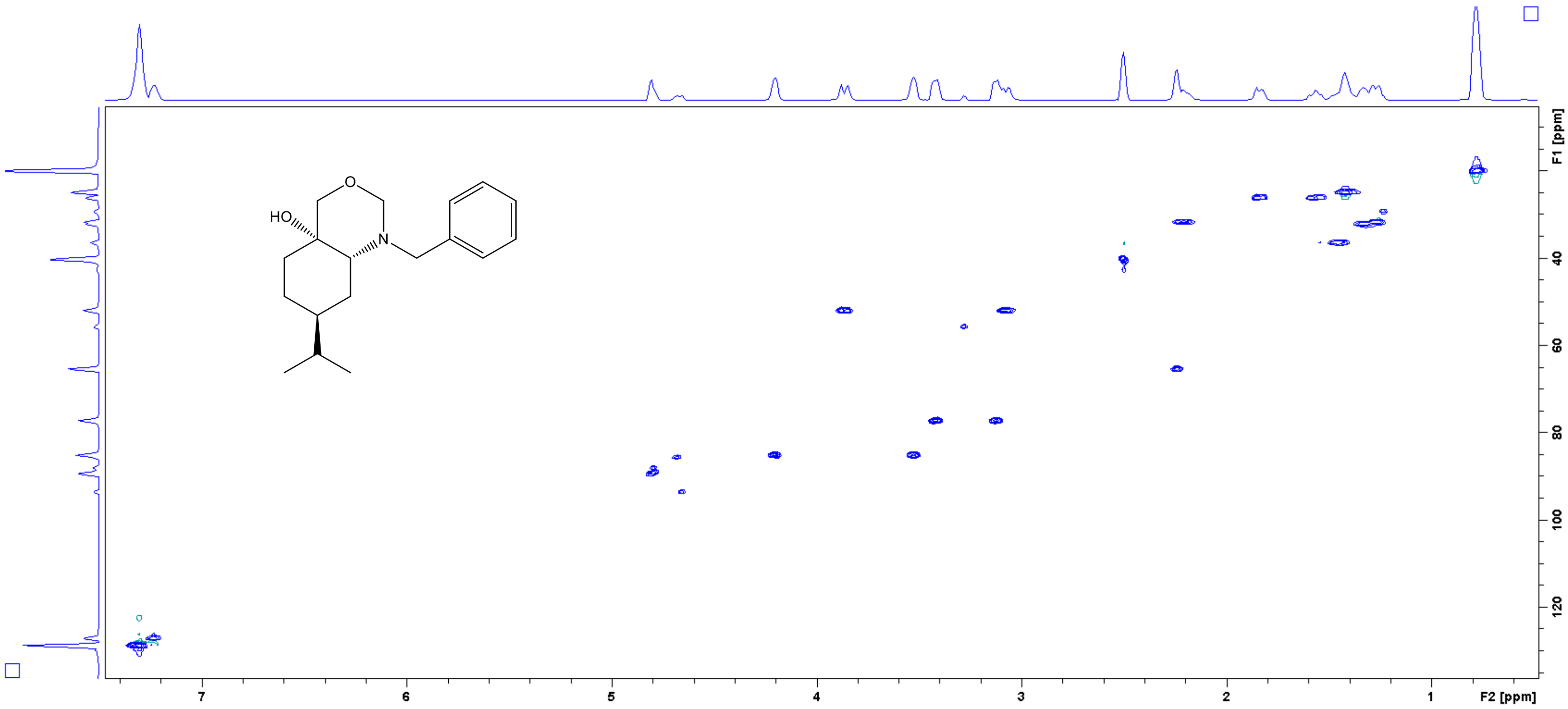

Figure S 139: ^{1}H-NMR of compound (4a*R*,7*S*,8a*R*)-1-Benzyl-7-isopropyloctahydro-1*H*-benzo[d][1,3]oxazine-4a-ol **25b**

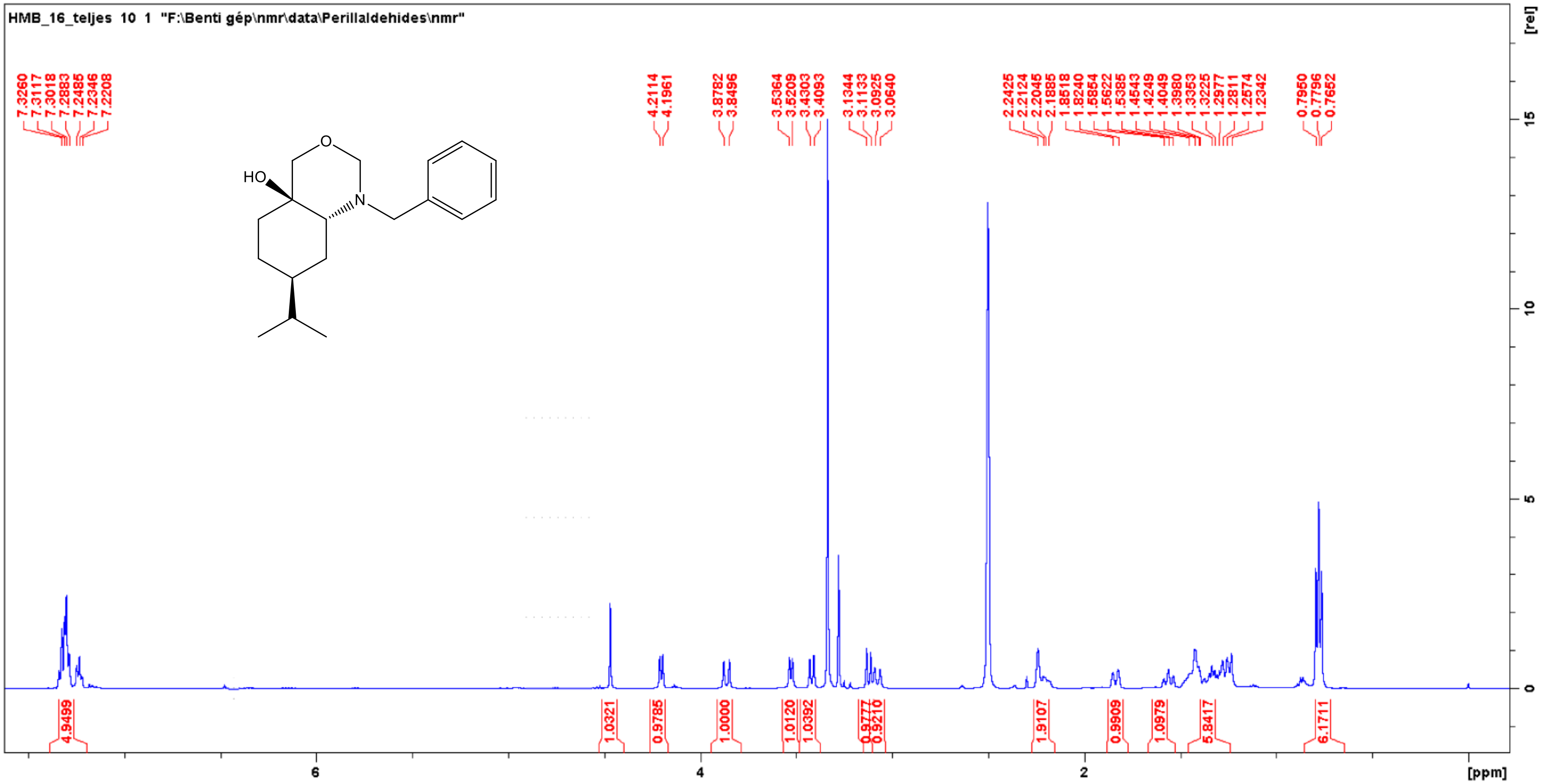

Figure S 140: ^{13}C-NMR of compound (4a*R*,7*S*,8a*R*)-1-Benzyl-7-isopropyloctahydro-1*H*-benzo[d][1,3]oxazine-4a-ol **25b**

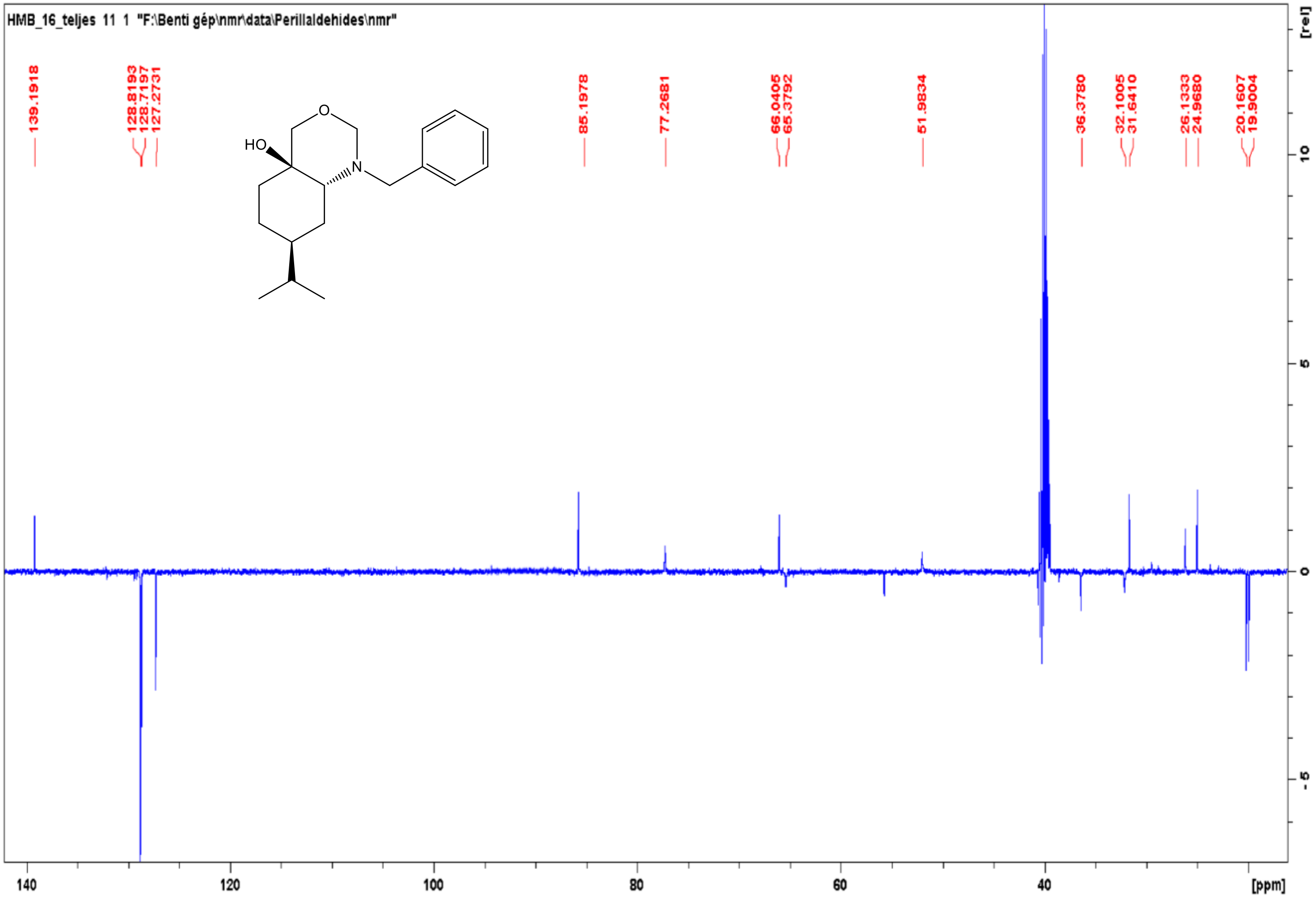

Figure S 141: ^{1}H-NMR of compound (4a*S*,7*S*,8a*R*)-7-Isopropyl-1-methyloctahyro-1*H*-benzo[d][1,3]oxazine-4a-ol **24**

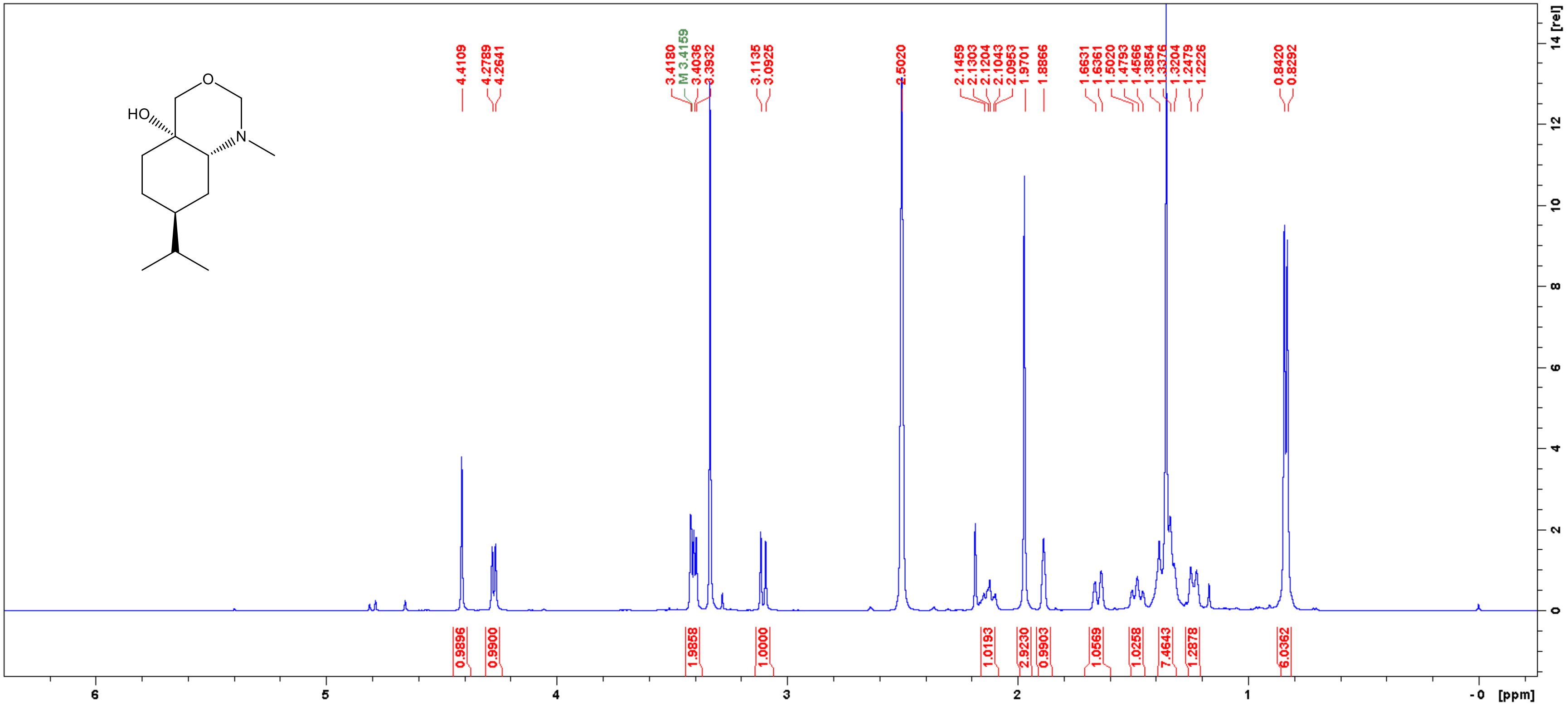

Figure S 142: ^{13}C-NMR of compound (4a*S*,7*S*,8a*R*)-7-Isopropyl-1-methyloctahyro-1*H*-benzo[d][1,3]oxazine-4a-ol **24**

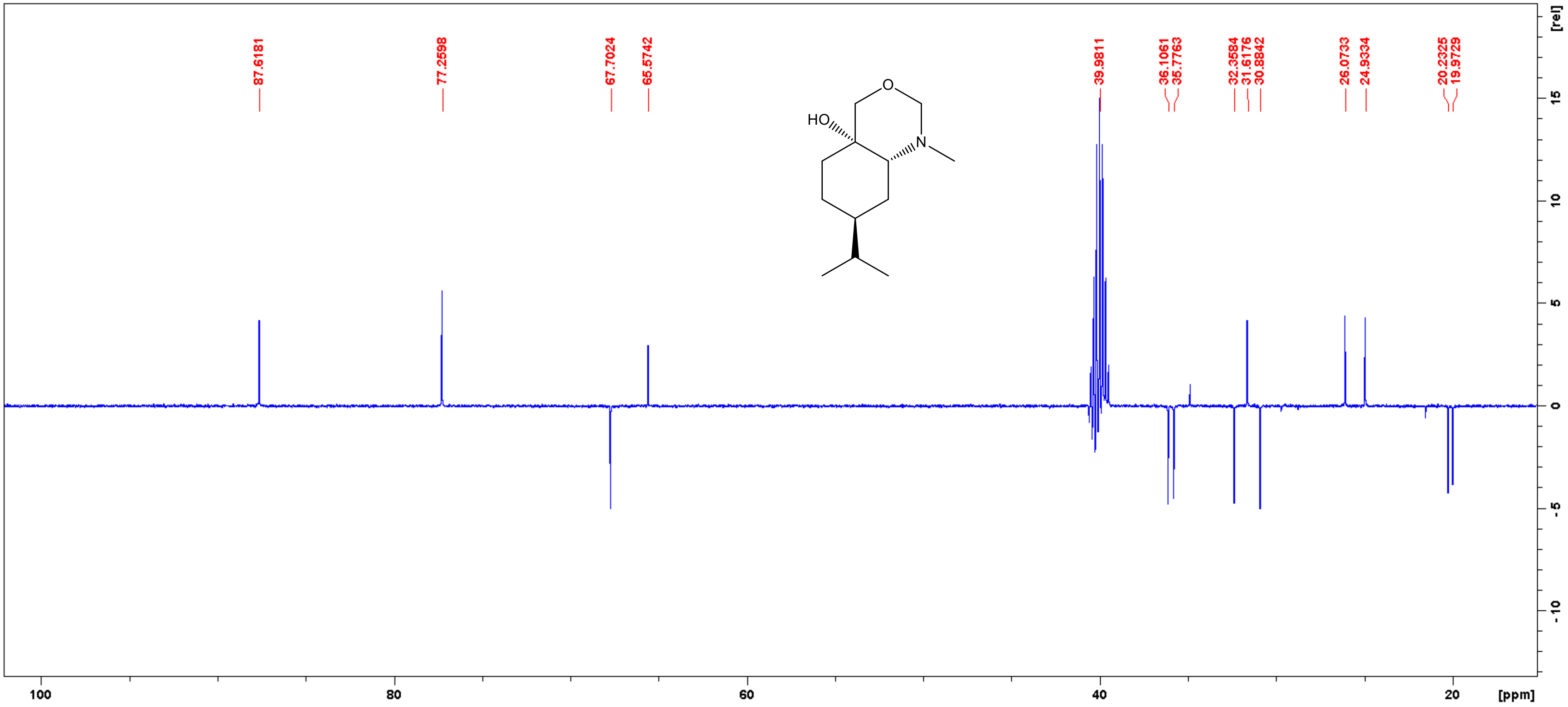

Figure S 143: COSY NMR of compound (4a*S*,7*S*,8a*R*)-7-Isopropyl-1-methyloctahyro-1*H*-benzo[d][1,3]oxazine-4a-ol **24**

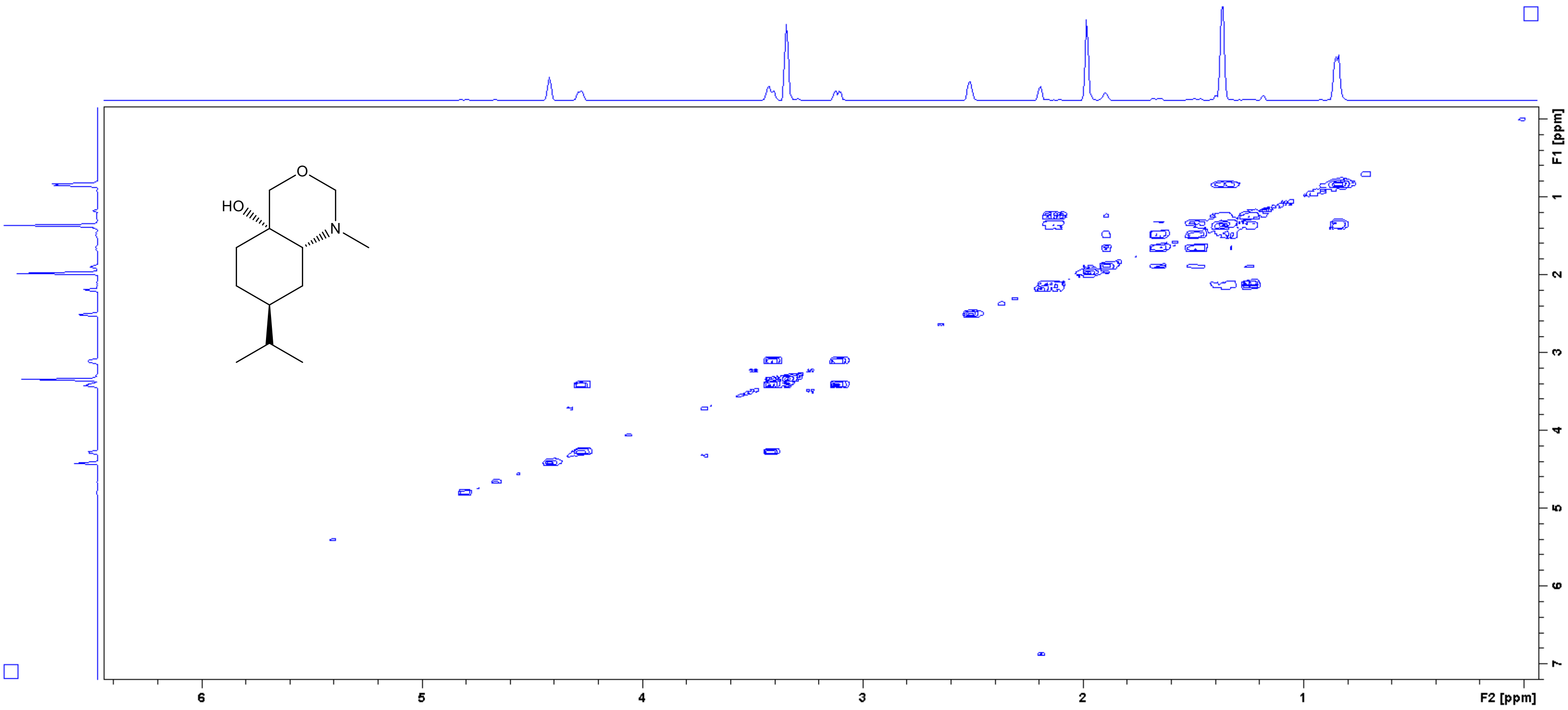

Figure S 144: HSQC NMR of compound (4a*S*,7*S*,8a*R*)-7-Isopropyl-1-methyloctahyro-1*H*-benzo[d][1,3]oxazine-4a-ol **24**

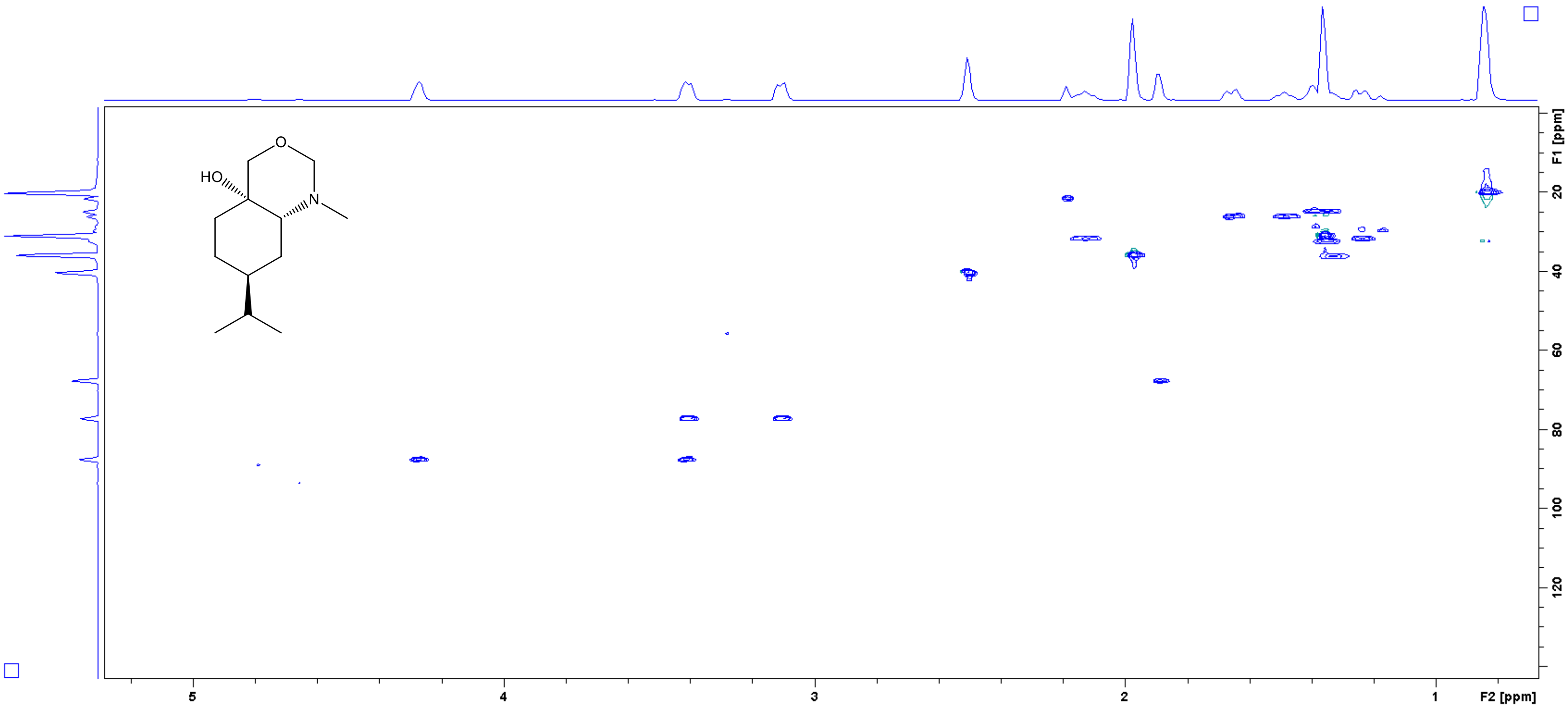

Figure S 145: Chiral GC chromatogram of the mixture 16a and 16b

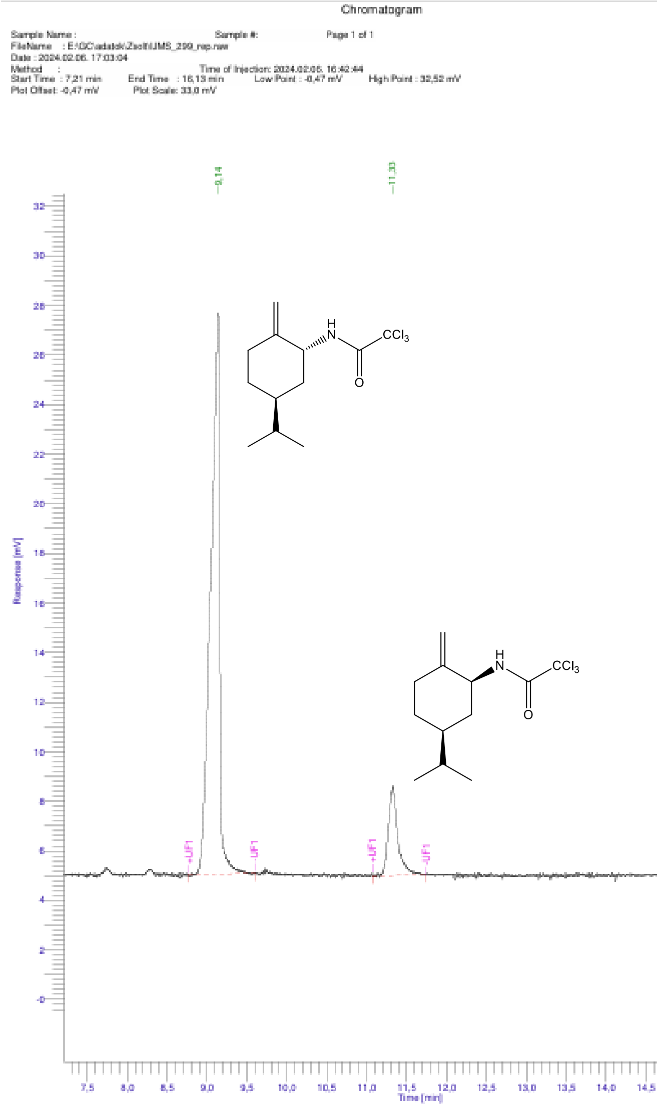

Software Version	: 6.3.2.0646	Date	: 2024.02.06. 17:02:57
Operator	: manager	Sample Name	:
Sample Number	:	Study	:
AutoSampler	: NONE	Rack/Vial	: 0/0
Instrument Name	: AutosystemXL	Channel	: A
Instrument Serial #	: 610N9042106	A/D mV Range	: 1000
Delay Time	: 0,00 min	End Time	: 16,24 min
Sampling Rate	: 12,5000 pts/s		
Sample Volume	: 1,000000 ul	Area Reject	: 0,000000
Sample Amount	: 1,0000	Dilution Factor	: 1,00
Data Acquisition Time	: 2024.02.06. 16:42:44	Cycle	: 1

Raw Data File : E:\GC\adatok\Zsolt\IJMS_299_rep.raw
Result File : E:\GC\adatok\Zsolt\IJMS_299_rep.rst [Editing in Progress]
Inst Method : E:\GC\method\Palko\kondicionalas from E:\GC\adatok\Zsolt\IJMS_299_rep.raw
Proc Method : E:\GC\method\Palko\kondicionalas from E:\GC\adatok\Zsolt\IJMS_299_rep.rst [Editing in Progress]
Calib Method : E:\GC\method\Palko\kondicionalas from E:\GC\adatok\Zsolt\IJMS_299_rep.rst [Editing in Progress]
Report Format File: E:\GC\method\Palko\kondicionalas.rpt
Sequence File : E:\GC\method\IJMS_299_rep.seq

DEFAULT REPORT

Peak #	Time [min]	Area [µV·s]	Height [µV]	Area [%]	Norm. Area [%]	BL	Area/Height [s]
1	9,141	175190,17	22611,41	85,71	85,71	*MM	7,7479
2	11,325	29216,45	3584,61	14,29	14,29	*MM	8,1505
		204406,62	26196,02	100,00	100,00		

Figure S 146: Chiral GC chromatogram of the mixture 18a and 18b

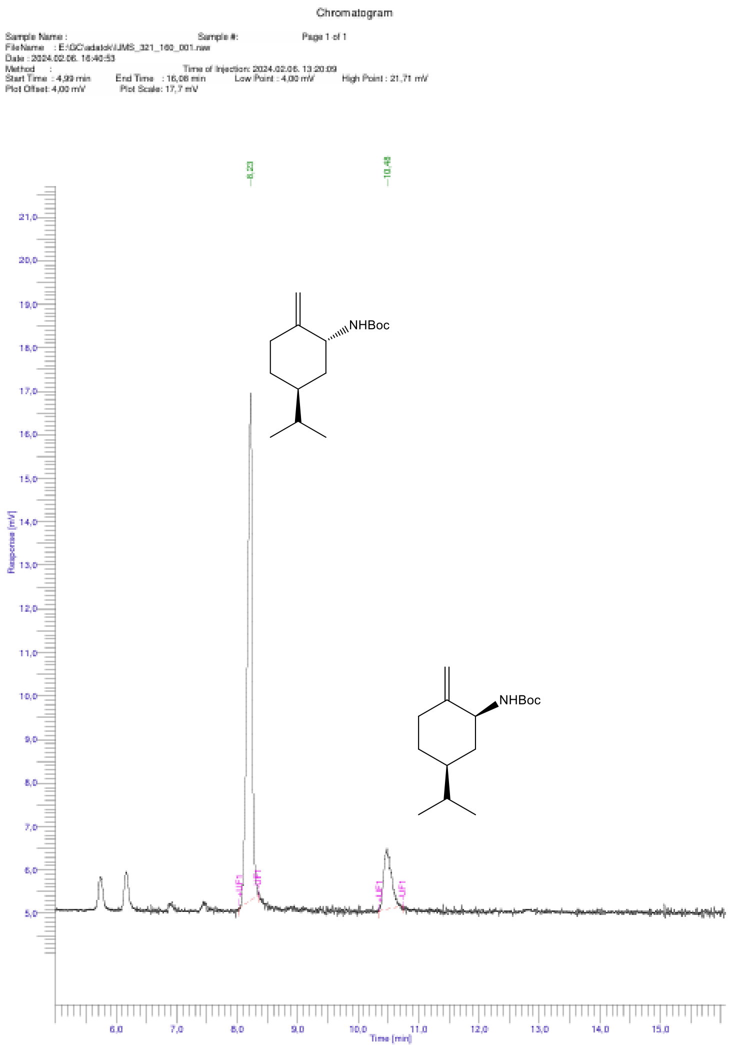

Software Version	: 6.3.2.0646	Date	: 2024.02.06. 16:42:03
Operator	: manager	Sample Name	:
Sample Number	:	Study	:
AutoSampler	: NONE	Rack/Vial	: 0/0
Instrument Name	: AutosystemXL	Channel	: A
Instrument Serial #	: 610N9042106	A/D mV Range	: 1000
Delay Time	: 0,00 min	End Time	: 30,02 min
Sampling Rate	: 12,5000 pts/s		
Sample Volume	: 1,000000 ul		
Sample Amount	: 1,0000	Area Reject	: 0,000000
Data Acquisition Time	: 2024.02.06. 13:20:09	Dilution Factor	: 1,00
		Cycle	: 1

Raw Data File : E:\GC\adatok\IJMS_321_160_001.raw
Result File : E:\GC\adatok\IJMS_321_160_001.rst [Editing in Progress]
Inst Method : E:\GC\method\Palko\kondicionalas from E:\GC\adatok\IJMS_321_160_001.raw
Proc Method : E:\GC\method\Palko\kondicionalas from E:\GC\adatok\IJMS_321_160_001.rst [Editing in Progress]
Calib Method : E:\GC\method\Palko\kondicionalas from E:\GC\adatok\IJMS_321_160_001.rst [Editing in Progress]
Report Format File: E:\GC\method\Palko\kondicionalas.rpt
Sequence File : E:\GC\method\IJMS_323A_160-20240206-131808.seq

DEFAULT REPORT

Peak #	Time [min]	Area [µV·s]	Height [µV]	Area [%]	Norm. Area [%]	BL	Area/Height [s]
1	8,228	66155,67	11647,80	84,59	84,59	*MM	5,6797
2	10,483	12052,12	1392,83	15,41	15,41	*MM	8,6530
		78207,79	13040,63	100,00	100,00		

Figure S 147: Chiral GC chromatogram of OAc 1-phenyl-1-propanol enantiomers

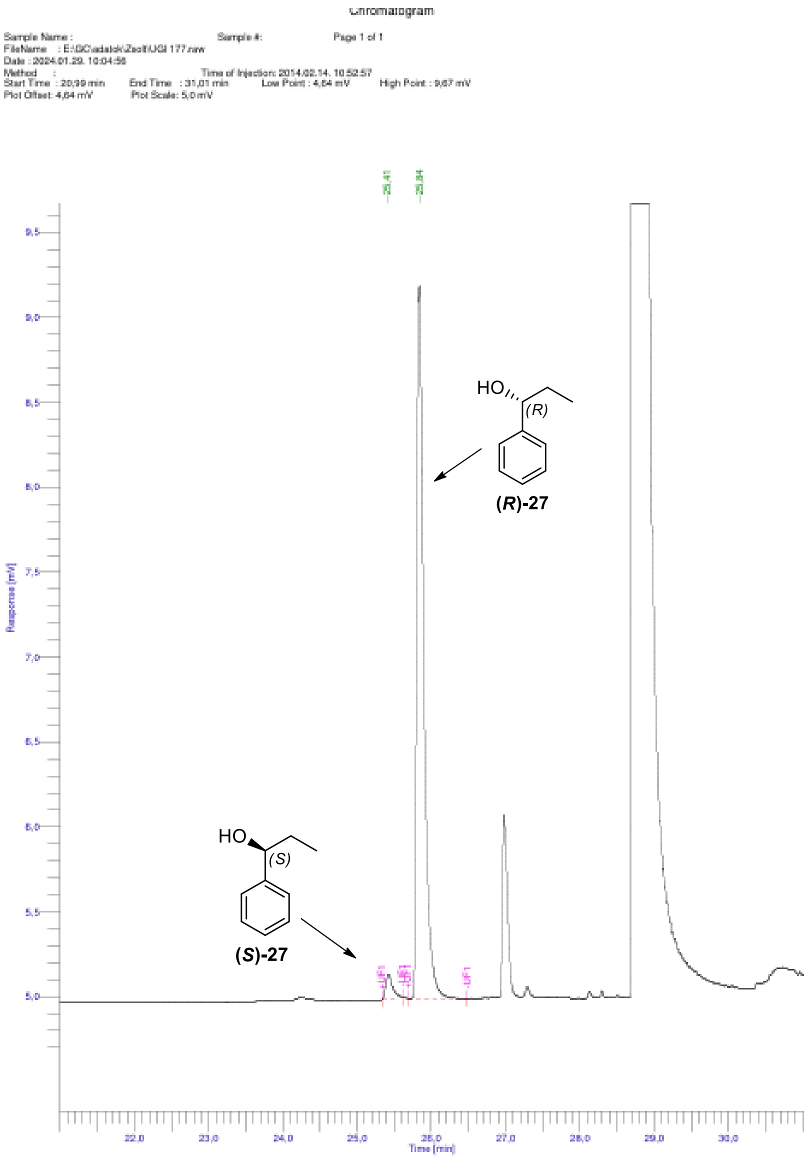

Software Version	: 6.3.2.0646	Date	: 2024.01.29. 10:07:10
Operator	: manager	Sample Name	:
Sample Number	:	Study	:
AutoSampler	: NONE	Rack/Vial	: 0/0
Instrument Name	: PerkinName	Channel	: A
Instrument Serial #	: 610N9042106	A/D mV Range	: 1000
Delay Time	: 0,00 min	End Time	: 42,00 min
Sampling Rate	: 12,5000 pts/s		
Sample Volume	: 1,000000 ul		
Sample Amount	: 1,0000	Area Reject	: 0,000000
Data Acquisition Time	: 2014.02.14. 10:52:57	Dilution Factor	: 1,00
		Cycle	: 1

Raw Data File : E:\GC\adatok\Zsolt\UGI 177.raw
Result File : E:\GC\adatok\Zsolt\UGI 177.rst [Editing in Progress]
Inst Method : D:\Doksik\Irodalom\GC\method\Zsolt\fenipropanol90 from E:\GC\adatok\Zsolt\UGI 177.raw
Proc Method : D:\Doksik\Irodalom\GC\method\Zsolt\fenipropanol90 from E:\GC\adatok\Zsolt\UGI 177.rst [Editing in Progress]
Calib Method : D:\Doksik\Irodalom\GC\method\Zsolt\fenipropanol90 from E:\GC\adatok\Zsolt\UGI 177.rst [Editing in Progress]
Report Format File: D:\Doksik\Irodalom\GC\method\Zsolt\fenipropanol90.rpt
Sequence File : D:\Doksik\Irodalom\GC\adatok\UGI 177.seq

DEFAULT REPORT

Peak #	Time [min]	Area [µV·s]	Height [µV]	Area [%]	Norm. Area [%]	BL	Area/Height [s]
1	25,415	883,78	141,53	3,06	3,06	*MM	6,2443
2	25,844	27991,38	4198,54	96,94	96,94	*MM	6,6669
		28875,16	4340,08	100,00	100,00		

Catalyst: **10a**

Figure S 148: Chiral GC chromatogram of OAc 1-phenyl-1-propanol enantiomers

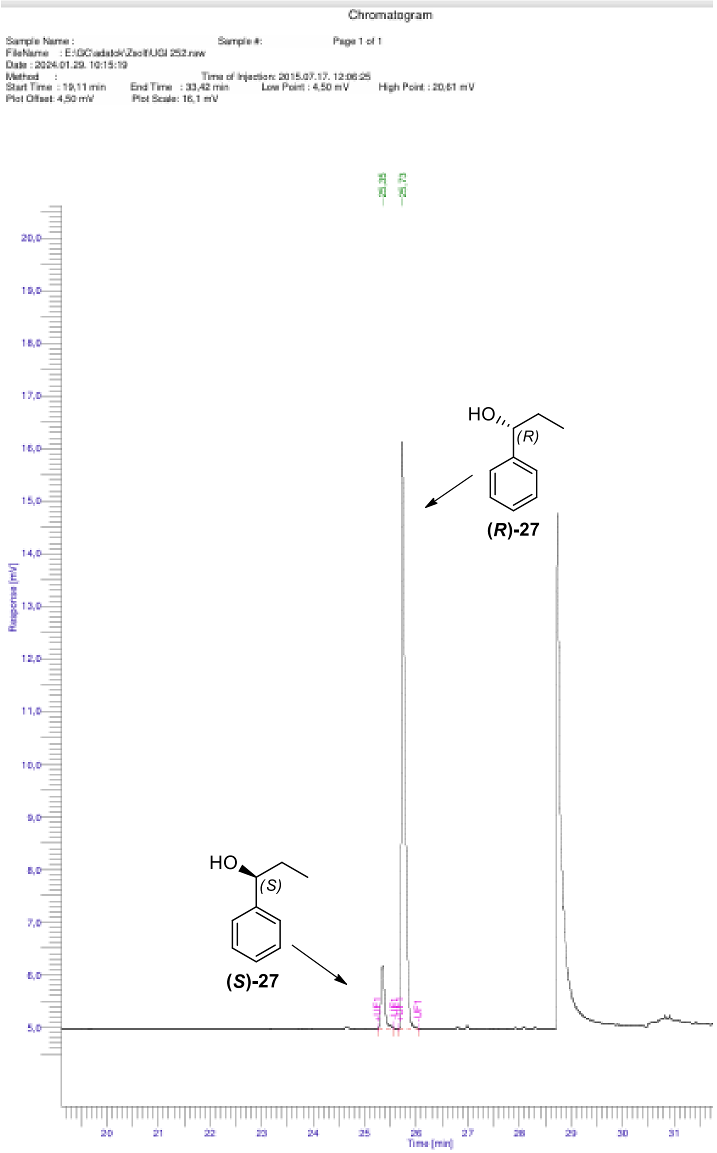

Software Version	: 6.3.2.0646	Date	: 2024.01.29. 10:14:37
Operator	: manager	Sample Name	:
Sample Number	:	Study	:
AutoSampler	: NONE	Rack/Vial	: 0/0
Instrument Name	: PerkinName	Channel	: A
Instrument Serial #	: 610N9042106	A/D mV Range	: 1000
Delay Time	: 0,00 min	End Time	: 42,00 min
Sampling Rate	: 12,5000 pts/s		
Sample Volume	: 1,000000 ul		
Sample Amount	: 1,0000	Area Reject	: 0,000000
Data Acquisition Time	: 2015.07.17. 12:06:25	Dilution Factor	: 1,00
		Cycle	: 1

Raw Data File : E:\GC\adatok\Zsolt\UGI 252.raw
Result File : E:\GC\adatok\Zsolt\UGI 252.rst [Editing in Progress]
Inst Method : D:\Doksik\Irodalom\GC\method\Zsolt\fenipropanol90 from E:\GC\adatok\Zsolt\UGI 252.raw
Proc Method : D:\Doksik\Irodalom\GC\method\Zsolt\fenipropanol90 from E:\GC\adatok\Zsolt\UGI 252.rst [Editing
Calib Method : D:\Doksik\Irodalom\GC\method\Zsolt\fenipropanol90 from E:\GC\adatok\Zsolt\UGI 252.rst [Editing
Report Format File: D:\Doksik\Irodalom\GC\method\Zsolt\fenipropanol90.rpt
Sequence File : D:\Doksik\Irodalom\GC\adatok\UGI 252.seq

DEFAULT REPORT

Peak #	Time [min]	Area [µV·s]	Height [µV]	Area [%]	Norm. Area [%]	BL	Area/Height [s]
1	25,349	5672,12	1197,12	9,58	9,58	*MM	4,7381
2	25,731	53560,85	11156,51	90,42	90,42	*MM	4,8009
		59232,97	12353,63	100,00	100,00		

Catalyst: **10b**

Figure S 149: Chiral GC chromatogram of OAc 1-phenyl-1-propanol enantiomers

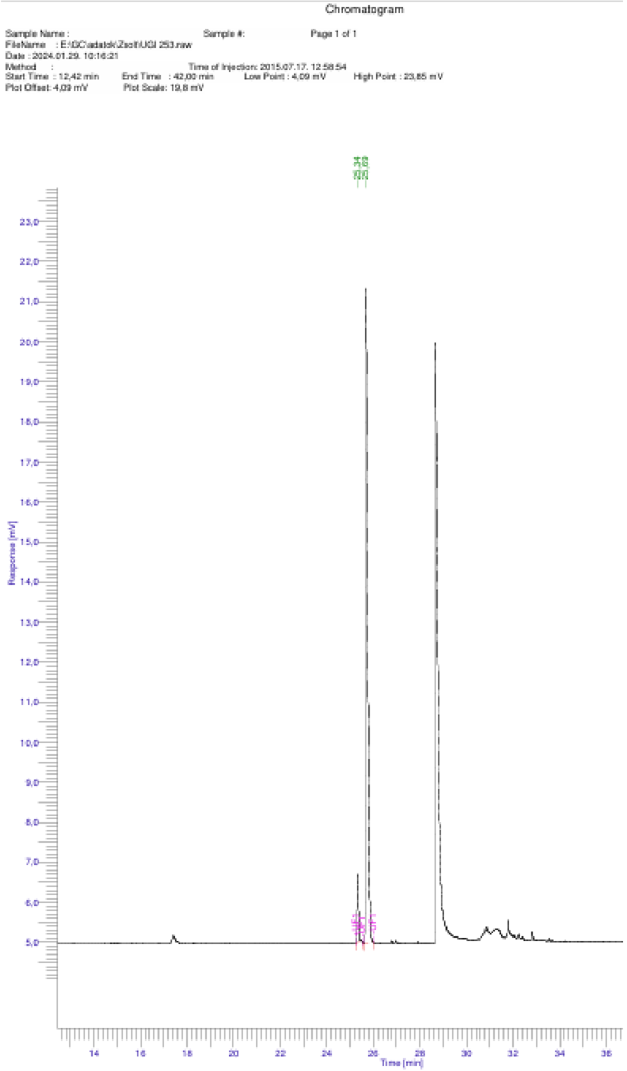

Software Version	: 6.3.2.0646		Date	: 2024.01.29. 10:16:54
Operator	: manager		Sample Name	:
Sample Number	:		Study	:
AutoSampler	: NONE		Rack/Vial	: 0/0
Instrument Name	: PerkinName		Channel	: A
Instrument Serial #	: 610N9042106		A/D mV Range	: 1000
Delay Time	: 0,00 min		End Time	: 42,00 min
Sampling Rate	: 12,5000 pts/s			
Sample Volume	: 1,000000 ul			
Sample Amount	: 1,0000		Area Reject	: 0,000000
Data Acquisition Time	: 2015.07.17. 12:58:54		Dilution Factor	: 1,00
			Cycle	: 1

Raw Data File : E:\GC\adatok\Zsolt\UGI 253.raw
Result File : E:\GC\adatok\Zsolt\UGI 253.rst [Editing in Progress]
Inst Method : D:\Doksik\Irodalom\GC\method\Zsolt\fenipropanol90 from E:\GC\adatok\Zsolt\UGI 253.raw
Proc Method : D:\Doksik\Irodalom\GC\method\Zsolt\fenipropanol90 from E:\GC\adatok\Zsolt\UGI 253.rst [Editing i
Calib Method : D:\Doksik\Irodalom\GC\method\Zsolt\fenipropanol90 from E:\GC\adatok\Zsolt\UGI 253.rst [Editing
Report Format File: D:\Doksik\Irodalom\GC\method\Zsolt\fenipropanol90.rpt
Sequence File : D:\Doksik\Irodalom\GC\adatok\UGI 253.seq

DEFAULT REPORT

Peak #	Time [min]	Area [µV·s]	Height [µV]	Area [%]	Norm. Area [%]	BL	Area/Height [s]
1	25,340	7465,87	1709,15	6,85	6,85	*MM	4,3682
2	25,692	101501,55	16338,34	93,15	93,15	*MM	6,2125
		108967,41	18047,49	100,00	100,00		

Missing Component Report

Catalyst: **10c**

Figure S 150: Chiral GC chromatogram of OAc 1-phenyl-1-propanol enantiomers

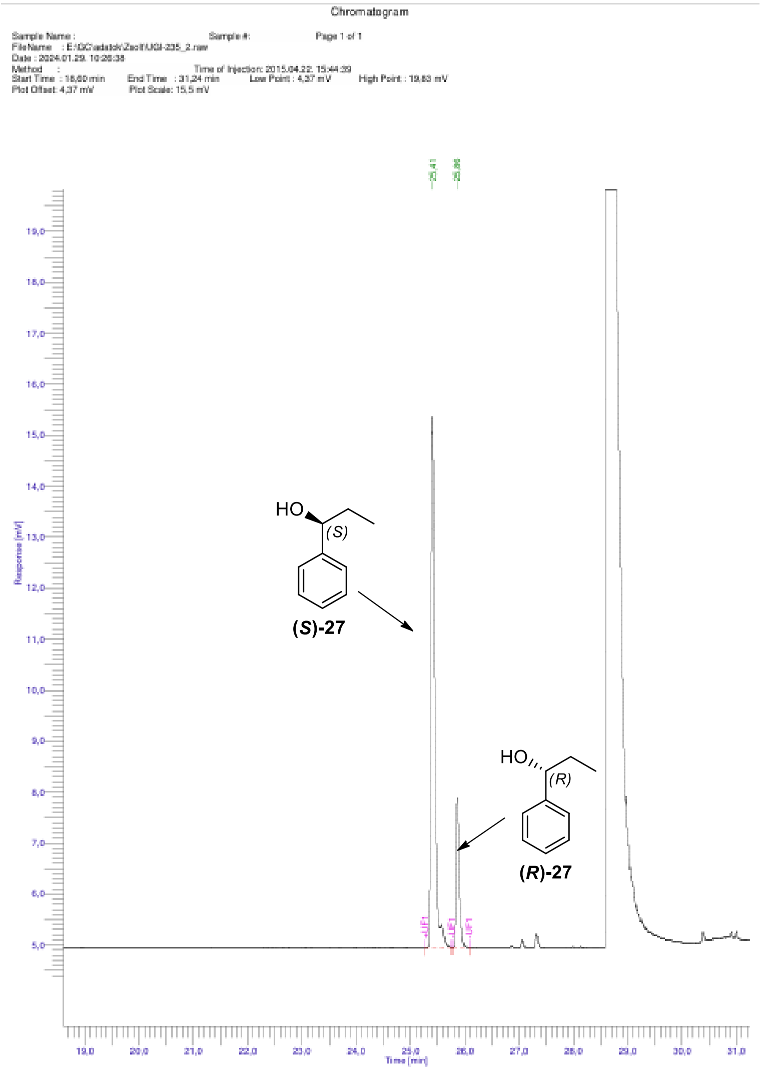

Software Version	: 6.3.2.0646	Date	: 2024.01.29. 10:25:17
Operator	: manager	Sample Name	:
Sample Number	:	Study	:
AutoSampler	: NONE	Rack/Vial	: 0/0
Instrument Name	: PerkinName	Channel	: A
Instrument Serial #	: 610N9042106	A/D mV Range	: 1000
Delay Time	: 0,00 min	End Time	: 42,00 min
Sampling Rate	: 12,5000 pts/s		
Sample Volume	: 1,000000 ul	Area Reject	: 0,000000
Sample Amount	: 1,0000	Dilution Factor	: 1,00
Data Acquisition Time	: 2015.04.22. 15:44:39	Cycle	: 1

Raw Data File : E:\GC\adatok\Zsolt\UGI-235_2.raw
Result File : E:\GC\adatok\Zsolt\UGI-235_2.rst [Editing in Progress]
Inst Method : D:\Doksik\Irodalom\GC\method\Zsolt\fenipropanol90 from E:\GC\adatok\Zsolt\UGI-235_2.raw
Proc Method : D:\Doksik\Irodalom\GC\method\Zsolt\fenipropanol90 from E:\GC\adatok\Zsolt\UGI-235_2.rst [Editing
Calib Method : D:\Doksik\Irodalom\GC\method\Zsolt\fenipropanol90 from E:\GC\adatok\Zsolt\UGI-235_2.rst [Editing
Report Format File: D:\Doksik\Irodalom\GC\method\Zsolt\fenipropanol90.rpt
Sequence File : D:\Doksik\Irodalom\GC\adatok\UGI-235_2.seq

DEFAULT REPORT

Peak #	Time [min]	Area [µV·s]	Height [µV]	Area [%]	Norm. Area [%]	BL	Area/Height [s]
1	25,409	49972,07	10420,99	80,05	80,05	*MM	4,7953
2	25,864	12452,16	2928,76	19,95	19,95	*MM	4,2517
		62424,23	13349,74	100,00	100,00		

Catalyst: **7b**

Figure S 151: Chiral GC chromatogram of OAc 1-phenyl-1-propanol enantiomers

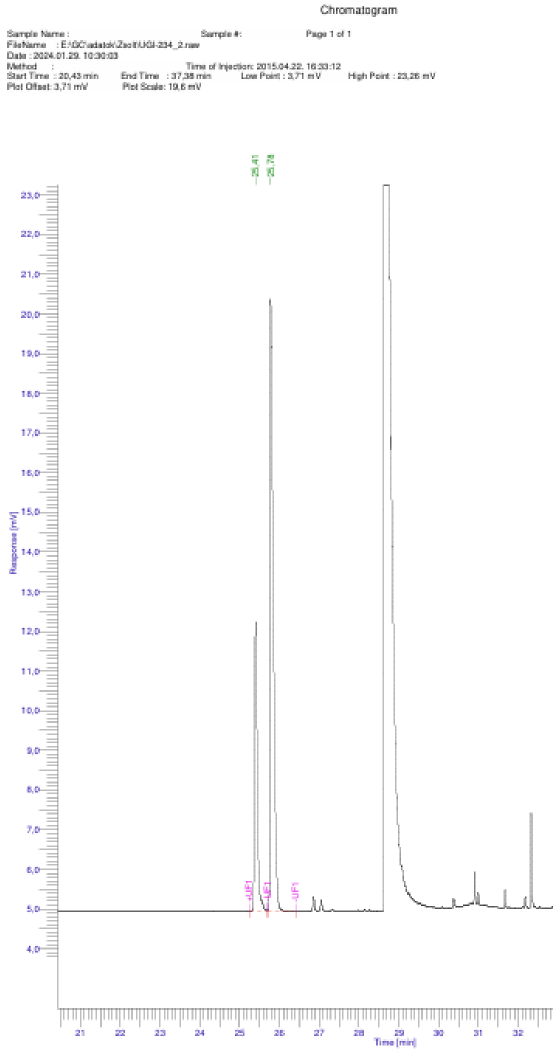

Software Version	: 6.3.2.0646		Date	: 2024.01.29. 10:23:09
Operator	: manager		Sample Name	:
Sample Number	:		Study	:
AutoSampler	: NONE		Rack/Vial	: 0/0
Instrument Name	: PerkinName		Channel	: A
Instrument Serial #	: 610N9042106		A/D mV Range	: 1000
Delay Time	: 0,00 min		End Time	: 42,00 min
Sampling Rate	: 12,5000 pts/s			
Sample Volume	: 1,000000 ul		Area Reject	: 0,000000
Sample Amount	: 1,0000		Dilution Factor	: 1,00
Data Acquisition Time	: 2015.04.22. 16:33:12		Cycle	: 1

Raw Data File : E:\GC\adatok\Zsolt\UGI-234_2.raw
Result File : E:\GC\adatok\Zsolt\UGI-234_2.rst [Editing in Progress]
Inst Method : D:\Doksik\Irodalom\GC\method\Zsolt\fenipropanol90 from E:\GC\adatok\Zsolt\UGI-234_2.raw
Proc Method : D:\Doksik\Irodalom\GC\method\Zsolt\fenipropanol90 from E:\GC\adatok\Zsolt\UGI-234_2.rst [Editi
Calib Method : D:\Doksik\Irodalom\GC\method\Zsolt\fenipropanol90 from E:\GC\adatok\Zsolt\UGI-234_2.rst [Edit
Report Format File: D:\Doksik\Irodalom\GC\method\Zsolt\fenipropanol90.rpt
Sequence File : D:\Doksik\Irodalom\GC\adatok\UGI-234_2.seq

DEFAULT REPORT

Peak #	Time [min]	Area [µV·s]	Height [µV]	Area [%]	Norm. Area [%]	BL	Area/Height [s]
1	25,405	33429,41	7284,76	28,93	28,93	*MM	4,5890
2	25,783	82121,21	15412,28	71,07	71,07	*MM	5,3283
		115550,61	22697,04	100,00	100,00		

Catalyst: **11c**

Figure S 152: Chiral GC chromatogram of OAc 1-phenyl-1-propanol enantiomers

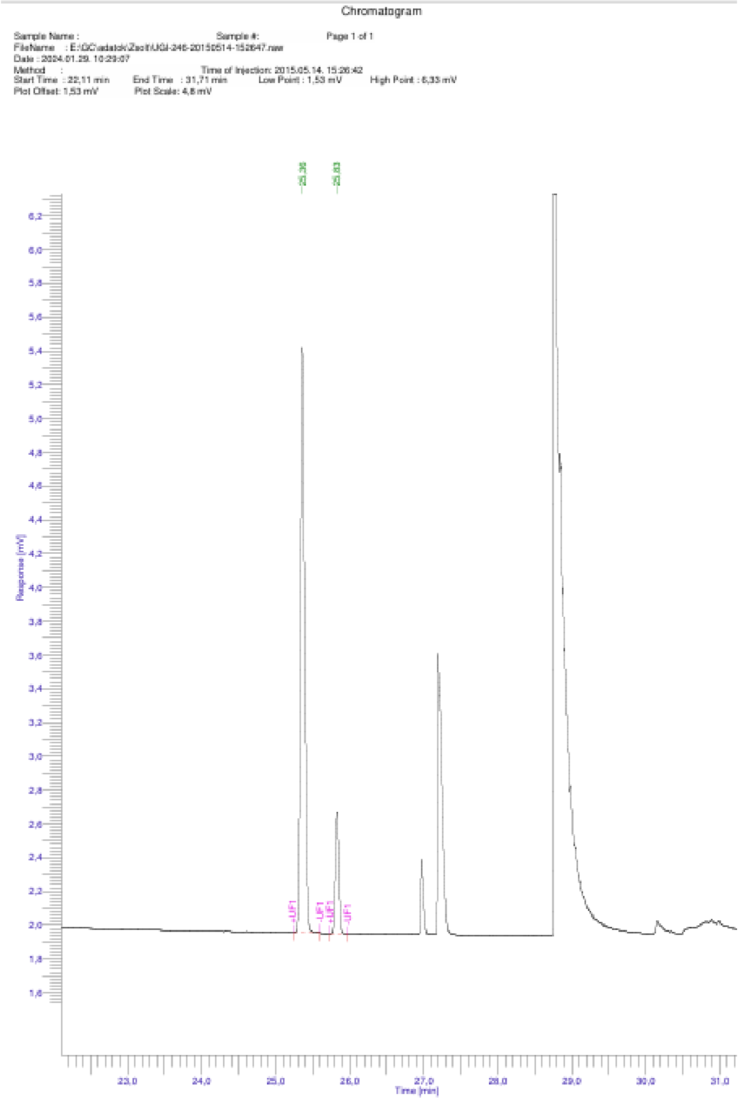

Software Version : 6.3.2.0646
Operator : manager
Sample Number :
AutoSampler : NONE
Instrument Name : PerkinName
Instrument Serial # : None
Delay Time : 0,00 min
Sampling Rate : 12,5000 pts/s
Sample Volume : 1,000000 ul
Sample Amount : 1,0000
Data Acquisition Time : 2015.05.14. 15:26:42

Date : 2024.01.29. 10:18:58
Sample Name :
Study :
Rack/Vial : 0/0
Channel : A
A/D mV Range : 1000
End Time : 42,00 min

Area Reject : 0,000000
Dilution Factor : 1,00
Cycle : 1

Raw Data File : E:\GC\adatok\Zsolt\UGI-246-20150514-152647.raw
Result File : E:\GC\adatok\Zsolt\UGI-246.rst [Editing in Progress]
Inst Method : D:\Doksik\Irodalom\GC\method\Zsolt\fenipropanol90 from E:\GC\adatok\Zsolt\UGI-246-20150514-1!
Proc Method : D:\Doksik\Irodalom\GC\method\Zsolt\fenipropanol90 from E:\GC\adatok\Zsolt\UGI-246.rst [Editing
Calib Method : D:\Doksik\Irodalom\GC\method\Zsolt\fenipropanol90 from E:\GC\adatok\Zsolt\UGI-246.rst [Editing
Report Format File: D:\Doksik\Irodalom\GC\method\Zsolt\fenipropanol90.rpt
Sequence File : D:\Doksik\Irodalom\GC\adatok\UGI-246.seq

DEFAULT REPORT

Peak #	Time [min]	Area [µV·s]	Height [µV]	Area [%]	Norm. Area [%]	BL	Area/Height [s]
1	25,359	13654,62	3468,80	85,16	85,16	*MM	3,9364
2	25,828	2380,09	722,38	14,84	14,84	*MM	3,2948
		16034,71	4191,17	100,00	100,00		

Catalyst: **8**

Figure S 153: Chiral GC chromatogram of OAc 1-phenyl-1-propanol enantiomers

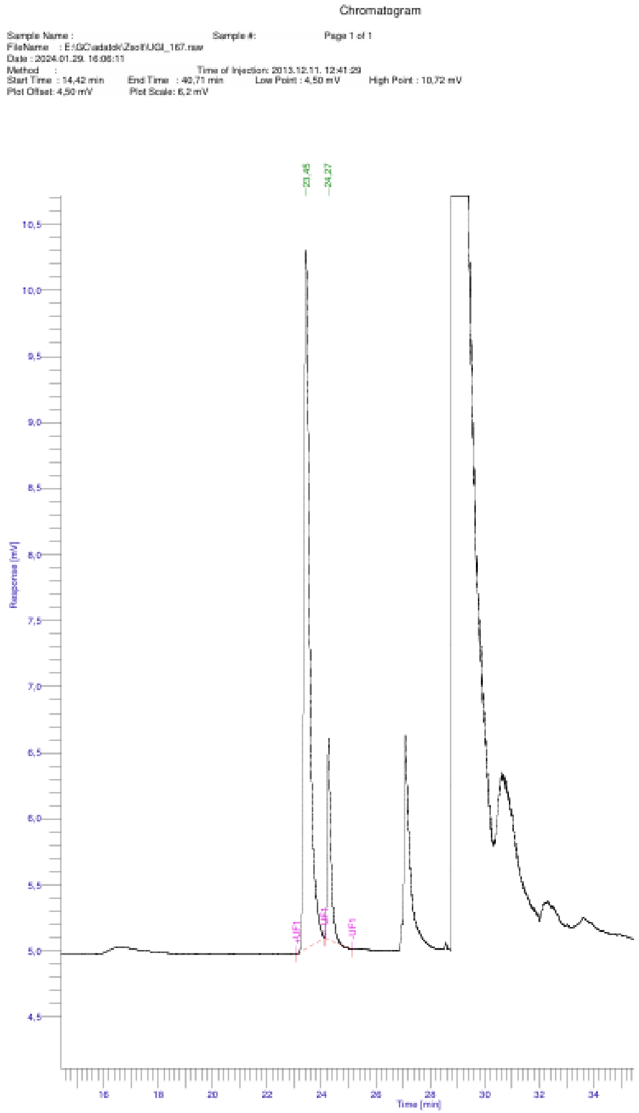

Software Version	: 6.3.2.0646	Date	: 2024.01.29. 16:06:30
Operator	: manager	Sample Name	:
Sample Number	:	Study	:
AutoSampler	: NONE	Rack/Vial	: 0/0
Instrument Name	: PerkinName	Channel	: A
Instrument Serial #	: 610N9042106	A/D mV Range	: 1000
Delay Time	: 0,00 min	End Time	: 42,00 min
Sampling Rate	: 12,5000 pts/s		
Sample Volume	: 1,000000 ul	Area Reject	: 0,000000
Sample Amount	: 1,0000	Dilution Factor	: 1,00
Data Acquisition Time	: 2013.12.11. 12:41:29	Cycle	: 1

Raw Data File : E:\GC\adatok\Zsolt\UGI_167.raw
Result File : E:\GC\adatok\Zsolt\UGI_167.rst [Editing in Progress]
Inst Method : D:\Doksik\Irodalom\GC\method\Zsolt\fenipropanol90 from E:\GC\adatok\Zsolt\UGI_167.raw
Proc Method : D:\Doksik\Irodalom\GC\method\Zsolt\fenipropanol90 from E:\GC\adatok\Zsolt\UGI_167.rst [Editing
Calib Method : D:\Doksik\Irodalom\GC\method\Zsolt\fenipropanol90 from E:\GC\adatok\Zsolt\UGI_167.rst [Editing
Report Format File: D:\Doksik\Irodalom\GC\method\Zsolt\fenipropanol90.rpt
Sequence File : D:\Doksik\Irodalom\GC\adatok\UGI_167.seq

DEFAULT REPORT

Peak #	Time [min]	Area [µV·s]	Height [µV]	Area [%]	Norm. Area [%]	BL	Area/Height [s]
1	23,449	75748,25	5286,24	84,33	84,33	*MM	14,3293
2	24,272	14079,15	1525,55	15,67	15,67	*MM	9,2289
		89827,39	6811,78	100,00	100,00		

Catalyst: **7a**

Figure S 154: Chiral GC chromatogram of OAc 1-phenyl-1-propanol enantiomers

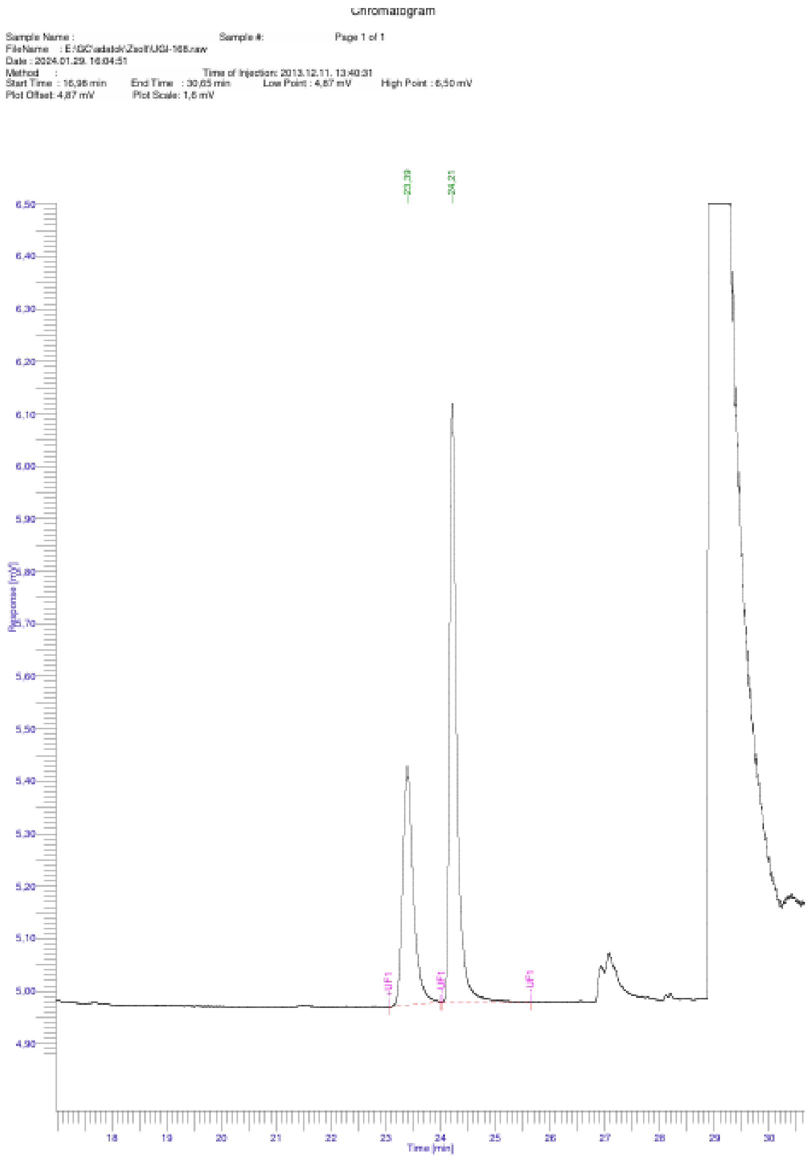

Software Version	: 6.3.2.0646		Date	: 2024.01.29. 16:05:24
Operator	: manager		Sample Name	:
Sample Number	:		Study	:
AutoSampler	: NONE		Rack/Vial	: 0/0
Instrument Name	: PerkinName		Channel	: A
Instrument Serial #	: 610N9042106		A/D mV Range	: 1000
Delay Time	: 0,00 min		End Time	: 42,00 min
Sampling Rate	: 12,5000 pts/s			
Sample Volume	: 1,000000 ul			
Sample Amount	: 1,0000		Area Reject	: 0,000000
Data Acquisition Time	: 2013.12.11. 13:40:31		Dilution Factor	: 1,00
			Cycle	: 1

Raw Data File : E:\GC\adatok\Zsolt\UGI-168.raw
Result File : E:\GC\adatok\Zsolt\UGI-168.rst [Editing in Progress]
Inst Method : D:\Doksik\Irodalom\GC\method\Zsolt\fenipropanol90 from E:\GC\adatok\Zsolt\UGI-168.raw
Proc Method : D:\Doksik\Irodalom\GC\method\Zsolt\fenipropanol90 from E:\GC\adatok\Zsolt\UGI-168.rst [Editing i
Calib Method : D:\Doksik\Irodalom\GC\method\Zsolt\fenipropanol90 from E:\GC\adatok\Zsolt\UGI-168.rst [Editing
Report Format File: D:\Doksik\Irodalom\GC\method\Zsolt\fenipropanol90.rpt
Sequence File : D:\Doksik\Irodalom\GC\adatok\UGI-168.seq

DEFAULT REPORT

Peak #	Time [min]	Area [µV·s]	Height [µV]	Area [%]	Norm. Area [%]	BL	Area/Height [s]
1	23,393	6112,60	454,93	36,95	36,95	*MM	13,4365
2	24,211	10432,39	1142,32	63,05	63,05	*MM	9,1326
		16544,99	1597,24	100,00	100,00		

Catalyst: **11a**

Figure S 155: Chiral GC chromatogram of OAc 1-phenyl-1-propanol enantiomers

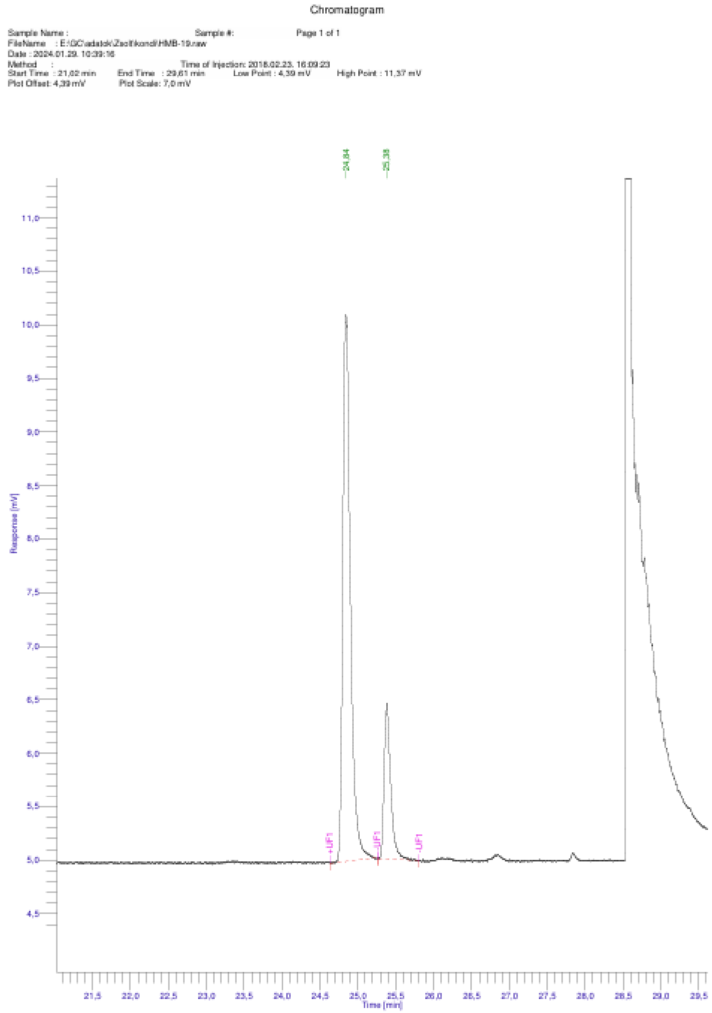

Software Version	: 6.3.2.0646	Date	: 2024.01.29. 10:39:39
Operator	: manager	Sample Name	:
Sample Number	:	Study	:
AutoSampler	: NONE	Rack/Vial	: 0/0
Instrument Name	: AutosystemXL	Channel	: A
Instrument Serial #	: 610N9042106	A/D mV Range	: 1000
Delay Time	: 0,00 min	End Time	: 34,30 min
Sampling Rate	: 12,5000 pts/s		
Sample Volume	: 1,000000 ul	Area Reject	: 0,000000
Sample Amount	: 1,0000	Dilution Factor	: 1,00
Data Acquisition Time	: 2018.02.23. 16:09:23	Cycle	: 1

Raw Data File : E:\GC\adatok\Zsolt\kondi\HMB-19.raw
Result File : E:\GC\adatok\Zsolt\kondi\HMB-19.rst [Editing in Progress]
Inst Method : E:\GC\method\Zsolt\fenipropanol90new from E:\GC\adatok\Zsolt\kondi\HMB-19.raw
Proc Method : E:\GC\method\Zsolt\fenipropanol90new from E:\GC\adatok\Zsolt\kondi\HMB-19.rst [Editing in Progress]
Calib Method : E:\GC\method\Zsolt\fenipropanol90new from E:\GC\adatok\Zsolt\kondi\HMB-19.rst [Editing in Progress]
Report Format File: E:\GC\method\Zsolt\fenipropanol90new.rpt
Sequence File : E:\GC\method\HMB-19.seq

DEFAULT REPORT

Peak #	Time [min]	Area [µV·s]	Height [µV]	Area [%]	Norm. Area [%]	BL	Area/Height [s]
1	24,841	35360,30	5105,23	80,41	80,41	*MM	6,9263
2	25,381	8617,43	1456,08	19,59	19,59	*MM	5,9182
		43977,73	6561,32	100,00	100,00		

Catalyst: **21b**

Figure S 156: Chiral GC chromatogram of OAc 1-phenyl-1-propanol enantiomers

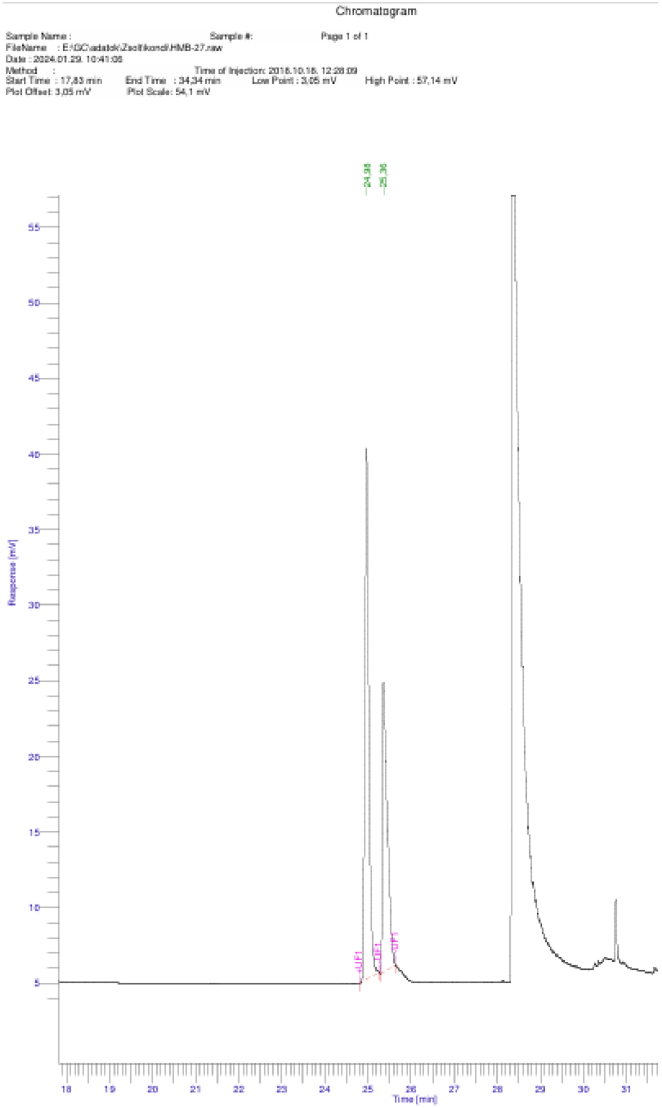

Software Version	: 6.3.2.0646		Date	: 2024.01.29. 10:41:32
Operator	: manager		Sample Name	:
Sample Number	:		Study	:
AutoSampler	: NONE		Rack/Vial	: 0/0
Instrument Name	: AutosystemXL		Channel	: A
Instrument Serial #	: None		A/D mV Range	: 1000
Delay Time	: 0,00 min		End Time	: 47,00 min
Sampling Rate	: 12,5000 pts/s			
Sample Volume	: 1,000000 ul			
Sample Amount	: 1,0000		Area Reject	: 0,000000
Data Acquisition Time	: 2018.10.18. 12:28:09		Dilution Factor	: 1,00
			Cycle	: 1

Raw Data File : E:\GC\adatok\Zsolt\kondi\HMB-27.raw
Result File : E:\GC\adatok\Zsolt\kondi\HMB-27.rst [Editing in Progress]
Inst Method : E:\GC\method\Zsolt\fenipropanol90new from E:\GC\adatok\Zsolt\kondi\HMB-27.raw
Proc Method : E:\GC\method\Zsolt\fenipropanol90new from E:\GC\adatok\Zsolt\kondi\HMB-27.rst [Editing in Prog
Calib Method : E:\GC\method\Zsolt\fenipropanol90new from E:\GC\adatok\Zsolt\kondi\HMB-27.rst [Editing in Prog
Report Format File: E:\GC\method\Zsolt\fenipropanol90new.rpt
Sequence File : E:\GC\method\HMB-27.seq

DEFAULT REPORT

Peak #	Time [min]	Area [µV·s]	Height [µV]	Area [%]	Norm. Area [%]	BL	Area/Height [s]
1	24,977	227863,70	35111,49	60,39	60,39	*MM	6,4897
2	25,360	149439,56	19118,44	39,61	39,61	*MM	7,8165
		377303,27	54229,93	100,00	100,00		

Catalyst: **22**

Figure S 157: Chiral HPLC chromatogram of 1-(*p*-tolyl)-1-propanol enantiomers

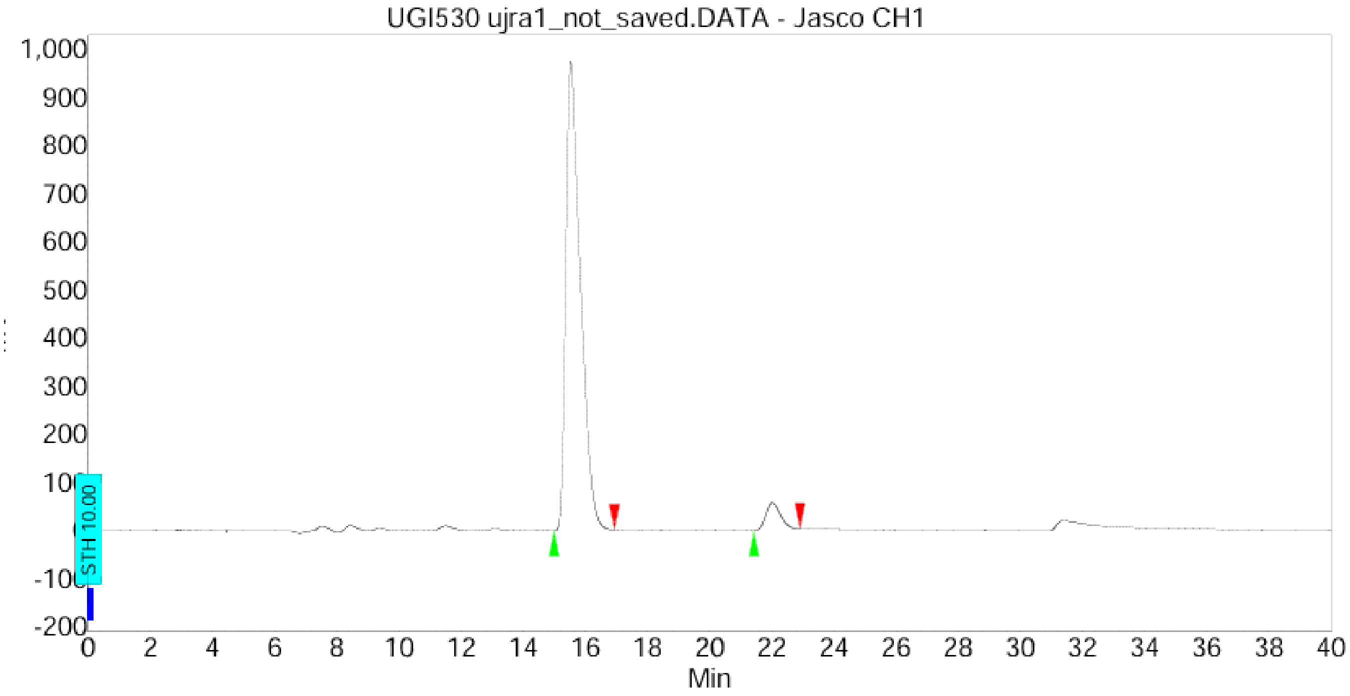

Peak results :

Index	Name	Time [Min]	Quantity [% Area]	Height [mV]	Area [mV.Min]	Area % [%]
1	UNKNOWN	15.532	94.51	973.6	529.3	94.510
2	UNKNOWN	22.017	5.49	56.7	30.7	5.490
Total			100.00	1030.3	560.1	100.000

Catalyst: **7a**

Figure S 158: Chiral HPLC chromatogram of 1-(*p*-tolyl)-1-propanol enantiomers

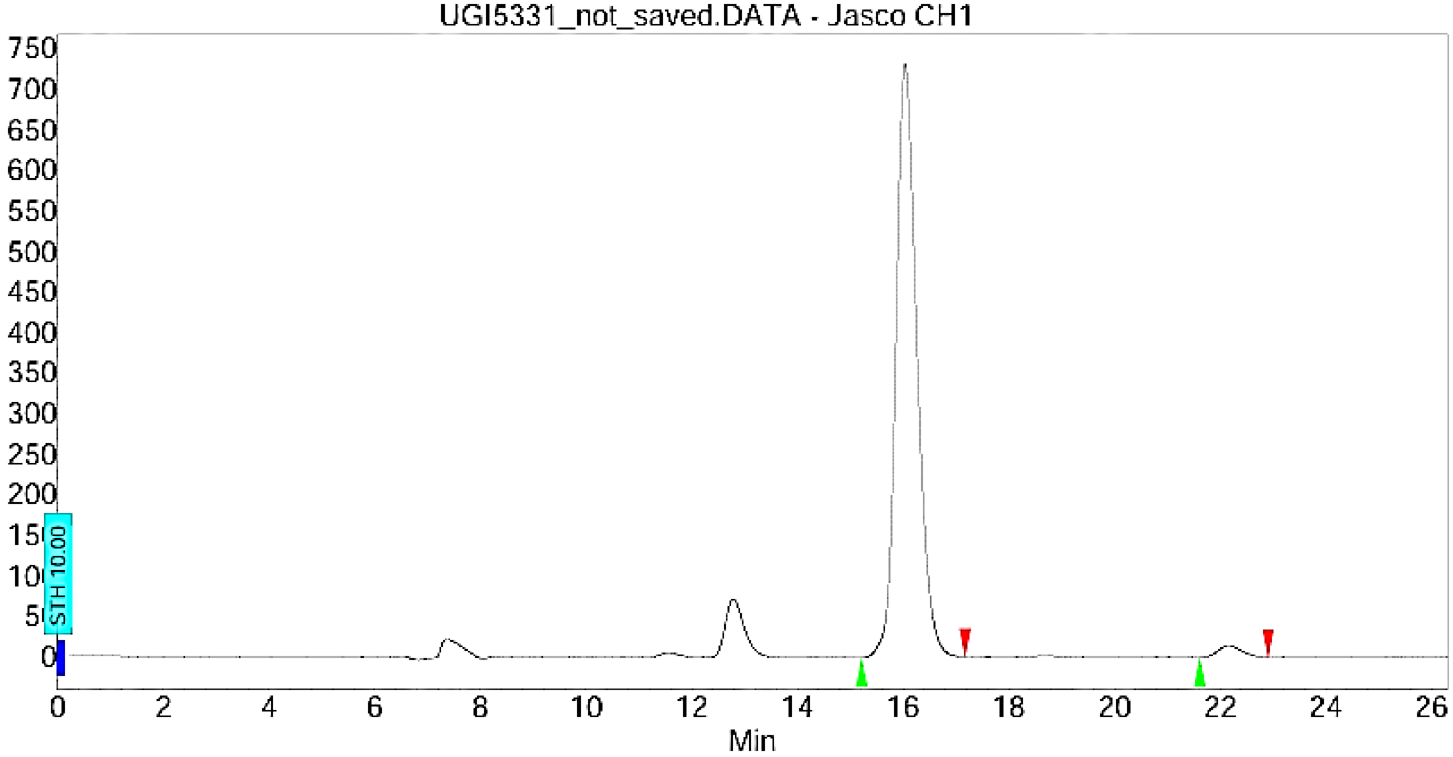

Peak results :

Index	Name	Time [Min]	Quantity [% Area]	Height [mV]	Area [mV.Min]	Area % [%]
1	UNKNOWN	16.037	97.77	731.5	336.2	97.771
2	UNKNOWN	22.163	2.23	14.3	7.7	2.229
Total			100.00	745.8	343.9	100.000

Catalyst: **10a**

Figure S 159: Chiral HPLC chromatogram of 1-(*p*-methoxyphenyl)-1-propanol enantiomers

Chromatogram : UGI5311_channel1

System : PrepiHPLC
Method : UGI2
User : User1

Acquired : 3/27/2024 1:05:09 PM
Processed : 3/27/2024 1:41:45 PM
Printed : 4/5/2024 12:17:44 PM

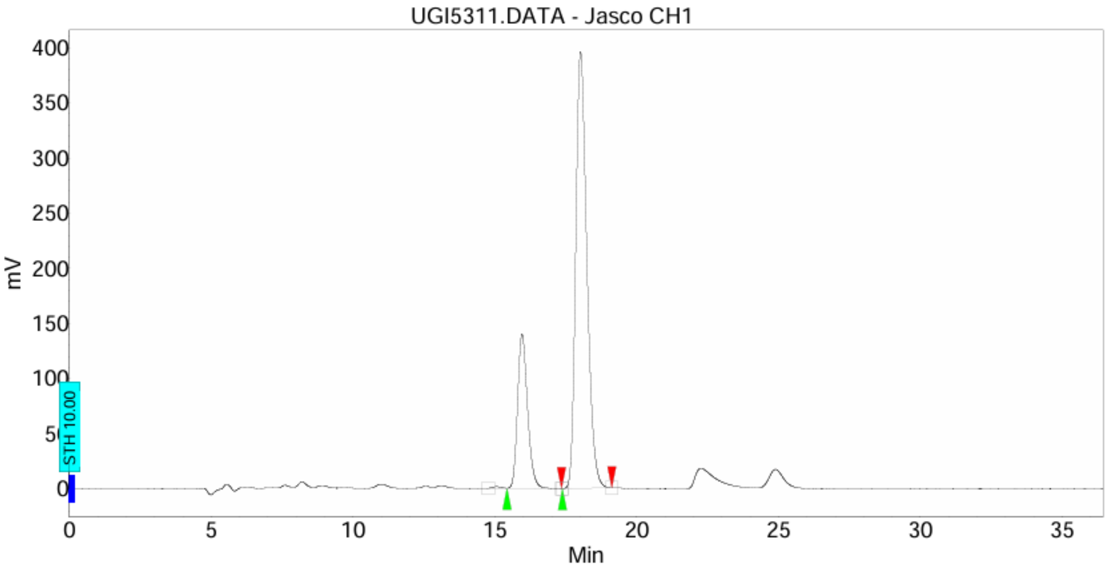

Peak results :

Index	Name	Time [Min]	Quantity [% Area]	Height [mV]	Area [mV.Min]	Area % [%]
1	UNKNOWN	15.953	24.07	139.6	56.8	24.066
2	UNKNOWN	18.017	75.93	395.3	179.3	75.934
Total			100.00	534.9	236.2	100.000

Catalyst: **7a**

Figure S 160: Chiral HPLC chromatogram of 1-(*p*-methoxyphenyl)-1-propanol enantiomers

Chromatogram : UGI5341_channel1

System : PrepiHPLC
Method : UGI2
User : User1

Acquired : 4/3/2024 3:04:53 PM
Processed : 4/4/2024 1:38:21 PM
Printed : 4/5/2024 12:15:37 PM

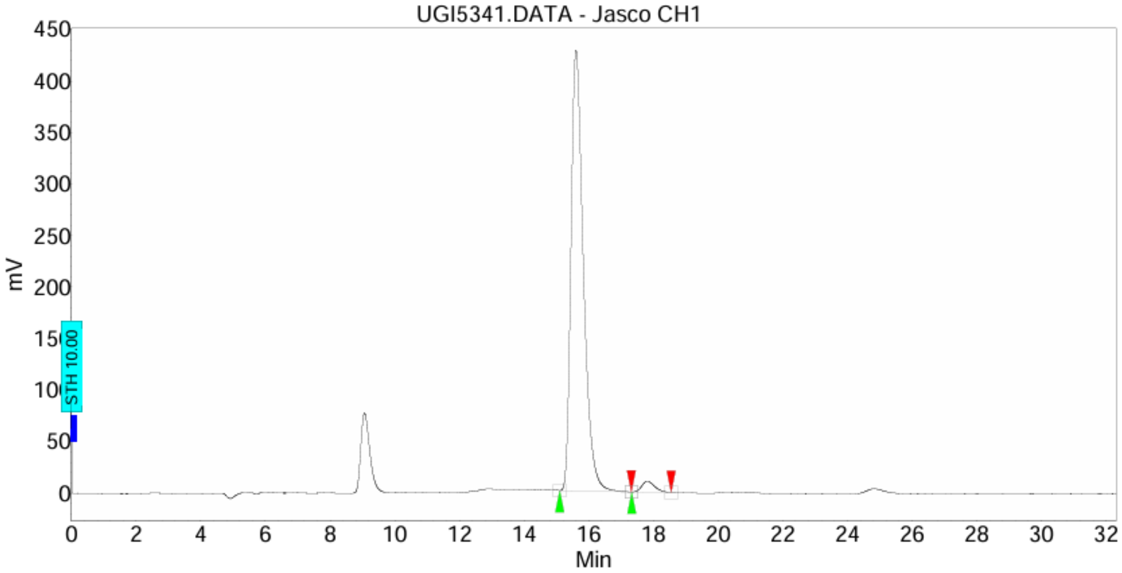

Peak results :

Index	Name	Time [Min]	Quantity [% Area]	Height [mV]	Area [mV.Min]	Area % [%]
1	UNKNOWN	15.607	97.51	427.2	189.8	97.505
2	UNKNOWN	17.817	2.49	10.5	4.9	2.495
Total			100.00	437.7	194.6	100.000

Catalyst: **10a**

Figure S 161: Chiral HPLC chromatogram of 1-(*m*-methoxyphenyl)-1-propanol enantiomers

Chromatogram : UGI5321_channel1

System : PrepiHPLC
Method : UGI3
User : User1

Acquired : 3/28/2024 10:30:59 AM
Processed : 3/28/2024 11:55:55 AM
Printed : 4/5/2024 12:23:20 PM

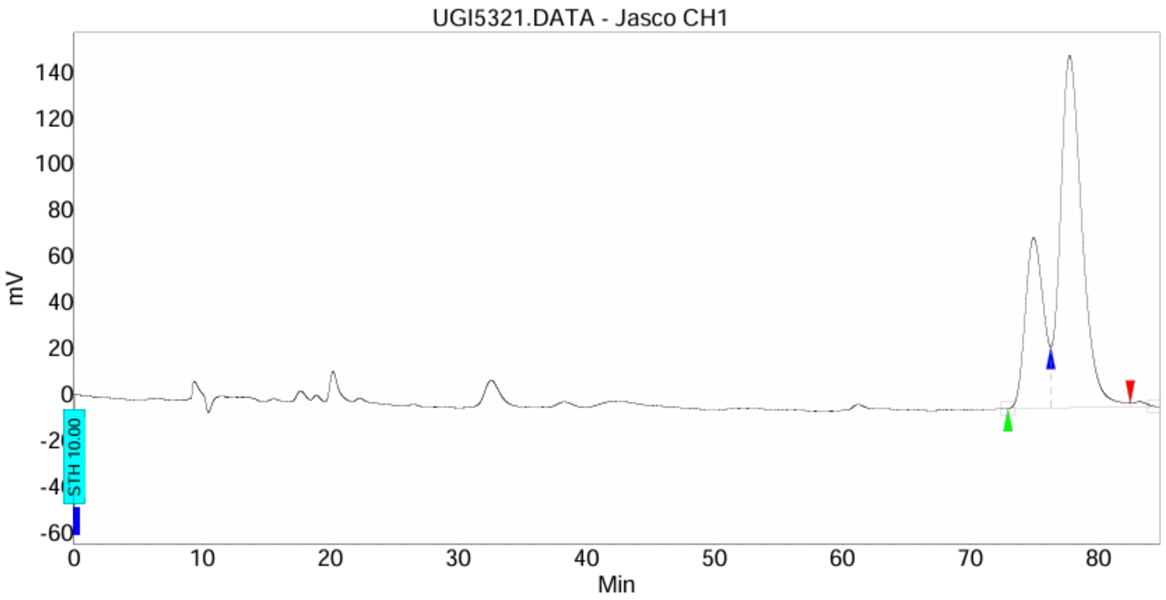

Peak results :

Index	Name	Time [Min]	Quantity [% Area]	Height [mV]	Area [mV.Min]	Area % [%]
1	UNKNOWN	74.943	28.83	74.0	122.5	28.830
2	UNKNOWN	77.752	71.17	152.7	302.3	71.170
Total			100.00	226.7	424.7	100.000

Catalyst: **7a**

Figure S 162: Chiral HPLC chromatogram of 1-(*m*-methoxyphenyl)-1-propanol enantiomers

Chromatogram : UGI535 1_channel1

System : PrepiHPLC
Method : UGI3mod2
User : User1

Acquired : 4/4/2024 4:25:40 PM
Processed : 4/5/2024 4:58:35 PM
Printed : 4/5/2024 5:07:32 PM

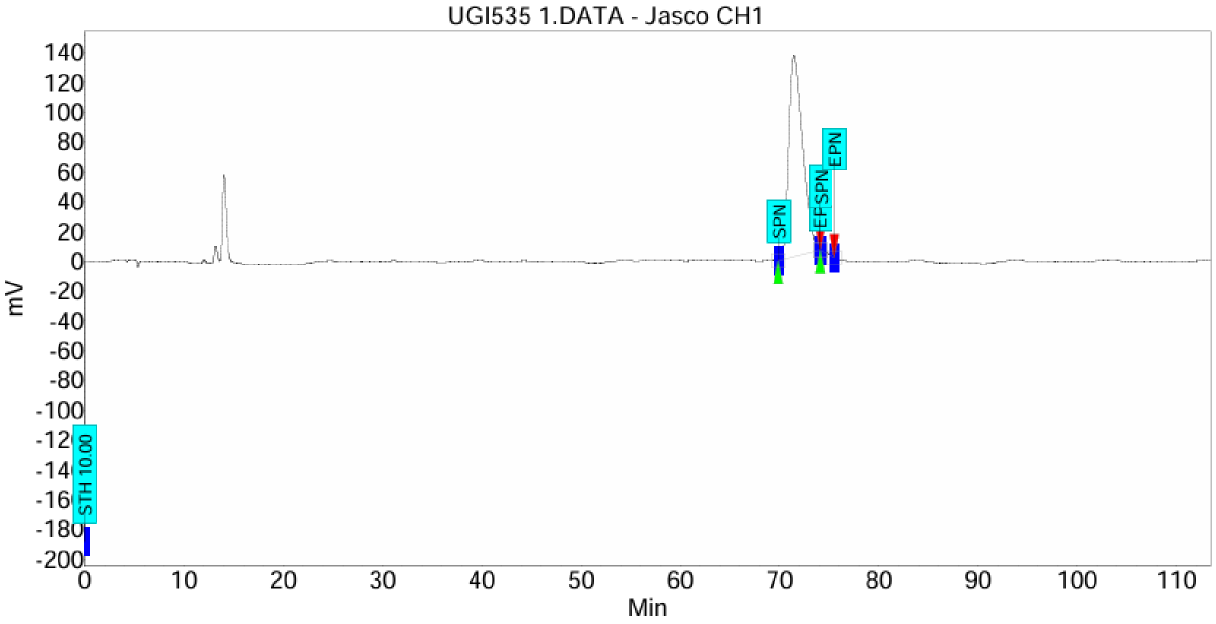

Peak results :

Index	Name	Time [Min]	Quantity [% Area]	Height [mV]	Area [mV.Min]	Area % [%]
1	UNKNOWN	71.432	99.83	135.2	221.3	99.829
2	UNKNOWN	74.762	0.17	0.6	0.4	0.171
Total			100.00	135.7	221.7	100.000

Catalyst: **10a**
